# Homelessness, Patient Navigation, and Lung Cancer Screening in a Health Center Setting: A Subgroup Analysis of a Randomized Clinical Trial

**DOI:** 10.1001/jamanetworkopen.2025.19780

**Published:** 2025-07-17

**Authors:** Travis P. Baggett, Nora Sporn, Joana Barbosa Teixeira, Elijah C. Rodriguez, Nillani Anandakugan, Bailey R. Little, Yuchiao Chang, Elyse R. Park, Nancy A. Rigotti, Danielle R. Fine

**Affiliations:** 1 Division of General Internal Medicine, Massachusetts General Hospital, Boston; 2 Mongan Institute, Massachusetts General Hospital, Boston; 3 Harvard Medical School, Boston, Massachusetts; 4 Institute for Research, Quality, and Policy in Homeless Health Care, Boston Health Care for the Homeless Program, Boston, Massachusetts; 5 Ragon Institute, Boston, Massachusetts; 6 New York University Grossman School of Medicine, New York; 7 University of Massachusetts Chan Medical School, Worcester

## Abstract

**Importance:**

Lung cancer is a major cause of death among people who experience homelessness. Patient navigation is an effective strategy for promoting lung cancer screening (LCS) in Health Care for the Homeless (HCH) settings, but little is known about whether the impact of this intervention differs for patients currently vs formerly experiencing homelessness.

**Objectives:**

To examine the effect of LCS patient navigation on individuals currently vs formerly experiencing homelessness, and to explore how navigation process measures differ for these subgroups.

**Design, Setting, and Participants:**

This is a subgroup analysis of the Investigating Navigation to Help Advance Lung Equity (INHALE) pragmatic randomized clinical trial of LCS patient navigation. The INHALE trial was conducted at Boston Health Care for the Homeless Program (BHCHP), a federally qualified health center serving nearly 10 000 patients who have experienced homelessness annually. The study included BHCHP primary care patients with a lifetime history of homelessness who were proficient in English and eligible for LCS under pre-2022 Medicare coverage criteria. The study was conducted between November 20, 2020, and March 29, 2023.

**Exposure:**

Current vs former homelessness, defined by self-reported responses to a detailed residential inventory. Sensitivity analyses further categorized individuals who formerly experienced homelessness as having stable or unstable housing.

**Main Outcomes and Measures:**

The primary outcome was verified receipt of a 1-time LCS low-dose computed tomography (LDCT) scan within 6 months after randomization. The risk difference (RD) in primary outcome attainment between navigation and usual care within each homelessness subgroup was calculated, and these RDs were compared by testing the interaction between study group and homelessness status in a linear binomial regression model with the identity link.

**Results:**

This study included 260 participants (mean [SD] age, 60.5 [4.7] years; 184 male individuals [70.8%]). At baseline, 84 patients (32.3%) were currently experiencing homelessness and 176 (67.7%) had formerly experienced homelessness. Patient navigation significantly increased LCS LDCT completion among both those currently (15 of 56 [26.8%] vs 2 of 28 [7.1%]; *P* = .04) and formerly (60 of 117 [51.3%] vs 6 of 59 [10.2%]; *P* < .001) experiencing homelessness. However, the treatment effect was significantly smaller among participants currently experiencing homelessness (RD, 19.7% vs 41.1%; *P* = .03), such that a disparity in LCS completion between these subgroups emerged under the navigation condition. Navigation process measures highlighted communication challenges with participants currently experiencing homelessness. In sensitivity analyses, LCS LDCT completion rates and navigation process measures were generally similar for stably vs unstably housed participants who formerly experienced homelessness.

**Conclusions and Relevance:**

In this subgroup analysis of a randomized clinical trial, patient navigation increased LCS participation among both patients currently and formerly experiencing homelessness; however, the effect size was smaller for those currently experiencing homelessness. Further improving cancer outcomes among HCH patients may require refinement of the patient navigation intervention, coupled with policy efforts to promote housing attainment among people experiencing homelessness.

**Trial Registration:**

ClinicalTrials.gov Identifier: NCT04308226

## Key Points

**Question:**

Does the effect of patient navigation on lung cancer screening completion differ for patients currently vs formerly experiencing homelessness?

**Findings:**

In this subgroup analysis of a randomized clinical trial involving 260 participants, patient navigation significantly improved lung cancer screening completion among both those currently (20% absolute increase) and formerly (41% absolute increase) experiencing homelessness, but the effect size was significantly smaller for those currently experiencing homelessness. Communication barriers likely underpinned this differential effect.

**Meaning:**

These findings suggest that optimizing screening participation among patients who have experienced homelessness may require further tailoring of the navigation intervention and policy efforts to promote housing for people who are currently experiencing homelessness.

## Teaser

This subgroup analysis of a randomized clinical trial examines differences in the effects of patient navigation for lung cancer screening among individuals currently or formerly experiencing homelessness.

## Introduction

In 2022, more than 1.38 million people experienced homelessness in the US,^1^ and this number is rising.^2^ Cancer mortality rates are up to 88% higher among people experiencing homelessness than in the general population, with lung cancer incidence and mortality rates being more than 2-fold higher.^3,4,5,6,7,8^ In addition, multilevel barriers to cancer screening among individuals who have experienced homelessness^9^ contribute to suboptimal rates of screening participation^10,11,12,13,14,15^ and later-stage diagnoses of screen-detectable cancers compared with the general population.^8^

Patient navigation, a community-based service delivery intervention designed to eliminate barriers to timely care by guiding individuals through complex health systems,^16^ is a promising approach for reducing these cancer health disparities. Numerous studies have shown that patient navigation interventions improve cancer screening participation in a range of vulnerable populations.^17,18,19,20,21,22,23,24,25,26,27,28,29,30,31,32,33,34^ In 2020 to 2023, we extended this evidence base through a pragmatic trial, Investigating Navigation to Help Advance Lung Equity (INHALE), of patient navigation to promote lung cancer screening (LCS) among patients currently and formerly experiencing homelessness receiving primary care at a large Health Care for the Homeless (HCH) program in Boston, Massachusetts.^35^ At 6 months, participants in the patient navigation group were 4.7-fold more likely than those receiving usual care to have completed an LCS low-dose computed tomography (LDCT) scan.^36^

In this follow-up analysis, we used data from the INHALE pragmatic trial to (1) examine whether the effect of the LCS patient navigation intervention differed between participants currently vs formerly experiencing homelessness and (2) compare navigation process measures for both groups to uncover potential mechanisms underpinning differential treatment responses. The findings of this analysis could inform efforts to improve patient navigation outcomes and cancer screening participation among the considerable number of US community health center patients with current and prior experiences of homelessness and related housing difficulties.^37^

## Methods

### Study Design, Participants, and Setting

This is a prespecified subgroup analysis of the INHALE pragmatic randomized clinical trial of patient navigation for LCS conducted at Boston Health Care for the Homeless Program (BHCHP). The INHALE trial protocol has been published elsewhere and is presented in Supplement 1.^35^ The study was approved by the Mass General Brigham Institutional Review Board. Recruitment began on November 20, 2020, and data collection concluded on March 29, 2023. To be eligible to participate, individuals were required to have a lifetime history of homelessness, have a BHCHP primary care practitioner (PCP), be proficient in English, and meet the pre-2022 Medicare coverage criteria for LCS (ie, aged 55-77 years with at least a 30–pack-year smoking history and smoking within the past 15 years).^38^ We included patients currently and formerly experiencing homelessness because HCH programs often continue to care for patients who have experienced homelessness after they move into housing,^39^ and because the psychosocial complexities of homelessness often persist after housing is attained.^40^ All participants provided verbal informed consent. The study followed the Consolidated Standards of Reporting Trials (CONSORT) reporting guideline.

We randomized participants 2:1 to usual care with or without patient navigation, with randomization stratified by 4 self-reported measures: homelessness status (current vs former), smoking status (current vs former), prior discussion of LCS with their PCP (yes vs no), and primary clinical site where they see their PCP (BHCHP headquarters vs satellite sites). We stratified randomization to ensure balance on these variables because we hypothesized that they would influence study outcomes. All participants had access to usual care at BHCHP, which provides highly tailored, comprehensive, multidisciplinary homeless health care services through a network of shelter, hospital, and outreach sites across greater Boston, Massachusetts.^41,42^ Participants in the patient navigation group were additionally offered access to an LCS patient navigator who provided lung cancer education, facilitated PCP visits for shared decision-making around LCS, assisted in scheduling LCS LDCT appointments, facilitated LDCT appointment attendance through reminder telephone calls and transportation assistance, arranged follow-up studies or office visits when needed, offered tobacco cessation support for participants who were currently smoking, and coordinated care with PCPs, referral specialists, and other care team members.^35,36^ Navigators were trained to conduct their work in a patient-centered manner and to tailor their efforts according to individual patient needs. The number, frequency, and duration of navigation interactions were dictated by the clinical scenario and patient preference, with navigation continuing through completion of LCS and any needed follow-up or until no longer desired by the patient. The navigation intervention was designed with the needs of participants currently experiencing homelessness in mind, and there were no formal or scripted adaptations, enhancements, or modifications to the navigation protocol for participants with housing (ie, who formerly experienced homelessness).

### Measures

#### Homelessness Status

We assessed baseline homelessness status through a series of self-reported items administered at the time of enrollment. All participants had a lifetime history of homelessness, defined as an affirmative response to the following question: “Was there ever a time in your life when you were homeless or did not have fixed, regular housing?” Based on homelessness definitions set forth in the Homeless Emergency Assistance and Rapid Transition to Housing Act of 2009^43^ and used by the US Department of Housing and Urban Development, participants were defined as currently experiencing homelessness if they reported any of the following: (1) usually staying in an emergency shelter, transitional shelter, abandoned building, place of business, car or other vehicle, church or mission, hotel or motel, or anywhere outside during the past 7 days; (2) usually being housed in the last 7 days but unable to stay for at least 14 days; (3) usually being institutionalized in the last 7 days if the admission was less than 90 days ago and they experienced homelessness prior to admission; or (4) usually being institutionalized in the last 7 days if the admission was more than 90 days ago and there was no identified residence where they could stay at least 14 days upon discharge. Institutional settings included jail or prison, an inpatient hospital unit, a medical respite program, a detoxification program, and a residential substance use or behavioral health treatment program. Participants were defined as formerly experiencing homelessness if they reported any living situation that did not meet criteria for current homelessness.

Our primary analyses focused on comparing participants experiencing current vs former homelessness because we believe this distinction is the most important one for policy and practice. Stratification of randomization by this dichotomous homelessness variable ensured unbiased subgroup estimates with respect to navigation treatment effects. Given the large percentage of individuals enrolled in the trial who had formerly experienced homelessness, we conducted sensitivity analyses in which we further distinguished these participants post hoc based on whether they had stable vs unstable housing at the time of enrollment. Participants who formerly experienced homelessness were defined as having unstable housing if they reported any of the following: (1) 2 or more residential moves in the past 12 months,^37,44^ (2) difficulty paying rent or a mortgage in the past 12 months,^37,45^ or (3) homelessness within the past 12 months.^44^ They were defined as having stable housing if they reported any living situation that did not meet the criteria for unstable housing.

#### Other Baseline Measures

##### Sociodemographic Measures

Self-reported sociodemographic measures included age, gender (male, female, or other gender), race and ethnicity, education, health insurance, and cell phone possession. We collected data on race and ethnicity because of the complex interplay among race and ethnicity, homelessness, and health outcomes.^46,47^ Participants identified as Black, Hispanic or Latino, White, or other race or ethnicity (which included American Indian or Alaska Native, other undefined race, or multiple races). We used a 5-item scale adapted from the RAND Course of Homelessness study^48^ to assess past 30-day difficulties meeting subsistence needs for shelter, food, clothing, a place to wash, and a place to go to the bathroom. We categorized summary subsistence difficulty scores as none (0), low (1-5), or high (≥6).^49^

##### General Health Measures

We asked participants to identify the BHCHP site where they usually saw their PCP. We assessed self-rated general health status with a single item.^50,51^ We assessed alcohol use with the Alcohol Use Disorders Identification Test–Concise,^52,53^ drug use with the 10-item Drug Abuse Screening Test,^54,55^ and mental health symptoms with the 6-item Kessler Psychological Distress Scale,^56,57^ and we used established cutoffs to identify disorders in each of these domains. We assessed self-efficacy with the General Self-Efficacy Scale.^58,59^

##### Lung Cancer Risk and LCS-Related Measures

We assessed self-reported years and quantity of cigarette smoking and used this to calculate pack-years of smoking exposure. We asked participants whether they had previously discussed LCS with their PCP. We assessed multiple dimensions of lung cancer risk perception (perceived personal and comparative risk, lung cancer worry and perceived severity, and perceived LCS benefits) using items developed and validated for the National Lung Screening Trial.^60^

#### Navigation Process Measures

Among navigation group participants, we examined navigator checklist logs to assess completion of various navigation tasks. These tasks included establishing contact, providing LCS counseling and education, facilitating LCS shared decision-making visits, scheduling or rescheduling LCS appointments, facilitating follow-up of results and additional testing when needed, addressing insurance issues or transportation needs, and coordinating care with health care personnel.

#### Outcome Measure

The primary outcome was receipt of a 1-time LCS LDCT scan within 6 months (26 weeks) of randomization. Receipt was ascertained via standardized blinded review of participants’ medical records, as described in depth elsewhere.^36^

### Statistical Analysis

We used descriptive statistics to summarize the baseline characteristics of study participants. We compared participants currently vs formerly experiencing homelessness on these baseline characteristics using Fisher exact tests for categorical variables and *t* tests for continuous variables.

To assess whether the patient navigation treatment effect differed between participants currently vs formerly experiencing homelessness, we first examined LCS LDCT completion rates for participants in the navigation and usual care groups, stratified by homelessness status. We calculated the risk difference (RD) between navigation and usual care within each homelessness subgroup, and we compared these RDs by testing the interaction between study group and homelessness status in a linear binomial regression model with the identity link. Among navigation group participants, we used Fisher exact tests to compare individuals currently vs formerly experiencing homelessness on various navigation-related process measures.

In sensitivity analyses, we repeated each of the aforementioned analyses using the 3-level homelessness variable (at the time of enrollment: currently experiencing homelessness, formerly experienced homelessness and have unstable housing, or formerly experienced homelessness and have stable housing). Analyses were conducted using SAS, version 9.4 (SAS Institute). For all primary analyses, *P* < .05 (2-tailed) was considered significant. We report 95% CIs rather than *P* values for the sensitivity analyses involving post hoc assessment of currently experiencing homelessness vs having unstable housing vs having stable housing because of power limitations on 3-way comparisons and to reduce multiple testing.

## Results

**Figure 1.**
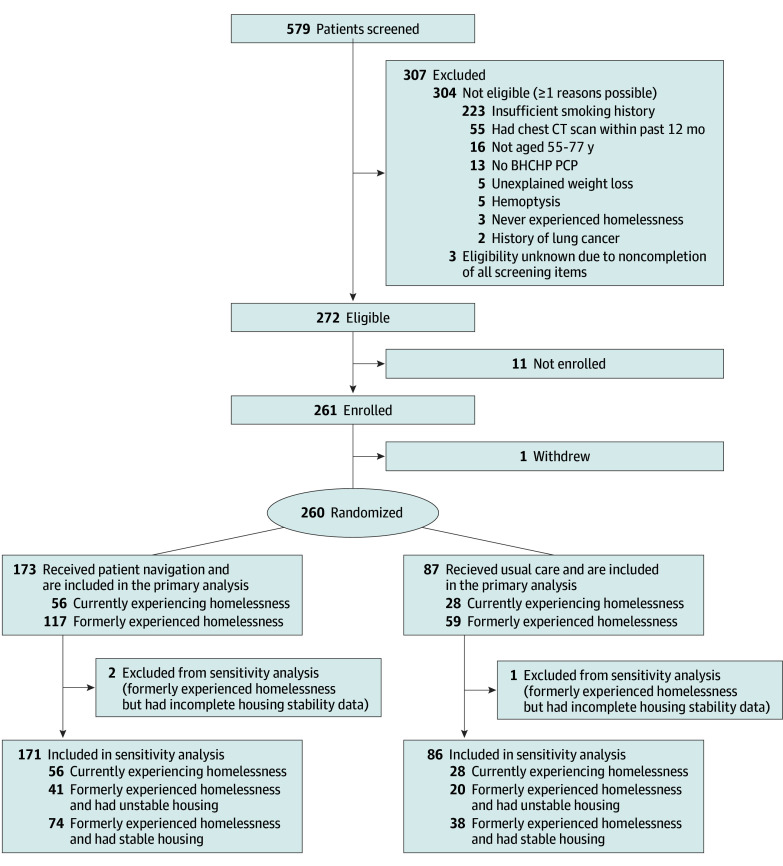
Study Flow Diagram BHCHP indicates Boston Health Care for the Homeless Program; CT, computed tomography; PCP, primary care practitioner.

This subgroup analysis included 260 randomized participants (mean [SD] age, 60.5 [4.7] years). Participants identified as male (184 [70.8%]), female (74 [28.5%]), or other gender (2 [0.8%]). In terms of race and ethnicity, 96 participants (36.9%) were Black, 38 (14.6%) were Hispanic or Latino, 96 (36.9%) were White, and 26 (10.0%) were of other race or ethnicity. At baseline, 84 participants (32.3%) were currently experiencing homelessness, and 176 (67.7%) had formerly experienced homelessness (Table 1). The stratified randomization scheme ensured that these proportions were balanced between study groups (Figure 1). Compared with individuals who formerly experienced homelessness, those currently experiencing homelessness were younger, less likely to have a high school education, less likely to have a cell phone, more likely to have difficulty meeting subsistence needs, more likely to have a mental health disorder, and more likely to currently smoke cigarettes despite having fewer accumulated pack-years of smoking (Table 1). Participants currently experiencing homelessness were also less likely to have previously discussed LCS with their PCP. Notably, these groups did not differ substantially with respect to any of the measured dimensions of lung cancer risk perception.

**Table 1.** Characteristics of Study Participants, Overall and by Baseline Homelessness Status^a^

Characteristic	All participants (N = 260)	Baseline homelessness status		*P* value
		Currently experiencing (n = 84)	Formerly experienced (n = 176)	
**Sociodemographic measures**				
Age, mean (SD), y	60.5 (4.7)	58.9 (4.2)	61.3 (4.8)	<.001
Gender				
Male	184 (70.8)	61 (72.6)	123 (69.9)	.65
Female	74 (28.5)	22 (26.2)	52 (29.5)	
Other gender^b^	2 (0.8)	1 (1.2)	1 (0.6)	
Race and ethnicity^c^				
Black	96 (36.9)	25 (29.8)	71 (40.3)	.13
Hispanic or Latino	38 (14.6)	17 (20.2)	21 (11.9)	
White	96 (36.9)	29 (34.5)	67 (38.1)	
Other race or ethnicity	26 (10.0)	12 (14.3)	14 (8.0)	
High school graduate or GED^d^	122 (47.3)	32 (38.6)	90 (51.4)	.06
Has a cell phone	236 (90.8)	62 (73.8)	174 (98.9)	<.001
Subsistence difficulties^e^				
None	141 (54.2)	20 (23.8)	121 (68.8)	<.001
Low	68 (26.2)	24 (28.6)	44 (25.0)	
High	51 (19.6)	40 (47.6)	11 (6.3)	
**General health measures**				
Health insurance	259 (99.6)	84 (100)	175 (99.4)	>.99
Primary care location				
Program headquarters	184 (70.8)	53 (63.1)	131 (74.4)	.08
Satellite site	76 (29.2)	31 (36.9)	45 (25.6)	
Fair or poor health status	132 (50.8)	45 (53.6)	87 (49.4)	.60
Mental health disorder^f^	75 (29.1)	33 (39.3)	42 (24.1)	.01
Alcohol use disorder^g^	61 (23.9)	25 (30.5)	36 (20.8)	.12
Drug use disorder^h^	72 (27.8)	28 (33.3)	44 (25.1)	.18
General self-efficacy score (10-40), mean (SD)^i^	32.5 (5.6)	31.6 (5.7)	33.0 (5.4)	.08
**Lung cancer risk and LCS-related measures**				
Smoking status				
Current	221 (85.0)	81 (96.4)	140 (79.5)	<.001
Former	39 (15.0)	3 (3.6)	36 (20.5)	
Pack-years of smoking, mean (SD)	48.1 (19.5)	44.0 (19.8)	50.1 (19.0)	.02
Prior discussion of LCS with PCP	99 (38.1)	24 (28.6)	75 (42.6)	.03
Lung cancer risk perceptions (score range), mean (SD)				
Perceived personal risk (2-10)^j^	7.0 (1.9)	7.1 (1.9)	7.0 (2.0)	.92
Perceived comparative risk (3-15)^k^	9.8 (2.7)	9.6 (2.7)	9.9 (2.8)	.43
Worry about lung cancer (2-8)^l^	4.7 (1.9)	4.7 (2.0)	4.7 (1.9)	.92
Perceived lung cancer severity (2-10)^m^	9.3 (1.4)	9.4 (1.2)	9.2 (1.5)	.17
Perceived LCS benefits (3-15)^n^	8.2 (1.6)	8.3 (1.6)	8.1 (1.6)	.41

Abbreviations: GED, general equivalency degree; LCS, lung cancer screening; PCP, primary care practitioner.

^a^ Unless indicated otherwise, values are presented as No. (%) of participants.

^b^ Includes participants who selected nonbinary/genderqueer (n = 1) or other (n = 1).

^c^ Four participants had unknown race or ethnicity due to item nonresponse. Other race or ethnicity includes participants who reported not being of Hispanic or Latino descent and who selected American Indian or Alaskan Native (n = 3), other race (n = 8), or multiple races (n = 15).

^d^ Two participants had unknown educational status.

^e^ Categories based on summed responses to the 5-item RAND Course of Homelessness subsistence difficulty scale (range, 0-15; none [0], low [1-5], or high [≥6]). The *P* value for this comparison is from the Mantel-Haenszel χ^2^ test of trend.

^f^ Defined as a score of 13 or greater on the 6-item Kessler Psychological Distress Scale. Two participants had unknown status due to item nonresponse.

^g^ Defined as a score of 3 or greater for female individuals and 4 or greater for male individuals and individuals of other genders on the Alcohol Use Disorders Identification Test–Concise. Five participants had unknown status due to item nonresponse.

^h^ Defined as a score of 3 or greater on the 10-item Drug Abuse Screening Test. One participant had unknown status due to item nonresponse.

^i^ Higher scores represent greater self-efficacy.

^j^ Assessed with 2 items with 5-point Likert-type response options (n = 258).

^k^ Assessed with 3 items with 5-point Likert-type response options (n = 251).

^l^ Assessed with 2 items with 4-point Likert-type response options (n = 260).

^m^ Assessed with 2 items with 5-point Likert-type response options (n = 258).

^n^ Assessed with 3 items with 5-point Likert-type response options (n = 231).

Among the 176 participants who formerly experienced homelessness, 61 (34.7%) had unstable housing, 112 (63.6%) had stable housing, and 3 (1.7%) could not be further classified due to item nonresponse. Individuals with unstable housing resembled those with stable housing in some respects (eg, cell phone possession, primary care location, alcohol use disorder, smoking status, and smoking pack-years), resembled participants currently experiencing homelessness in other respects (eg, gender, Black race, and drug use disorder), and were intermediate to those groups in other ways (eg, education, subsistence difficulties, and mental health disorder) (eTable 1 in Supplement 2).

### Navigation Treatment Effect by Homelessness Status

**Figure 2.**
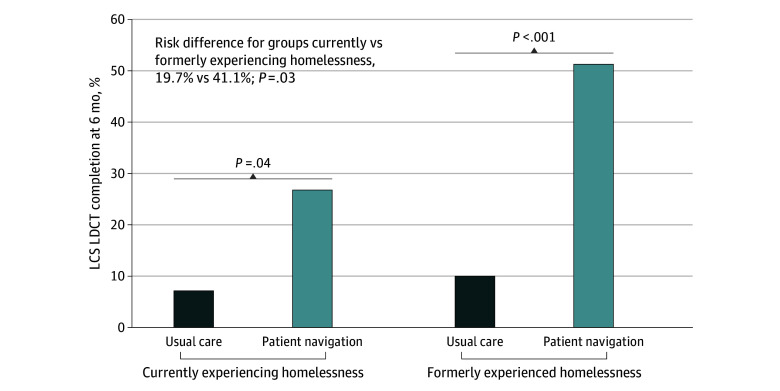
Lung Cancer Screening (LCS) Low-Dose Computed Tomography (LDCT) Scan Attainment Within 6 Months, Comparing Usual Care and Patient Navigation Stratified by Baseline Homelessness Status

Compared with usual care, the navigation intervention significantly increased LCS LDCT completion among both participants currently (15 of 56 [26.8%] vs 2 of 28 [7.1%]; *P* = .04) and formerly (60 of 117 [51.3%] vs 6 of 59 [10.2%]; *P* < .001) experiencing homelessness (Figure 2). However, the treatment effect was smaller among those currently vs formerly experiencing homelessness (RD, 19.7% vs 41.1%; *P* = .03 for comparison of differences). Presented differently (eFigure 1 in Supplement 2), the gap in LCS LDCT completion between participants currently vs formerly experiencing homelessness widened under the patient navigation condition (15 of 56 [26.8%] vs 60 of 117 [51.3%]; RD, 24.5%) compared with the usual care condition (2 of 28 [7.1%] vs 6 of 59 [10.2%]; RD, 3.1%).

In sensitivity analyses, the navigation vs usual care RDs were 48.5% (95% CI, 28.5%-68.5%) for participants with unstable housing and 35.4% (95% CI, 20.5%-50.4%) for those with stable housing (eFigure 2 in Supplement 2).

### Navigation Process Measures by Homelessness Status

Among the 173 patients in the navigation group, the 56 patients currently experiencing homelessness had fewer total contacts (principally, telephone contacts) with the navigator than the 117 patients who formerly experienced homelessness (Table 2). Similar percentages of each subgroup had a shared decision-making visit arranged with their PCP and an LCS appointment subsequently scheduled; however, navigation group participants currently experiencing homelessness were marginally less likely to have been reached with an LCS reminder (25 of 56 [44.6%] vs 70 of 117 [59.8%]; *P* = .07) and were significantly less likely to have had an LCS follow-up appointment arranged (owing to lower LCS completion rates) (19 of 56 [33.9%] vs 65 of 117 [55.6%]; *P* = .01). Other dimensions of navigation engagement were generally lower among those currently experiencing homelessness compared with those formerly experiencing homelessness, but this was not significant.

**Table 2.** Navigation Process Measures Among Navigation Group Participants, Overall and by Baseline Homelessness Status^a^

Navigation activity	All navigation group participants (n = 173)	Baseline homelessness status		*P* value
		Currently experiencing (n = 56)	Formerly experienced (n = 117)	
Established initial contact	163 (94.2)	50 (89.3)	113 (96.6)	.08
No. of contacts per patient, mean (SD)	9.6 (8.5)	7.7 (8.7)	10.5 (8.3)	.049
No. of telephone contacts per patient, mean (SD)	9.5 (8.4)	7.6 (8.7)	10.4 (8.2)	.049
No. of in-person contacts per patient, mean (SD)	0.1 (0.4)	.1 (.5)	0.1 (0.4)	.85
Provided LCS education and counseling	120 (69.4)	36 (64.3)	84 (71.8)	.38
Facilitated LCS shared decision-making visit with PCP	130 (75.1)	41 (73.2)	89 (76.1)	.71
Facilitated LCS referral	136 (78.6)	41 (73.2)	95 (81.2)	.24
Scheduled or arranged LCS appointment	122 (70.5)	38 (67.9)	84 (71.8)	.60
Reminded about LCS appointment	95 (54.9)	25 (44.6)	70 (59.8)	.07
Rescheduled missed LCS appointment	49 (28.3)	16 (28.6)	33 (28.2)	>.99
Facilitated follow-up of LCS results	84 (48.6)	19 (33.9)	65 (55.6)	.01
Facilitated additional studies or testing	14 (8.1)	3 (5.4)	11 (9.4)	.55
Provided smoking cessation support	58 (33.5)	17 (3.4)	41 (35.0)	.61
Addressed insurance issues	50 (28.9)	9 (16.1)	41 (35.0)	.01
Facilitated transportation	27 (15.6)	6 (1.7)	21 (17.9)	.27
Coordinated care with PCP	159 (91.9)	51 (91.1)	108 (92.3)	.77
No. of navigator contacts with PCP per patient, mean (SD)	5.0 (4.1)	4.8 (4.5)	5.1 (3.9)	.65
Provided non-LCS care coordination	63 (36.4)	19 (33.9)	44 (37.6)	.74

Abbreviations: LCS, lung cancer screening; PCP, primary care practitioner.

^a^ Unless indicated otherwise, values are presented as No. (%) of participants.

In sensitivity analyses, navigation group participants with unstable housing (n = 41) and stable housing (n = 74) were generally similar to each other—and distinct from navigation group participants currently experiencing homelessness—on most navigation process metrics (eTable 2 in Supplement 2).

## Discussion

In this pragmatic trial of LCS patient navigation at a large HCH program in Boston, the patient navigation intervention produced sizable and significant effects on screening participation among both patients currently and formerly experiencing homelessness, resulting in LCS screening rates for both subgroups that were higher than LCS estimates in the US general population during a similar time frame.^61^ However, the patient navigation effect size among individuals currently experiencing homelessness was about half of that seen in those who formerly experienced homelessness, leading to an unintended widening of the screening disparity between these groups. Navigation process measure data suggest that impediments to communication (eg, initial contact, number of contacts, reminder contacts) may have underpinned this differential response to the intervention, although more subtle differences appeared across a range of navigation activities. Taken together, these findings offer another example of how the digital divide can contribute to differential benefits from a new health care intervention or innovation.^62^

Individuals currently experiencing homelessness were less likely at baseline to have been counseled previously about LCS by their PCP, which may reflect reasonable consideration of competing health and social priorities among these individuals. Interestingly, despite having a higher burden of difficulty meeting basic survival needs, participants currently experiencing homelessness did not differ from those who formerly experienced homelessness in their lung cancer risk perceptions, including level of worry about lung cancer and perceived benefits of screening. Conventional wisdom has long held that the daily struggles of homelessness render considerations around cancer screening irrelevant or even misguided. Yet our findings demonstrate that even in the face of competing subsistence priorities, individuals currently experiencing homelessness still harbor high levels of concern about their longer-term health outcomes and belief in the value of recommended preventive measures. The difficulty may lie more in their available bandwidth and resources to execute on these concerns.^63^

Our findings have 2 major implications. First, even though the patient navigation intervention was designed specifically with the needs of patients experiencing homelessness in mind,^35^ further adaptations or implementation strategies may be required in order to optimize outcomes for individuals currently experiencing homelessness. For instance, coupling cancer screening navigation with social needs assessment and intervention may help individuals experiencing homelessness cope with more immediate survival priorities while also working toward their longer-term health goals. The difficulty with telephone communication, combined with patients currently experiencing homelessness being less likely to have cell phones at baseline, suggests a need for creative ways to overcome communication and care coordination barriers in this population. Based on our prior experience, simply providing such patients with free cell phones and plans does very little to overcome these barriers, because mobile devices are frequently lost or stolen in the context of homelessness.^64^ Instead, effective solutions may require using more proactive in-person outreach methods and capitalizing on in-person clinical encounters to coordinate multiple dimensions of care. Given the limited prior studies on cancer screening navigation in homeless health care settings,^65,66^ these and other adaptations or modifications should be the subject of future research.

The second implication is the need to address homelessness itself as a potent social determinant of health. A large body of evidence has documented substantial disparities in a range of health-related conditions and outcomes among people experiencing homelessness.^67,68,69^ The literature on the health benefits of providing housing to people experiencing homelessness is still evolving.^70^ Notably, in this study, our sensitivity analyses found that people living in unstable housing had comparable outcomes to those living in stable housing, suggesting that even a precarious housing arrangement is better than absolute homelessness.

### Limitations

This study has some limitations. Limitations in the INHALE parent trial have been described elsewhere.^36^ Additional limitations specific to this analysis include the fact that homelessness status was measured only at baseline, so we did not capture potential changes in participants’ housing arrangements that may have occurred during the 6-month follow-up period. We would expect any resulting misclassification to bias analyses toward the null hypothesis of no difference between groups. Because the experience of homelessness is influenced by local contextual factors, our findings may not be generalizable to other geographic settings. Stratification of randomization by homelessness status enabled unbiased subgroup effect estimates for participants currently vs formerly experiencing homelessness; however, post hoc examinations of individuals with unstable vs stable housing were not necessarily free from confounding bias. Comparisons of navigation process measures for participants currently vs formerly experiencing homelessness were likely underpowered to detect subtle differences in such measures. Additionally, differences in these process measures should be regarded as hypothesis-generating rather than causal in nature; for instance, it is not possible to determine whether disparities in the number of navigator telephone contacts between subgroups caused differing screening completion between subgroups, or whether both of these observations were jointly influenced by an underlying unmeasured confounder.

## Conclusions

In this subgroup analysis of a randomized clinical trial, patient navigation improved LCS participation among HCH patients both currently and formerly experiencing homelessness, but the effect size was smaller for those experiencing current homelessness. Optimizing outcomes in this patient population will likely require further refinement or adaptation of the patient navigation intervention, coupled with continued policy efforts to reduce homelessness through the provision of housing and supportive services.

## Notes

Supplement 1.
Trial Protocol

Supplement 2. **eFigure 1.** LCS LDCT Scan Attainment Within 6 Months, Comparing Participants Currently and Formerly Experiencing Homelessness Stratified by Study Group **eFigure 2.** LCS LDCT Scan Attainment Within 6 Months Among Participants With Stable or Unstable Housing Who Formerly Experienced Homelessness **eTable 1.** Characteristics of Study Participants by Baseline Homelessness Status, With Individuals Who Formerly Experienced Homelessness Subdivided as Having Unstable or Stable Housing **eTable 2.** Navigation Activities Among Navigation Group Participants by Baseline Homelessness Status, With Individuals Who Formerly Experienced Homelessness Subdivided as Having Unstable or Stable Housing

Supplement 3.
Data Sharing Statement
