# Supplementary material for: Homelessness, Patient Navigation, and Lung Cancer Screening in a Health Center Setting: A Subgroup Analysis of a Randomized Clinical Trial
Source: JAMA Netw Open. 2025 Jul 17;8(7):e2519780. doi: 10.1001/jamanetworkopen.2025.19780 (PMC12272284; doi:10.1001/jamanetworkopen.2025.19780)
Supplement: Supplement 1. — Trial Protocol [file jamanetwopen-e2519780-s001.pdf]

INHALE Study Protocol

Version summary

This document contains the original IRB-approved INHALE trial protocol in addition to all revised versions, presented in chronological order. A brief summary of the changes made in each version follows:

1. Version 11/12/2020: This is the original protocol that was in place when the study was launched on 11/20/2020.

2. Version 12/16/2020: This version contains minor revisions to provide additional details on the recruitment, consent, and remuneration procedures for participants enrolled via phone vs. in-person.

3. Version 06/17/2021: This version clarifies that, for the primary outcome of lung cancer screening (LCS) low-dose computed tomography (LDCT) completion at 6 months, the timeframe of "6 months" is defined precisely as 26 weeks. In addition, the "one month" timeframe on timely follow-up of abnormal LDCT results is clarified as 4 weeks.

4. Version 07/23/2021: This version contains minor revisions to clarify some of the language in the consent process.

5. Version 09/30/2021: This version allows for expanded in-person recruitment efforts to include Boston Health Care for the Homeless Program (BHCHP) sites other than the 6 core BHCHP sites identified in the original protocol.

TITLE: Lung cancer screening navigation for homeless people: A pragmatic trial

PRINCIPAL INVESTIGATOR: Travis P. Baggett, MD, MPH

GRANT NUMBER: 131162-RSG-17-157-01-CPPB (American Cancer Society)

VERSION DATE: November 12, 2020

I. BACKGROUND AND SIGNIFICANCE

A. Historical background

An estimated 2.3-3.5 million people experience homelessness annually in the US.[1] Lung cancer incidence and mortality are substantially higher in this vulnerable population than in comparably-aged non-homeless individuals.[2-4] To address this disparity, we propose a pragmatic clinical trial to test the effect of patient navigation on lung cancer screening (LCS) completion among homeless-experienced adults at increased risk for lung cancer. Work that we and others have done suggests 2 reasons why such an intervention is needed.

1) Lung cancer is a major cause of death among homeless adults and the leading cause of tobacco-attributable death in this population.

In our study of 28,033 adults who used Boston Health Care for the Homeless Program (BHCHP) services in 2003-08, cancer was the second-leading cause of death and the leading killer of adults ≥45 years old.[5] Lung cancer accounted for one-third of these cancer deaths.[5] In a subsequent study of cancer epidemiology in this cohort, lung cancer was the leading type of incident cancer and the leading cause of cancer death among both men and women, with age-standardized incidence and mortality rates exceeding those in the Massachusetts adult population more than 2-fold.[2] An estimated 88% of incident lung cancer cases[2] and 93% of lung cancer deaths[6] were attributable to tobacco smoking, making lung cancer the leading type of smoking-attributable cancer and the leading cause of smoking-attributable death among homeless people. The excess burden of lung cancer in these studies of homeless adults in Boston was similar to that observed in studies of homeless and marginally housed people in Canada[4] and Glasgow[3] (Table 1), suggesting that these disparities are not geographically constrained. This body of evidence highlights the need for interventions to reduce lung cancer disparities in homeless populations. One avenue for doing so is LCS with low-dose computed tomography (LDCT) to promote detection and potentially curative treatment of early-stage lung cancer. This strategy was associated with a 20% reduction in lung cancer mortality in the National Lung Screening Trial (NLST).[7] However, implementing such a strategy presents formidable hurdles in the setting of homelessness.

Table 1. Lung cancer among homeless adults

Study location (yrs)	SIR/SMR (95% CI)
Boston (2003-2008)	
Incidence, men	2.30 (1.84, 2.84)
Incidence, women	2.23 (1.41, 3.35)
Mortality, men	2.39 (1.83, 3.08)
Mortality, women	2.31 (1.26, 3.88)
Canada (1991-2001)	
Mortality, men	1.91 (1.67, 2.18)
Mortality, women	1.73 (1.26, 2.36)
Glasgow (1975-1993)	
Incidence, men	1.64 (1.41, 1.86)

2) Homeless people have suboptimal rates of cancer screening and are diagnosed with screen-detectable malignancies at later stages than non-homeless people; barriers to implementing LCS in the setting of homelessness could exacerbate existing disparities in lung cancer outcomes.

In the 2003-08 BHCHP study of cancer epidemiology described above, homeless individuals were diagnosed with colorectal cancer and female breast cancer at significantly later stages than adults in the Massachusetts general population.[2] Several studies have documented low rates of screening for these malignancies among homeless individuals.[8-12] Given the high prevalence of smoking among homeless people[8,13-18] and the aging of the homeless population,[19] many homeless individuals are expected to meet the recommended criteria for consideration of LCS with LDCT[20,21]; however, as with colorectal and breast cancer screening, there are several obstacles to successfully implementing LCS in homeless populations. Homeless individuals struggling to meet basic subsistence needs may place a low priority on screening for asymptomatic illness[22] or may feel too emotionally overwhelmed to cope with the possibility of an abnormal result. Additionally, despite a high burden of cancer risk factors, most homeless people appear to believe that their susceptibility to cancer is equal to or less than that of others the same age.[8] Lack of insurance may pose another barrier,[23,24] although this is less of a concern in Massachusetts and could improve elsewhere in states that expanded Medicaid under the Affordable Care Act. Even among insured individuals who are interested in obtaining LDCT screening, the process of scheduling and attending the test may prove logistically challenging because of difficulty navigating complex facilities and care systems,[25] especially for the large proportions of homeless individuals with psychiatric and cognitive impairments.[26-32] Furthermore, ensuring timely follow-up of abnormal results may be challenging because of communication barriers and competing psychosocial issues. These barriers introduce the possibility that existing disparities in lung cancer mortality could worsen if homeless people are less able to complete LCS than other segments of the population.

B. Preliminary data

Our prior epidemiologic work demonstrating the burden of lung cancer disparities among homeless people is described above and provides the motivation for the proposed study. In addition, this study is informed by administrative data from BHCHP, survey and clinical trial studies with homeless smokers conducted by the PI (Dr. Baggett), navigation studies conducted by co-investigator Dr. Percac-Lima, and LCS-oriented studies conducted by co-investigators Dr. Park and Dr. Rigotti. Collectively, this preliminary data has demonstrated the following:

1) We have the patient volume to support this study. Based on internal data from the BHCHP Institute, 3,443 unique individuals aged 55-77 years old were seen at any BHCHP site from 9/1/2015 to 9/1/2016. Of these, 53% were current smokers and 23% were former smokers, totaling 2,616 potentially eligible individuals seen by the program in a 12-month period. When extrapolated over the planned 2.5-year enrollment period, we anticipate being able to recruit and randomize the proposed number of 300 participants.

2) We have characterized the target population. In April-July 2014, we used time-location sampling to conduct a survey of 306 homeless adult smokers at 5 high-volume BHCHP clinical sites.[33-35] The response rate was 86%. One-quarter of respondents (N=78) were 55-77 years old. Of these, the vast majority were non-Hispanic white or black men with a high school degree. Seventy percent had ever experienced a traumatic head injury, 64% had used any drug in the past month, 39% had consumed alcohol to intoxication in the past month, 60% reported feeling seriously depressed, and half screened positive for PTSD. One-third reported COPD and 17% reported cardiovascular disease. Past-month difficulty finding shelter, food, and clothing were common. The mean number of cigarettes per day was 12 and the mean Fagerstrom nicotine dependence score was 3.8 (range 0-12). These findings highlight the medical complexity and competing life issues of older homeless smokers and underscore the need for a navigation-based intervention to reduce the barriers to LCS.

3) We can recruit and longitudinally retain homeless people in a clinical trial. From October 2015 to June 2016, we conducted a 3-arm (N=25 per arm) pilot randomized controlled trial testing the effect of a) contingent financial rewards for smoking abstinence, and b) text messaging to support smoking abstinence, against c) a shared control condition, among homeless smokers at BHCHP (NCT02565381). Sixty-eight percent of eligible individuals enrolled, and we reached our target enrollment within 6 months. Study attendance and retention rates were excellent. Of 14 possible assessment visits, participants attended a median of 10, with 97% attending ≥1 visit and 77% attending ≥7 visits. These findings demonstrate that our team has the expertise to conduct a behavioral treatment trial in the context of homelessness and to engage and retain this vulnerable population in a longitudinal fashion. This is facilitated by our extensive knowledge of the target population, our person-centered approach to participant engagement, our strong partnership with BHCHP, and the central location of BHCHP headquarters with respect to other homeless services.

4) Our navigation intervention is based on a model that has proven effective at improving lung and other cancer screening among underserved patient populations in Boston. Key collaborator, Dr. Sanja Percac-Lima, has developed and tested navigation interventions that have been associated with improved cancer screening rates among diverse, low-income community health center patients in Boston.[36,37] She conducted a randomized controlled trial (RCT) across 18 clinical practices in an academic primary care network evaluating the impact of patient navigation relative to usual care for high-risk individuals who were overdue for breast, colorectal, and/or cervical cancer screening.[38] Patients assigned to navigation were significantly more likely to complete screening for breast (23.4% vs. 16.6%, p=0.009), cervical (14.4% vs. 8.6%, p=0.007), and colorectal (13.7% vs. 7.0%, p<0.001) cancer during the follow-up period.[38] More recently, with grant support from ACS and in collaboration with Drs. Park and Rigotti, Dr. Percac-Lima was the principal investigator of an RCT testing the effect of patient navigation on chest CT completion among low-income patients eligible for LCS at 5 MGH-affiliated health centers in greater Boston. Preliminary analysis of data after 11 months of follow-up has shown that participants in the navigation arm were significantly more likely to obtain a screening lung CT than participants assigned to usual care (24% vs. 9%, p<0.01). The LCS navigation approach used in our study will be based on these successful models.

C. Study rationale

Mitigating disparities in lung cancer outcomes and fully realizing the potential benefits of LCS among homeless people will require thoughtful interventions deployed through the clinical programs that serve this vulnerable population. Patient navigation, a strategy for guiding individuals through complex health systems,[39] may be a promising approach for helping homeless people overcome their unique barriers to LCS.[40] Navigation interventions have been shown to improve cancer screening participation[36,37,41-56] and diagnostic resolution of abnormal results[57-63] in vulnerable populations. Patient navigation has been endorsed by the ACS[64] and the National Cancer Institute[65] as a valuable approach to reducing cancer health disparities,[65] but this approach has not been tested rigorously in a homeless health care setting. Our research group is well-poised to conduct such a study by leveraging our extensive clinical and research experience with this population and our 9-year partnership with BHCHP, an internationally-renowned organization that serves over 12,000 currently (84%) and formerly (16%) homeless people annually through dozens of clinical sites in greater Boston.[66] This innovative community partnership has given rise to numerous impactful studies on the health of homeless-experienced people,[2,5,6,33-35,67] including a recently pilot trial for homeless smokers that had brisk recruitment, high retention rates, and encouraging results (NCT02565381).

II. SPECIFIC AIMS

Aim 1: To determine the effect of patient navigation, added to usual care, on 1°) LCS LDCT completion at 6 months and 2°) LCS LDCT completion at 6 months and diagnostic follow-up of abnormal results within 1 month of the recommended timeframe, among homeless-experienced people who are eligible for LCS.

Hypothesis: Participants assigned to receive patient navigation will have significantly greater 1°) LCS LDCT completion at 6 months and 2°) LCS LDCT completion at 6 months with timely diagnostic follow-up of abnormal results.

Aim 2. To conduct post-intervention qualitative interviews of trial participants and BHCHP PCPs to explain and interpret the quantitative findings of the pragmatic clinical trial.

Based on a sequential explanatory mixed methods approach, Aim 2 interviews will examine how and why participants decided whether to undergo LCS, the barriers they encountered in doing so, the ways in which the navigator helped them overcome these barriers, and opportunities for improving the intervention for future use.

III. SUBJECT SELECTION

A. Inclusion and exclusion criteria

To be eligible to participate, individuals must meet **all** the following criteria:

1) Aged 55-77 years old, assessed by self-report and verified by date of birth.
   Rationale: LCS LCDT is covered only for patients aged 55-77.
2) Have a 30 pack-year smoking history *and* have smoked within the past 15 years.
   Rationale: We will be recruiting exclusively smokers because they are at the highest risk for lung cancer and therefore stand to receive the most benefit from a LCS. These smoking history parameters are consistent with current practice guidelines and Medicare eligibility criteria.
3) Have a BHCHP primary care provider (PCP).
   Rationale: Patient navigation is designed to support and extend services provided by medical practitioners. Having a PCP will help to ensure longitudinal medical oversight of the LCS process and appropriate follow-up of abnormal results.
4) Are currently or formerly homeless, assessed by self-report and defined as ever having experienced a time in their life where they did not have fixed, regular housing.
   Rationale: Both currently and formerly homeless individuals were included in our epidemiologic analysis showing a more than 2-fold higher incidence and mortality rate from lung cancer compared to the general Massachusetts adult population.[2] BHCHP and most HCH programs nationally continue to serve patients after they have gained housing.
5) Be proficient in English, assessed with items asking about native language and self-reported comfort communicating in English among non-native speakers.
   Rationale: Because of a budget reduction by the funding agency, we do not have the resources to develop study materials or conduct in-person navigation in languages other than English. Additionally, the vast majority of individuals in the target population speak English.

Individuals meeting **any** of the following criteria will be excluded from the study:

1) A recent prior chest computed tomography (CT) imaging (in the past 12 months).
   Rationale: This study is aimed at individuals who are most likely to benefit from LCS, based on evidence from the National Lung Screening Trial. Potential participants that have recently received a chest CT will have recently been scanned for lung cancer, therefore an additional screening will not benefit their health outcomes.
2) Any personal history of lung cancer, or current presentation with symptoms concerning for lung cancer (e.g. hemoptysis or unexplained weight loss of >15 lbs. in the past year).
   Rationale: Individuals with a history of lung cancer or symptoms concerning for lung cancer require surveillance or diagnostic lung imaging, respectively, and are not appropriate candidates for screening lung imaging, which by definition is designed for individuals without symptoms or history of the illness being screened for.
3) Inability to provide informed consent, assessed with knowledge questions about the material presented during the informed consent process that individuals must correctly answer before providing informed consent to participate.
   Rationale: In this vulnerable population, we wish to take a conservative approach to the informed consent process to help ensure that participants fully understand the pros and cons of participating in the research study.
4) PCP is the study PI (Dr. Travis Baggett).
   Rationale: BHCHP patients under the primary care of Dr. Baggett may feel unduly influenced to participate. In addition, since the primary study outcome (receipt of LDCT for LCS) requires PCP involvement, the inclusion of Dr. Baggett's patients creates a conflict of interest and the potential for bias.

B. Source of subjects and recruitment methods

This study will be registered with ClinicalTrials.gov prior to the recruitment and enrollment of human subjects. All participants will be recruited from BHCHP clinical sites. BHCHP does not have its own IRB but instead will rely on the Partners Human Research Committee for IRB review through a reliance agreement initiated through SMART IRB. In addition to being a faculty physician-investigator at Massachusetts General Hospital and Harvard Medical School, the study PI is the Director of Research at BHCHP and is very familiar with the clinical environment and patient population served by the program.

Individuals must be homeless to enroll in services at BHCHP; however, some patients continue receiving care at the program after they are no longer homeless. An internal analysis of individuals seen at BHCHP in 2011 found that 16% of patients were housed, most without supportive services and therefore at potentially high risk for recurrent homelessness. Because our epidemiologic analyses demonstrating the dramatic lung cancer disparities experienced by BHCHP patients included both currently and formerly homeless individuals, and because of the often cyclic nature of homelessness, we will include both currently and formerly homeless individuals in the proposed study. We refer to this group collectively as "homeless-experienced." Owing to the Massachusetts system of universal health insurance, about 80% of BHCHP patients are insured by the state Medicaid program,[68] which covers LCS LDCT among eligible beneficiaries,[69] and a sizable proportion of those remaining are covered by Medicare alone. This will help ensure the availability of LCS LDCT for the vast majority of potential participants.

Participants will be recruited through a combination of 3 methods:

1) In-person screening of patients at BHCHP clinical sites: Study personnel will screen patients in-person at 6 high-volume BHCHP clinic sites: a) Pine Street Inn, a 470-bed shelter where BHCHP operates a medical clinic 7 days/week in the men's shelter and 6 days/week in the women's shelter; b) Southampton Shelter, a 400-bed men's shelter where BHCHP operates a medical clinic 7 days/week; c) Woods Mullen Shelter, a 200-bed women's shelter where BHCHP operates a medical clinic 5 days/week; d) New England Center and Home for Veterans, a 209-bed emergency and transitional shelter where BHCHP operates a medical clinic 5 days/week; e) St. Francis House, a daytime multiservice facility for homeless people where BHCHP operates a medical clinic 5 days/week; and f) Jean Yawkey Place, the BHCHP headquarters facility where medical, behavioral health, and dental clinics operate 5 days/week with an on-site pharmacy and medical respite unit. Our prior survey fieldwork with this population has given us extensive experience with tactfully deploying this proactive, in-person recruitment technique.[33-35] In light of the COVID-19 pandemic, BHCHP clinical sites and shelters are experiencing a significant decrease in patient volume and a lack of additional space to safely conduct in-person recruitment and data collection activities. Under these circumstances, study personnel will initially focus in-person screening and data collection efforts at Jean Yawkey Place, which has the highest volume of patients and the greatest degree of flexibility in physical space and layout. This will allow the study personnel to have an in-person presence at the clinic while practicing physical distancing and prioritizing the health and well-being of participants and study staff. If COVID-19 restrictions loosen and patient volume increases at shelter sites, we will expand our efforts to include the five additional sites, depending on COVID-19 public health guidelines and facility protocols.

2) Review of BHCHP electronic records: BHCHP has a comprehensive EHR system (Epic) that prompts providers to assess tobacco use at every clinical encounter, facilitating determination of smoking history and current smoking status. Individuals with a BHCHP PCP who have been seen in a BHCHP clinic in the past year and who were current or former smokers at the time of their most recent clinical encounter will be approached in person at BHCHP clinical sites if they have a scheduled appointment or contacted proactively via phone (if they have listed contact information) by a research coordinator and screened for eligibility to participate. The BHCHP Chief Medical Officer has approved this recruitment strategy on behalf of all primary care providers at BHCHP clinical sites, who will in turn be notified about our recruitment approach prior to the start of the study (letter of approval in Appendix J).

3) Referrals from BHCHP providers: We will advertise the study to all BHCHP providers and in all BHCHP clinical sites. Individuals deemed by a BHCHP provider to be potential candidates for LCS can be referred to the research coordinator, who will screen referred patients for eligibility to participate.

IV. SUBJECT ENROLLMENT

An integrated study schema depicting recruitment, enrollment, treatment assignment, and outcomes assessment has been uploaded as an attachment to this application.

A. COVID-19 symptom and exposure screening

BHCHP staff currently screen all patients for COVID-19 symptoms or a recent positive COVID-19 test upon entry to BHCHP clinics and facilities. BHCHP is also providing patients with facemasks before entering the building. Study personnel will re-screen participants using the same approach before conducting any research activities in an enclosed room. If BHCHP ceases their COVID-19 screening procedure, study personnel will use BHCHP's "COVID-19

Screening Tool for People Experiencing Homelessness" to screen all participants for current COVID-19 symptoms and past 14-day COVID-19 exposures (i.e., travel to high risk countries or states; unmasked exposure to a person with COVID-19). Study staff will wear appropriate personal protective equipment (PPE) per MGH guidelines and policies and remain 6 feet away from the patient. If patient endorses any COVID-19 symptoms or high-risk exposures, study personnel will immediately notify a clinic staff member of the presence of a symptomatic patient and will not engage in any study activities. Immediately after departure of the symptomatic patient, study personnel will disinfect surfaces that were within 6 feet of the symptomatic patient. If the participant reports not having symptoms, they can be directed to the study table/interview room. If the study coordinator makes initial contact with a participant over the phone, before scheduling an in-person visit to obtain the ROI consent, study personnel will screen the participant within 72 hours before the appointment. If participants report COVID-19 symptoms or exposures, study staff will request participants' permission to contact their BHCHP PCP via Epic messaging who can then advise the participant on next steps (e.g., testing and/or quarantine). If participants do not provide permission to study staff to contact their PCP, study staff will recommend that the participant seek care for their symptoms and/or exposure and offer to do the initial study procedures over the phone. If they are not interested in completing any further study procedures over the phone, study staff will call the participant again in 10 days to check-in and rescreen before attempting to schedule any study procedures in person. Participants will be advised to put on a facemask, regardless of symptoms, before coming in to meet with the research coordinator. Research personnel will instruct participants to notify them before arriving if they have fever or symptoms of COVID-19.

B. Methods of enrollment

Participants recruited through the above methods (section III.B) will be screened for eligibility either in-person or by phone. Prior to contacting participants, study staff will use the eligibility screener (Appendix A) to record information related to eligibility that was able to be collected during the prior review of prospective participants' BHCHP EHR (as referenced above).

Upon approaching the participants, study staff will use participant self-report to verify any information collected via their EHR and will collect the minimum amount of additional information necessary to assess and verify study eligibility. Eligible individuals identified through in-person screening will be offered the chance to enroll immediately. Those identified through phone screening will be asked to attend an in-person enrollment visit at one of clinic sites listed above. Participants will be asked to provide verbal informed consent before enrolling.

At least six months after initial enrollment, participants who were randomized to the patient navigation arm of the study will be contacted via phone or approached in person at their next BHCHP appointment to be offered the opportunity to participate in a 45-minute qualitative interview. Forty participants will be interviewed in total, with purposive sampling of participants who did (N=20) and participants who did not (N=20) attain the primary outcome of completing a lung screening CT scan within six months.

At approximately 6 months after the last patient participant is enrolled, providers who had at least one patient enrolled in the trial will be contacted via phone, email, or in person to be offered the opportunity to participate in a 45-minute qualitative interview. Ten participants will be interviewed in total, with purposive sampling to ensure that at least one provider who works at each of the targeted BHCHP clinic sites are interviewed.

C. Informed consent procedures

We will obtain verbal informed consent for this study. Eligible participants will be read a verbal consent script (Appendix B) and be presented with a study fact sheet (Appendix C) that will reinforce the consent script information. Depending on space availability, participant and research coordinator preferences, and whether in-person research activities are safe and permissible, informed consent will be obtained in one of the following ways: 1) “telephone booth” approach where the research coordinator would conduct the consent via Microsoft Teams or Enterprise Zoom on an encrypted laptop with the participant in a dedicated space on an encrypted study tablet; 2) meet in the clinic lobby, in a screened off area while maintaining at least 6 feet distance; 3) meet in an enclosed BHCHP clinic room while maintaining at least 6 feet distance if safe to do so; or 4) over the phone (only available if in-person non-essential research activities are completely restricted). The research coordinator will confirm participants' understanding of the consent materials with basic knowledge questions about the information presented to them to ensure that they have the capacity to provide informed consent. Individuals who are able to correctly answer these questions will be asked to give their verbal consent to participant, which will be recorded by the research coordinator. Participants who provide informed consent over the phone will be given the option to receive a copy of the study fact sheet via picking up in-person at the JYP clinic, via mail if the participant has a mailing address, via secure email, or via email without send secure after obtaining participant's permission.

40 participants who agree to participate in the post-intervention qualitative interviews will participate in a second verbal informed consent process at least 6 months after their initial enrollment. The research coordinator will read a separate verbal consent script (Appendix G) that reinforces the information presented in a fact sheet (Appendix F) that will be distributed to participants. The fact sheet will focus on the purpose, requirements, risks, and benefits of participating in the qualitative interview. The research coordinator will confirm participants' understanding of the consent materials with basic knowledge questions about the information presented to them to ensure that they have the capacity to provide informed consent. Individuals who are able to correctly answer these questions will be asked to give their verbal consent to participant, which will be recorded by the research coordinator.

Additionally, we will obtain verbal consent to participate in qualitative interviews from 10 BHCHP PCPs who had at least 1 patient enrolled in the trial. Similar to the participants who participate in the interviews, the providers will be read a verbal consent script (Appendix I) that coincides with a fact sheet (Appendix H) that will be distributed to them. The research coordinator will record the providers' verbal consent to participate in the interview.

We believe the verbal consent is justified for several reasons:

1) The experimental intervention, patient navigation, is a low-risk and widely-recommended behavioral intervention strategy with a sound evidence base in promoting cancer screening in vulnerable populations. Indeed, patient navigation has been endorsed by the ACS[64] and the National Cancer Institute[65] as a valuable approach to reducing cancer health disparities,[65] and is now part of routine care in some practice settings, including at MGH-affiliated health centers.

2) The outcome that the navigator is facilitating – obtaining LDCT for LCS among individuals at increased risk for lung cancer – is concordant with the guideline recommendations of the USPSTF,[20] ACS,[70] and multiple other professional groups.[71-74]

3) The study procedures present no more than minimal risk, as outlined below in section VII.

In order to ascertain the primary and secondary study outcomes of LDCT completion and LDCT completion with timely diagnostic follow-up (see section VII.B.), we will obtain written authorization from all enrolled participants to obtain their health/medical records for the 14 months following their trial enrollment date from the following Boston-area hospitals: Massachusetts General Hospital, Brigham & Women's Hospital, Boston Medical Center, Cambridge Health Alliance hospitals, and Tufts / New England Medical Center, in addition to any other hospital that the participant identifies as being one they typically use. Written authorization will be obtained in person after participants complete verbal informed consent using HIPAA-compliant Release of Information form which has been vetted by privacy officers at BHCHP, Boston Medical Center, and MGH (Appendix N).

If COVID-19 prompts further restrictions around in-person research activities at BHCHP, the research coordinator will transition to completing the verbal consent process over the phone or via the HIPAA compliant video conferencing platforms, Microsoft Teams or Enterprise Zoom depending on each participant's preference and access to technology. Based on feedback from the Boston Medical Center Compliance and Privacy Office, obtaining participant medical records for research purposes will continue to require written authorization regardless of current restrictions. Therefore, we will need to wait until in-person research restrictions are lifted in order to obtain a signed ROI form from each enrolled participant in person. In this circumstance, a research coordinator will either directly contact all research participants by phone or attempt to meet them in person at a clinic site to complete the form. A research coordinator will also offer participants with a stable home address the option to receive a pre-stamped envelope with the ROI form to have them sign and return back by mail to our MGH office.

D. Treatment assignment

After providing informed consent and completing a baseline survey (see section V. A.), participants will be block-randomized in a 2:1 ratio to usual care with (N=200) or without (N=100) LCS navigation. The randomization scheme will be concealed from the study coordinator responsible for randomizing participants. Randomization will be stratified by smoking status (current/former), housing status (homeless/housed), prior discussion of lung cancer screening with their PCP, and primary clinical site where the patient sees their PCP (JYP vs. non-JYP sites) to ensure balance between study arms on these variables. We will stratify by smoking status (determined based on responses to the eligibility screener) because lung cancer risk perceptions and LCS motivations may differ between current and former smokers.[75,76] Current smoking will be defined as having smoked all or part of a cigarette in the past 30 days.[77] We will stratify by housing status based on responses to various items within the baseline survey because the navigation needs of formerly homeless people in housing may differ from those who are currently homeless. Current homelessness will be defined in the following ways: a) usually staying in an emergency shelter, transitional shelter, abandoned building, place of business, car or other vehicle, church or mission, hotel or motel, or anywhere outside during the past 7 days, b) usually housed in last 7 days but cannot stay in a residence for at least 14 days, c) usually institutionalized in the last 7 days if the admission was less than 90 days and they were homeless prior to admission, or d) usually institutionalized in the last 7 days if the admission was more than 90 days and they have no identified residence where they can stay at least 14 days upon discharge. These definitions are concordant with definitions of homelessness used by the US Department of Health and Human Services (section 330 of the Public Health Service Act, 42 USC § 254b)[78] and by the US Department of Housing and Urban

Development (S. 896 HEARTH Act of 2009).[79] We will stratify based on prior discussion of lung cancer screening with their PCP (defined by an item within the baseline survey) due to the fact that these participants are farther along in the lung cancer screening process. Finally, we will stratify by the patient's primary care clinical site since clinics may differ in their proximity to facilities that offer LCS or in their practice patterns around recommending and ordering LDCT for LCS. We will create 2 clinical site strata: 1 for JYP and one encompassing all of the 5 non-JYP study sites described above.

The 2 study arms are as follows:

Arm 1: Usual care ***without*** patient navigation (N=100). Participants assigned to this arm will be given basic educational materials on general lung health (Appendix K) and referred back to their PCP to discuss whether to pursue lung cancer screening per usual practice.

Arm 2: Usual care ***with*** patient navigation (N=200). Participants assigned to this arm will be informed about LCS, provided educational materials on LCS and patient navigation (Appendix L), and offered access to an LCS navigator. The navigator's principal role is to guide participants through the LCS process. The navigator's secondary role is to provide a brief tobacco intervention for participants who currently smoke. These roles are described below:

1) LCS navigation: The navigator will introduce the concept of LCS and assist participants in scheduling and attending a shared decision-making (SDM) visit with their PCP to discuss the test in greater depth. The navigator will proactively reach out to PCPs about this via electronic messaging through the BHCHP EHR and make him/herself available to assist in the LCS process. If a PCP and a participant jointly decide to pursue LCS, the navigator will assist with booking the LDCT appointment at a local facility that offers this test and interprets it according to the Lung-RADS framework.[80] BHCHP maintains strong clinical ties with Massachusetts General Hospital, Boston Medical Center, and several other area hospitals, enhancing the feasibility of referring patients to these facilities for LDCT. The navigator will work collaboratively with participants to reduce the barriers to LCS completion while building participants' self-efficacy. If a participant has difficulty obtaining LDCT because of lack of insurance, the navigator will connect the participant with a BHCHP benefits specialist to enroll in health insurance under the state's universal coverage system. Following LDCT, the navigator will coordinate a follow-up appointment with a participant's PCP to discuss the results and arrange any necessary follow-up testing. Throughout this process, the navigator will provide reminder phone calls about upcoming appointments, arrange for transportation assistance and other enabling services when needed, and reinforce the content and concepts covered in the LCS SDM visit through in-person or phone contact. Data from our survey of homeless smokers at BHCHP indicate that over 70% have mobile phones, which will facilitate communication between the navigator and study participants. In addition to documenting their encounters and activities with patients for research data collection purposes (see section VI.A.), the navigator will document within the patients' BHCHP EHR for their care teams to reference as needed.

The navigator will work with each participant for up to 6 months to receive the initial LCS LDCT scan. If a participant receives their initial LCS LDCT scan within the 6-month timeframe and requires follow-up diagnostic testing of abnormal results anytime sooner than the usual LCS LDCT screening interval of 12 months, the navigator will continue to work with the participant until the follow-up diagnostic testing is completed or until the participant no longer wishes to be navigated.

2) Brief tobacco intervention: The navigator will provide a brief tobacco intervention and

distribute educational handouts on smoking cessation (on an ad hoc basis; Appendix M) consistent with the “5 A’s” approach.[81] Within this framework, the navigator will Ask participants if they currently smoke cigarettes and Advise current smokers to quit, emphasizing the connection between smoking and lung cancer and the importance of quitting smoking to lower their lung cancer risk. The navigator will then Assess current smokers’ willingness to quit and Assist them in accessing no-cost or low-cost smoking cessation resources, including:

a) BHCHP PCPs, to discuss smoking cessation medications (e.g. nicotine replacement products, bupropion, or varenicline), which are covered by the state Medicaid program with a $1-3 copayment at retail pharmacies or at no charge through the BHCHP pharmacy. Over 80% of BHCHP patients are insured by the Massachusetts Medicaid program under the state’s universal health insurance system.[68]

b) BHCHP tobacco counselors, who are formally trained in tobacco counseling and conduct weekly support groups at 4 BHCHP venues and individual counseling sessions at 5 BHCHP venues, all free of charge.

c) The Massachusetts Smokers’ Helpline, a free and confidential phone counseling service, for participants with mobile phones. Participants who call the Massachusetts Smokers’ Helpline may receive up to 4 weeks of nicotine replacement therapy (patch, gum, or mini-lozenge) at no cost.

The navigator will then arrange a time to follow-up with current smokers either by phone or in person to discuss their progress on smoking and their engagement in available services. For participants who undergo LDCT, the navigator will conduct a follow-up brief intervention to head off false reassurance about smoking if the findings are negative. Brief tobacco interventions are an evidence-based, effective treatment approach to promoting smoking cessation.[81,82] The brief intervention strategy described above acknowledges the centrality of smoking cessation in reducing lung cancer deaths, capitalizes on the “teachable moment” afforded by the LCS process,[83] satisfies the CMS requirements for LCS LDCT approval,[21] and complements our pragmatic trial design by working within existing tobacco cessation resources available to the target population.

V. Study Procedures

A. Study visits and measurements

Participants who are screened and deemed eligible to participate, whether in person or over the phone, will be asked to complete a research visit during which enrollment, collection of baseline data, and randomization to study arm will occur. Unless non-essential in-person research activities are significantly restricted due to the COVID-19 pandemic, participants will meet with the research coordinator in person at a BHCHP clinic site to complete these activities. After verbal consent and the written ROI form is completed, a study coordinator will meet with the participant to administer a 30-minute baseline survey. In a similar fashion to the verbal consent, the survey can be completed in one of the following ways depending on current pandemic status and/or participant/staff preferences: a) “telephone booth” approach where the research coordinator would conduct the survey on an encrypted laptop via Microsoft Teams or Enterprise Zoom with the participant in a dedicated space with an encrypted study tablet; b) meet in the clinic lobby in a screened off area while maintaining at least 6 feet distance; c) meet in an enclosed BHCHP clinic room while maintaining at least 6 feet distance of safe to do so; or d) over the phone if the participant has adequate access to their own phone and phone plan (only available if in-person non-essential research activities are completely restricted).

Participants will receive a one-time $25 gift card upon completion of the baseline survey in recognition of their time and effort. If participants complete the baseline survey over the phone, research staff will offer participants the chance to pick up the study gift card in-person at JYP or via mail if the participant has a mailing address.

Arm 2 participants will additionally have a series of ad hoc meetings with the LCS navigator as part of the intervention. These meetings may occur in person or over the phone. The navigator will be asked to log the date, time, and nature of activities carried out during these navigation sessions/meetings.

Due to the nature of the primary outcome, which will be ascertained via medical record review, no formal research follow-up visit is necessary. The screening, baseline, and outcome variables for the study are detailed in section VI.A.

40 participants in Arm 1 will participate in post-intervention interviews and be stratified according to whether they did (N=20) or did not (N=20) attain the primary outcome of completing a lung screening CAT scan within six months of initial enrollment. Participants who agree to participate will go through a separate verbal consent process as illustrated previously. Interviews will occur after at least 6 months of exposure to the navigation intervention. The interviews will last up to 45 minutes and be audio recorded. The selection of qualitative participants following ascertainment of the primary quantitative outcome avoids introducing a treatment effect from the interviews while remaining temporally close enough to the LCS process to facilitate accurate recall. These 40 participants will receive a one-time $25 gift card upon completion of the interview in recognition of their time and effort.

Additionally, we will interview 10 BHCHP PCPs among those who had at least 1 patient enrolled in the trial. Providers who agree to participate will provide verbal consent as illustrated previously. Interviews will last up to 45 minutes and be audio recorded. Interviews will occur following completion of the trial (at least 6 months after the last patient participant is enrolled) to avoid influencing provider practice around LCS ordering and follow-up. The 10 PCPs will receive a one-time $25 gift card upon completion of the interview in recognition of their time and effort.

Study personnel will follow various safety precautions to prevent COVID-19 transmission. For patients that are screened for eligibility over the phone and interested in scheduling an in-person study visit, study personnel will administer a screening instrument for COVID-19 symptoms and recent positive COVID-19 tests within 72 hours prior to the scheduled in-person study visit. Prior to entering BHCHP clinic buildings, per BHCHP policy, all patients are already required to wear a face mask and be screened for COVID-19 symptoms and recent positive COVID-19 tests at the door. If BHCHP ceases these screening procedures, study personnel will implement the same screening measures at the start of any in-person visit and will continue to require participants to wear a face mask while engaging with study staff. If a patient endorses any COVID-19 symptoms, study personnel will immediately cease study activities and notify a clinic staff of the presence of a symptomatic patient.

If rising COVID-19 rates necessitate temporary restrictions on all in-person study activities at BHCHP facilities, we will transition to remotely conducting as many study activities as possible until the restrictions are lifted. Study personnel will able to screen, perform verbal informed consent, conduct the baseline survey, perform qualitative interviews, and provide navigation services over the phone or by the HIPAA-compliant video conferencing platforms Microsoft

Teams or Enterprise Zoom depending on patient preference and access to technology (i.e., owning a device with adequate minutes or video conferencing capabilities). Study personnel will always follow the MGB requirements for using Teams or Zoom with no study visits or activities being audio- or video-recorded within the Teams or Zoom platform. Microsoft Teams will be the preferred platform to use for video call study visits, but in the event of technical difficulties, study personnel will use Enterprise Zoom as a backup platform.

B. Drugs

While the LCS navigator will assist participants who currently smoke with accessing smoking cessation resources, neither the navigator nor study staff will provide participants with medications. Participants in both arms may choose to seek smoking cessation medications through their BHCHP PCP or other health care provider.

C. Devices

No devices will be used in this study.

D. Procedures

There are no procedures or surgical interventions in this study. Participants may elect to undergo LCS with LDCT after consultation with their PCP (and with the LCS navigator, for those in Arm 2), but the ordering and oversight of LDCTs will be handled by BHCHP PCPs as per usual practice for participants in both study arms.

E. Data Collection

We will collect all study data electronically using Partners-approved laptops or tablet devices with full disk encryption. Participant data will be recorded, saved, and managed using the Partners-hosted Research Electronic Data Capture (REDCap) program with data collection forms custom-designed for this study. We will also use BHCHP EHR to collect information from participants' medical records that relates to their general health history, any charted mental health problems, and any other information that directly relates to lung cancer screening and smoking. The specific data elements collected at each study visit are detailed below in section VI.A.

VI. BIOSTATISTICAL ANALYSIS

A. Specific data variables

1) Screening variables: Screening variables will include self-reported measures related to the inclusion and exclusion criteria for study participation. These will include the following variables, assessed as shown in Appendix A:
- First and last name
- Date of birth
- Age
- Preferred language
- BHCHP PCP
- Preferred BHCHP clinic site
- History of homelessness

- History or symptoms of lung cancer
- Recent non-lung cancer or cancer treatment
- Home oxygen use
- Cigarette smoking history

2) Contact form variables: Enrolled participants will be asked to complete a contact information form. This information will be valuable for following up with participants who cannot complete the baseline survey on the same day as enrollment, as well as for the patient navigator to aid with patient outreach and EHR documentation. This form will include the following variables, assessed as shown in Appendix O:
- BHCHP Epic MRN (entered by the research coordinator from patient's EHR)
- Last 4 digits of social security number
- Phone number (if applicable)
- Address/location of usual nighttime residence
- Alternative contact (who may know of participant's whereabouts)
- Contact in the case of an emergency
- Participant photograph (with participant permission) to facilitate subsequent contact with patient navigator

3) Baseline variables: Enrolled participants will be asked to complete a baseline assessment (Appendix D) of variables mapping to core domains of the Health Belief Model in addition to other key factors that could impact one or more dimensions of the Health Belief Model, as displayed in Table 2.

4) BHCHP electronic health record variables: The following information will be collected from the review of patients' BHCHP electronic health records:
- History of care with BHCHP and BHCHP PCP prior to study enrollment
- Comorbid health conditions.
- Documentation of a shared decision making (SDM) appointment with PCP to discuss lung cancer screening.
- Documentation of referrals for lung CT scans.
- Documentation of follow-up appointments with PCP that address results of lung cancer screening.
- Documented smoking status (and any changes in smoking status during study enrollment).
- Documented prescriptions to any smoking cessation medications
- Documented insurance status and insurance type.

Table 2. Baseline measures of study participants corresponding to Health Belief Model (HBM) domains and other key factors.

**HBM domains**	**Measures**
**Sociodemographic factors**	Age; sex; race/ethnicity; education; (all based on items from the 2003 HCH User Survey[23,84] and our prior survey work with homeless individuals[33,34]); homelessness onset, episodes, and duration[1]; current living situation
**Perceived lung cancer susceptibility/severity**	Perceived risk of lung cancer and smoking, perceived severity of lung cancer, worry about lung cancer, (all developed by Dr. Park for NLST)[76,85];
**Cues for LCS**	Prior LCS recommendation by health care provider; prior exposure to LCS brochures, flyers, posters, or advertisements
**Perceived LCS**	Lung cancer screening knowledge[86]; perceived benefits of quitting

**benefits/barriers**	smoking[76,85], comfort navigating hospital/health system; health insurance status[23,84]; competing priorities for meeting subsistence needs[22]
**Self-efficacy for LCS**	General self-efficacy scale[87,88]; self-efficacy for completing LDCT
**Other key factors**	**Measures**
**Tobacco use**	Duration of smoking; past quit attempts; current cigarettes/day; alternative tobacco use[35,89,90], Fagerstrom Test for Nicotine Dependence[91]; stage of change[92,93]; Contemplation Ladder[94]
**COVID-19**	Comfort navigating hospital/attending medical appointment during COVID-19 outbreak[95-97]; Previous history of COVID-19 And Lung Infection/Pneumonia
**Comorbid conditions**	Alcohol Use Disorders Identification Test-Concise (AUDIT-C)[98,99];! Drug Abuse Screening Test (DAST-10)[100,101], and K6 screen for psychological distress[102,103]; single-item general health status (SF-1)[104,105]

3) Navigation log: The navigator will complete a log (Appendix E) capturing the following information each time he/she interacts with a participant in Arm 2.
- Name of study staff who completes navigation activity
- Date and time
- Participant ID number
- Who was contacted for navigation activity
- Contact modality for navigation activity
- Tasks/activities undertaken during encounter

4) Qualitative interview data elements:

Participant interviews: Participants will be asked to articulate their thought process in considering whether to pursue LCS and to identify the factors that most shaped their decision, including logistical, physical, or emotional barriers and facilitators. Based on our Health Belief Model framework (Table 2), we will prompt participants to consider the impact of the navigator on a) altering their perceived susceptibility to lung cancer, b) providing them with cues or reminders to pursue LCS, c) enhancing their self-efficacy for pursuing LCS, and d) helping them to overcome barriers to completing LCS. Among participants who undergo LDCT, we will assess their perspectives on the ease of the process, its impact on lung cancer worry, perceived lung cancer risk, and smoking behavior, and the need for any follow-up testing.

PCP interviews: These interviews will elicit PCP perspectives on the challenges of implementing LCS guideline in primary care practice for a vulnerable population and probe the ways in which the navigator did or did not help to reduce these challenges. We will also seek feedback from PCPs on ways to strengthen the integration of patient navigators into clinical care teams.

<u>B. Study Endpoints</u>

1) Primary outcome: The primary outcome is receipt of LDCT for LCS at 6 months. This will be based on radiology records verifying that a chest CT was performed for LCS and interpreted according to the Lung-RADS framework.[80] To ascertain this outcome, study staff will ask participants at the time of enrollment to provide written consent to obtain radiology records from

Boston-area facilities that perform LDCT for LCS, as described above. At 6 months after enrollment, radiology records will be requested/obtained from these facilities. Most Boston-area hospital records are accessible through Care Everywhere in the BHCHP version of Epic, supporting the feasibility of gathering this information in an efficient manner. This approach does not rely on participant self-report and therefore does not require in-person or phone follow-up to ascertain. For those verified to have undergone LDCT for LCS within the 6-month timeframe, the Lung-RADS result along with any abnormal or incidental findings will be recorded.

2) Secondary outcome: Increasing LDCT completion will only reduce lung cancer mortality if individuals with abnormal results can undergo evaluation and treatment of early stage lung cancer in a timely fashion. In the setting of homelessness, a valid concern is whether participants with abnormal LDCT results can be recalled for diagnostic follow-up in a timely manner. To address this concern, our secondary outcome is receipt of LDCT with timely diagnostic follow-up of any abnormal results. To satisfy this composite outcome, participants must achieve the primary outcome (LDCT at 6 months) <u>and</u>, if the result is abnormal, also obtain the next recommended follow-up test within 1 month of the advised timeframe, based on the Lung-RADS framework.[80] Lung-RADS category descriptions, recommended follow-up, associated probabilities of malignancy, and estimated general population prevalences are shown in Table 3.[104] As described above, we will obtain radiology records for participants who underwent LDCT for LCS to document the findings of the study, determine the Lung-RADS category associated with those findings, and ascertain the recommended diagnostic follow-up plan. Participants with Lung-RADS 1-2 findings do not require specific diagnostic action, but rather may continue with annual screening at 12 months. These individuals will automatically satisfy the secondary outcome. In contrast, Lung-RADS 3-4 findings signal an increased risk for malignancy and merit sooner-than-annual follow-up testing. These individuals will satisfy the secondary outcome only if they obtain the next recommended diagnostic study within 1 month of the recommended timeframe. For example, if a participant has a Lung-RADS 3 finding for which follow-up LDCT in 6 months is recommended, we will assess whether this follow-up test occurred within 7 months of the initial LDCT. If a participant has a Lung-RADS 4B/X finding suggesting a high suspicion for malignancy, we will assess whether the recommended follow-up testing occurred within 1 month of the initial LDCT. The offer of delayed navigation to control arm participants will not interfere with this outcome because a) delayed navigation will be offered only to those who did not undergo an initial LCS LDCT within 6 months (i.e. the primary outcome), and b) attaining the primary outcome is necessary for satisfying the secondary outcome.

Table 3. Lung-RADS categories.

Category	Description	Management	Malignancy probability	Gen. pop. prevalence
1	Negative	Repeat LDCT 12 mos	<1%	90%
2	Benign			
3	Probably benign	Repeat LDCT 6 mos	1-2%	5%
4A	Suspicious	Repeat LDCT 3 mos +/- PET/CT	5-15%	2%
4B/X		Chest CT, PET/CT, +/- biopsy	>15%	2%

*Adapted from the American College of Radiology

3) Other outcomes: We will examine time to receipt of LDCT for LCS. Process outcomes include acceptance of navigation when offered, number of contacts with the navigator, and attendance of a LCS SDM visit with a BHCHP PCP. Tobacco-related outcomes include receipt of a prescription for any smoking cessation medication and changes in clinically-recorded smoking status (i.e. quitting among current smokers or relapsing among former smokers), both ascertained by BHCHP medical record review. We will track the frequency and types of abnormal findings detected on LCS LDCT, any additional diagnostic testing or therapeutic changes pursued in response to these findings, and the percentage of participants diagnosed

with lung cancer (based on radiology and BHCHP records review). We will also track diagnostic chest CT scans obtained for reasons other than LCS.

C. Statistical Methods

Quantitative analysis: LDCT completion at 6 months (1° outcome) and LDCT completion with timely diagnostic follow-up (2°outcome) are both binary outcomes. In the principal analysis, we will assess the difference between the immediate and delayed navigation arms on attainment of these outcomes using the Chi-square test. We will also use logistic regression models to examine the associations between treatment condition and outcomes including clinical site and other known predictors of outcomes to increase the precision of effect estimates. Additional analyses will examine time to receipt of LDCT for LCS between study arms using time-to-event methods (log-rank tests and Cox regression models). Although we expect to be underpowered to detect significant interactions, we will examine whether the effect of navigation varies by smoking status, housing status, or clinical site in an exploratory analysis.

Qualitative analysis: Qualitative interviews will be audiotaped and transcribed. We will use inductive content analysis methods to identify major and minor themes, and we will use deductive methods to strengthen, refine, and enhance understanding of identified themes.[105] Qualitative data coding will be performed by 2 trained study staff members using NVivo 11 software. The data coders will meet regularly with Drs. Baggett and Park to discuss the structural thematic framework, categories, and coding plan. We will resolve discrepancies through discussion and comparison to raw data. Coding will continue iteratively until a high level of reliability (Kappa>0.80) is established.

Integration with quantitative data: Based on a mixed methods approach,[106] we will use the qualitative interview data to interpret and explain the quantitative observations from the trial. This will aid in better understanding why overall LDCT completion was higher or lower than expected and why the navigation intervention did or did not improve these outcomes. Following from our stratified sampling scheme, we will integrate the quantitative and qualitative data by assessing for thematic differences in the interview content of participants who did (N=20) and did not (N=20) attain the primary outcome. We will focus in particular on comparing and contrasting these two groups' views on the utility of the navigator with respect to the Health Belief Model domains of perceived susceptibility, cues, self-efficacy, and barriers, as described above. We will additionally incorporate the quantitative baseline measures tapping these Health Belief Model domains into integrated analyses. Table 4 illustrates the way in which these quantitative and qualitative data elements will be integrated and presented in a blended fashion.

Table 4. Proposed integration of quantitative and qualitative data.

<table>
<tr><th rowspan="2">Health Belief Model domains</th><th rowspan="2">Measurement (method)</th><th colspan="2">LCS LDCT at 6 months</th></tr>
<tr><th>Yes (N=20)</th><th>No (N=20)</th></tr>
<tr><td rowspan="2">Perceived susceptibility / severity</td><td>Baseline survey (quan)</td><td colspan="2">Lung cancer risk perception, severity, and worry scores</td></tr>
<tr><td>Post-intervention interview (qual)</td><td colspan="2">Quotes/themes about navigator impact on perceived susceptibility/severity</td></tr>
<tr><td rowspan="2">Cues</td><td>Baseline survey (quan)</td><td colspan="2">Past provider recommendation for LCS, exposure to LCS information</td></tr>
<tr><td>Post-intervention interview (qual)</td><td colspan="2">Quotes/themes about navigator provision of cues for LCS</td></tr>
<tr><td rowspan="2">Self-efficacy</td><td>Baseline survey (quan)</td><td colspan="2">General and LCS-specific self-efficacy scores</td></tr>
<tr><td>Post-intervention interview (qual)</td><td colspan="2">Quotes/themes about navigator impact on self-efficacy</td></tr>
<tr><td rowspan="2">Perceived benefits / barriers</td><td>Baseline survey (quan)</td><td colspan="2">LCS knowledge, competing subsistence difficulty scores</td></tr>
<tr><td>Post-intervention interview (qual)</td><td colspan="2">Quotes/themes about navigator assistance with barriers</td></tr>
</table>

D. Power Analysis

The sample size justification is based on the primary outcome of LCS LDCT completion at 6 months. Internal BHCHP data suggests a presently negligible baseline rate of LCS LDCT. Allowing for improvement in this rate as LCS comes into wider practice, we will assume that 5% of usual care (Arm 1) participants will obtain an LDCT within 6 months. In our judgment, increasing this completion rate to 15% would represent a clinically meaningful change. We will have >80% power to detect such a difference with a sample size of 200 in the navigation arm (Arm 2) and 100 in the usual care arm (Arm 1).

VII. RISKS AND DISCOMFORTS

The potential risks and discomforts associated with this study are detailed below. The methods for mitigating these risks and monitoring the safety of participants are described in section IX.b.

A. Complications of surgical / non-surgical procedures

Not applicable.

B. Drug side effects and toxicities

Study staff will not be offering any drugs or over-the-counter products to participants.

C. Device complications

Not applicable.

D. Psychosocial (non-medical) risks

Potential risks to participants include psychological distress arising from the possibility of false-positive CT findings leading to additional diagnostic studies or procedures. Psychological and/or physical distress may also be brought about by discussing smoking and/or changing smoking behavior. Finally, because we will collect and record identifying information (e.g. name, date of birth, and social security number), there is a risk to privacy if the data are compromised. Section IX (Monitoring and Quality Assurance) describes our methods for safeguarding the data and privacy of participants. The potential risks of this study are reasonable in relation to the potential benefits of reducing the risk of dying from lung cancer.

E. Radiation risks

There is a slight physical risk associated with exposure to radiation during CT scanning, although the “low-dose” nature of the test is designed to help mitigate this risk. For this reason, LDCT LCS for individuals at increased risk of lung cancer is recommended by a variety of professional societies since the benefits of screening for early stage cancer vastly outweigh the risks presented by small radiation exposures.

VIII. POTENTIAL BENEFITS

A. Potential benefits to participating individuals

Potential benefits for participants include the opportunity to receive assistance in completing LCS, which has been associated with a 20% reduction in lung cancer mortality. Additionally, current smokers will be informed of and referred to various resources to help them quit smoking, which may confer numerous health and financial benefits.

B. Potential benefits to society

Considerable knowledge could be gained from this study. To our knowledge, this will be the first study to focus on LCS among homeless people and the first randomized trial of any cancer screening intervention in this population. By focusing on an understudied topic area pertaining to a common cause of death among homeless people, our study fills an important gap in the literature and could serve as a blueprint for improving cancer screening and outcomes in homeless health care settings, particularly in the 295 HCH programs that serve 890,000 people annually in the US.[107]

IX. MONITORING AND QUALITY ASSURANCE

A. Data monitoring

All data will be collected using password-protected laptops or tablets with full disk encryption. We will use the Partners-hosted Research Electronic Data Capture (REDCap) application for data collection and management. REDCap is a secure, HIPAA compliant web-based application hosted by Partners HealthCare Research Computing, Enterprise Research Infrastructure & Services (ERIS). Because a Partners username is required for logging in to REDCap, all activity on study documents is electronically logged and therefore traceable. All data will be collected according to a standardized protocol preprogrammed into REDCap, which has built-in functions providing real-time data entry validation to help ensure accuracy and completeness. Each data collection form will have instructions or prompts for collecting the required data elements, and the response fields for each item will have appropriate ranges and formats to ensure that the data is entered in a valid way. Participants who agree to be photographed will have their photo taken using the same device on which the rest of the data is collected (encrypted laptop or tablet). The photograph will be uploaded immediately to the REDCap database and the original photograph file will be permanently deleted from the device.

The PI will review all study data weekly to monitor its integrity. Additionally, the PI, biostatistician, and/or data analyst will download the study data on a weekly basis from the Partners REDCap server and back it up to the PI's SFA on the Partners network, which is protected by the Partners IS firewall, backed up nightly, and accessible only to authorized study staff. Only the minimum necessary number of study staff will have access to this data. Data analyses will be conducted by authorized study staff on the data files residing on the PI's Partners SFA. The data will not be transferred to investigators outside the Partners system.

The qualitative interviews conducted post-intervention will be recorded by a microphone connected directly to a password-protected, Partners-approved laptop or tablet with full-disk encryption. Audio files will be backed up to the PI's SFA described above and then deleted from the portable device.

If study activities are administered to participants using Enterprise Zoom or Microsoft Teams, personnel will always follow the MGB requirements for using video conferencing for research activities with no study visits or activities being audio- or video-recorded, with the exception of qualitative interviews, which will be audio-recorded with an encrypted laptop or tablet microphone (and not via the audio-recording capabilities within the Zoom or Teams platform) as described above. Study personnel will always follow the Principles of Privacy and share the minimum necessary amount of information.

B. Safety monitoring

Because of the narrow range of risks of the proposed study, there will not be a Data and Safety Monitoring Board for this trial. Instead, the safety of participants will be monitored by the PI on a continual basis. Navigators will stay in close contact with BHCHP PCPs and clinical staff to ensure the safety and well-being of all participants.

To minimize the risk to privacy, we will adhere to the data management procedures described above to help ensure the safety and integrity of data collected about study participants. All identifying information will be saved securely within the REDCap database. Paper ROI forms will be scanned into the PI's SFA and paper copies retained in a locked cabinet in the PI's office. Only the minimum necessary number of study staff will have access to this data. At the conclusion of the study, personal identifiers will be removed from the dataset and all analyses will occur in a de-identified fashion.

To help ensure the safety of participants, adverse events monitoring will be handled in the manner described below.

C. Outcomes monitoring

Because of the low-risk nature of the intervention, we will not conduct a formal interim analysis of the data nor define rules for early stopping of the trial.

D. Adverse event reporting guidelines

We will adhere to the Partners policy statement on "Reporting Unanticipated Problems including Adverse Events." Consistent with this policy, an adverse event will be defined as "any untoward or unfavorable medical occurrence in a human subject, including any abnormal sign, symptom, or disease, temporally associated with the subject's participation in the research, whether or not considered related to the subject's participation in the research." The PI and his designated study staff will be responsible for the monitoring of adverse events in study participants.

Adverse events reported to the PI or other study staff will be documented in an adverse event report containing a description of the event, the date and time of onset, the date and time of resolution, the expectedness of the event, the relationship to the study, the seriousness of the event, and the action taken in response to the event. An adverse event will be deemed unexpected if the nature, severity, or frequency of the event is not consistent with a known or foreseeable risk given the research procedures, the characteristics of the subject population, or with an expected progression of an underlying condition. The following attribution scale will be used to describe the relationship of the adverse event to the study protocol: unrelated to the protocol, or possibly, probably, or definitely related to the protocol. A serious adverse event will be defined as an adverse event that results in death, is life-threatening, requires hospitalization, causes persistent or significant disability, or requires medical or surgical intervention.

A summary safety report detailing all adverse events and their handling will be included in annual study progress reports to the Partners IRB and the American Cancer Society. Unexpected adverse events of any severity that are at least possibly related to the research and suggest the research puts participants at increased harm will be reported to the Partners IRB within 5 working days or 7 calendar days. Serious adverse events that are unexpected and at least possibly related to the research will be assumed to suggest that the research puts participants at increased harm and will be reported to the Partners IRB within the same time frame. Any action resulting in a temporary or permanent suspension of this study will be reported to the American Cancer Society's program official. Adverse event reports and annual safety summaries will be documented by study ID number; personal identifiers will not be included in these reports.

<u>E. Protocol adherence</u>

1) Research staff: The study coordinators will be trained on the data collection protocols described above. Their understanding of these protocols will be assessed by asking them to demonstrate competency in using the REDCap data collection forms.

2) LCS navigator: To ensure adherence to the navigation intervention strategy, the navigator will receive in-depth training on patient navigation and focused training on tobacco cessation counseling.

a) Navigation training: The navigator will receive training in the core skills of patient navigation, including establishing rapport with patients while maintaining appropriate professional boundaries, motivational interviewing, performing an initial assessment with patients to explore barriers to LCS, working with patients to overcome these barriers, educating patients about the LCS process, introducing the concept of shared decision-making (SDM) around LCS, and empowering participants to discuss the benefits and risks of LCS with their PCP. Training will also encompass an overview of lung cancer, the rationale behind LCS, and the practical steps involved in completing LDCT so that the navigator can better counsel patients on what to expect from the LCS process. Additionally, the navigator will be trained in identifying and managing logistical hurdles to completing LCS, such as insurance coverage lapses that may need to be remedied before LDCT is feasible. To facilitate timely follow-up of abnormal LDCT results, the navigator will received focused education on the American College of Radiology (ACR) Lung Imaging Reporting and Data System (Lung-RADS) framework.[80] Lung-RADS is a quality assurance tool designed to standardize LCS LDCT reporting and management recommendations, reduce confusion in LCS LDCT interpretations, and facilitate outcome monitoring.[80] The navigator training will be based on the protocol developed for Dr. Percac-Lima 's successful RCT of patient navigation for LCS among community health center patients (see section E). Dr. Percac-Lima, who has extensive experience in navigator training, will provide key components of this training and facilitate role playing sessions to reinforce skills. To the greatest extent possible, experienced navigators will be included in the training process. Depending upon prior experience, the navigator trainee will spend up to 2 weeks shadowing and observing an experienced navigator in the field prior to the start of the study. Refresher training and review of specific case scenarios will occur every 3 months throughout the study to ensure that the navigator is adhering faithfully to the intended roles and responsibilities.

b) Tobacco counseling training: The goal of the tobacco training component is to equip the navigator with the skills to provide a brief tobacco intervention. To achieve this goal, the navigator will complete an online 9-module course titled "Basic Skills for Working with Smokers," sponsored by the University of Massachusetts Center for Tobacco Treatment Research &

Training.[108] This course provides an introduction to the theory and practice of working with smokers in a health care setting. The navigator will additionally receive 4 hours of case-based teaching from Dr. Baggett on practical approaches to working with homeless smokers, followed by 6 hours of observing a certified Tobacco Treatment Specialist interact with and counsel smokers about tobacco cessation.

The navigator's adherence to their intended duties will be assessed during weekly team meetings and through periodic audit of their navigation encounter logs.

X. REFERENCES

1. Burt MR, Aron LY, Douglas T, et al. Homelessness: Programs and the People They Serve: Findings of the National Survey of Homeless Assistance Providers and Clients: Technical Report. In. Washington, D.C.: U.S. Department of Housing and Urban Development, Office of Policy Development and Research; 1999.
2. Baggett TP, Chang Y, Porneala BC, Bharel M, Singer DE, Rigotti NA. Disparities in Cancer Incidence, Stage, and Mortality at Boston Health Care for the Homeless Program. *Am J Prev Med.* 2015;49(5):694-702.
3. Lamont DW, Toal FM, Crawford M. Socioeconomic deprivation and health in Glasgow and the west of Scotland--a study of cancer incidence among male residents of hostels for the single homeless. *J Epidemiol Community Health.* 1997;51(6):668-671.
4. Hwang SW, Wilkins R, Tjepkema M, O'Campo PJ, Dunn JR. Mortality among residents of shelters, rooming houses, and hotels in Canada: 11 year follow-up study. *BMJ.* 2009;339:b4036.
5. Baggett TP, Hwang SW, O'Connell JJ, et al. Mortality among homeless adults in Boston: shifts in causes of death over a 15-year period. *JAMA Intern Med.* 2013;173(3):189-195.
6. Baggett TP, Chang Y, Singer DE, et al. Tobacco-, alcohol-, and drug-attributable deaths and their contribution to mortality disparities in a cohort of homeless adults in Boston. *Am J Public Health.* 2015;105(6):1189-1197.
7. Aberle DR, Adams AM, Berg CD, et al. Reduced lung-cancer mortality with low-dose computed tomographic screening. *N Engl J Med.* 2011;365(5):395-409.
8. Chau S, Chin M, Chang J, et al. Cancer risk behaviors and screening rates among homeless adults in Los Angeles County. *Cancer Epidemiol Biomarkers Prev.* 2002;11(5):431-438.
9. Lebrun-Harris LA, Baggett TP, Jenkins DM, et al. Health status and health care experiences among homeless patients in federally supported health centers: findings from the 2009 patient survey. *Health services research.* 2013;48(3):992-1017.
10. Asgary R, Garland V, Jakubowski A, Sckell B. Colorectal cancer screening among the homeless population of New York City shelter-based clinics. *Am J Public Health.* 2014;104(7):1307-1313.
11. Asgary R, Garland V, Sckell B. Breast Cancer Screening Among Homeless Women of New York City Shelter-Based Clinics. *Womens Health Issues.* 2014.
12. Long HL, Tulsky JP, Chambers DB, et al. Cancer screening in homeless women: attitudes and behaviors. *J Health Care Poor Underserved.* 1998;9(3):276-292.
13. Baggett TP, Rigotti NA. Cigarette smoking and advice to quit in a national sample of homeless adults. *American Journal of Preventive Medicine.* 2010;39(2):164-172.
14. Connor SE, Cook RL, Herbert MI, Neal SM, Williams JT. Smoking cessation in a homeless population: there is a will, but is there a way? *Journal of General Internal Medicine.* 2002;17(5):369-372.
15. Snyder LD, Eisner MD. Obstructive lung disease among the urban homeless. *Chest.* 2004;125(5):1719-1725.
16. Szerlip MI, Szerlip HM. Identification of cardiovascular risk factors in homeless adults. *Am J Med Sci.* 2002;324(5):243-246.
17. Tsai J, Rosenheck RA. Smoking Among Chronically Homeless Adults: Prevalence and Correlates. *Psychiatr Serv.* 2012;63(6):569-576.
18. Torchalla I, Strehlau V, Okoli CT, Li K, Schuetz C, Krausz M. Smoking and predictors of nicotine dependence in a homeless population. *Nicotine Tob Res.* 2011;13(10):934-942.
19. Hahn JA, Kushel MB, Bangsberg DR, Riley E, Moss AR. BRIEF REPORT: the aging of the homeless population: fourteen-year trends in San Francisco. *J Gen Intern Med.* 2006;21(7):775-778.

20. Moyer VA. Screening for lung cancer: U.S. Preventive Services Task Force recommendation statement. *Ann Intern Med.* 2014;160(5):330-338.
21. Jenson TS, Chin J, Ashby L, Hermansen J, Hutter JD. Decision Memo for Screening for Lung Cancer with Low Dose Computed Tomography (LDCT) (CAG-00439N). . In. Baltimore, MD: Centers for Medicare & Medicaid Services; 2015.
22. Gelberg L, Gallagher TC, Andersen RM, Koegel P. Competing priorities as a barrier to medical care among homeless adults in Los Angeles. *Am J Public Health.* 1997;87(2):217-220.
23. Baggett TP, O'Connell JJ, Singer DE, Rigotti NA. The unmet health care needs of homeless adults: a national study. *Am J Public Health.* 2010;100(7):1326-1333.
24. Kushel MB, Vittinghoff E, Haas JS. Factors associated with the health care utilization of homeless persons. *JAMA.* 2001;285(2):200-206.
25. Weinstein LC, LaNoue M, Hurley K, Sifri R, Myers R. Using Concept Mapping to Explore Barriers and Facilitators to Breast Cancer Screening in Formerly Homeless Women with Serious Mental Illness. *J Health Care Poor Underserved.* 2015;26(3):908-925.
26. Fazel S, Khosla V, Doll H, Geddes J. The prevalence of mental disorders among the homeless in western countries: systematic review and meta-regression analysis. *PLoS Med.* 2008;5(12):e225.
27. Koegel P, Burnam MA, Farr RK. The prevalence of specific psychiatric disorders among homeless individuals in the inner city of Los Angeles. *Arch Gen Psychiatry.* 1988;45(12):1085-1092.
28. Brown RT, Kiely DK, Bharel M, Mitchell SL. Geriatric syndromes in older homeless adults. *J Gen Intern Med.* 2011;27(1):16-22.
29. Topolovec-Vranic J, Ennis N, Colantonio A, et al. Traumatic brain injury among people who are homeless: a systematic review. *BMC Public Health.* 2012;12:1059.
30. Topolovec-Vranic J, Ennis N, Howatt M, et al. Traumatic brain injury among men in an urban homeless shelter: observational study of rates and mechanisms of injury. *CMAJ Open.* 2014;2(2):E69-76.
31. Hwang SW, Colantonio A, Chiu S, et al. The effect of traumatic brain injury on the health of homeless people. *CMAJ.* 2008;179(8):779-784.
32. Oddy M, Moir JF, Fortescue D, Chadwick S. The prevalence of traumatic brain injury in the homeless community in a UK city. *Brain Inj.* 2012;26(9):1058-1064.
33. Baggett TP, Campbell EG, Chang Y, Magid LM, Rigotti NA. Posttraumatic Stress Symptoms and Their Association With Smoking Outcome Expectancies Among Homeless Smokers in Boston. *Nicotine Tob Res.* 2015.
34. Baggett TP, Rigotti NA, Campbell EG. Cost of Smoking among Homeless Adults. *N Engl J Med.* 2016;374(7):697-698.
35. Baggett TP, Campbell EG, Chang Y, Rigotti NA. Other tobacco product and electronic cigarette use among homeless cigarette smokers. *Addict Behav.* 2016;60:124-130.
36. Percac-Lima S, Ashburner JM, Bond B, Oo SA, Atlas SJ. Decreasing disparities in breast cancer screening in refugee women using culturally tailored patient navigation. *J Gen Intern Med.* 2013;28(11):1463-1468.
37. Percac-Lima S, Lopez L, Ashburner JM, Green AR, Atlas SJ. The longitudinal impact of patient navigation on equity in colorectal cancer screening in a large primary care network. *Cancer.* 2014;120(13):2025-2031.
38. Percac-Lima S, Ashburner JM, Zai AH, et al. Patient Navigation for Comprehensive Cancer Screening in High-Risk Patients Using a Population-Based Health Information Technology System: A Randomized Clinical Trial. *JAMA Intern Med.* 2016;176(7):930-937.
39. Freeman HP. Patient navigation: a community based strategy to reduce cancer disparities. *J Urban Health.* 2006;83(2):139-141.

40. Asgary R, Naderi R, Wisnivesky J. Opt-Out Patient Navigation to Improve Breast and Cervical Cancer Screening Among Homeless Women. *J Womens Health (Larchmt).* 2017.
41. Braun KL, Thomas WL, Jr., Domingo JL, et al. Reducing cancer screening disparities in medicare beneficiaries through cancer patient navigation. *J Am Geriatr Soc.* 2015;63(2):365-370.
42. Myers RE, Sifri R, Daskalakis C, et al. Increasing colon cancer screening in primary care among African Americans. *J Natl Cancer Inst.* 2014;106(12).
43. Honeycutt S, Green R, Ballard D, et al. Evaluation of a patient navigation program to promote colorectal cancer screening in rural Georgia, USA. *Cancer.* 2013;119(16):3059-3066.
44. Wang X, Fang C, Tan Y, Liu A, Ma GX. Evidence-based intervention to reduce access barriers to cervical cancer screening among underserved Chinese American women. *J Womens Health (Larchmt).* 2010;19(3):463-469.
45. Paskett ED, Harrop JP, Wells KJ. Patient navigation: an update on the state of the science. *CA Cancer J Clin.* 2011;61(4):237-249.
46. Phillips CE, Rothstein JD, Beaver K, Sherman BJ, Freund KM, Battaglia TA. Patient navigation to increase mammography screening among inner city women. *J Gen Intern Med.* 2010;26(2):123-129.
47. Robinson-White S, Conroy B, Slavish KH, Rosenzweig M. Patient navigation in breast cancer: a systematic review. *Cancer Nurs.* 2010;33(2):127-140.
48. Wells KJ, Battaglia TA, Dudley DJ, et al. Patient navigation: state of the art or is it science? *Cancer.* 2008;113(8):1999-2010.
49. Chen LA, Santos S, Jandorf L, et al. A program to enhance completion of screening colonoscopy among urban minorities. *Clin Gastroenterol Hepatol.* 2008;6(4):443-450.
50. Jandorf L, Braschi C, Ernstoff E, et al. Culturally targeted patient navigation for increasing african americans' adherence to screening colonoscopy: a randomized clinical trial. *Cancer Epidemiol Biomarkers Prev.* 2013;22(9):1577-1587.
51. Lasser KE, Murillo J, Medlin E, et al. A multilevel intervention to promote colorectal cancer screening among community health center patients: results of a pilot study. *BMC Fam Pract.* 2009;10:37.
52. Christie J, Itzkowitz S, Lihau-Nkanza I, Castillo A, Redd W, Jandorf L. A randomized controlled trial using patient navigation to increase colonoscopy screening among low-income minorities. *J Natl Med Assoc.* 2008;100(3):278-284.
53. Jandorf L, Gutierrez Y, Lopez J, Christie J, Itzkowitz SH. Use of a patient navigator to increase colorectal cancer screening in an urban neighborhood health clinic. *J Urban Health.* 2005;82(2):216-224.
54. Lasser KE, Murillo J, Lisboa S, et al. Colorectal cancer screening among ethnically diverse, low-income patients: a randomized controlled trial. *Archives of internal medicine.* 2011;171(10):906-912.
55. Horne HN, Phelan-Emrick DF, Pollack CE, et al. Effect of patient navigation on colorectal cancer screening in a community-based randomized controlled trial of urban African American adults. *Cancer Causes Control.* 2015;26(2):239-246.
56. Percac-Lima S, Grant RW, Green AR, et al. A culturally tailored navigator program for colorectal cancer screening in a community health center: a randomized, controlled trial. *J Gen Intern Med.* 2009;24(2):211-217.
57. Hoffman HJ, LaVerda NL, Young HA, et al. Patient navigation significantly reduces delays in breast cancer diagnosis in the District of Columbia. *Cancer Epidemiol Biomarkers Prev.* 2012;21(10):1655-1663.

58. Markossian TW, Darnell JS, Calhoun EA. Follow-up and timeliness after an abnormal cancer screening among underserved, urban women in a patient navigation program. *Cancer Epidemiol Biomarkers Prev.* 2012;21(10):1691-1700.
59. Battaglia TA, Bak SM, Heeren T, et al. Boston Patient Navigation Research Program: the impact of navigation on time to diagnostic resolution after abnormal cancer screening. *Cancer Epidemiol Biomarkers Prev.* 2012;21(10):1645-1654.
60. Raich PC, Whitley EM, Thorland W, Valverde P, Fairclough D. Patient navigation improves cancer diagnostic resolution: an individually randomized clinical trial in an underserved population. *Cancer Epidemiol Biomarkers Prev.* 2012;21(10):1629-1638.
61. Ramirez AG, Perez-Stable EJ, Penedo FJ, et al. Navigating Latinas with breast screen abnormalities to diagnosis: the Six Cities Study. *Cancer.* 2013;119(7):1298-1305.
62. Lee JH, Fulp W, Wells KJ, Meade CD, Calcano E, Roetzheim R. Patient navigation and time to diagnostic resolution: results for a cluster randomized trial evaluating the efficacy of patient navigation among patients with breast cancer screening abnormalities, Tampa, FL. *PLoS One.* 2013;8(9):e74542.
63. Lee JH, Fulp W, Wells KJ, Meade CD, Calcano E, Roetzheim R. Effect of patient navigation on time to diagnostic resolution among patients with colorectal cancer-related abnormalities. *J Cancer Educ.* 2014;29(1):144-150.
64. American Cancer Society. Patient Navigators Help Cancer Patients Manage Care. https://www.cancer.org/latest-news/navigators-help-cancer-patients-manage-their-care.html. Published February 24, 2017. Accessed March 1, 2017.
65. National Cancer Institute. Patient Navigation Research Program (PNRP). U.S. Department of Health and Human Services, National Institutes of Health, National Cancer Institute. https://www.cancer.gov/about-nci/organization/crchd/disparities-research/pnrp. Accessed March 28, 2017.
66. O'Connell JJ, Oppenheimer SC, Judge CM, et al. The Boston Health Care for the Homeless Program: a public health framework. *Am J Public Health.* 2010;100(8):1400-1408.
67. Bauer LK, Brody JK, Leon C, Baggett TP. Characteristics of Homeless Adults Who Died of Drug Overdose: A Retrospective Record Review. *J Health Care Poor Underserved.* 2016;27(2):846-859.
68. Bharel M, Lin WC, Zhang J, O'Connell E, Taube R, Clark RE. Health care utilization patterns of homeless individuals in Boston: preparing for Medicaid expansion under the Affordable Care Act. *Am J Public Health.* 2013;103 Suppl 2:S311-317.
69. Tsai D. MassHealth Transmittal Letter PHY-147. In: Office of Medicaid, Massachusetts Department of Health and Human Services; March 2016.
70. Wender R, Fontham ET, Barrera E, Jr., et al. American Cancer Society lung cancer screening guidelines. *CA Cancer J Clin.* 2013;63(2):107-117.
71. Detterbeck FC, Mazzone PJ, Naidich DP, Bach PB. Screening for lung cancer: Diagnosis and management of lung cancer, 3rd ed: American College of Chest Physicians evidence-based clinical practice guidelines. *Chest.* 2013;143(5 Suppl):e78S-92S.
72. Jaklitsch MT, Jacobson FL, Austin JH, et al. The American Association for Thoracic Surgery guidelines for lung cancer screening using low-dose computed tomography scans for lung cancer survivors and other high-risk groups. *J Thorac Cardiovasc Surg.* 2012;144(1):33-38.
73. Samet JH, Crowell R, Estepar RSJ, et al. Providing Guidance on Lung Cancer Screening to Patients and Physicians: An Update from the American Lung Association Lung Cancer Screening Committee. . In. Chicago: American Lung Association; April 30, 2015.

74. Bach PB, Mirkin JN, Oliver TK, et al. Benefits and harms of CT screening for lung cancer: a systematic review. *JAMA.* 2012;307(22):2418-2429.
75. Park ER, Streck JM, Gareen IF, et al. A qualitative study of lung cancer risk perceptions and smoking beliefs among national lung screening trial participants. *Nicotine Tob Res.* 2014;16(2):166-173.
76. Park ER, Ostroff JS, Rakowski W, et al. Risk perceptions among participants undergoing lung cancer screening: baseline results from the National Lung Screening Trial. *Ann Behav Med.* 2009;37(3):268-279.
77. Ryan H, Trosclair A, Gfroerer J. Adult current smoking: differences in definitions and prevalence estimates--NHIS and NSDUH, 2008. *J Environ Public Health.* 2012;2012:918368.
78. Health Resources and Services Administration, Bureau of Primary Health Care. Authorizing Legislation of the Health Center Program: Section 330 of the Public Health Service Act (42 USCS § 254b). http://bphc.hrsa.gov/policiesregulations/legislation/authorizing330.pdf. Accessed March 28, 2017.
79. U.S. Department of Housing and Urban Development. S. 896 HEARTH Act of 2009. https://www.hudexchange.info/resource/1717/s-896-hearth-act/. Accessed March 12, 2017.
80. American College of Radiology. Lung CT Screening Reporting and Data System (Lung-RADS). http://www.acr.org/Quality-Safety/Resources/LungRADS. Accessed March 28, 2017.
81. Fiore MC, Jaen CR, Baker TB, et al. Treating Tobacco Use and Dependence: 2008 Update. Clinical Practice Guideline. . In. Rockville, MD: U.S. Department of Health and Human Services. Public Health Service; May 2008.
82. Park ER, Gareen IF, Japuntich S, et al. Primary Care Provider-Delivered Smoking Cessation Interventions and Smoking Cessation Among Participants in the National Lung Screening Trial. *JAMA Intern Med.* 2015;175(9):1509-1516.
83. Poghosyan H, Kennedy Sheldon L, Cooley ME. The impact of computed tomography screening for lung cancer on smoking behaviors: a teachable moment? *Cancer Nurs.* 2012;35(6):446-475.
84. Greene J, Fahrney K, Byron M. Health Care for the Homeless User/Visit Surveys, RTI Project Number 07147.021. . In. Research Triangle Park, NC: RTI International; 2004.
85. Kaufman AR, Koblitz AR, Persoskie A, et al. Factor Structure and Stability of Smoking-Related Health Beliefs in the National Lung Screening Trial. *Nicotine Tob Res.* 2016;18(3):321-329.
86. Lowenstein LM, Richards VF, Leal VB, et al. A brief measure of Smokers' knowledge of lung cancer screening with low-dose computed tomography. *Prev Med Rep.* 2016;4:351-356.
87. Schwarzer R, Jerusalem M. Generalized Self-Efficacy scale. In: Weinman J, Wright S, Johnston M, eds. *Measures in health psychology: A user's portfolio. Causal and control beliefs.* Windsor, UK: NFER-NELSON; 1995:35-37.
88. Scholz U, Gutierrez Dona B, Sud S, Schwarzer R. Is General Self-Efficacy a Universal Construct? Psychometric Findings from 25 Countries. *European Journal of Psychological Assessment.* 2002;18(3):242-251.
89. US Department of Commerce CB. National Cancer Institute and Food and Drug Administration co-sponsored Tobacco Use Supplement to the Current Population Survey. In. *2018-2019*2020.
90. Kish DH, Reitzel LR, Kendzor DE, Okamoto H, Businelle MS. Characterizing Concurrent Tobacco Product Use Among Homeless Cigarette Smokers. *Nicotine Tob Res.* 2014.

91. Heatherton TF, Kozlowski LT, Frecker RC, Fagerstrom KO. The Fagerstrom Test for Nicotine Dependence: a revision of the Fagerstrom Tolerance Questionnaire. *Br J Addict.* 1991;86(9):1119-1127.
92. DiClemente CC, Prochaska JO, Fairhurst SK, Velicer WF, Velasquez MM, Rossi JS. The process of smoking cessation: an analysis of precontemplation, contemplation, and preparation stages of change. *J Consult Clin Psychol.* 1991;59(2):295-304.
93. Snow MG, Prochaska JO, Rossi JS. Stages of change for smoking cessation among former problem drinkers: a cross-sectional analysis. *J Subst Abuse.* 1992;4(2):107-116.
94. Biener L, Abrams DB. The Contemplation Ladder: validation of a measure of readiness to consider smoking cessation. *Health Psychol.* 1991;10(5):360-365.
95. Wong LE, Hawkins JE, Murrell KL. Where are all the patients? Addressing Covid-19 fear to encourage sick patients to seek emergency care. *NEJM Catalyst Innovations in Care Delivery.* 2020.
96. Toolkit P. COVID-19 Community Response Survey Guidance. In:2020.
97. (C3PNO) CCoCPNO. COVID-19 Module for NIDA-Supported Cohorts Affected by HIV and Substance Use. In:2020.
98. Bush K, Kivlahan DR, McDonell MB, Fihn SD, Bradley KA. The AUDIT alcohol consumption questions (AUDIT-C): an effective brief screening test for problem drinking. *Archives of internal medicine.* 1998;158(16):1789-1795.
99. Bradley KA, Bush KR, Epler AJ, et al. Two brief alcohol-screening tests From the Alcohol Use Disorders Identification Test (AUDIT): validation in a female Veterans Affairs patient population. *Archives of internal medicine.* 2003;163(7):821-829.
100. Skinner HA. The drug abuse screening test. *Addictive behaviors.* 1982;7(4):363-371.
101. Yudko E, Lozhkina O, Fouts A. A comprehensive review of the psychometric properties of the Drug Abuse Screening Test. *Journal of substance abuse treatment.* 2007;32(2):189-198.
102. Kessler RC, Andrews G, Colpe LJ, et al. Short screening scales to monitor population prevalences and trends in non-specific psychological distress. *Psychological medicine.* 2002;32(6):959-976.
103. Kessler RC, Barker PR, Colpe LJ, et al. Screening for serious mental illness in the general population. *Arch Gen Psychiatry.* 2003;60:184-189.
104. American College of Radiology. Lung-RADS Version 1.0 Assessment Categories. Available at http://www.acr.org/Quality-Safety/Resources/LungRADS. In:April 28, 2014.
105. Elo S, Kyngas H. The qualitative content analysis process. *J Adv Nurs.* 2008;62(1):107-115.
106. Creswell JW, Plano Clark VL. Chapter 3: Choosing a Mixed Methods Design. In: *Designing and Conducting Mixed Methods Research.* 2nd ed. Los Angeles: Sage; 2011:53-106.
107. Health Resources and Services Administration, U.S. Department of Health & Human Services. 2015 Health Center Data: National Health Care for the Homeless Program Grantee Data. https://bphc.hrsa.gov/uds/datacenter.aspx?year=2015&fd=ho. Accessed March 16, 2017.
108. Center for Tobacco Treatment Research & Training, University of Massachusetts Medical School. Basic Skills for Working with Smokers - Online Course. http://www.umassmed.edu/tobacco/training/basicskills_online/. Accessed March 28, 2017.

TITLE: Lung cancer screening navigation for homeless people: A pragmatic trial

PRINCIPAL INVESTIGATOR: Travis P. Baggett, MD, MPH

GRANT NUMBER: 131162-RSG-17-157-01-CPPB (American Cancer Society)

VERSION DATE: December 16, 2020

I. BACKGROUND AND SIGNIFICANCE

A. Historical background

An estimated 2.3-3.5 million people experience homelessness annually in the US.[1] Lung cancer incidence and mortality are substantially higher in this vulnerable population than in comparably-aged non-homeless individuals.[2-4] To address this disparity, we propose a pragmatic clinical trial to test the effect of patient navigation on lung cancer screening (LCS) completion among homeless-experienced adults at increased risk for lung cancer. Work that we and others have done suggests 2 reasons why such an intervention is needed.

1) Lung cancer is a major cause of death among homeless adults and the leading cause of tobacco-attributable death in this population.

In our study of 28,033 adults who used Boston Health Care for the Homeless Program (BHCHP) services in 2003-08, cancer was the second-leading cause of death and the leading killer of adults ≥45 years old.[5] Lung cancer accounted for one-third of these cancer deaths.[5] In a subsequent study of cancer epidemiology in this cohort, lung cancer was the leading type of incident cancer and the leading cause of cancer death among both men and women, with age-standardized incidence and mortality rates exceeding those in the Massachusetts adult population more than 2-fold.[2] An estimated 88% of incident lung cancer cases[2] and 93% of lung cancer deaths[6] were attributable to tobacco smoking, making lung cancer the leading type of smoking-attributable cancer and the leading cause of smoking-attributable death among homeless people. The excess burden of lung cancer in these studies of homeless adults in Boston was similar to that observed in studies of homeless and marginally housed people in Canada[4] and Glasgow[3] (Table 1), suggesting that these disparities are not geographically constrained. This body of evidence highlights the need for interventions to reduce lung cancer disparities in homeless populations. One avenue for doing so is LCS with low-dose computed tomography (LDCT) to promote detection and potentially curative treatment of early-stage lung cancer. This strategy was associated with a 20% reduction in lung cancer mortality in the National Lung Screening Trial (NLST).[7] However, implementing such a strategy presents formidable hurdles in the setting of homelessness.

Table 1. Lung cancer among homeless adults

Study location (yrs)	SIR/SMR (95% CI)
Boston (2003-2008)	
Incidence, men	2.30 (1.84, 2.84)
Incidence, women	2.23 (1.41, 3.35)
Mortality, men	2.39 (1.83, 3.08)
Mortality, women	2.31 (1.26, 3.88)
Canada (1991-2001)	
Mortality, men	1.91 (1.67, 2.18)
Mortality, women	1.73 (1.26, 2.36)
Glasgow (1975-1993)	
Incidence, men	1.64 (1.41, 1.86)

2) Homeless people have suboptimal rates of cancer screening and are diagnosed with screen-detectable malignancies at later stages than non-homeless people; barriers to implementing LCS in the setting of homelessness could exacerbate existing disparities in lung cancer outcomes.

In the 2003-08 BHCHP study of cancer epidemiology described above, homeless individuals were diagnosed with colorectal cancer and female breast cancer at significantly later stages than adults in the Massachusetts general population.[2] Several studies have documented low rates of screening for these malignancies among homeless individuals.[8-12] Given the high prevalence of smoking among homeless people[8,13-18] and the aging of the homeless population,[19] many homeless individuals are expected to meet the recommended criteria for consideration of LCS with LDCT[20,21]; however, as with colorectal and breast cancer screening, there are several obstacles to successfully implementing LCS in homeless populations. Homeless individuals struggling to meet basic subsistence needs may place a low priority on screening for asymptomatic illness[22] or may feel too emotionally overwhelmed to cope with the possibility of an abnormal result. Additionally, despite a high burden of cancer risk factors, most homeless people appear to believe that their susceptibility to cancer is equal to or less than that of others the same age.[8] Lack of insurance may pose another barrier,[23,24] although this is less of a concern in Massachusetts and could improve elsewhere in states that expanded Medicaid under the Affordable Care Act. Even among insured individuals who are interested in obtaining LDCT screening, the process of scheduling and attending the test may prove logistically challenging because of difficulty navigating complex facilities and care systems,[25] especially for the large proportions of homeless individuals with psychiatric and cognitive impairments.[26-32] Furthermore, ensuring timely follow-up of abnormal results may be challenging because of communication barriers and competing psychosocial issues. These barriers introduce the possibility that existing disparities in lung cancer mortality could worsen if homeless people are less able to complete LCS than other segments of the population.

B. Preliminary data

Our prior epidemiologic work demonstrating the burden of lung cancer disparities among homeless people is described above and provides the motivation for the proposed study. In addition, this study is informed by administrative data from BHCHP, survey and clinical trial studies with homeless smokers conducted by the PI (Dr. Baggett), navigation studies conducted by co-investigator Dr. Percac-Lima, and LCS-oriented studies conducted by co-investigators Dr. Park and Dr. Rigotti. Collectively, this preliminary data has demonstrated the following:

1) We have the patient volume to support this study. Based on internal data from the BHCHP Institute, 3,443 unique individuals aged 55-77 years old were seen at any BHCHP site from 9/1/2015 to 9/1/2016. Of these, 53% were current smokers and 23% were former smokers, totaling 2,616 potentially eligible individuals seen by the program in a 12-month period. When extrapolated over the planned 2.5-year enrollment period, we anticipate being able to recruit and randomize the proposed number of 300 participants.

2) We have characterized the target population. In April-July 2014, we used time-location sampling to conduct a survey of 306 homeless adult smokers at 5 high-volume BHCHP clinical sites.[33-35] The response rate was 86%. One-quarter of respondents (N=78) were 55-77 years old. Of these, the vast majority were non-Hispanic white or black men with a high school degree. Seventy percent had ever experienced a traumatic head injury, 64% had used any drug in the past month, 39% had consumed alcohol to intoxication in the past month, 60% reported feeling seriously depressed, and half screened positive for PTSD. One-third reported COPD and 17% reported cardiovascular disease. Past-month difficulty finding shelter, food, and clothing were common. The mean number of cigarettes per day was 12 and the mean Fagerstrom nicotine dependence score was 3.8 (range 0-12). These findings highlight the medical complexity and competing life issues of older homeless smokers and underscore the need for a navigation-based intervention to reduce the barriers to LCS.

3) We can recruit and longitudinally retain homeless people in a clinical trial. From October 2015 to June 2016, we conducted a 3-arm (N=25 per arm) pilot randomized controlled trial testing the effect of a) contingent financial rewards for smoking abstinence, and b) text messaging to support smoking abstinence, against c) a shared control condition, among homeless smokers at BHCHP (NCT02565381). Sixty-eight percent of eligible individuals enrolled, and we reached our target enrollment within 6 months. Study attendance and retention rates were excellent. Of 14 possible assessment visits, participants attended a median of 10, with 97% attending ≥1 visit and 77% attending ≥7 visits. These findings demonstrate that our team has the expertise to conduct a behavioral treatment trial in the context of homelessness and to engage and retain this vulnerable population in a longitudinal fashion. This is facilitated by our extensive knowledge of the target population, our person-centered approach to participant engagement, our strong partnership with BHCHP, and the central location of BHCHP headquarters with respect to other homeless services.

4) Our navigation intervention is based on a model that has proven effective at improving lung and other cancer screening among underserved patient populations in Boston. Key collaborator, Dr. Sanja Percac-Lima, has developed and tested navigation interventions that have been associated with improved cancer screening rates among diverse, low-income community health center patients in Boston.[36,37] She conducted a randomized controlled trial (RCT) across 18 clinical practices in an academic primary care network evaluating the impact of patient navigation relative to usual care for high-risk individuals who were overdue for breast, colorectal, and/or cervical cancer screening.[38] Patients assigned to navigation were significantly more likely to complete screening for breast (23.4% vs. 16.6%, $p=0.009$), cervical (14.4% vs. 8.6%, $p=0.007$), and colorectal (13.7% vs. 7.0%, $p<0.001$) cancer during the follow-up period.[38] More recently, with grant support from ACS and in collaboration with Drs. Park and Rigotti, Dr. Percac-Lima was the principal investigator of an RCT testing the effect of patient navigation on chest CT completion among low-income patients eligible for LCS at 5 MGH-affiliated health centers in greater Boston. Preliminary analysis of data after 11 months of follow-up has shown that participants in the navigation arm were significantly more likely to obtain a screening lung CT than participants assigned to usual care (24% vs. 9%, $p<0.01$). The LCS navigation approach used in our study will be based on these successful models.

C. Study rationale

Mitigating disparities in lung cancer outcomes and fully realizing the potential benefits of LCS among homeless people will require thoughtful interventions deployed through the clinical programs that serve this vulnerable population. Patient navigation, a strategy for guiding individuals through complex health systems,[39] may be a promising approach for helping homeless people overcome their unique barriers to LCS.[40] Navigation interventions have been shown to improve cancer screening participation[36,37,41-56] and diagnostic resolution of abnormal results[57-63] in vulnerable populations. Patient navigation has been endorsed by the ACS[64] and the National Cancer Institute[65] as a valuable approach to reducing cancer health disparities,[65] but this approach has not been tested rigorously in a homeless health care setting. Our research group is well-poised to conduct such a study by leveraging our extensive clinical and research experience with this population and our 9-year partnership with BHCHP, an internationally-renowned organization that serves over 12,000 currently (84%) and formerly (16%) homeless people annually through dozens of clinical sites in greater Boston.[66] This innovative community partnership has given rise to numerous impactful studies on the health of homeless-experienced people,[2,5,6,33-35,67] including a recently pilot trial for homeless smokers that had brisk recruitment, high retention rates, and encouraging results (NCT02565381).

II. SPECIFIC AIMS

Aim 1: To determine the effect of patient navigation, added to usual care, on 1°) LCS LDCT completion at 6 months and 2°) LCS LDCT completion at 6 months and diagnostic follow-up of abnormal results within 1 month of the recommended timeframe, among homeless-experienced people who are eligible for LCS.

Hypothesis: Participants assigned to receive patient navigation will have significantly greater 1°) LCS LDCT completion at 6 months and 2°) LCS LDCT completion at 6 months with timely diagnostic follow-up of abnormal results.

Aim 2. To conduct post-intervention qualitative interviews of trial participants and BHCHP PCPs to explain and interpret the quantitative findings of the pragmatic clinical trial.

Based on a sequential explanatory mixed methods approach, Aim 2 interviews will examine how and why participants decided whether to undergo LCS, the barriers they encountered in doing so, the ways in which the navigator helped them overcome these barriers, and opportunities for improving the intervention for future use.

III. SUBJECT SELECTION

A. Inclusion and exclusion criteria

To be eligible to participate, individuals must meet **all** the following criteria:

1) Aged 55-77 years old, assessed by self-report and verified by date of birth.
   Rationale: LCS LCDT is covered only for patients aged 55-77.
2) Have a 30 pack-year smoking history *and* have smoked within the past 15 years.
   Rationale: We will be recruiting exclusively smokers because they are at the highest risk for lung cancer and therefore stand to receive the most benefit from a LCS. These smoking history parameters are consistent with current practice guidelines and Medicare eligibility criteria.
3) Have a BHCHP primary care provider (PCP).
   Rationale: Patient navigation is designed to support and extend services provided by medical practitioners. Having a PCP will help to ensure longitudinal medical oversight of the LCS process and appropriate follow-up of abnormal results.
4) Are currently or formerly homeless, assessed by self-report and defined as ever having experienced a time in their life where they did not have fixed, regular housing.
   Rationale: Both currently and formerly homeless individuals were included in our epidemiologic analysis showing a more than 2-fold higher incidence and mortality rate from lung cancer compared to the general Massachusetts adult population.[2] BHCHP and most HCH programs nationally continue to serve patients after they have gained housing.
5) Be proficient in English, assessed with items asking about native language and self-reported comfort communicating in English among non-native speakers.
   Rationale: Because of a budget reduction by the funding agency, we do not have the resources to develop study materials or conduct in-person navigation in languages other than English. Additionally, the vast majority of individuals in the target population speak English.

Individuals meeting **any** of the following criteria will be excluded from the study:

1) A recent prior chest computed tomography (CT) imaging (in the past 12 months).
   Rationale: This study is aimed at individuals who are most likely to benefit from LCS, based on evidence from the National Lung Screening Trial. Potential participants that have recently received a chest CT will have recently been scanned for lung cancer, therefore an additional screening will not benefit their health outcomes.
2) Any personal history of lung cancer, or current presentation with symptoms concerning for lung cancer (e.g. hemoptysis or unexplained weight loss of >15 lbs. in the past year).
   Rationale: Individuals with a history of lung cancer or symptoms concerning for lung cancer require surveillance or diagnostic lung imaging, respectively, and are not appropriate candidates for screening lung imaging, which by definition is designed for individuals without symptoms or history of the illness being screened for.
3) Inability to provide informed consent, assessed with knowledge questions about the material presented during the informed consent process that individuals must correctly answer before providing informed consent to participate.
   Rationale: In this vulnerable population, we wish to take a conservative approach to the informed consent process to help ensure that participants fully understand the pros and cons of participating in the research study.
4) PCP is the study PI (Dr. Travis Baggett).
   Rationale: BHCHP patients under the primary care of Dr. Baggett may feel unduly influenced to participate. In addition, since the primary study outcome (receipt of LDCT for LCS) requires PCP involvement, the inclusion of Dr. Baggett's patients creates a conflict of interest and the potential for bias.

B. Source of subjects and recruitment methods

This study will be registered with ClinicalTrials.gov prior to the recruitment and enrollment of human subjects. All participants will be recruited from BHCHP clinical sites. BHCHP does not have its own IRB but instead will rely on the Partners Human Research Committee for IRB review through a reliance agreement initiated through SMART IRB. In addition to being a faculty physician-investigator at Massachusetts General Hospital and Harvard Medical School, the study PI is the Director of Research at BHCHP and is very familiar with the clinical environment and patient population served by the program.

Individuals must be homeless to enroll in services at BHCHP; however, some patients continue receiving care at the program after they are no longer homeless. An internal analysis of individuals seen at BHCHP in 2011 found that 16% of patients were housed, most without supportive services and therefore at potentially high risk for recurrent homelessness. Because our epidemiologic analyses demonstrating the dramatic lung cancer disparities experienced by BHCHP patients included both currently and formerly homeless individuals, and because of the often cyclic nature of homelessness, we will include both currently and formerly homeless individuals in the proposed study. We refer to this group collectively as "homeless-experienced." Owing to the Massachusetts system of universal health insurance, about 80% of BHCHP patients are insured by the state Medicaid program,[68] which covers LCS LDCT among eligible beneficiaries,[69] and a sizable proportion of those remaining are covered by Medicare alone. This will help ensure the availability of LCS LDCT for the vast majority of potential participants.

Participants will be recruited through a combination of 3 methods:

1) In-person screening of patients at BHCHP clinical sites: Study personnel will screen patients in-person at 6 high-volume BHCHP clinic sites: a) Pine Street Inn, a 470-bed shelter where BHCHP operates a medical clinic 7 days/week in the men's shelter and 6 days/week in the women's shelter; b) Southampton Shelter, a 400-bed men's shelter where BHCHP operates a medical clinic 7 days/week; c) Woods Mullen Shelter, a 200-bed women's shelter where BHCHP operates a medical clinic 5 days/week; d) New England Center and Home for Veterans, a 209-bed emergency and transitional shelter where BHCHP operates a medical clinic 5 days/week; e) St. Francis House, a daytime multiservice facility for homeless people where BHCHP operates a medical clinic 5 days/week; and f) Jean Yawkey Place, the BHCHP headquarters facility where medical, behavioral health, and dental clinics operate 5 days/week with an on-site pharmacy and medical respite unit. Our prior survey fieldwork with this population has given us extensive experience with tactfully deploying this proactive, in-person recruitment technique.[33-35] In light of the COVID-19 pandemic, BHCHP clinical sites and shelters are experiencing a significant decrease in patient volume and a lack of additional space to safely conduct in-person recruitment and data collection activities. Under these circumstances, study personnel will initially focus in-person screening and data collection efforts at Jean Yawkey Place, which has the highest volume of patients and the greatest degree of flexibility in physical space and layout. This will allow the study personnel to have an in-person presence at the clinic while practicing physical distancing and prioritizing the health and well-being of participants and study staff. If COVID-19 restrictions loosen and patient volume increases at shelter sites, we will expand our efforts to include the five additional sites, depending on COVID-19 public health guidelines and facility protocols.

2) Review of BHCHP electronic records: BHCHP has a comprehensive EHR system (Epic) that prompts providers to assess tobacco use at every clinical encounter, facilitating determination of smoking history and current smoking status. Individuals with a BHCHP PCP who have been seen in a BHCHP clinic in the past year and who were current or former smokers at the time of their most recent clinical encounter will be approached in person at BHCHP clinical sites if they have a scheduled appointment or contacted proactively via phone (if they have listed contact information) by a research coordinator and screened for eligibility to participate. The BHCHP Chief Medical Officer has approved this recruitment strategy on behalf of all primary care providers at BHCHP clinical sites, who will in turn be notified about our recruitment approach prior to the start of the study (letter of approval in Appendix J).

3) Referrals from BHCHP providers: We will advertise the study to all BHCHP providers and in all BHCHP clinical sites. Individuals deemed by a BHCHP provider to be potential candidates for LCS can be referred to the research coordinator, who will screen referred patients for eligibility to participate.

IV. SUBJECT ENROLLMENT

An integrated study schema depicting recruitment, enrollment, treatment assignment, and outcomes assessment has been uploaded as an attachment to this application.

A. COVID-19 symptom and exposure screening

BHCHP staff currently screen all patients for COVID-19 symptoms or a recent positive COVID-19 test upon entry to BHCHP clinics and facilities. BHCHP is also providing patients with facemasks before entering the building. Study personnel will re-screen participants using the same approach before conducting any research activities in an enclosed room. If BHCHP ceases their COVID-19 screening procedure, study personnel will use BHCHP's "COVID-19

Screening Tool for People Experiencing Homelessness" to screen all participants for current COVID-19 symptoms and past 14-day COVID-19 exposures (i.e., travel to high risk countries or states; unmasked exposure to a person with COVID-19). Study staff will wear appropriate personal protective equipment (PPE) per MGH guidelines and policies and remain 6 feet away from the patient. If patient endorses any COVID-19 symptoms or high-risk exposures, study personnel will immediately notify a clinic staff member of the presence of a symptomatic patient and will not engage in any study activities. Immediately after departure of the symptomatic patient, study personnel will disinfect surfaces that were within 6 feet of the symptomatic patient. If the participant reports not having symptoms, they can be directed to the study table/interview room. If the study coordinator makes initial contact with a participant over the phone, before scheduling an in-person visit, study personnel will screen the participant within 72 hours before the appointment. If participants report COVID-19 symptoms or exposures, study staff will request participants' permission to contact their BHCHP PCP via Epic messaging who can then advise the participant on next steps (e.g., testing and/or quarantine). If participants do not provide permission to study staff to contact their PCP, study staff will recommend that the participant seek care for their symptoms and/or exposure and offer to do the initial study procedures over the phone. If they are not interested in completing any further study procedures over the phone, study staff will call the participant again in 10 days to check-in and rescreen before attempting to schedule any study procedures in person. Participants will be advised to put on a facemask, regardless of symptoms, before coming in to meet with the research coordinator. Research personnel will instruct participants to notify them before arriving if they have fever or symptoms of COVID-19.

B. Methods of enrollment

Participants recruited through the above methods (section III.B) will be screened for eligibility either in-person or by phone. Prior to contacting participants, study staff will use the eligibility screener (Appendix A) to record information related to eligibility that was able to be collected during the prior review of prospective participants' BHCHP EHR (as referenced above).

Upon approaching the participants, study staff will use participant self-report to verify any information collected via their EHR and will collect the minimum amount of additional information necessary to assess and verify study eligibility. Eligible individuals identified through in-person screening will be offered the chance to enroll immediately. Those identified through phone screening will be asked to attend an in-person enrollment visit at one of clinic sites listed above. Participants will be asked to provide verbal informed consent before enrolling.

At least six months after initial enrollment, participants who were randomized to the patient navigation arm of the study will be contacted via phone or approached in person at their next BHCHP appointment to be offered the opportunity to participate in a 45-minute qualitative interview. Forty participants will be interviewed in total, with purposive sampling of participants who did (N=20) and participants who did not (N=20) attain the primary outcome of completing a lung screening CT scan within six months.

At approximately 6 months after the last patient participant is enrolled, providers who had at least one patient enrolled in the trial will be contacted via phone, email, or in person to be offered the opportunity to participate in a 45-minute qualitative interview. Ten participants will be interviewed in total, with purposive sampling to ensure that at least one provider who works at each of the targeted BHCHP clinic sites are interviewed.

C. Informed consent procedures

We will obtain verbal informed consent for this study. Eligible participants will be read a verbal consent script (Appendix B) and be presented with a study fact sheet (Appendix C) that will reinforce the consent script information. Depending on space availability, participant and research coordinator preferences, and whether in-person research activities are safe and permissible, informed consent will be obtained in one of the following ways: 1) "telephone booth" approach where the research coordinator would conduct the consent via Microsoft Teams or Enterprise Zoom on an encrypted laptop with the participant in a dedicated space on an encrypted study tablet; 2) meet in the clinic lobby, in a screened off area while maintaining at least 6 feet distance; 3) meet in an enclosed BHCHP clinic room while maintaining at least 6 feet distance if safe to do so; or 4) over the phone. The research coordinator will confirm participants' understanding of the consent materials with basic knowledge questions about the information presented to them to ensure that they have the capacity to provide informed consent. Individuals who are able to correctly answer these questions will be asked to give their verbal consent to participant, which will be recorded by the research coordinator. Participants who provide informed consent over the phone will be given the option to receive a copy of the study fact sheet via picking up in-person at the JYP clinic, via mail if the participant has a mailing address, via secure email, or via email without send secure after obtaining participant's permission.

40 participants who agree to participate in the post-intervention qualitative interviews will participate in a second verbal informed consent process at least 6 months after their initial enrollment. The research coordinator will read a separate verbal consent script (Appendix G) that reinforces the information presented in a fact sheet (Appendix F) that will be distributed to participants. The fact sheet will focus on the purpose, requirements, risks, and benefits of participating in the qualitative interview. The research coordinator will confirm participants' understanding of the consent materials with basic knowledge questions about the information presented to them to ensure that they have the capacity to provide informed consent. Individuals who are able to correctly answer these questions will be asked to give their verbal consent to participant, which will be recorded by the research coordinator.

Additionally, we will obtain verbal consent to participate in qualitative interviews from 10 BHCHP PCPs who had at least 1 patient enrolled in the trial. Similar to the participants who participate in the interviews, the providers will be read a verbal consent script (Appendix I) that coincides with a fact sheet (Appendix H) that will be distributed to them. The research coordinator will record the providers' verbal consent to participate in the interview.

We believe the verbal consent is justified for several reasons:

1) The experimental intervention, patient navigation, is a low-risk and widely-recommended behavioral intervention strategy with a sound evidence base in promoting cancer screening in vulnerable populations. Indeed, patient navigation has been endorsed by the ACS[64] and the National Cancer Institute[65] as a valuable approach to reducing cancer health disparities,[65] and is now part of routine care in some practice settings, including at MGH-affiliated health centers.

2) The outcome that the navigator is facilitating – obtaining LDCT for LCS among individuals at increased risk for lung cancer – is concordant with the guideline recommendations of the USPSTF,[20] ACS,[70] and multiple other professional groups.[71-74]

3) The study procedures present no more than minimal risk, as outlined below in section VII.

In order to ascertain the primary and secondary study outcomes of LDCT completion and LDCT completion with timely diagnostic follow-up (see section VII.B.), we will obtain written authorization from all enrolled participants to obtain their health/medical records for the 14 months following their trial enrollment date from the following Boston-area hospitals: Massachusetts General Hospital, Brigham & Women's Hospital, Boston Medical Center, Cambridge Health Alliance hospitals, and Tufts / New England Medical Center, in addition to any other hospital that the participant identifies as being one they typically use. Written authorization will be obtained after participants complete verbal informed consent using a HIPAA-compliant Release of Information (ROI) form which has been vetted by privacy officers at BHCHP, Boston Medical Center, and MGH (Appendix N). Participants who provide verbal consent during an in-person enrollment appointment will also complete the ROI form in person. Participants with a stable home address who complete the enrollment appointment remotely will be given the option to receive a pre-stamped envelope with the ROI form to have them sign and return back by mail to our MGH or BHCHP office.

If COVID-19 prompts further restrictions around in-person research activities at BHCHP, the research coordinator will transition to completing the verbal consent process over the phone or via the HIPAA compliant video conferencing platforms, Microsoft Teams or Enterprise Zoom depending on each participant's preference and access to technology. Based on feedback from the Boston Medical Center Compliance and Privacy Office, obtaining participant medical records for research purposes will continue to require written authorization regardless of current restrictions. Therefore, as noted previously, the research coordinator will offer participants with a stable home address the option to receive a pre-stamped envelope with the ROI form to sign and mail back. For participants who cannot receive a mailed ROI form, we will need to wait until in-person research restrictions are lifted. In this circumstance, once safe to do so, a research coordinator will either directly contact these research participants by phone or attempt to meet them in person at a clinic site to complete the form in person.

D. Treatment assignment

After providing informed consent and completing a baseline survey (see section V. A.), participants will be block-randomized in a 2:1 ratio to usual care with (N=200) or without (N=100) LCS navigation. The randomization scheme will be concealed from the study coordinator responsible for randomizing participants. Randomization will be stratified by smoking status (current/former), housing status (homeless/housed), prior discussion of lung cancer screening with their PCP, and primary clinical site where the patient sees their PCP (JYP vs. non-JYP sites) to ensure balance between study arms on these variables. We will stratify by smoking status (determined based on responses to the eligibility screener) because lung cancer risk perceptions and LCS motivations may differ between current and former smokers.[75,76] Current smoking will be defined as having smoked all or part of a cigarette in the past 30 days.[77] We will stratify by housing status based on responses to various items within the baseline survey because the navigation needs of formerly homeless people in housing may differ from those who are currently homeless. Current homelessness will be defined in the following ways: a) usually staying in an emergency shelter, transitional shelter, abandoned building, place of business, car or other vehicle, church or mission, hotel or motel, or anywhere outside during the past 7 days, b) usually housed in last 7 days but cannot stay in a residence for at least 14 days, c) usually institutionalized in the last 7 days if the admission was less than 90 days and they were homeless prior to admission, or d) usually institutionalized in the last 7 days if the admission was more than 90 days and they have no identified residence where they can stay at least 14 days upon discharge. These definitions are concordant with definitions of

homelessness used by the US Department of Health and Human Services (section 330 of the Public Health Service Act, 42 USC § 254b)[78] and by the US Department of Housing and Urban Development (S. 896 HEARTH Act of 2009).[79] We will stratify based on prior discussion of lung cancer screening with their PCP (defined by an item within the baseline survey) due to the fact that these participants are farther along in the lung cancer screening process. Finally, we will stratify by the patient's primary care clinical site since clinics may differ in their proximity to facilities that offer LCS or in their practice patterns around recommending and ordering LDCT for LCS. We will create 2 clinical site strata: 1 for JYP and one encompassing all of the 5 non-JYP study sites described above.

The 2 study arms are as follows:

Arm 1: Usual care ***without*** patient navigation (N=100). Participants assigned to this arm will be given basic educational materials on general lung health (Appendix K) and referred back to their PCP to discuss whether to pursue lung cancer screening per usual practice.

Arm 2: Usual care ***with*** patient navigation (N=200). Participants assigned to this arm will be informed about LCS, provided educational materials on LCS and patient navigation (Appendix L), and offered access to an LCS navigator. The navigator's principal role is to guide participants through the LCS process. The navigator's secondary role is to provide a brief tobacco intervention for participants who currently smoke. These roles are described below:

1) LCS navigation: The navigator will introduce the concept of LCS and assist participants in scheduling and attending a shared decision-making (SDM) visit with their PCP to discuss the test in greater depth. The navigator will proactively reach out to PCPs about this via electronic messaging through the BHCHP EHR and make him/herself available to assist in the LCS process. If a PCP and a participant jointly decide to pursue LCS, the navigator will assist with booking the LDCT appointment at a local facility that offers this test and interprets it according to the Lung-RADS framework.[80] BHCHP maintains strong clinical ties with Massachusetts General Hospital, Boston Medical Center, and several other area hospitals, enhancing the feasibility of referring patients to these facilities for LDCT. The navigator will work collaboratively with participants to reduce the barriers to LCS completion while building participants' self-efficacy. If a participant has difficulty obtaining LDCT because of lack of insurance, the navigator will connect the participant with a BHCHP benefits specialist to enroll in health insurance under the state's universal coverage system. Following LDCT, the navigator will coordinate a follow-up appointment with a participant's PCP to discuss the results and arrange any necessary follow-up testing. Throughout this process, the navigator will provide reminder phone calls about upcoming appointments, arrange for transportation assistance and other enabling services when needed, and reinforce the content and concepts covered in the LCS SDM visit through in-person or phone contact. Data from our survey of homeless smokers at BHCHP indicate that over 70% have mobile phones, which will facilitate communication between the navigator and study participants. In addition to documenting their encounters and activities with patients for research data collection purposes (see section VI.A.), the navigator will document within the patients' BHCHP EHR for their care teams to reference as needed.

The navigator will work with each participant for up to 6 months to receive the initial LCS LDCT scan. If a participant receives their initial LCS LDCT scan within the 6-month timeframe and requires follow-up diagnostic testing of abnormal results anytime sooner than the usual LCS LDCT screening interval of 12 months, the navigator will continue to work with the participant until the follow-up diagnostic testing is completed or until the participant no longer wishes to be navigated.

2) Brief tobacco intervention: The navigator will provide a brief tobacco intervention and distribute educational handouts on smoking cessation (on an ad hoc basis; Appendix M) consistent with the "5 A's" approach.[81] Within this framework, the navigator will Ask participants if they currently smoke cigarettes and Advise current smokers to quit, emphasizing the connection between smoking and lung cancer and the importance of quitting smoking to lower their lung cancer risk. The navigator will then Assess current smokers' willingness to quit and Assist them in accessing no-cost or low-cost smoking cessation resources, including:

a) BHCHP PCPs, to discuss smoking cessation medications (e.g. nicotine replacement products, bupropion, or varenicline), which are covered by the state Medicaid program with a $1-3 copayment at retail pharmacies or at no charge through the BHCHP pharmacy. Over 80% of BHCHP patients are insured by the Massachusetts Medicaid program under the state's universal health insurance system.[68]

b) BHCHP tobacco counselors, who are formally trained in tobacco counseling and conduct weekly support groups at 4 BHCHP venues and individual counseling sessions at 5 BHCHP venues, all free of charge.

c) The Massachusetts Smokers' Helpline, a free and confidential phone counseling service, for participants with mobile phones. Participants who call the Massachusetts Smokers' Helpline may receive up to 4 weeks of nicotine replacement therapy (patch, gum, or mini-lozenge) at no cost.

The navigator will then arrange a time to follow-up with current smokers either by phone or in person to discuss their progress on smoking and their engagement in available services. For participants who undergo LDCT, the navigator will conduct a follow-up brief intervention to head off false reassurance about smoking if the findings are negative. Brief tobacco interventions are an evidence-based, effective treatment approach to promoting smoking cessation.[81,82] The brief intervention strategy described above acknowledges the centrality of smoking cessation in reducing lung cancer deaths, capitalizes on the "teachable moment" afforded by the LCS process,[83] satisfies the CMS requirements for LCS LDCT approval,[21] and complements our pragmatic trial design by working within existing tobacco cessation resources available to the target population.

V. Study Procedures

A. Study visits and measurements

Participants who are screened and deemed eligible to participate, whether in person or over the phone, will be asked to complete a research visit during which enrollment, collection of baseline data, and randomization to study arm will occur. Participants will meet with the research coordinator in person at a BHCHP clinic site to complete these activities or complete these activities remotely. After verbal consent and the written ROI form is completed, a study coordinator will meet with the participant to administer a 30-minute baseline survey. In a similar fashion to the verbal consent, the survey can be completed in one of the following ways depending on current pandemic status and/or participant/staff preferences: a) "telephone booth" approach where the research coordinator would conduct the survey on an encrypted laptop via Microsoft Teams or Enterprise Zoom with the participant in a dedicated space with an encrypted study tablet; b) meet in the clinic lobby in a screened off area while maintaining at least 6 feet distance; c) meet in an enclosed BHCHP clinic room while maintaining at least 6 feet distance of safe to do so; or d) remotely, either over the phone if the participant has adequate access to

their own phone and phone plan, or over secure video conferencing (Microsoft Teams or Enterprise Zoom) if the participant has adequate access to the necessary technology.

Participants will receive a one-time $25 gift card upon completion of the baseline survey in recognition of their time and effort. If participants complete the baseline survey over the phone, research staff will offer participants the chance to pick up the study gift card in-person at JYP or via mail if the participant has a mailing address.

Participants with a stable home address who complete enrollment and the baseline survey over the phone and who are not willing to travel to JYP to sign the ROI in person, will be given the option to receive a pre-stamped envelope with the ROI form to have them sign and mail back to our MGH or BHCHP office. Participants will be mailed the $25 gift card once the research coordinator receives a signed ROI.

Arm 2 participants will additionally have a series of ad hoc meetings with the LCS navigator as part of the intervention. These meetings may occur in person or over the phone. The navigator will be asked to log the date, time, and nature of activities carried out during these navigation sessions/meetings.

Due to the nature of the primary outcome, which will be ascertained via medical record review, no formal research follow-up visit is necessary. The screening, baseline, and outcome variables for the study are detailed in section VI.A.

40 participants in Arm 1 will participate in post-intervention interviews and be stratified according to whether they did (N=20) or did not (N=20) attain the primary outcome of completing a lung screening CAT scan within six months of initial enrollment. Participants who agree to participate will go through a separate verbal consent process as illustrated previously. Interviews will occur after at least 6 months of exposure to the navigation intervention. The interviews will last up to 45 minutes and be audio recorded. The selection of qualitative participants following ascertainment of the primary quantitative outcome avoids introducing a treatment effect from the interviews while remaining temporally close enough to the LCS process to facilitate accurate recall. These 40 participants will receive a one-time $25 gift card upon completion of the interview in recognition of their time and effort.

Additionally, we will interview 10 BHCHP PCPs among those who had at least 1 patient enrolled in the trial. Providers who agree to participate will provide verbal consent as illustrated previously. Interviews will last up to 45 minutes and be audio recorded. Interviews will occur following completion of the trial (at least 6 months after the last patient participant is enrolled) to avoid influencing provider practice around LCS ordering and follow-up. The 10 PCPs will receive a one-time $25 gift card upon completion of the interview in recognition of their time and effort.

Study personnel will follow various safety precautions to prevent COVID-19 transmission. For patients that are screened for eligibility over the phone and interested in scheduling an in-person study visit, study personnel will administer a screening instrument for COVID-19 symptoms and recent positive COVID-19 tests within 72 hours prior to the scheduled in-person study visit. Prior to entering BHCHP clinic buildings, per BHCHP policy, all patients are already required to wear a face mask and be screened for COVID-19 symptoms and recent positive COVID-19 tests at the door. If BHCHP ceases these screening procedures, study personnel will implement the same screening measures at the start of any in-person visit and will continue to require participants to wear a face mask while engaging with study staff. If a patient endorses

any COVID-19 symptoms, study personnel will immediately cease study activities and notify a clinic staff of the presence of a symptomatic patient.

If rising COVID-19 rates necessitate temporary restrictions on all in-person study activities at BHCHP facilities, we will transition to remotely conducting as many study activities as possible until the restrictions are lifted. Study personnel will be able to screen, perform verbal informed consent, conduct the baseline survey, perform qualitative interviews, and provide navigation services over the phone or by the HIPAA-compliant video conferencing platforms Microsoft Teams or Enterprise Zoom depending on patient preference and access to technology (i.e., owning a device with adequate minutes or video conferencing capabilities). Study personnel will always follow the MGB requirements for using Teams or Zoom with no study visits or activities being audio- or video-recorded within the Teams or Zoom platform. Microsoft Teams will be the preferred platform to use for video call study visits, but in the event of technical difficulties, study personnel will use Enterprise Zoom as a backup platform.

B. Drugs

While the LCS navigator will assist participants who currently smoke with accessing smoking cessation resources, neither the navigator nor study staff will provide participants with medications. Participants in both arms may choose to seek smoking cessation medications through their BHCHP PCP or other health care provider.

C. Devices

No devices will be used in this study.

D. Procedures

There are no procedures or surgical interventions in this study. Participants may elect to undergo LCS with LDCT after consultation with their PCP (and with the LCS navigator, for those in Arm 2), but the ordering and oversight of LDCTs will be handled by BHCHP PCPs as per usual practice for participants in both study arms.

E. Data Collection

We will collect all study data electronically using Partners-approved laptops or tablet devices with full disk encryption. Participant data will be recorded, saved, and managed using the Partners-hosted Research Electronic Data Capture (REDCap) program with data collection forms custom-designed for this study. We will also use BHCHP EHR to collect information from participants' medical records that relates to their general health history, any charted mental health problems, and any other information that directly relates to lung cancer screening and smoking. The specific data elements collected at each study visit are detailed below in section VI.A.

VI. BIOSTATISTICAL ANALYSIS

A. Specific data variables

1) Screening variables: Screening variables will include self-reported measures related to the inclusion and exclusion criteria for study participation. These will include the following variables,

assessed as shown in Appendix A:
- First and last name
- Date of birth
- Age
- Preferred language
- BHCHP PCP
- Preferred BHCHP clinic site
- History of homelessness
- History or symptoms of lung cancer
- Recent non-lung cancer or cancer treatment
- Home oxygen use
- Cigarette smoking history

2) Contact form variables: Enrolled participants will be asked to complete a contact information form. This information will be valuable for following up with participants who cannot complete the baseline survey on the same day as enrollment, as well as for the patient navigator to aid with patient outreach and EHR documentation. This form will include the following variables, assessed as shown in Appendix O:
- BHCHP Epic MRN (entered by the research coordinator from patient's EHR)
- Last 4 digits of social security number
- Phone number (if applicable)
- Address/location of usual nighttime residence
- Alternative contact (who may know of participant's whereabouts)
- Contact in the case of an emergency
- Participant photograph (with participant permission) to facilitate subsequent contact with patient navigator

3) Baseline variables: Enrolled participants will be asked to complete a baseline assessment (Appendix D) of variables mapping to core domains of the Health Belief Model in addition to other key factors that could impact one or more dimensions of the Health Belief Model, as displayed in Table 2.

4) BHCHP electronic health record variables: The following information will be collected from the review of patients' BHCHP electronic health records:
- History of care with BHCHP and BHCHP PCP prior to study enrollment
- Comorbid health conditions.
- Documentation of a shared decision making (SDM) appointment with PCP to discuss lung cancer screening.
- Documentation of referrals for lung CT scans.
- Documentation of follow-up appointments with PCP that address results of lung cancer screening.
- Documented smoking status (and any changes in smoking status during study enrollment).
- Documented prescriptions to any smoking cessation medications
- Documented insurance status and insurance type.

Table 2. Baseline measures of study participants corresponding to Health Belief Model

(HBM) domains and other key factors.

HBM domains	Measures
**Sociodemographic factors**	Age; sex; race/ethnicity; education; (all based on items from the 2003 HCH User Survey[23,84] and our prior survey work with homeless individuals[33,34]); homelessness onset, episodes, and duration[1]; current living situation
**Perceived lung cancer susceptibility/severity**	Perceived risk of lung cancer and smoking, perceived severity of lung cancer, worry about lung cancer, (all developed by Dr. Park for NLST)[76,85];
**Cues for LCS**	Prior LCS recommendation by health care provider; prior exposure to LCS brochures, flyers, posters, or advertisements
**Perceived LCS benefits/barriers**	Lung cancer screening knowledge[86]; perceived benefits of quitting smoking[76,85], comfort navigating hospital/health system; health insurance status[23,84]; competing priorities for meeting subsistence needs[22]
**Self-efficacy for LCS**	General self-efficacy scale[87,88]; self-efficacy for completing LDCT
**Other key factors**	**Measures**
**Tobacco use**	Duration of smoking; past quit attempts; current cigarettes/day; alternative tobacco use[35,89,90], Fagerstrom Test for Nicotine Dependence[91]; stage of change[92,93]; Contemplation Ladder[94]
**COVID-19**	Comfort navigating hospital/attending medical appointment during COVID-19 outbreak[95-97]; Previous history of COVID-19 And Lung Infection/Pneumonia
**Comorbid conditions**	Alcohol Use Disorders Identification Test-Concise (AUDIT-C)[98,99];! Drug Abuse Screening Test (DAST-10)[100,101], and K6 screen for psychological distress[102,103]; single-item general health status (SF-1)[104,105]

3) Navigation log: The navigator will complete a log (Appendix E) capturing the following information each time he/she interacts with a participant in Arm 2.
- Name of study staff who completes navigation activity
- Date and time
- Participant ID number
- Who was contacted for navigation activity
- Contact modality for navigation activity
- Tasks/activities undertaken during encounter

4) Qualitative interview data elements:

Participant interviews: Participants will be asked to articulate their thought process in considering whether to pursue LCS and to identify the factors that most shaped their decision, including logistical, physical, or emotional barriers and facilitators. Based on our Health Belief Model framework (Table 2), we will prompt participants to consider the impact of the navigator on a) altering their perceived susceptibility to lung cancer, b) providing them with cues or reminders to pursue LCS, c) enhancing their self-efficacy for pursuing LCS, and d) helping them to overcome barriers to completing LCS. Among participants who undergo LDCT, we will assess their perspectives on the ease of the process, its impact on lung cancer worry, perceived lung cancer risk, and smoking behavior, and the need for any follow-up testing.

PCP interviews: These interviews will elicit PCP perspectives on the challenges of implementing LCS guideline in primary care practice for a vulnerable population and probe the ways in which the navigator did or did not help to reduce these challenges. We will also seek feedback from PCPs on ways to strengthen the integration of patient navigators into clinical care teams.

<u>B. Study Endpoints</u>

1) Primary outcome: The primary outcome is receipt of LDCT for LCS at 6 months. This will be based on radiology records verifying that a chest CT was performed for LCS and interpreted according to the Lung-RADS framework.[80] To ascertain this outcome, study staff will ask participants at the time of enrollment to provide written consent to obtain radiology records from Boston-area facilities that perform LDCT for LCS, as described above. At 6 months after enrollment, radiology records will be requested/obtained from these facilities. Most Boston-area hospital records are accessible through Care Everywhere in the BHCHP version of Epic, supporting the feasibility of gathering this information in an efficient manner. This approach does not rely on participant self-report and therefore does not require in-person or phone follow-up to ascertain. For those verified to have undergone LDCT for LCS within the 6-month timeframe, the Lung-RADS result along with any abnormal or incidental findings will be recorded.

2) Secondary outcome: Increasing LDCT completion will only reduce lung cancer mortality if individuals with abnormal results can undergo evaluation and treatment of early stage lung cancer in a timely fashion. In the setting of homelessness, a valid concern is whether participants with abnormal LDCT results can be recalled for diagnostic follow-up in a timely manner. To address this concern, our secondary outcome is receipt of LDCT with timely diagnostic follow-up of any abnormal results. To satisfy this composite outcome, participants must achieve the primary outcome (LDCT at 6 months) <u>and</u>, if the result is abnormal, also obtain the next recommended follow-up test within 1 month of the advised timeframe, based on the Lung-RADS framework.[80] Lung-RADS category descriptions, recommended follow-up, associated probabilities of malignancy, and estimated general population prevalences are shown in Table 3.[104] As described above, we will obtain radiology records for participants who underwent LDCT for LCS to document the findings of the study, determine the Lung-RADS category associated with those findings, and ascertain the recommended diagnostic follow-up plan. Participants with Lung-RADS 1-2 findings do not require specific diagnostic action, but rather may continue with annual screening at 12 months. These individuals will automatically satisfy the secondary outcome. In contrast, Lung-RADS 3-4 findings signal an increased risk for malignancy and merit sooner-than-annual follow-up testing. These individuals will satisfy the secondary outcome only if they obtain the next recommended diagnostic study within 1 month of the recommended timeframe. For example, if a participant has a Lung-RADS 3 finding for which follow-up LDCT in 6 months is recommended, we will assess whether this follow-up test occurred within 7 months of the initial LDCT. If a participant has a Lung-RADS 4B/X finding suggesting a high suspicion for malignancy, we will assess whether the recommended follow-up testing occurred within 1 month of the initial LDCT. The

Table 3. Lung-RADS categories.

Category	Description	Management	Malignancy probability	Gen. pop. prevalence
1	Negative	Repeat LDCT 12 mos	<1%	90%
2	Benign			
3	Probably benign	Repeat LDCT 6 mos	1-2%	5%
4A	Suspicious	Repeat LDCT 3 mos +/- PET/CT	5-15%	2%
4B/X		Chest CT, PET/CT, +/- biopsy	>15%	2%

*Adapted from the American College of Radiology

offer of delayed navigation to control arm participants will not interfere with this outcome because a) delayed navigation will be offered only to those who did not undergo an initial LCS LDCT within 6 months (i.e. the primary outcome), and b) attaining the primary outcome is necessary for satisfying the secondary outcome.

3) Other outcomes: We will examine time to receipt of LDCT for LCS. Process outcomes include acceptance of navigation when offered, number of contacts with the navigator, and attendance of a LCS SDM visit with a BHCHP PCP. Tobacco-related outcomes include receipt of a prescription for any smoking cessation medication and changes in clinically-recorded smoking status (i.e. quitting among current smokers or relapsing among former smokers), both ascertained by BHCHP medical record review. We will track the frequency and types of abnormal findings detected on LCS LDCT, any additional diagnostic testing or therapeutic changes pursued in response to these findings, and the percentage of participants diagnosed with lung cancer (based on radiology and BHCHP records review). We will also track diagnostic chest CT scans obtained for reasons other than LCS.

C. Statistical Methods

Quantitative analysis: LDCT completion at 6 months (1° outcome) and LDCT completion with timely diagnostic follow-up (2°outcome) are both binary outcomes. In the principal analysis, we will assess the difference between the immediate and delayed navigation arms on attainment of these outcomes using the Chi-square test. We will also use logistic regression models to examine the associations between treatment condition and outcomes including clinical site and other known predictors of outcomes to increase the precision of effect estimates. Additional analyses will examine time to receipt of LDCT for LCS between study arms using time-to-event methods (log-rank tests and Cox regression models). Although we expect to be underpowered to detect significant interactions, we will examine whether the effect of navigation varies by smoking status, housing status, or clinical site in an exploratory analysis.

Qualitative analysis: Qualitative interviews will be audiotaped and transcribed. We will use inductive content analysis methods to identify major and minor themes, and we will use deductive methods to strengthen, refine, and enhance understanding of identified themes.[105] Qualitative data coding will be performed by 2 trained study staff members using NVivo 11 software. The data coders will meet regularly with Drs. Baggett and Park to discuss the structural thematic framework, categories, and coding plan. We will resolve discrepancies through discussion and comparison to raw data. Coding will continue iteratively until a high level of reliability (Kappa>0.80) is established.

Integration with quantitative data: Based on a mixed methods approach,[106] we will use the qualitative interview data to interpret and explain the quantitative observations from the trial. This will aid in better understanding why overall LDCT completion was higher or lower than expected and why the navigation intervention did or did not improve these outcomes. Following

Table 4. Proposed integration of quantitative and qualitative data.

Health Belief Model domains	Measurement (method)	LCS LDCT at 6 months	
		Yes (N=20)	No (N=20)
Perceived susceptibility / severity	Baseline survey (quan)	Lung cancer risk perception, severity, and worry scores	
	Post-intervention interview (qual)	Quotes/themes about navigator impact on perceived susceptibility/severity	

Cues	Baseline survey (quan)	Past provider recommendation for LCS, exposure to LCS information
	Post-intervention interview (qual)	Quotes/themes about navigator provision of cues for LCS
Self-efficacy	Baseline survey (quan)	General and LCS-specific self-efficacy scores
	Post-intervention interview (qual)	Quotes/themes about navigator impact on self-efficacy
Perceived benefits / barriers	Baseline survey (quan)	LCS knowledge, competing subsistence difficulty scores
	Post-intervention interview (qual)	Quotes/themes about navigator assistance with barriers

from our stratified sampling scheme, we will integrate the quantitative and qualitative data by assessing for thematic differences in the interview content of participants who did (N=20) and did not (N=20) attain the primary outcome. We will focus in particular on comparing and contrasting these two groups' views on the utility of the navigator with respect to the Health Belief Model domains of perceived susceptibility, cues, self-efficacy, and barriers, as described above. We will additionally incorporate the quantitative baseline measures tapping these Health Belief Model domains into integrated analyses. Table 4 illustrates the way in which these quantitative and qualitative data elements will be integrated and presented in a blended fashion.

D. Power Analysis

The sample size justification is based on the primary outcome of LCS LDCT completion at 6 months. Internal BHCHP data suggests a presently negligible baseline rate of LCS LDCT. Allowing for improvement in this rate as LCS comes into wider practice, we will assume that 5% of usual care (Arm 1) participants will obtain an LDCT within 6 months. In our judgment, increasing this completion rate to 15% would represent a clinically meaningful change. We will have >80% power to detect such a difference with a sample size of 200 in the navigation arm (Arm 2) and 100 in the usual care arm (Arm 1).

VII. RISKS AND DISCOMFORTS

The potential risks and discomforts associated with this study are detailed below. The methods for mitigating these risks and monitoring the safety of participants are described in section IX.b.

A. Complications of surgical / non-surgical procedures

Not applicable.

B. Drug side effects and toxicities

Study staff will not be offering any drugs or over-the-counter products to participants.

C. Device complications

Not applicable.

D. Psychosocial (non-medical) risks

Potential risks to participants include psychological distress arising from the possibility of false-positive CT findings leading to additional diagnostic studies or procedures. Psychological and/or physical distress may also be brought about by discussing smoking and/or changing smoking behavior. Finally, because we will collect and record identifying information (e.g. name, date of

birth, and social security number), there is a risk to privacy if the data are compromised. Section IX (Monitoring and Quality Assurance) describes our methods for safeguarding the data and privacy of participants. The potential risks of this study are reasonable in relation to the potential benefits of reducing the risk of dying from lung cancer.

E. Radiation risks

There is a slight physical risk associated with exposure to radiation during CT scanning, although the "low-dose" nature of the test is designed to help mitigate this risk. For this reason, LDCT LCS for individuals at increased risk of lung cancer is recommended by a variety of professional societies since the benefits of screening for early stage cancer vastly outweigh the risks presented by small radiation exposures.

VIII. POTENTIAL BENEFITS

A. Potential benefits to participating individuals

Potential benefits for participants include the opportunity to receive assistance in completing LCS, which has been associated with a 20% reduction in lung cancer mortality. Additionally, current smokers will be informed of and referred to various resources to help them quit smoking, which may confer numerous health and financial benefits.

B. Potential benefits to society

Considerable knowledge could be gained from this study. To our knowledge, this will be the first study to focus on LCS among homeless people and the first randomized trial of any cancer screening intervention in this population. By focusing on an understudied topic area pertaining to a common cause of death among homeless people, our study fills an important gap in the literature and could serve as a blueprint for improving cancer screening and outcomes in homeless health care settings, particularly in the 295 HCH programs that serve 890,000 people annually in the US.[107]

IX. MONITORING AND QUALITY ASSURANCE

A. Data monitoring

All data will be collected using password-protected laptops or tablets with full disk encryption. We will use the Partners-hosted Research Electronic Data Capture (REDCap) application for data collection and management. REDCap is a secure, HIPAA compliant web-based application hosted by Partners HealthCare Research Computing, Enterprise Research Infrastructure & Services (ERIS). Because a Partners username is required for logging in to REDCap, all activity on study documents is electronically logged and therefore traceable. All data will be collected according to a standardized protocol preprogrammed into REDCap, which has built-in functions providing real-time data entry validation to help ensure accuracy and completeness. Each data collection form will have instructions or prompts for collecting the required data elements, and the response fields for each item will have appropriate ranges and formats to ensure that the data is entered in a valid way. Participants who agree to be photographed will have their photo taken using the same device on which the rest of the data is collected (encrypted laptop or

tablet). The photograph will be uploaded immediately to the REDCap database and the original photograph file will be permanently deleted from the device.

The PI will review all study data weekly to monitor its integrity. Additionally, the PI, biostatistician, and/or data analyst will download the study data on a weekly basis from the Partners REDCap server and back it up to the PI's SFA on the Partners network, which is protected by the Partners IS firewall, backed up nightly, and accessible only to authorized study staff. Only the minimum necessary number of study staff will have access to this data. Data analyses will be conducted by authorized study staff on the data files residing on the PI's Partners SFA. The data will not be transferred to investigators outside the Partners system.

The qualitative interviews conducted post-intervention will be recorded by a microphone connected directly to a password-protected, Partners-approved laptop or tablet with full-disk encryption. Audio files will be backed up to the PI's SFA described above and then deleted from the portable device.

If study activities are administered to participants using Enterprise Zoom or Microsoft Teams, personnel will always follow the MGB requirements for using video conferencing for research activities with no study visits or activities being audio- or video-recorded, with the exception of qualitative interviews, which will be audio-recorded with an encrypted laptop or tablet microphone (and not via the audio-recording capabilities within the Zoom or Teams platform) as described above. Study personnel will always follow the Principles of Privacy and share the minimum necessary amount of information.

B. Safety monitoring

Because of the narrow range of risks of the proposed study, there will not be a Data and Safety Monitoring Board for this trial. Instead, the safety of participants will be monitored by the PI on a continual basis. Navigators will stay in close contact with BHCHP PCPs and clinical staff to ensure the safety and well-being of all participants.

To minimize the risk to privacy, we will adhere to the data management procedures described above to help ensure the safety and integrity of data collected about study participants. All identifying information will be saved securely within the REDCap database. Paper ROI forms will be scanned into the PI's SFA and paper copies retained in a locked cabinet in the PI's office. Only the minimum necessary number of study staff will have access to this data. At the conclusion of the study, personal identifiers will be removed from the dataset and all analyses will occur in a de-identified fashion.

To help ensure the safety of participants, adverse events monitoring will be handled in the manner described below.

C. Outcomes monitoring

Because of the low-risk nature of the intervention, we will not conduct a formal interim analysis of the data nor define rules for early stopping of the trial.

D. Adverse event reporting guidelines

We will adhere to the Partners policy statement on "Reporting Unanticipated Problems including Adverse Events." Consistent with this policy, an adverse event will be defined as "any untoward

or unfavorable medical occurrence in a human subject, including any abnormal sign, symptom, or disease, temporally associated with the subject's participation in the research, whether or not considered related to the subject's participation in the research." The PI and his designated study staff will be responsible for the monitoring of adverse events in study participants.

Adverse events reported to the PI or other study staff will be documented in an adverse event report containing a description of the event, the date and time of onset, the date and time of resolution, the expectedness of the event, the relationship to the study, the seriousness of the event, and the action taken in response to the event. An adverse event will be deemed unexpected if the nature, severity, or frequency of the event is not consistent with a known or foreseeable risk given the research procedures, the characteristics of the subject population, or with an expected progression of an underlying condition. The following attribution scale will be used to describe the relationship of the adverse event to the study protocol: unrelated to the protocol, or possibly, probably, or definitely related to the protocol. A serious adverse event will be defined as an adverse event that results in death, is life-threatening, requires hospitalization, causes persistent or significant disability, or requires medical or surgical intervention.

A summary safety report detailing all adverse events and their handling will be included in annual study progress reports to the Partners IRB and the American Cancer Society. Unexpected adverse events of any severity that are at least possibly related to the research and suggest the research puts participants at increased harm will be reported to the Partners IRB within 5 working days or 7 calendar days. Serious adverse events that are unexpected and at least possibly related to the research will be assumed to suggest that the research puts participants at increased harm and will be reported to the Partners IRB within the same time frame. Any action resulting in a temporary or permanent suspension of this study will be reported to the American Cancer Society's program official. Adverse event reports and annual safety summaries will be documented by study ID number; personal identifiers will not be included in these reports.

E. Protocol adherence

1) Research staff: The study coordinators will be trained on the data collection protocols described above. Their understanding of these protocols will be assessed by asking them to demonstrate competency in using the REDCap data collection forms.

2) LCS navigator: To ensure adherence to the navigation intervention strategy, the navigator will receive in-depth training on patient navigation and focused training on tobacco cessation counseling.

a) Navigation training: The navigator will receive training in the core skills of patient navigation, including establishing rapport with patients while maintaining appropriate professional boundaries, motivational interviewing, performing an initial assessment with patients to explore barriers to LCS, working with patients to overcome these barriers, educating patients about the LCS process, introducing the concept of shared decision-making (SDM) around LCS, and empowering participants to discuss the benefits and risks of LCS with their PCP. Training will also encompass an overview of lung cancer, the rationale behind LCS, and the practical steps involved in completing LDCT so that the navigator can better counsel patients on what to expect from the LCS process. Additionally, the navigator will be trained in identifying and managing logistical hurdles to completing LCS, such as insurance coverage lapses that may need to be remedied before LDCT is feasible. To facilitate timely follow-up of abnormal LDCT results, the navigator will received focused education on the American College

of Radiology (ACR) Lung Imaging Reporting and Data System (Lung-RADS) framework.[80] Lung-RADS is a quality assurance tool designed to standardize LCS LDCT reporting and management recommendations, reduce confusion in LCS LDCT interpretations, and facilitate outcome monitoring.[80] The navigator training will be based on the protocol developed for Dr. Percac-Lima 's successful RCT of patient navigation for LCS among community health center patients (see section E). Dr. Percac-Lima, who has extensive experience in navigator training, will provide key components of this training and facilitate role playing sessions to reinforce skills. To the greatest extent possible, experienced navigators will be included in the training process. Depending upon prior experience, the navigator trainee will spend up to 2 weeks shadowing and observing an experienced navigator in the field prior to the start of the study. Refresher training and review of specific case scenarios will occur every 3 months throughout the study to ensure that the navigator is adhering faithfully to the intended roles and responsibilities.

b) Tobacco counseling training: The goal of the tobacco training component is to equip the navigator with the skills to provide a brief tobacco intervention. To achieve this goal, the navigator will complete an online 9-module course titled “Basic Skills for Working with Smokers,” sponsored by the University of Massachusetts Center for Tobacco Treatment Research & Training.[108] This course provides an introduction to the theory and practice of working with smokers in a health care setting. The navigator will additionally receive 4 hours of case-based teaching from Dr. Baggett on practical approaches to working with homeless smokers, followed by 6 hours of observing a certified Tobacco Treatment Specialist interact with and counsel smokers about tobacco cessation.

The navigator’s adherence to their intended duties will be assessed during weekly team meetings and through periodic audit of their navigation encounter logs.

X. REFERENCES

1. Burt MR, Aron LY, Douglas T, et al. Homelessness: Programs and the People They Serve: Findings of the National Survey of Homeless Assistance Providers and Clients: Technical Report. In. Washington, D.C.: U.S. Department of Housing and Urban Development, Office of Policy Development and Research; 1999.
2. Baggett TP, Chang Y, Porneala BC, Bharel M, Singer DE, Rigotti NA. Disparities in Cancer Incidence, Stage, and Mortality at Boston Health Care for the Homeless Program. *Am J Prev Med.* 2015;49(5):694-702.
3. Lamont DW, Toal FM, Crawford M. Socioeconomic deprivation and health in Glasgow and the west of Scotland--a study of cancer incidence among male residents of hostels for the single homeless. *J Epidemiol Community Health.* 1997;51(6):668-671.
4. Hwang SW, Wilkins R, Tjepkema M, O'Campo PJ, Dunn JR. Mortality among residents of shelters, rooming houses, and hotels in Canada: 11 year follow-up study. *BMJ.* 2009;339:b4036.
5. Baggett TP, Hwang SW, O'Connell JJ, et al. Mortality among homeless adults in Boston: shifts in causes of death over a 15-year period. *JAMA Intern Med.* 2013;173(3):189-195.
6. Baggett TP, Chang Y, Singer DE, et al. Tobacco-, alcohol-, and drug-attributable deaths and their contribution to mortality disparities in a cohort of homeless adults in Boston. *Am J Public Health.* 2015;105(6):1189-1197.
7. Aberle DR, Adams AM, Berg CD, et al. Reduced lung-cancer mortality with low-dose computed tomographic screening. *N Engl J Med.* 2011;365(5):395-409.
8. Chau S, Chin M, Chang J, et al. Cancer risk behaviors and screening rates among homeless adults in Los Angeles County. *Cancer Epidemiol Biomarkers Prev.* 2002;11(5):431-438.
9. Lebrun-Harris LA, Baggett TP, Jenkins DM, et al. Health status and health care experiences among homeless patients in federally supported health centers: findings from the 2009 patient survey. *Health services research.* 2013;48(3):992-1017.
10. Asgary R, Garland V, Jakubowski A, Sckell B. Colorectal cancer screening among the homeless population of New York City shelter-based clinics. *Am J Public Health.* 2014;104(7):1307-1313.
11. Asgary R, Garland V, Sckell B. Breast Cancer Screening Among Homeless Women of New York City Shelter-Based Clinics. *Womens Health Issues.* 2014.
12. Long HL, Tulsky JP, Chambers DB, et al. Cancer screening in homeless women: attitudes and behaviors. *J Health Care Poor Underserved.* 1998;9(3):276-292.
13. Baggett TP, Rigotti NA. Cigarette smoking and advice to quit in a national sample of homeless adults. *American Journal of Preventive Medicine.* 2010;39(2):164-172.
14. Connor SE, Cook RL, Herbert MI, Neal SM, Williams JT. Smoking cessation in a homeless population: there is a will, but is there a way? *Journal of General Internal Medicine.* 2002;17(5):369-372.
15. Snyder LD, Eisner MD. Obstructive lung disease among the urban homeless. *Chest.* 2004;125(5):1719-1725.
16. Szerlip MI, Szerlip HM. Identification of cardiovascular risk factors in homeless adults. *Am J Med Sci.* 2002;324(5):243-246.
17. Tsai J, Rosenheck RA. Smoking Among Chronically Homeless Adults: Prevalence and Correlates. *Psychiatr Serv.* 2012;63(6):569-576.
18. Torchalla I, Strehlau V, Okoli CT, Li K, Schuetz C, Krausz M. Smoking and predictors of nicotine dependence in a homeless population. *Nicotine Tob Res.* 2011;13(10):934-942.
19. Hahn JA, Kushel MB, Bangsberg DR, Riley E, Moss AR. BRIEF REPORT: the aging of the homeless population: fourteen-year trends in San Francisco. *J Gen Intern Med.* 2006;21(7):775-778.

20. Moyer VA. Screening for lung cancer: U.S. Preventive Services Task Force recommendation statement. *Ann Intern Med.* 2014;160(5):330-338.
21. Jenson TS, Chin J, Ashby L, Hermansen J, Hutter JD. Decision Memo for Screening for Lung Cancer with Low Dose Computed Tomography (LDCT) (CAG-00439N). . In. Baltimore, MD: Centers for Medicare & Medicaid Services; 2015.
22. Gelberg L, Gallagher TC, Andersen RM, Koegel P. Competing priorities as a barrier to medical care among homeless adults in Los Angeles. *Am J Public Health.* 1997;87(2):217-220.
23. Baggett TP, O'Connell JJ, Singer DE, Rigotti NA. The unmet health care needs of homeless adults: a national study. *Am J Public Health.* 2010;100(7):1326-1333.
24. Kushel MB, Vittinghoff E, Haas JS. Factors associated with the health care utilization of homeless persons. *JAMA.* 2001;285(2):200-206.
25. Weinstein LC, LaNoue M, Hurley K, Sifri R, Myers R. Using Concept Mapping to Explore Barriers and Facilitators to Breast Cancer Screening in Formerly Homeless Women with Serious Mental Illness. *J Health Care Poor Underserved.* 2015;26(3):908-925.
26. Fazel S, Khosla V, Doll H, Geddes J. The prevalence of mental disorders among the homeless in western countries: systematic review and meta-regression analysis. *PLoS Med.* 2008;5(12):e225.
27. Koegel P, Burnam MA, Farr RK. The prevalence of specific psychiatric disorders among homeless individuals in the inner city of Los Angeles. *Arch Gen Psychiatry.* 1988;45(12):1085-1092.
28. Brown RT, Kiely DK, Bharel M, Mitchell SL. Geriatric syndromes in older homeless adults. *J Gen Intern Med.* 2011;27(1):16-22.
29. Topolovec-Vranic J, Ennis N, Colantonio A, et al. Traumatic brain injury among people who are homeless: a systematic review. *BMC Public Health.* 2012;12:1059.
30. Topolovec-Vranic J, Ennis N, Howatt M, et al. Traumatic brain injury among men in an urban homeless shelter: observational study of rates and mechanisms of injury. *CMAJ Open.* 2014;2(2):E69-76.
31. Hwang SW, Colantonio A, Chiu S, et al. The effect of traumatic brain injury on the health of homeless people. *CMAJ.* 2008;179(8):779-784.
32. Oddy M, Moir JF, Fortescue D, Chadwick S. The prevalence of traumatic brain injury in the homeless community in a UK city. *Brain Inj.* 2012;26(9):1058-1064.
33. Baggett TP, Campbell EG, Chang Y, Magid LM, Rigotti NA. Posttraumatic Stress Symptoms and Their Association With Smoking Outcome Expectancies Among Homeless Smokers in Boston. *Nicotine Tob Res.* 2015.
34. Baggett TP, Rigotti NA, Campbell EG. Cost of Smoking among Homeless Adults. *N Engl J Med.* 2016;374(7):697-698.
35. Baggett TP, Campbell EG, Chang Y, Rigotti NA. Other tobacco product and electronic cigarette use among homeless cigarette smokers. *Addict Behav.* 2016;60:124-130.
36. Percac-Lima S, Ashburner JM, Bond B, Oo SA, Atlas SJ. Decreasing disparities in breast cancer screening in refugee women using culturally tailored patient navigation. *J Gen Intern Med.* 2013;28(11):1463-1468.
37. Percac-Lima S, Lopez L, Ashburner JM, Green AR, Atlas SJ. The longitudinal impact of patient navigation on equity in colorectal cancer screening in a large primary care network. *Cancer.* 2014;120(13):2025-2031.
38. Percac-Lima S, Ashburner JM, Zai AH, et al. Patient Navigation for Comprehensive Cancer Screening in High-Risk Patients Using a Population-Based Health Information Technology System: A Randomized Clinical Trial. *JAMA Intern Med.* 2016;176(7):930-937.
39. Freeman HP. Patient navigation: a community based strategy to reduce cancer disparities. *J Urban Health.* 2006;83(2):139-141.

40. Asgary R, Naderi R, Wisnivesky J. Opt-Out Patient Navigation to Improve Breast and Cervical Cancer Screening Among Homeless Women. *J Womens Health (Larchmt).* 2017.
41. Braun KL, Thomas WL, Jr., Domingo JL, et al. Reducing cancer screening disparities in medicare beneficiaries through cancer patient navigation. *J Am Geriatr Soc.* 2015;63(2):365-370.
42. Myers RE, Sifri R, Daskalakis C, et al. Increasing colon cancer screening in primary care among African Americans. *J Natl Cancer Inst.* 2014;106(12).
43. Honeycutt S, Green R, Ballard D, et al. Evaluation of a patient navigation program to promote colorectal cancer screening in rural Georgia, USA. *Cancer.* 2013;119(16):3059-3066.
44. Wang X, Fang C, Tan Y, Liu A, Ma GX. Evidence-based intervention to reduce access barriers to cervical cancer screening among underserved Chinese American women. *J Womens Health (Larchmt).* 2010;19(3):463-469.
45. Paskett ED, Harrop JP, Wells KJ. Patient navigation: an update on the state of the science. *CA Cancer J Clin.* 2011;61(4):237-249.
46. Phillips CE, Rothstein JD, Beaver K, Sherman BJ, Freund KM, Battaglia TA. Patient navigation to increase mammography screening among inner city women. *J Gen Intern Med.* 2010;26(2):123-129.
47. Robinson-White S, Conroy B, Slavish KH, Rosenzweig M. Patient navigation in breast cancer: a systematic review. *Cancer Nurs.* 2010;33(2):127-140.
48. Wells KJ, Battaglia TA, Dudley DJ, et al. Patient navigation: state of the art or is it science? *Cancer.* 2008;113(8):1999-2010.
49. Chen LA, Santos S, Jandorf L, et al. A program to enhance completion of screening colonoscopy among urban minorities. *Clin Gastroenterol Hepatol.* 2008;6(4):443-450.
50. Jandorf L, Braschi C, Ernstoff E, et al. Culturally targeted patient navigation for increasing african americans' adherence to screening colonoscopy: a randomized clinical trial. *Cancer Epidemiol Biomarkers Prev.* 2013;22(9):1577-1587.
51. Lasser KE, Murillo J, Medlin E, et al. A multilevel intervention to promote colorectal cancer screening among community health center patients: results of a pilot study. *BMC Fam Pract.* 2009;10:37.
52. Christie J, Itzkowitz S, Lihau-Nkanza I, Castillo A, Redd W, Jandorf L. A randomized controlled trial using patient navigation to increase colonoscopy screening among low-income minorities. *J Natl Med Assoc.* 2008;100(3):278-284.
53. Jandorf L, Gutierrez Y, Lopez J, Christie J, Itzkowitz SH. Use of a patient navigator to increase colorectal cancer screening in an urban neighborhood health clinic. *J Urban Health.* 2005;82(2):216-224.
54. Lasser KE, Murillo J, Lisboa S, et al. Colorectal cancer screening among ethnically diverse, low-income patients: a randomized controlled trial. *Archives of internal medicine.* 2011;171(10):906-912.
55. Horne HN, Phelan-Emrick DF, Pollack CE, et al. Effect of patient navigation on colorectal cancer screening in a community-based randomized controlled trial of urban African American adults. *Cancer Causes Control.* 2015;26(2):239-246.
56. Percac-Lima S, Grant RW, Green AR, et al. A culturally tailored navigator program for colorectal cancer screening in a community health center: a randomized, controlled trial. *J Gen Intern Med.* 2009;24(2):211-217.
57. Hoffman HJ, LaVerda NL, Young HA, et al. Patient navigation significantly reduces delays in breast cancer diagnosis in the District of Columbia. *Cancer Epidemiol Biomarkers Prev.* 2012;21(10):1655-1663.

58. Markossian TW, Darnell JS, Calhoun EA. Follow-up and timeliness after an abnormal cancer screening among underserved, urban women in a patient navigation program. *Cancer Epidemiol Biomarkers Prev.* 2012;21(10):1691-1700.
59. Battaglia TA, Bak SM, Heeren T, et al. Boston Patient Navigation Research Program: the impact of navigation on time to diagnostic resolution after abnormal cancer screening. *Cancer Epidemiol Biomarkers Prev.* 2012;21(10):1645-1654.
60. Raich PC, Whitley EM, Thorland W, Valverde P, Fairclough D. Patient navigation improves cancer diagnostic resolution: an individually randomized clinical trial in an underserved population. *Cancer Epidemiol Biomarkers Prev.* 2012;21(10):1629-1638.
61. Ramirez AG, Perez-Stable EJ, Penedo FJ, et al. Navigating Latinas with breast screen abnormalities to diagnosis: the Six Cities Study. *Cancer.* 2013;119(7):1298-1305.
62. Lee JH, Fulp W, Wells KJ, Meade CD, Calcano E, Roetzheim R. Patient navigation and time to diagnostic resolution: results for a cluster randomized trial evaluating the efficacy of patient navigation among patients with breast cancer screening abnormalities, Tampa, FL. *PLoS One.* 2013;8(9):e74542.
63. Lee JH, Fulp W, Wells KJ, Meade CD, Calcano E, Roetzheim R. Effect of patient navigation on time to diagnostic resolution among patients with colorectal cancer-related abnormalities. *J Cancer Educ.* 2014;29(1):144-150.
64. American Cancer Society. Patient Navigators Help Cancer Patients Manage Care. https://www.cancer.org/latest-news/navigators-help-cancer-patients-manage-their-care.html. Published February 24, 2017. Accessed March 1, 2017.
65. National Cancer Institute. Patient Navigation Research Program (PNRP). U.S. Department of Health and Human Services, National Institutes of Health, National Cancer Institute. https://www.cancer.gov/about-nci/organization/crchd/disparities-research/pnrp. Accessed March 28, 2017.
66. O'Connell JJ, Oppenheimer SC, Judge CM, et al. The Boston Health Care for the Homeless Program: a public health framework. *Am J Public Health.* 2010;100(8):1400-1408.
67. Bauer LK, Brody JK, Leon C, Baggett TP. Characteristics of Homeless Adults Who Died of Drug Overdose: A Retrospective Record Review. *J Health Care Poor Underserved.* 2016;27(2):846-859.
68. Bharel M, Lin WC, Zhang J, O'Connell E, Taube R, Clark RE. Health care utilization patterns of homeless individuals in Boston: preparing for Medicaid expansion under the Affordable Care Act. *Am J Public Health.* 2013;103 Suppl 2:S311-317.
69. Tsai D. MassHealth Transmittal Letter PHY-147. In: Office of Medicaid, Massachusetts Department of Health and Human Services; March 2016.
70. Wender R, Fontham ET, Barrera E, Jr., et al. American Cancer Society lung cancer screening guidelines. *CA Cancer J Clin.* 2013;63(2):107-117.
71. Detterbeck FC, Mazzone PJ, Naidich DP, Bach PB. Screening for lung cancer: Diagnosis and management of lung cancer, 3rd ed: American College of Chest Physicians evidence-based clinical practice guidelines. *Chest.* 2013;143(5 Suppl):e78S-92S.
72. Jaklitsch MT, Jacobson FL, Austin JH, et al. The American Association for Thoracic Surgery guidelines for lung cancer screening using low-dose computed tomography scans for lung cancer survivors and other high-risk groups. *J Thorac Cardiovasc Surg.* 2012;144(1):33-38.
73. Samet JH, Crowell R, Estepar RSJ, et al. Providing Guidance on Lung Cancer Screening to Patients and Physicians: An Update from the American Lung Association Lung Cancer Screening Committee. . In. Chicago: American Lung Association; April 30, 2015.

74. Bach PB, Mirkin JN, Oliver TK, et al. Benefits and harms of CT screening for lung cancer: a systematic review. *JAMA.* 2012;307(22):2418-2429.
75. Park ER, Streck JM, Gareen IF, et al. A qualitative study of lung cancer risk perceptions and smoking beliefs among national lung screening trial participants. *Nicotine Tob Res.* 2014;16(2):166-173.
76. Park ER, Ostroff JS, Rakowski W, et al. Risk perceptions among participants undergoing lung cancer screening: baseline results from the National Lung Screening Trial. *Ann Behav Med.* 2009;37(3):268-279.
77. Ryan H, Trosclair A, Gfroerer J. Adult current smoking: differences in definitions and prevalence estimates--NHIS and NSDUH, 2008. *J Environ Public Health.* 2012;2012:918368.
78. Health Resources and Services Administration, Bureau of Primary Health Care. Authorizing Legislation of the Health Center Program: Section 330 of the Public Health Service Act (42 USCS § 254b). http://bphc.hrsa.gov/policiesregulations/legislation/authorizing330.pdf. Accessed March 28, 2017.
79. U.S. Department of Housing and Urban Development. S. 896 HEARTH Act of 2009. https://www.hudexchange.info/resource/1717/s-896-hearth-act/. Accessed March 12, 2017.
80. American College of Radiology. Lung CT Screening Reporting and Data System (Lung-RADS). http://www.acr.org/Quality-Safety/Resources/LungRADS. Accessed March 28, 2017.
81. Fiore MC, Jaen CR, Baker TB, et al. Treating Tobacco Use and Dependence: 2008 Update. Clinical Practice Guideline. . In. Rockville, MD: U.S. Department of Health and Human Services. Public Health Service; May 2008.
82. Park ER, Gareen IF, Japuntich S, et al. Primary Care Provider-Delivered Smoking Cessation Interventions and Smoking Cessation Among Participants in the National Lung Screening Trial. *JAMA Intern Med.* 2015;175(9):1509-1516.
83. Poghosyan H, Kennedy Sheldon L, Cooley ME. The impact of computed tomography screening for lung cancer on smoking behaviors: a teachable moment? *Cancer Nurs.* 2012;35(6):446-475.
84. Greene J, Fahrney K, Byron M. Health Care for the Homeless User/Visit Surveys, RTI Project Number 07147.021. . In. Research Triangle Park, NC: RTI International; 2004.
85. Kaufman AR, Koblitz AR, Persoskie A, et al. Factor Structure and Stability of Smoking-Related Health Beliefs in the National Lung Screening Trial. *Nicotine Tob Res.* 2016;18(3):321-329.
86. Lowenstein LM, Richards VF, Leal VB, et al. A brief measure of Smokers' knowledge of lung cancer screening with low-dose computed tomography. *Prev Med Rep.* 2016;4:351-356.
87. Schwarzer R, Jerusalem M. Generalized Self-Efficacy scale. In: Weinman J, Wright S, Johnston M, eds. *Measures in health psychology: A user's portfolio. Causal and control beliefs.* Windsor, UK: NFER-NELSON; 1995:35-37.
88. Scholz U, Gutierrez Dona B, Sud S, Schwarzer R. Is General Self-Efficacy a Universal Construct? Psychometric Findings from 25 Countries. *European Journal of Psychological Assessment.* 2002;18(3):242-251.
89. US Department of Commerce CB. National Cancer Institute and Food and Drug Administration co-sponsored Tobacco Use Supplement to the Current Population Survey. In. *2018-2019*2020.
90. Kish DH, Reitzel LR, Kendzor DE, Okamoto H, Businelle MS. Characterizing Concurrent Tobacco Product Use Among Homeless Cigarette Smokers. *Nicotine Tob Res.* 2014.

91. Heatherton TF, Kozlowski LT, Frecker RC, Fagerstrom KO. The Fagerstrom Test for Nicotine Dependence: a revision of the Fagerstrom Tolerance Questionnaire. *Br J Addict.* 1991;86(9):1119-1127.
92. DiClemente CC, Prochaska JO, Fairhurst SK, Velicer WF, Velasquez MM, Rossi JS. The process of smoking cessation: an analysis of precontemplation, contemplation, and preparation stages of change. *J Consult Clin Psychol.* 1991;59(2):295-304.
93. Snow MG, Prochaska JO, Rossi JS. Stages of change for smoking cessation among former problem drinkers: a cross-sectional analysis. *J Subst Abuse.* 1992;4(2):107-116.
94. Biener L, Abrams DB. The Contemplation Ladder: validation of a measure of readiness to consider smoking cessation. *Health Psychol.* 1991;10(5):360-365.
95. Wong LE, Hawkins JE, Murrell KL. Where are all the patients? Addressing Covid-19 fear to encourage sick patients to seek emergency care. *NEJM Catalyst Innovations in Care Delivery.* 2020.
96. Toolkit P. COVID-19 Community Response Survey Guidance. In:2020.
97. (C3PNO) CCoCPNO. COVID-19 Module for NIDA-Supported Cohorts Affected by HIV and Substance Use. In:2020.
98. Bush K, Kivlahan DR, McDonell MB, Fihn SD, Bradley KA. The AUDIT alcohol consumption questions (AUDIT-C): an effective brief screening test for problem drinking. *Archives of internal medicine.* 1998;158(16):1789-1795.
99. Bradley KA, Bush KR, Epler AJ, et al. Two brief alcohol-screening tests From the Alcohol Use Disorders Identification Test (AUDIT): validation in a female Veterans Affairs patient population. *Archives of internal medicine.* 2003;163(7):821-829.
100. Skinner HA. The drug abuse screening test. *Addictive behaviors.* 1982;7(4):363-371.
101. Yudko E, Lozhkina O, Fouts A. A comprehensive review of the psychometric properties of the Drug Abuse Screening Test. *Journal of substance abuse treatment.* 2007;32(2):189-198.
102. Kessler RC, Andrews G, Colpe LJ, et al. Short screening scales to monitor population prevalences and trends in non-specific psychological distress. *Psychological medicine.* 2002;32(6):959-976.
103. Kessler RC, Barker PR, Colpe LJ, et al. Screening for serious mental illness in the general population. *Arch Gen Psychiatry.* 2003;60:184-189.
104. American College of Radiology. Lung-RADS Version 1.0 Assessment Categories. Available at http://www.acr.org/Quality-Safety/Resources/LungRADS. In:April 28, 2014.
105. Elo S, Kyngas H. The qualitative content analysis process. *J Adv Nurs.* 2008;62(1):107-115.
106. Creswell JW, Plano Clark VL. Chapter 3: Choosing a Mixed Methods Design. In: *Designing and Conducting Mixed Methods Research.* 2nd ed. Los Angeles: Sage; 2011:53-106.
107. Health Resources and Services Administration, U.S. Department of Health & Human Services. 2015 Health Center Data: National Health Care for the Homeless Program Grantee Data. https://bphc.hrsa.gov/uds/datacenter.aspx?year=2015&fd=ho. Accessed March 16, 2017.
108. Center for Tobacco Treatment Research & Training, University of Massachusetts Medical School. Basic Skills for Working with Smokers - Online Course. http://www.umassmed.edu/tobacco/training/basicskills_online/. Accessed March 28, 2017.

TITLE: Lung cancer screening navigation for homeless people: A pragmatic trial

PRINCIPAL INVESTIGATOR: Travis P. Baggett, MD, MPH

GRANT NUMBER: 131162-RSG-17-157-01-CPPB (American Cancer Society)

VERSION DATE: June 17, 2021

I. BACKGROUND AND SIGNIFICANCE

A. Historical background

An estimated 2.3-3.5 million people experience homelessness annually in the US.[1] Lung cancer incidence and mortality are substantially higher in this vulnerable population than in comparably-aged non-homeless individuals.[2-4] To address this disparity, we propose a pragmatic clinical trial to test the effect of patient navigation on lung cancer screening (LCS) completion among homeless-experienced adults at increased risk for lung cancer. Work that we and others have done suggests 2 reasons why such an intervention is needed.

1) Lung cancer is a major cause of death among homeless adults and the leading cause of tobacco-attributable death in this population.

In our study of 28,033 adults who used Boston Health Care for the Homeless Program (BHCHP) services in 2003-08, cancer was the second-leading cause of death and the leading killer of adults ≥45 years old.[5] Lung cancer accounted for one-third of these cancer deaths.[5] In a subsequent study of cancer epidemiology in this cohort, lung cancer was the leading type of incident cancer and the leading cause of cancer death among both men and women, with age-standardized incidence and mortality rates exceeding those in the Massachusetts adult population more than 2-fold.[2] An estimated 88% of incident lung cancer cases[2] and 93% of lung cancer deaths[6] were attributable to tobacco smoking, making lung cancer the leading type of smoking-attributable cancer and the leading cause of smoking-attributable death among homeless people. The excess burden of lung cancer in these studies of homeless adults in Boston was similar to that observed in studies of homeless and marginally housed people in Canada[4] and Glasgow[3] (Table 1), suggesting that these disparities are not geographically constrained. This body of evidence highlights the need for interventions to reduce lung cancer disparities in homeless populations. One avenue for doing so is LCS with low-dose computed tomography (LDCT) to promote detection and potentially curative treatment of early-stage lung cancer. This strategy was associated with a 20% reduction in lung cancer mortality in the National Lung Screening Trial (NLST).[7] However, implementing such a strategy presents formidable hurdles in the setting of homelessness.

Table 1. Lung cancer among homeless adults

Study location (yrs)	SIR/SMR (95% CI)
Boston (2003-2008)	
Incidence, men	2.30 (1.84, 2.84)
Incidence, women	2.23 (1.41, 3.35)
Mortality, men	2.39 (1.83, 3.08)
Mortality, women	2.31 (1.26, 3.88)
Canada (1991-2001)	
Mortality, men	1.91 (1.67, 2.18)
Mortality, women	1.73 (1.26, 2.36)
Glasgow (1975-1993)	
Incidence, men	1.64 (1.41, 1.86)

2) Homeless people have suboptimal rates of cancer screening and are diagnosed with screen-detectable malignancies at later stages than non-homeless people; barriers to implementing LCS in the setting of homelessness could exacerbate existing disparities in lung cancer outcomes.

In the 2003-08 BHCHP study of cancer epidemiology described above, homeless individuals were diagnosed with colorectal cancer and female breast cancer at significantly later stages than adults in the Massachusetts general population.[2] Several studies have documented low rates of screening for these malignancies among homeless individuals.[8-12] Given the high prevalence of smoking among homeless people[8,13-18] and the aging of the homeless population,[19] many homeless individuals are expected to meet the recommended criteria for consideration of LCS with LDCT[20,21]; however, as with colorectal and breast cancer screening, there are several obstacles to successfully implementing LCS in homeless populations. Homeless individuals struggling to meet basic subsistence needs may place a low priority on screening for asymptomatic illness[22] or may feel too emotionally overwhelmed to cope with the possibility of an abnormal result. Additionally, despite a high burden of cancer risk factors, most homeless people appear to believe that their susceptibility to cancer is equal to or less than that of others the same age.[8] Lack of insurance may pose another barrier,[23,24] although this is less of a concern in Massachusetts and could improve elsewhere in states that expanded Medicaid under the Affordable Care Act. Even among insured individuals who are interested in obtaining LDCT screening, the process of scheduling and attending the test may prove logistically challenging because of difficulty navigating complex facilities and care systems,[25] especially for the large proportions of homeless individuals with psychiatric and cognitive impairments.[26-32] Furthermore, ensuring timely follow-up of abnormal results may be challenging because of communication barriers and competing psychosocial issues. These barriers introduce the possibility that existing disparities in lung cancer mortality could worsen if homeless people are less able to complete LCS than other segments of the population.

B. Preliminary data

Our prior epidemiologic work demonstrating the burden of lung cancer disparities among homeless people is described above and provides the motivation for the proposed study. In addition, this study is informed by administrative data from BHCHP, survey and clinical trial studies with homeless smokers conducted by the PI (Dr. Baggett), navigation studies conducted by co-investigator Dr. Percac-Lima, and LCS-oriented studies conducted by co-investigators Dr. Park and Dr. Rigotti. Collectively, this preliminary data has demonstrated the following:

1) We have the patient volume to support this study. Based on internal data from the BHCHP Institute, 3,443 unique individuals aged 55-77 years old were seen at any BHCHP site from 9/1/2015 to 9/1/2016. Of these, 53% were current smokers and 23% were former smokers, totaling 2,616 potentially eligible individuals seen by the program in a 12-month period. When extrapolated over the planned 2.5-year enrollment period, we anticipate being able to recruit and randomize the proposed number of 300 participants.

2) We have characterized the target population. In April-July 2014, we used time-location sampling to conduct a survey of 306 homeless adult smokers at 5 high-volume BHCHP clinical sites.[33-35] The response rate was 86%. One-quarter of respondents (N=78) were 55-77 years old. Of these, the vast majority were non-Hispanic white or black men with a high school degree. Seventy percent had ever experienced a traumatic head injury, 64% had used any drug in the past month, 39% had consumed alcohol to intoxication in the past month, 60% reported feeling seriously depressed, and half screened positive for PTSD. One-third reported COPD and 17% reported cardiovascular disease. Past-month difficulty finding shelter, food, and clothing were common. The mean number of cigarettes per day was 12 and the mean Fagerstrom nicotine dependence score was 3.8 (range 0-12). These findings highlight the medical complexity and competing life issues of older homeless smokers and underscore the need for a navigation-based intervention to reduce the barriers to LCS.

3) We can recruit and longitudinally retain homeless people in a clinical trial. From October 2015 to June 2016, we conducted a 3-arm (N=25 per arm) pilot randomized controlled trial testing the effect of a) contingent financial rewards for smoking abstinence, and b) text messaging to support smoking abstinence, against c) a shared control condition, among homeless smokers at BHCHP (NCT02565381). Sixty-eight percent of eligible individuals enrolled, and we reached our target enrollment within 6 months. Study attendance and retention rates were excellent. Of 14 possible assessment visits, participants attended a median of 10, with 97% attending ≥1 visit and 77% attending ≥7 visits. These findings demonstrate that our team has the expertise to conduct a behavioral treatment trial in the context of homelessness and to engage and retain this vulnerable population in a longitudinal fashion. This is facilitated by our extensive knowledge of the target population, our person-centered approach to participant engagement, our strong partnership with BHCHP, and the central location of BHCHP headquarters with respect to other homeless services.

4) Our navigation intervention is based on a model that has proven effective at improving lung and other cancer screening among underserved patient populations in Boston. Key collaborator, Dr. Sanja Percac-Lima, has developed and tested navigation interventions that have been associated with improved cancer screening rates among diverse, low-income community health center patients in Boston.[36,37] She conducted a randomized controlled trial (RCT) across 18 clinical practices in an academic primary care network evaluating the impact of patient navigation relative to usual care for high-risk individuals who were overdue for breast, colorectal, and/or cervical cancer screening.[38] Patients assigned to navigation were significantly more likely to complete screening for breast (23.4% vs. 16.6%, p=0.009), cervical (14.4% vs. 8.6%, p=0.007), and colorectal (13.7% vs. 7.0%, p<0.001) cancer during the follow-up period.[38] More recently, with grant support from ACS and in collaboration with Drs. Park and Rigotti, Dr. Percac-Lima was the principal investigator of an RCT testing the effect of patient navigation on chest CT completion among low-income patients eligible for LCS at 5 MGH-affiliated health centers in greater Boston. Preliminary analysis of data after 11 months of follow-up has shown that participants in the navigation arm were significantly more likely to obtain a screening lung CT than participants assigned to usual care (24% vs. 9%, p<0.01). The LCS navigation approach used in our study will be based on these successful models.

C. Study rationale

Mitigating disparities in lung cancer outcomes and fully realizing the potential benefits of LCS among homeless people will require thoughtful interventions deployed through the clinical programs that serve this vulnerable population. Patient navigation, a strategy for guiding individuals through complex health systems,[39] may be a promising approach for helping homeless people overcome their unique barriers to LCS.[40] Navigation interventions have been shown to improve cancer screening participation[36,37,41-56] and diagnostic resolution of abnormal results[57-63] in vulnerable populations. Patient navigation has been endorsed by the ACS[64] and the National Cancer Institute[65] as a valuable approach to reducing cancer health disparities,[65] but this approach has not been tested rigorously in a homeless health care setting. Our research group is well-poised to conduct such a study by leveraging our extensive clinical and research experience with this population and our 9-year partnership with BHCHP, an internationally-renowned organization that serves over 12,000 currently (84%) and formerly (16%) homeless people annually through dozens of clinical sites in greater Boston.[66] This innovative community partnership has given rise to numerous impactful studies on the health of homeless-experienced people,[2,5,6,33-35,67] including a recently pilot trial for homeless smokers that had brisk recruitment, high retention rates, and encouraging results (NCT02565381).

II. SPECIFIC AIMS

Aim 1: To determine the effect of patient navigation, added to usual care, on 1°) LCS LDCT completion at 6 months (26 weeks) and 2°) LCS LDCT completion at 6 months (26 weeks) and diagnostic follow-up of abnormal results within 1 month (4 weeks) of the recommended timeframe, among homeless-experienced people who are eligible for LCS.

Hypothesis: Participants assigned to receive patient navigation will have significantly greater 1°) LCS LDCT completion at 6 months (26 weeks) and 2°) LCS LDCT completion at 6 months (26 weeks) with timely diagnostic follow-up of abnormal results.

Aim 2. To conduct post-intervention qualitative interviews of trial participants and BHCHP PCPs to explain and interpret the quantitative findings of the pragmatic clinical trial.

Based on a sequential explanatory mixed methods approach, Aim 2 interviews will examine how and why participants decided whether to undergo LCS, the barriers they encountered in doing so, the ways in which the navigator helped them overcome these barriers, and opportunities for improving the intervention for future use.

III. SUBJECT SELECTION

A. Inclusion and exclusion criteria

To be eligible to participate, individuals must meet **all** the following criteria:

1) Aged 55-77 years old, assessed by self-report and verified by date of birth.
   Rationale: LCS LCDT is covered only for patients aged 55-77.
2) Have a 30 pack-year smoking history *and* have smoked within the past 15 years.
   Rationale: We will be recruiting exclusively smokers because they are at the highest risk for lung cancer and therefore stand to receive the most benefit from a LCS. These smoking history parameters are consistent with current practice guidelines and Medicare eligibility criteria.
3) Have a BHCHP primary care provider (PCP).
   Rationale: Patient navigation is designed to support and extend services provided by medical practitioners. Having a PCP will help to ensure longitudinal medical oversight of the LCS process and appropriate follow-up of abnormal results.
4) Are currently or formerly homeless, assessed by self-report and defined as ever having experienced a time in their life where they did not have fixed, regular housing.
   Rationale: Both currently and formerly homeless individuals were included in our epidemiologic analysis showing a more than 2-fold higher incidence and mortality rate from lung cancer compared to the general Massachusetts adult population.[2] BHCHP and most HCH programs nationally continue to serve patients after they have gained housing.
5) Be proficient in English, assessed with items asking about native language and self-reported comfort communicating in English among non-native speakers.
   Rationale: Because of a budget reduction by the funding agency, we do not have the resources to develop study materials or conduct in-person navigation in languages other than English. Additionally, the vast majority of individuals in the target population speak English.

Individuals meeting **any** of the following criteria will be excluded from the study:

1) A recent prior chest computed tomography (CT) imaging (in the past 12 months).
   Rationale: This study is aimed at individuals who are most likely to benefit from LCS, based on evidence from the National Lung Screening Trial. Potential participants that have recently received a chest CT will have recently been scanned for lung cancer, therefore an additional screening will not benefit their health outcomes.
2) Any personal history of lung cancer, or current presentation with symptoms concerning for lung cancer (e.g. hemoptysis or unexplained weight loss of >15 lbs. in the past year).
   Rationale: Individuals with a history of lung cancer or symptoms concerning for lung cancer require surveillance or diagnostic lung imaging, respectively, and are not appropriate candidates for screening lung imaging, which by definition is designed for individuals without symptoms or history of the illness being screened for.
3) Inability to provide informed consent, assessed with knowledge questions about the material presented during the informed consent process that individuals must correctly answer before providing informed consent to participate.
   Rationale: In this vulnerable population, we wish to take a conservative approach to the informed consent process to help ensure that participants fully understand the pros and cons of participating in the research study.
4) PCP is the study PI (Dr. Travis Baggett).
   Rationale: BHCHP patients under the primary care of Dr. Baggett may feel unduly influenced to participate. In addition, since the primary study outcome (receipt of LDCT for LCS) requires PCP involvement, the inclusion of Dr. Baggett's patients creates a conflict of interest and the potential for bias.

B. Source of subjects and recruitment methods

This study will be registered with ClinicalTrials.gov prior to the recruitment and enrollment of human subjects. All participants will be recruited from BHCHP clinical sites. BHCHP does not have its own IRB but instead will rely on the Partners Human Research Committee for IRB review through a reliance agreement initiated through SMART IRB. In addition to being a faculty physician-investigator at Massachusetts General Hospital and Harvard Medical School, the study PI is the Director of Research at BHCHP and is very familiar with the clinical environment and patient population served by the program.

Individuals must be homeless to enroll in services at BHCHP; however, some patients continue receiving care at the program after they are no longer homeless. An internal analysis of individuals seen at BHCHP in 2011 found that 16% of patients were housed, most without supportive services and therefore at potentially high risk for recurrent homelessness. Because our epidemiologic analyses demonstrating the dramatic lung cancer disparities experienced by BHCHP patients included both currently and formerly homeless individuals, and because of the often cyclic nature of homelessness, we will include both currently and formerly homeless individuals in the proposed study. We refer to this group collectively as "homeless-experienced." Owing to the Massachusetts system of universal health insurance, about 80% of BHCHP patients are insured by the state Medicaid program,[68] which covers LCS LDCT among eligible beneficiaries,[69] and a sizable proportion of those remaining are covered by Medicare alone. This will help ensure the availability of LCS LDCT for the vast majority of potential participants.

Participants will be recruited through a combination of 3 methods:

1) In-person screening of patients at BHCHP clinical sites: Study personnel will screen patients in-person at 6 high-volume BHCHP clinic sites: a) Pine Street Inn, a 470-bed shelter where BHCHP operates a medical clinic 7 days/week in the men's shelter and 6 days/week in the women's shelter; b) Southampton Shelter, a 400-bed men's shelter where BHCHP operates a medical clinic 7 days/week; c) Woods Mullen Shelter, a 200-bed women's shelter where BHCHP operates a medical clinic 5 days/week; d) New England Center and Home for Veterans, a 209-bed emergency and transitional shelter where BHCHP operates a medical clinic 5 days/week; e) St. Francis House, a daytime multiservice facility for homeless people where BHCHP operates a medical clinic 5 days/week; and f) Jean Yawkey Place, the BHCHP headquarters facility where medical, behavioral health, and dental clinics operate 5 days/week with an on-site pharmacy and medical respite unit. Our prior survey fieldwork with this population has given us extensive experience with tactfully deploying this proactive, in-person recruitment technique.[33-35] In light of the COVID-19 pandemic, BHCHP clinical sites and shelters are experiencing a significant decrease in patient volume and a lack of additional space to safely conduct in-person recruitment and data collection activities. Under these circumstances, study personnel will initially focus in-person screening and data collection efforts at Jean Yawkey Place, which has the highest volume of patients and the greatest degree of flexibility in physical space and layout. This will allow the study personnel to have an in-person presence at the clinic while practicing physical distancing and prioritizing the health and well-being of participants and study staff. If COVID-19 restrictions loosen and patient volume increases at shelter sites, we will expand our efforts to include the five additional sites, depending on COVID-19 public health guidelines and facility protocols.

2) Review of BHCHP electronic records: BHCHP has a comprehensive EHR system (Epic) that prompts providers to assess tobacco use at every clinical encounter, facilitating determination of smoking history and current smoking status. Individuals with a BHCHP PCP who have been seen in a BHCHP clinic in the past year and who were current or former smokers at the time of their most recent clinical encounter will be approached in person at BHCHP clinical sites if they have a scheduled appointment or contacted proactively via phone (if they have listed contact information) by a research coordinator and screened for eligibility to participate. The BHCHP Chief Medical Officer has approved this recruitment strategy on behalf of all primary care providers at BHCHP clinical sites, who will in turn be notified about our recruitment approach prior to the start of the study (letter of approval in Appendix J).

3) Referrals from BHCHP providers: We will advertise the study to all BHCHP providers and in all BHCHP clinical sites. Individuals deemed by a BHCHP provider to be potential candidates for LCS can be referred to the research coordinator, who will screen referred patients for eligibility to participate.

IV. SUBJECT ENROLLMENT

An integrated study schema depicting recruitment, enrollment, treatment assignment, and outcomes assessment has been uploaded as an attachment to this application.

A. COVID-19 symptom and exposure screening

BHCHP staff currently screen all patients for COVID-19 symptoms or a recent positive COVID-19 test upon entry to BHCHP clinics and facilities. BHCHP is also providing patients with facemasks before entering the building. Study personnel will re-screen participants using the same approach before conducting any research activities in an enclosed room. If BHCHP ceases their COVID-19 screening procedure, study personnel will use BHCHP's "COVID-19

Screening Tool for People Experiencing Homelessness" to screen all participants for current COVID-19 symptoms and past 14-day COVID-19 exposures (i.e., travel to high risk countries or states; unmasked exposure to a person with COVID-19). Study staff will wear appropriate personal protective equipment (PPE) per MGH guidelines and policies and remain 6 feet away from the patient. If patient endorses any COVID-19 symptoms or high-risk exposures, study personnel will immediately notify a clinic staff member of the presence of a symptomatic patient and will not engage in any study activities. Immediately after departure of the symptomatic patient, study personnel will disinfect surfaces that were within 6 feet of the symptomatic patient. If the participant reports not having symptoms, they can be directed to the study table/interview room. If the study coordinator makes initial contact with a participant over the phone, before scheduling an in-person visit, study personnel will screen the participant within 72 hours before the appointment. If participants report COVID-19 symptoms or exposures, study staff will request participants' permission to contact their BHCHP PCP via Epic messaging who can then advise the participant on next steps (e.g., testing and/or quarantine). If participants do not provide permission to study staff to contact their PCP, study staff will recommend that the participant seek care for their symptoms and/or exposure and offer to do the initial study procedures over the phone. If they are not interested in completing any further study procedures over the phone, study staff will call the participant again in 10 days to check-in and rescreen before attempting to schedule any study procedures in person. Participants will be advised to put on a facemask, regardless of symptoms, before coming in to meet with the research coordinator. Research personnel will instruct participants to notify them before arriving if they have fever or symptoms of COVID-19.

B. Methods of enrollment

Participants recruited through the above methods (section III.B) will be screened for eligibility either in-person or by phone. Prior to contacting participants, study staff will use the eligibility screener (Appendix A) to record information related to eligibility that was able to be collected during the prior review of prospective participants' BHCHP EHR (as referenced above).

Upon approaching the participants, study staff will use participant self-report to verify any information collected via their EHR and will collect the minimum amount of additional information necessary to assess and verify study eligibility. Eligible individuals identified through in-person screening will be offered the chance to enroll immediately. Those identified through phone screening will be asked to attend an in-person enrollment visit at one of clinic sites listed above. Participants will be asked to provide verbal informed consent before enrolling.

At least six months after initial enrollment, participants who were randomized to the patient navigation arm of the study will be contacted via phone or approached in person at their next BHCHP appointment to be offered the opportunity to participate in a 45-minute qualitative interview. Forty participants will be interviewed in total, with purposive sampling of participants who did (N=20) and participants who did not (N=20) attain the primary outcome of completing a lung screening CT scan within six months.

At approximately 6 months after the last patient participant is enrolled, providers who had at least one patient enrolled in the trial will be contacted via phone, email, or in person to be offered the opportunity to participate in a 45-minute qualitative interview. Ten participants will be interviewed in total, with purposive sampling to ensure that at least one provider who works at each of the targeted BHCHP clinic sites are interviewed.

C. Informed consent procedures

We will obtain verbal informed consent for this study. Eligible participants will be read a verbal consent script (Appendix B) and be presented with a study fact sheet (Appendix C) that will reinforce the consent script information. Depending on space availability, participant and research coordinator preferences, and whether in-person research activities are safe and permissible, informed consent will be obtained in one of the following ways: 1) “telephone booth” approach where the research coordinator would conduct the consent via Microsoft Teams or Enterprise Zoom on an encrypted laptop with the participant in a dedicated space on an encrypted study tablet; 2) meet in the clinic lobby, in a screened off area while maintaining at least 6 feet distance; 3) meet in an enclosed BHCHP clinic room while maintaining at least 6 feet distance if safe to do so; or 4) over the phone. The research coordinator will confirm participants’ understanding of the consent materials with basic knowledge questions about the information presented to them to ensure that they have the capacity to provide informed consent. Individuals who are able to correctly answer these questions will be asked to give their verbal consent to participant, which will be recorded by the research coordinator. Participants who provide informed consent over the phone will be given the option to receive a copy of the study fact sheet via picking up in-person at the JYP clinic, via mail if the participant has a mailing address, via secure email, or via email without send secure after obtaining participant’s permission.

40 participants who agree to participate in the post-intervention qualitative interviews will participate in a second verbal informed consent process at least 6 months after their initial enrollment. The research coordinator will read a separate verbal consent script (Appendix G) that reinforces the information presented in a fact sheet (Appendix F) that will be distributed to participants. The fact sheet will focus on the purpose, requirements, risks, and benefits of participating in the qualitative interview. The research coordinator will confirm participants’ understanding of the consent materials with basic knowledge questions about the information presented to them to ensure that they have the capacity to provide informed consent. Individuals who are able to correctly answer these questions will be asked to give their verbal consent to participant, which will be recorded by the research coordinator.

Additionally, we will obtain verbal consent to participate in qualitative interviews from 10 BHCHP PCPs who had at least 1 patient enrolled in the trial. Similar to the participants who participate in the interviews, the providers will be read a verbal consent script (Appendix I) that coincides with a fact sheet (Appendix H) that will be distributed to them. The research coordinator will record the providers’ verbal consent to participate in the interview.

We believe the verbal consent is justified for several reasons:

1) The experimental intervention, patient navigation, is a low-risk and widely-recommended behavioral intervention strategy with a sound evidence base in promoting cancer screening in vulnerable populations. Indeed, patient navigation has been endorsed by the ACS[64] and the National Cancer Institute[65] as a valuable approach to reducing cancer health disparities,[65] and is now part of routine care in some practice settings, including at MGH-affiliated health centers.

2) The outcome that the navigator is facilitating – obtaining LDCT for LCS among individuals at increased risk for lung cancer – is concordant with the guideline recommendations of the USPSTF,[20] ACS,[70] and multiple other professional groups.[71-74]

3) The study procedures present no more than minimal risk, as outlined below in section VII.

In order to ascertain the primary and secondary study outcomes of LDCT completion and LDCT completion with timely diagnostic follow-up (see section VII.B.), we will obtain written authorization from all enrolled participants to obtain their health/medical records for the 14 months following their trial enrollment date from the following Boston-area hospitals: Massachusetts General Hospital, Brigham & Women's Hospital, Boston Medical Center, Cambridge Health Alliance hospitals, and Tufts / New England Medical Center, in addition to any other hospital that the participant identifies as being one they typically use. Written authorization will be obtained after participants complete verbal informed consent using a HIPAA-compliant Release of Information (ROI) form which has been vetted by privacy officers at BHCHP, Boston Medical Center, and MGH (Appendix N). Participants who provide verbal consent during an in-person enrollment appointment will also complete the ROI form in person. Participants with a stable home address who complete the enrollment appointment remotely will be given the option to receive a pre-stamped envelope with the ROI form to have them sign and return back by mail to our MGH or BHCHP office.

If COVID-19 prompts further restrictions around in-person research activities at BHCHP, the research coordinator will transition to completing the verbal consent process over the phone or via the HIPAA compliant video conferencing platforms, Microsoft Teams or Enterprise Zoom depending on each participant's preference and access to technology. Based on feedback from the Boston Medical Center Compliance and Privacy Office, obtaining participant medical records for research purposes will continue to require written authorization regardless of current restrictions. Therefore, as noted previously, the research coordinator will offer participants with a stable home address the option to receive a pre-stamped envelope with the ROI form to sign and mail back. For participants who cannot receive a mailed ROI form, we will need to wait until in-person research restrictions are lifted. In this circumstance, once safe to do so, a research coordinator will either directly contact these research participants by phone or attempt to meet them in person at a clinic site to complete the form in person.

D. Treatment assignment

After providing informed consent and completing a baseline survey (see section V. A.), participants will be block-randomized in a 2:1 ratio to usual care with (N=200) or without (N=100) LCS navigation. The randomization scheme will be concealed from the study coordinator responsible for randomizing participants. Randomization will be stratified by smoking status (current/former), housing status (homeless/housed), prior discussion of lung cancer screening with their PCP, and primary clinical site where the patient sees their PCP (JYP vs. non-JYP sites) to ensure balance between study arms on these variables. We will stratify by smoking status (determined based on responses to the eligibility screener) because lung cancer risk perceptions and LCS motivations may differ between current and former smokers.[75,76] Current smoking will be defined as having smoked all or part of a cigarette in the past 30 days.[77] We will stratify by housing status based on responses to various items within the baseline survey because the navigation needs of formerly homeless people in housing may differ from those who are currently homeless. Current homelessness will be defined in the following ways: a) usually staying in an emergency shelter, transitional shelter, abandoned building, place of business, car or other vehicle, church or mission, hotel or motel, or anywhere outside during the past 7 days, b) usually housed in last 7 days but cannot stay in a residence for at least 14 days, c) usually institutionalized in the last 7 days if the admission was less than 90 days and they were homeless prior to admission, or d) usually institutionalized in the last 7 days if the admission was more than 90 days and they have no identified residence where they can stay at least 14 days upon discharge. These definitions are concordant with definitions of

homelessness used by the US Department of Health and Human Services (section 330 of the Public Health Service Act, 42 USC § 254b)[78] and by the US Department of Housing and Urban Development (S. 896 HEARTH Act of 2009).[79] We will stratify based on prior discussion of lung cancer screening with their PCP (defined by an item within the baseline survey) due to the fact that these participants are farther along in the lung cancer screening process. Finally, we will stratify by the patient's primary care clinical site since clinics may differ in their proximity to facilities that offer LCS or in their practice patterns around recommending and ordering LDCT for LCS. We will create 2 clinical site strata: 1 for JYP and one encompassing all of the 5 non-JYP study sites described above.

The 2 study arms are as follows:

Arm 1: Usual care ***without*** patient navigation (N=100). Participants assigned to this arm will be given basic educational materials on general lung health (Appendix K) and referred back to their PCP to discuss whether to pursue lung cancer screening per usual practice.

Arm 2: Usual care ***with*** patient navigation (N=200). Participants assigned to this arm will be informed about LCS, provided educational materials on LCS and patient navigation (Appendix L), and offered access to an LCS navigator. The navigator's principal role is to guide participants through the LCS process. The navigator's secondary role is to provide a brief tobacco intervention for participants who currently smoke. These roles are described below:

1) LCS navigation: The navigator will introduce the concept of LCS and assist participants in scheduling and attending a shared decision-making (SDM) visit with their PCP to discuss the test in greater depth. The navigator will proactively reach out to PCPs about this via electronic messaging through the BHCHP EHR and make him/herself available to assist in the LCS process. If a PCP and a participant jointly decide to pursue LCS, the navigator will assist with booking the LDCT appointment at a local facility that offers this test and interprets it according to the Lung-RADS framework.[80] BHCHP maintains strong clinical ties with Massachusetts General Hospital, Boston Medical Center, and several other area hospitals, enhancing the feasibility of referring patients to these facilities for LDCT. The navigator will work collaboratively with participants to reduce the barriers to LCS completion while building participants' self-efficacy. If a participant has difficulty obtaining LDCT because of lack of insurance, the navigator will connect the participant with a BHCHP benefits specialist to enroll in health insurance under the state's universal coverage system. Following LDCT, the navigator will coordinate a follow-up appointment with a participant's PCP to discuss the results and arrange any necessary follow-up testing. Throughout this process, the navigator will provide reminder phone calls about upcoming appointments, arrange for transportation assistance and other enabling services when needed, and reinforce the content and concepts covered in the LCS SDM visit through in-person or phone contact. Data from our survey of homeless smokers at BHCHP indicate that over 70% have mobile phones, which will facilitate communication between the navigator and study participants. In addition to documenting their encounters and activities with patients for research data collection purposes (see section VI.A.), the navigator will document within the patients' BHCHP EHR for their care teams to reference as needed.

The navigator will work with each participant for up to 6 months (26 weeks) to receive the initial LCS LDCT scan. If a participant receives their initial LCS LDCT scan within the 6-month (26-week) timeframe and requires follow-up diagnostic testing of abnormal results anytime sooner than the usual LCS LDCT screening interval of 12 months, the navigator will continue to work with the participant until the follow-up diagnostic testing is completed or until the participant no longer wishes to be navigated.

2) Brief tobacco intervention: The navigator will provide a brief tobacco intervention and distribute educational handouts on smoking cessation (on an ad hoc basis; Appendix M) consistent with the "5 A's" approach.[81] Within this framework, the navigator will Ask participants if they currently smoke cigarettes and Advise current smokers to quit, emphasizing the connection between smoking and lung cancer and the importance of quitting smoking to lower their lung cancer risk. The navigator will then Assess current smokers' willingness to quit and Assist them in accessing no-cost or low-cost smoking cessation resources, including:

a) BHCHP PCPs, to discuss smoking cessation medications (e.g. nicotine replacement products, bupropion, or varenicline), which are covered by the state Medicaid program with a $1-3 copayment at retail pharmacies or at no charge through the BHCHP pharmacy. Over 80% of BHCHP patients are insured by the Massachusetts Medicaid program under the state's universal health insurance system.[68]

b) BHCHP tobacco counselors, who are formally trained in tobacco counseling and conduct weekly support groups at 4 BHCHP venues and individual counseling sessions at 5 BHCHP venues, all free of charge.

c) The Massachusetts Smokers' Helpline, a free and confidential phone counseling service, for participants with mobile phones. Participants who call the Massachusetts Smokers' Helpline may receive up to 4 weeks of nicotine replacement therapy (patch, gum, or mini-lozenge) at no cost.

The navigator will then arrange a time to follow-up with current smokers either by phone or in person to discuss their progress on smoking and their engagement in available services. For participants who undergo LDCT, the navigator will conduct a follow-up brief intervention to head off false reassurance about smoking if the findings are negative. Brief tobacco interventions are an evidence-based, effective treatment approach to promoting smoking cessation.[81,82] The brief intervention strategy described above acknowledges the centrality of smoking cessation in reducing lung cancer deaths, capitalizes on the "teachable moment" afforded by the LCS process,[83] satisfies the CMS requirements for LCS LDCT approval,[21] and complements our pragmatic trial design by working within existing tobacco cessation resources available to the target population.

V. Study Procedures

A. Study visits and measurements

Participants who are screened and deemed eligible to participate, whether in person or over the phone, will be asked to complete a research visit during which enrollment, collection of baseline data, and randomization to study arm will occur. Participants will meet with the research coordinator in person at a BHCHP clinic site to complete these activities or complete these activities remotely. After verbal consent and the written ROI form is completed, a study coordinator will meet with the participant to administer a 30-minute baseline survey. In a similar fashion to the verbal consent, the survey can be completed in one of the following ways depending on current pandemic status and/or participant/staff preferences: a) "telephone booth" approach where the research coordinator would conduct the survey on an encrypted laptop via Microsoft Teams or Enterprise Zoom with the participant in a dedicated space with an encrypted study tablet; b) meet in the clinic lobby in a screened off area while maintaining at least 6 feet distance; c) meet in an enclosed BHCHP clinic room while maintaining at least 6 feet distance of safe to do so; or d) remotely, either over the phone if the participant has adequate access to

their own phone and phone plan, or over secure video conferencing (Microsoft Teams or Enterprise Zoom) if the participant has adequate access to the necessary technology.

Participants will receive a one-time $25 gift card upon completion of the baseline survey in recognition of their time and effort. If participants complete the baseline survey over the phone, research staff will offer participants the chance to pick up the study gift card in-person at JYP or via mail if the participant has a mailing address.

Participants with a stable home address who complete enrollment and the baseline survey over the phone and who are not willing to travel to JYP to sign the ROI in person, will be given the option to receive a pre-stamped envelope with the ROI form to have them sign and mail back to our MGH or BHCHP office. Participants will be mailed the $25 gift card once the research coordinator receives a signed ROI.

Arm 2 participants will additionally have a series of ad hoc meetings with the LCS navigator as part of the intervention. These meetings may occur in person or over the phone. The navigator will be asked to log the date, time, and nature of activities carried out during these navigation sessions/meetings.

Due to the nature of the primary outcome, which will be ascertained via medical record review, no formal research follow-up visit is necessary. The screening, baseline, and outcome variables for the study are detailed in section VI.A.

40 participants in Arm 1 will participate in post-intervention interviews and be stratified according to whether they did (N=20) or did not (N=20) attain the primary outcome of completing a lung screening CAT scan within six months (26 weeks) of initial enrollment. Participants who agree to participate will go through a separate verbal consent process as illustrated previously. Interviews will occur after at least 6 months of exposure to the navigation intervention. The interviews will last up to 45 minutes and be audio recorded. The selection of qualitative participants following ascertainment of the primary quantitative outcome avoids introducing a treatment effect from the interviews while remaining temporally close enough to the LCS process to facilitate accurate recall. These 40 participants will receive a one-time $25 gift card upon completion of the interview in recognition of their time and effort.

Additionally, we will interview 10 BHCHP PCPs among those who had at least 1 patient enrolled in the trial. Providers who agree to participate will provide verbal consent as illustrated previously. Interviews will last up to 45 minutes and be audio recorded. Interviews will occur following completion of the trial (at least 6 months after the last patient participant is enrolled) to avoid influencing provider practice around LCS ordering and follow-up. The 10 PCPs will receive a one-time $25 gift card upon completion of the interview in recognition of their time and effort.

Study personnel will follow various safety precautions to prevent COVID-19 transmission. For patients that are screened for eligibility over the phone and interested in scheduling an in-person study visit, study personnel will administer a screening instrument for COVID-19 symptoms and recent positive COVID-19 tests within 72 hours prior to the scheduled in-person study visit. Prior to entering BHCHP clinic buildings, per BHCHP policy, all patients are already required to wear a face mask and be screened for COVID-19 symptoms and recent positive COVID-19 tests at the door. If BHCHP ceases these screening procedures, study personnel will implement the same screening measures at the start of any in-person visit and will continue to require participants to wear a face mask while engaging with study staff. If a patient endorses

any COVID-19 symptoms, study personnel will immediately cease study activities and notify a clinic staff of the presence of a symptomatic patient.

If rising COVID-19 rates necessitate temporary restrictions on all in-person study activities at BHCHP facilities, we will transition to remotely conducting as many study activities as possible until the restrictions are lifted. Study personnel will be able to screen, perform verbal informed consent, conduct the baseline survey, perform qualitative interviews, and provide navigation services over the phone or by the HIPAA-compliant video conferencing platforms Microsoft Teams or Enterprise Zoom depending on patient preference and access to technology (i.e., owning a device with adequate minutes or video conferencing capabilities). Study personnel will always follow the MGB requirements for using Teams or Zoom with no study visits or activities being audio- or video-recorded within the Teams or Zoom platform. Microsoft Teams will be the preferred platform to use for video call study visits, but in the event of technical difficulties, study personnel will use Enterprise Zoom as a backup platform.

B. Drugs

While the LCS navigator will assist participants who currently smoke with accessing smoking cessation resources, neither the navigator nor study staff will provide participants with medications. Participants in both arms may choose to seek smoking cessation medications through their BHCHP PCP or other health care provider.

C. Devices

No devices will be used in this study.

D. Procedures

There are no procedures or surgical interventions in this study. Participants may elect to undergo LCS with LDCT after consultation with their PCP (and with the LCS navigator, for those in Arm 2), but the ordering and oversight of LDCTs will be handled by BHCHP PCPs as per usual practice for participants in both study arms.

E. Data Collection

We will collect all study data electronically using Partners-approved laptops or tablet devices with full disk encryption. Participant data will be recorded, saved, and managed using the Partners-hosted Research Electronic Data Capture (REDCap) program with data collection forms custom-designed for this study. We will also use BHCHP EHR to collect information from participants' medical records that relates to their general health history, any charted mental health problems, and any other information that directly relates to lung cancer screening and smoking. The specific data elements collected at each study visit are detailed below in section VI.A.

VI. BIOSTATISTICAL ANALYSIS

A. Specific data variables

1) Screening variables: Screening variables will include self-reported measures related to the inclusion and exclusion criteria for study participation. These will include the following variables,

assessed as shown in Appendix A:
- First and last name
- Date of birth
- Age
- Preferred language
- BHCHP PCP
- Preferred BHCHP clinic site
- History of homelessness
- History or symptoms of lung cancer
- Recent non-lung cancer or cancer treatment
- Home oxygen use
- Cigarette smoking history

2) Contact form variables: Enrolled participants will be asked to complete a contact information form. This information will be valuable for following up with participants who cannot complete the baseline survey on the same day as enrollment, as well as for the patient navigator to aid with patient outreach and EHR documentation. This form will include the following variables, assessed as shown in Appendix O:
- BHCHP Epic MRN (entered by the research coordinator from patient's EHR)
- Last 4 digits of social security number
- Phone number (if applicable)
- Address/location of usual nighttime residence
- Alternative contact (who may know of participant's whereabouts)
- Contact in the case of an emergency
- Participant photograph (with participant permission) to facilitate subsequent contact with patient navigator

3) Baseline variables: Enrolled participants will be asked to complete a baseline assessment (Appendix D) of variables mapping to core domains of the Health Belief Model in addition to other key factors that could impact one or more dimensions of the Health Belief Model, as displayed in Table 2.

4) BHCHP electronic health record variables: The following information will be collected from the review of patients' BHCHP electronic health records:
- History of care with BHCHP and BHCHP PCP prior to study enrollment
- Comorbid health conditions.
- Documentation of a shared decision making (SDM) appointment with PCP to discuss lung cancer screening.
- Documentation of referrals for lung CT scans.
- Documentation of follow-up appointments with PCP that address results of lung cancer screening.
- Documented smoking status (and any changes in smoking status during study enrollment).
- Documented prescriptions to any smoking cessation medications
- Documented insurance status and insurance type.

Table 2. Baseline measures of study participants corresponding to Health Belief Model (HBM) domains and other key factors.

**HBM domains**	**Measures**
**Sociodemographic factors**	Age; sex; race/ethnicity; education; (all based on items from the 2003 HCH User Survey[23,84] and our prior survey work with homeless individuals[33,34]); homelessness onset, episodes, and duration[1]; current living situation
**Perceived lung cancer susceptibility/severity**	Perceived risk of lung cancer and smoking, perceived severity of lung cancer, worry about lung cancer, (all developed by Dr. Park for NLST)[76,85];
**Cues for LCS**	Prior LCS recommendation by health care provider; prior exposure to LCS brochures, flyers, posters, or advertisements
**Perceived LCS benefits/barriers**	Lung cancer screening knowledge[86]; perceived benefits of quitting smoking[76,85], comfort navigating hospital/health system; health insurance status[23,84]; competing priorities for meeting subsistence needs[22]
**Self-efficacy for LCS**	General self-efficacy scale[87,88]; self-efficacy for completing LDCT
**Other key factors**	**Measures**
**Tobacco use**	Duration of smoking; past quit attempts; current cigarettes/day; alternative tobacco use[35,89,90], Fagerstrom Test for Nicotine Dependence[91]; stage of change[92,93]; Contemplation Ladder[94]
**COVID-19**	Comfort navigating hospital/attending medical appointment during COVID-19 outbreak[95-97]; Previous history of COVID-19 And Lung Infection/Pneumonia
**Comorbid conditions**	Alcohol Use Disorders Identification Test-Concise (AUDIT-C)[98,99];! Drug Abuse Screening Test (DAST-10)[100,101], and K6 screen for psychological distress[102,103]; single-item general health status (SF-1)[104,105]

3) Navigation log: The navigator will complete a log (Appendix E) capturing the following information each time he/she interacts with a participant in Arm 2.
- Name of study staff who completes navigation activity
- Date and time
- Participant ID number
- Who was contacted for navigation activity
- Contact modality for navigation activity
- Tasks/activities undertaken during encounter

4) Qualitative interview data elements:

Participant interviews: Participants will be asked to articulate their thought process in considering whether to pursue LCS and to identify the factors that most shaped their decision, including logistical, physical, or emotional barriers and facilitators. Based on our Health Belief Model framework (Table 2), we will prompt participants to consider the impact of the navigator on a) altering their perceived susceptibility to lung cancer, b) providing them with cues or reminders to pursue LCS, c) enhancing their self-efficacy for pursuing LCS, and d) helping them to overcome barriers to completing LCS. Among participants who undergo LDCT, we will

assess their perspectives on the ease of the process, its impact on lung cancer worry, perceived lung cancer risk, and smoking behavior, and the need for any follow-up testing.

PCP interviews: These interviews will elicit PCP perspectives on the challenges of implementing LCS guidelines in primary care practice for a vulnerable population and probe the ways in which the navigator did or did not help to reduce these challenges. We will also seek feedback from PCPs on ways to strengthen the integration of patient navigators into clinical care teams.

B. Study Endpoints

1) Primary outcome: The primary outcome is receipt of LDCT for LCS at 6 months (26 weeks). This will be based on radiology records verifying that a chest CT was performed for LCS and interpreted according to the Lung-RADS framework.[80] To ascertain this outcome, study staff will ask participants at the time of enrollment to provide written consent to obtain radiology records from Boston-area facilities that perform LDCT for LCS, as described above. At 6 months (26 weeks) after enrollment, radiology records will be requested/obtained from these facilities. Most Boston-area hospital records are accessible through Care Everywhere in the BHCHP version of Epic, supporting the feasibility of gathering this information in an efficient manner. This approach does not rely on participant self-report and therefore does not require in-person or phone follow-up to ascertain. For those verified to have undergone LDCT for LCS within the 6 month (26-week) timeframe, the Lung-RADS result along with any abnormal or incidental findings will be recorded.

2) Secondary outcome: Increasing LDCT completion will only reduce lung cancer mortality if individuals with abnormal results can undergo evaluation and treatment of early stage lung cancer in a timely fashion. In the setting of homelessness, a valid concern is whether participants with abnormal LDCT results can be recalled for diagnostic follow-up in a timely manner. To address this concern, our secondary outcome is receipt of LDCT with timely diagnostic follow-up of any abnormal results. To satisfy this composite outcome, participants must achieve the primary outcome (LDCT at 6 months (26 weeks) and, if the result is abnormal, also obtain the next recommended follow-up test within 1 month (4 weeks) of the advised timeframe, based on the Lung-RADS framework.[80] Lung-RADS category descriptions, recommended follow-up, associated probabilities of malignancy, and estimated general population prevalences are shown in Table 3.[104] As described above, we will obtain radiology records for participants who underwent LDCT for LCS to document the findings of the study, determine the Lung-RADS category associated with those findings, and ascertain the recommended diagnostic follow-up plan. Participants with Lung-RADS 1-2 findings do not require specific diagnostic action, but rather may continue with annual screening at 12 months. These individuals will automatically satisfy the secondary outcome. In contrast, Lung-RADS 3-4 findings signal an increased risk for malignancy and merit sooner-than-annual follow-up testing. These individuals will satisfy the secondary outcome only if they obtain the next recommended diagnostic study within 1 month (4 weeks) of the recommended timeframe. For example, if a participant has a Lung-RADS 3 finding for which

Table 3. Lung-RADS categories.

Category	Description	Management	Malignancy probability	Gen. pop. prevalence
1	Negative	Repeat LDCT 12 mos	<1%	90%
2	Benign			
3	Probably benign	Repeat LDCT 6 mos	1-2%	5%
4A	Suspicious	Repeat LDCT 3 mos +/- PET/CT	5-15%	2%
4B/X		Chest CT, PET/CT, +/- biopsy	>15%	2%

*Adapted from the American College of Radiology

follow-up LDCT in 6 months is recommended, we will assess whether this follow-up test occurred within 7 months (30 weeks) of the initial LDCT. If a participant has a Lung-RADS 4B/X finding suggesting a high suspicion for malignancy, we will assess whether the recommended follow-up testing occurred within 1 month (4 weeks) of the initial LDCT. The offer of delayed navigation to control arm participants will not interfere with this outcome because a) delayed navigation will be offered only to those who did not undergo an initial LCS LDCT within 6 months (26 weeks) (i.e. the primary outcome), and b) attaining the primary outcome is necessary for satisfying the secondary outcome.

3) Other outcomes: We will examine time to receipt of LDCT for LCS. Process outcomes include acceptance of navigation when offered, number of contacts with the navigator, and attendance of a LCS SDM visit with a BHCHP PCP. Tobacco-related outcomes include receipt of a prescription for any smoking cessation medication and changes in clinically-recorded smoking status (i.e. quitting among current smokers or relapsing among former smokers), both ascertained by BHCHP medical record review. We will track the frequency and types of abnormal findings detected on LCS LDCT, any additional diagnostic testing or therapeutic changes pursued in response to these findings, and the percentage of participants diagnosed with lung cancer (based on radiology and BHCHP records review). We will also track diagnostic chest CT scans obtained for reasons other than LCS.

C. Statistical Methods

Quantitative analysis: LDCT completion at 6 months (26 weeks) (1° outcome) and LDCT completion with timely diagnostic follow-up (2°outcome) are both binary outcomes. In the principal analysis, we will assess the difference between the navigation and usual care arms on attainment of these outcomes using the Chi-square test. We will also use logistic regression models to examine the associations between treatment condition and outcomes including clinical site and other known predictors of outcomes to increase the precision of effect estimates. Additional analyses will examine time to receipt of LDCT for LCS between study arms using time-to-event methods (log-rank tests and Cox regression models). Although we expect to be underpowered to detect significant interactions, we will examine whether the effect of navigation varies by smoking status, housing status, or clinical site in an exploratory analysis.

Qualitative analysis: Qualitative interviews will be audiotaped and transcribed. We will use inductive content analysis methods to identify major and minor themes, and we will use deductive methods to strengthen, refine, and enhance understanding of identified themes.[105] Qualitative data coding will be performed by 2 trained study staff members using NVivo 11 software. The data coders will meet regularly with Drs. Baggett and Park to discuss the structural thematic framework, categories, and coding plan. We will resolve discrepancies through discussion and comparison to raw data. Coding will continue iteratively until a high level of reliability (Kappa>0.80) is established.

Integration with quantitative data: Based on a mixed methods approach,[106] we will use the qualitative interview data to interpret and explain the quantitative observations from the trial. This will aid in better understanding why overall LDCT completion was higher or lower than expected and why the navigation intervention did or did not improve these outcomes. Following

Table 4. Proposed integration of quantitative and qualitative data.

Health Belief Model domains	Measurement (method)	LCS LDCT at 6 months	
		Yes (N=20)	No (N=20)

Perceived susceptibility / severity	Baseline survey (quan)	Lung cancer risk perception, severity, and worry scores
	Post-intervention interview (qual)	Quotes/themes about navigator impact on perceived susceptibility/severity
Cues	Baseline survey (quan)	Past provider recommendation for LCS, exposure to LCS information
	Post-intervention interview (qual)	Quotes/themes about navigator provision of cues for LCS
Self-efficacy	Baseline survey (quan)	General and LCS-specific self-efficacy scores
	Post-intervention interview (qual)	Quotes/themes about navigator impact on self-efficacy
Perceived benefits / barriers	Baseline survey (quan)	LCS knowledge, competing subsistence difficulty scores
	Post-intervention interview (qual)	Quotes/themes about navigator assistance with barriers

from our stratified sampling scheme, we will integrate the quantitative and qualitative data by assessing for thematic differences in the interview content of participants who did (N=20) and did not (N=20) attain the primary outcome. We will focus in particular on comparing and contrasting these two groups' views on the utility of the navigator with respect to the Health Belief Model domains of perceived susceptibility, cues, self-efficacy, and barriers, as described above. We will additionally incorporate the quantitative baseline measures tapping these Health Belief Model domains into integrated analyses. Table 4 illustrates the way in which these quantitative and qualitative data elements will be integrated and presented in a blended fashion.

D. Power Analysis

The sample size justification is based on the primary outcome of LCS LDCT completion at 6 months (26 weeks). Internal BHCHP data suggests a presently negligible baseline rate of LCS LDCT. Allowing for improvement in this rate as LCS comes into wider practice, we will assume that 5% of usual care (Arm 1) participants will obtain an LDCT within 6 months (26 weeks). In our judgment, increasing this completion rate to 15% would represent a clinically meaningful change. We will have >80% power to detect such a difference with a sample size of 200 in the navigation arm (Arm 2) and 100 in the usual care arm (Arm 1).

VII. RISKS AND DISCOMFORTS

The potential risks and discomforts associated with this study are detailed below. The methods for mitigating these risks and monitoring the safety of participants are described in section IX.b.

A. Complications of surgical / non-surgical procedures

Not applicable.

B. Drug side effects and toxicities

Study staff will not be offering any drugs or over-the-counter products to participants.

C. Device complications

Not applicable.

D. Psychosocial (non-medical) risks

Potential risks to participants include psychological distress arising from the possibility of false-positive CT findings leading to additional diagnostic studies or procedures. Psychological and/or

physical distress may also be brought about by discussing smoking and/or changing smoking behavior. Finally, because we will collect and record identifying information (e.g. name, date of birth, and social security number), there is a risk to privacy if the data are compromised. Section IX (Monitoring and Quality Assurance) describes our methods for safeguarding the data and privacy of participants. The potential risks of this study are reasonable in relation to the potential benefits of reducing the risk of dying from lung cancer.

E. Radiation risks

There is a slight physical risk associated with exposure to radiation during CT scanning, although the "low-dose" nature of the test is designed to help mitigate this risk. For this reason, LDCT LCS for individuals at increased risk of lung cancer is recommended by a variety of professional societies since the benefits of screening for early stage cancer vastly outweigh the risks presented by small radiation exposures.

VIII. POTENTIAL BENEFITS

A. Potential benefits to participating individuals

Potential benefits for participants include the opportunity to receive assistance in completing LCS, which has been associated with a 20% reduction in lung cancer mortality. Additionally, current smokers will be informed of and referred to various resources to help them quit smoking, which may confer numerous health and financial benefits.

B. Potential benefits to society

Considerable knowledge could be gained from this study. To our knowledge, this will be the first study to focus on LCS among homeless people and the first randomized trial of any cancer screening intervention in this population. By focusing on an understudied topic area pertaining to a common cause of death among homeless people, our study fills an important gap in the literature and could serve as a blueprint for improving cancer screening and outcomes in homeless health care settings, particularly in the 295 HCH programs that serve 890,000 people annually in the US.[107]

IX. MONITORING AND QUALITY ASSURANCE

A. Data monitoring

All data will be collected using password-protected laptops or tablets with full disk encryption. We will use the Partners-hosted Research Electronic Data Capture (REDCap) application for data collection and management. REDCap is a secure, HIPAA compliant web-based application hosted by Partners HealthCare Research Computing, Enterprise Research Infrastructure & Services (ERIS). Because a Partners username is required for logging in to REDCap, all activity on study documents is electronically logged and therefore traceable. All data will be collected according to a standardized protocol preprogrammed into REDCap, which has built-in functions providing real-time data entry validation to help ensure accuracy and completeness. Each data collection form will have instructions or prompts for collecting the required data elements, and the response fields for each item will have appropriate ranges and formats to ensure that the data is entered in a valid way. Participants who agree to be photographed will have their photo

taken using the same device on which the rest of the data is collected (encrypted laptop or tablet). The photograph will be uploaded immediately to the REDCap database and the original photograph file will be permanently deleted from the device.

The PI will review all study data weekly to monitor its integrity. Additionally, the PI, biostatistician, and/or data analyst will download the study data on a weekly basis from the Partners REDCap server and back it up to the PI's SFA on the Partners network, which is protected by the Partners IS firewall, backed up nightly, and accessible only to authorized study staff. Only the minimum necessary number of study staff will have access to this data. Data analyses will be conducted by authorized study staff on the data files residing on the PI's Partners SFA. The data will not be transferred to investigators outside the Partners system.

The qualitative interviews conducted post-intervention will be recorded by a microphone connected directly to a password-protected, Partners-approved laptop or tablet with full-disk encryption. Audio files will be backed up to the PI's SFA described above and then deleted from the portable device.

If study activities are administered to participants using Enterprise Zoom or Microsoft Teams, personnel will always follow the MGB requirements for using video conferencing for research activities with no study visits or activities being audio- or video-recorded, with the exception of qualitative interviews, which will be audio-recorded with an encrypted laptop or tablet microphone (and not via the audio-recording capabilities within the Zoom or Teams platform) as described above. Study personnel will always follow the Principles of Privacy and share the minimum necessary amount of information.

B. Safety monitoring

Because of the narrow range of risks of the proposed study, there will not be a Data and Safety Monitoring Board for this trial. Instead, the safety of participants will be monitored by the PI on a continual basis. Navigators will stay in close contact with BHCHP PCPs and clinical staff to ensure the safety and well-being of all participants.

To minimize the risk to privacy, we will adhere to the data management procedures described above to help ensure the safety and integrity of data collected about study participants. All identifying information will be saved securely within the REDCap database. Paper ROI forms will be scanned into the PI's SFA and paper copies retained in a locked cabinet in the PI's office. Only the minimum necessary number of study staff will have access to this data. At the conclusion of the study, personal identifiers will be removed from the dataset and all analyses will occur in a de-identified fashion.

To help ensure the safety of participants, adverse events monitoring will be handled in the manner described below.

C. Outcomes monitoring

Because of the low-risk nature of the intervention, we will not conduct a formal interim analysis of the data nor define rules for early stopping of the trial.

D. Adverse event reporting guidelines

We will adhere to the Partners policy statement on “Reporting Unanticipated Problems including Adverse Events.” Consistent with this policy, an adverse event will be defined as “any untoward or unfavorable medical occurrence in a human subject, including any abnormal sign, symptom, or disease, temporally associated with the subject’s participation in the research, whether or not considered related to the subject’s participation in the research.” The PI and his designated study staff will be responsible for the monitoring of adverse events in study participants.

Adverse events reported to the PI or other study staff will be documented in an adverse event report containing a description of the event, the date and time of onset, the date and time of resolution, the expectedness of the event, the relationship to the study, the seriousness of the event, and the action taken in response to the event. An adverse event will be deemed unexpected if the nature, severity, or frequency of the event is not consistent with a known or foreseeable risk given the research procedures, the characteristics of the subject population, or with an expected progression of an underlying condition. The following attribution scale will be used to describe the relationship of the adverse event to the study protocol: unrelated to the protocol, or possibly, probably, or definitely related to the protocol. A serious adverse event will be defined as an adverse event that results in death, is life-threatening, requires hospitalization, causes persistent or significant disability, or requires medical or surgical intervention.

A summary safety report detailing all adverse events and their handling will be included in annual study progress reports to the Partners IRB and the American Cancer Society. Unexpected adverse events of any severity that are at least possibly related to the research and suggest the research puts participants at increased harm will be reported to the Partners IRB within 5 working days or 7 calendar days. Serious adverse events that are unexpected and at least possibly related to the research will be assumed to suggest that the research puts participants at increased harm and will be reported to the Partners IRB within the same time frame. Any action resulting in a temporary or permanent suspension of this study will be reported to the American Cancer Society’s program official. Adverse event reports and annual safety summaries will be documented by study ID number; personal identifiers will not be included in these reports.

E. Protocol adherence

1) Research staff: The study coordinators will be trained on the data collection protocols described above. Their understanding of these protocols will be assessed by asking them to demonstrate competency in using the REDCap data collection forms.

2) LCS navigator: To ensure adherence to the navigation intervention strategy, the navigator will receive in-depth training on patient navigation and focused training on tobacco cessation counseling.

a) Navigation training: The navigator will receive training in the core skills of patient navigation, including establishing rapport with patients while maintaining appropriate professional boundaries, motivational interviewing, performing an initial assessment with patients to explore barriers to LCS, working with patients to overcome these barriers, educating patients about the LCS process, introducing the concept of shared decision-making (SDM) around LCS, and empowering participants to discuss the benefits and risks of LCS with their PCP. Training will also encompass an overview of lung cancer, the rationale behind LCS, and the practical steps involved in completing LDCT so that the navigator can better counsel patients on what to expect from the LCS process. Additionally, the navigator will be trained in identifying and managing logistical hurdles to completing LCS, such as insurance coverage

lapses that may need to be remedied before LDCT is feasible. To facilitate timely follow-up of abnormal LDCT results, the navigator will received focused education on the American College of Radiology (ACR) Lung Imaging Reporting and Data System (Lung-RADS) framework.[80] Lung-RADS is a quality assurance tool designed to standardize LCS LDCT reporting and management recommendations, reduce confusion in LCS LDCT interpretations, and facilitate outcome monitoring.[80] The navigator training will be based on the protocol developed for Dr. Percac-Lima 's successful RCT of patient navigation for LCS among community health center patients (see section E). Dr. Percac-Lima, who has extensive experience in navigator training, will provide key components of this training and facilitate role playing sessions to reinforce skills. To the greatest extent possible, experienced navigators will be included in the training process. Depending upon prior experience, the navigator trainee will spend up to 2 weeks shadowing and observing an experienced navigator in the field prior to the start of the study. Refresher training and review of specific case scenarios will occur every 3 months throughout the study to ensure that the navigator is adhering faithfully to the intended roles and responsibilities.

b) Tobacco counseling training: The goal of the tobacco training component is to equip the navigator with the skills to provide a brief tobacco intervention. To achieve this goal, the navigator will complete an online 9-module course titled "Basic Skills for Working with Smokers," sponsored by the University of Massachusetts Center for Tobacco Treatment Research & Training.[108] This course provides an introduction to the theory and practice of working with smokers in a health care setting. The navigator will additionally receive 4 hours of case-based teaching from Dr. Baggett on practical approaches to working with homeless smokers, followed by 6 hours of observing a certified Tobacco Treatment Specialist interact with and counsel smokers about tobacco cessation.

The navigator's adherence to their intended duties will be assessed during weekly team meetings and through periodic audit of their navigation encounter logs.

X. REFERENCES

1. Burt MR, Aron LY, Douglas T, et al. Homelessness: Programs and the People They Serve: Findings of the National Survey of Homeless Assistance Providers and Clients: Technical Report. In. Washington, D.C.: U.S. Department of Housing and Urban Development, Office of Policy Development and Research; 1999.
2. Baggett TP, Chang Y, Porneala BC, Bharel M, Singer DE, Rigotti NA. Disparities in Cancer Incidence, Stage, and Mortality at Boston Health Care for the Homeless Program. *Am J Prev Med.* 2015;49(5):694-702.
3. Lamont DW, Toal FM, Crawford M. Socioeconomic deprivation and health in Glasgow and the west of Scotland--a study of cancer incidence among male residents of hostels for the single homeless. *J Epidemiol Community Health.* 1997;51(6):668-671.
4. Hwang SW, Wilkins R, Tjepkema M, O'Campo PJ, Dunn JR. Mortality among residents of shelters, rooming houses, and hotels in Canada: 11 year follow-up study. *BMJ.* 2009;339:b4036.
5. Baggett TP, Hwang SW, O'Connell JJ, et al. Mortality among homeless adults in Boston: shifts in causes of death over a 15-year period. *JAMA Intern Med.* 2013;173(3):189-195.
6. Baggett TP, Chang Y, Singer DE, et al. Tobacco-, alcohol-, and drug-attributable deaths and their contribution to mortality disparities in a cohort of homeless adults in Boston. *Am J Public Health.* 2015;105(6):1189-1197.
7. Aberle DR, Adams AM, Berg CD, et al. Reduced lung-cancer mortality with low-dose computed tomographic screening. *N Engl J Med.* 2011;365(5):395-409.
8. Chau S, Chin M, Chang J, et al. Cancer risk behaviors and screening rates among homeless adults in Los Angeles County. *Cancer Epidemiol Biomarkers Prev.* 2002;11(5):431-438.
9. Lebrun-Harris LA, Baggett TP, Jenkins DM, et al. Health status and health care experiences among homeless patients in federally supported health centers: findings from the 2009 patient survey. *Health services research.* 2013;48(3):992-1017.
10. Asgary R, Garland V, Jakubowski A, Sckell B. Colorectal cancer screening among the homeless population of New York City shelter-based clinics. *Am J Public Health.* 2014;104(7):1307-1313.
11. Asgary R, Garland V, Sckell B. Breast Cancer Screening Among Homeless Women of New York City Shelter-Based Clinics. *Womens Health Issues.* 2014.
12. Long HL, Tulsky JP, Chambers DB, et al. Cancer screening in homeless women: attitudes and behaviors. *J Health Care Poor Underserved.* 1998;9(3):276-292.
13. Baggett TP, Rigotti NA. Cigarette smoking and advice to quit in a national sample of homeless adults. *American Journal of Preventive Medicine.* 2010;39(2):164-172.
14. Connor SE, Cook RL, Herbert MI, Neal SM, Williams JT. Smoking cessation in a homeless population: there is a will, but is there a way? *Journal of General Internal Medicine.* 2002;17(5):369-372.
15. Snyder LD, Eisner MD. Obstructive lung disease among the urban homeless. *Chest.* 2004;125(5):1719-1725.
16. Szerlip MI, Szerlip HM. Identification of cardiovascular risk factors in homeless adults. *Am J Med Sci.* 2002;324(5):243-246.
17. Tsai J, Rosenheck RA. Smoking Among Chronically Homeless Adults: Prevalence and Correlates. *Psychiatr Serv.* 2012;63(6):569-576.
18. Torchalla I, Strehlau V, Okoli CT, Li K, Schuetz C, Krausz M. Smoking and predictors of nicotine dependence in a homeless population. *Nicotine Tob Res.* 2011;13(10):934-942.
19. Hahn JA, Kushel MB, Bangsberg DR, Riley E, Moss AR. BRIEF REPORT: the aging of the homeless population: fourteen-year trends in San Francisco. *J Gen Intern Med.* 2006;21(7):775-778.

20. Moyer VA. Screening for lung cancer: U.S. Preventive Services Task Force recommendation statement. *Ann Intern Med.* 2014;160(5):330-338.
21. Jenson TS, Chin J, Ashby L, Hermansen J, Hutter JD. Decision Memo for Screening for Lung Cancer with Low Dose Computed Tomography (LDCT) (CAG-00439N). . In. Baltimore, MD: Centers for Medicare & Medicaid Services; 2015.
22. Gelberg L, Gallagher TC, Andersen RM, Koegel P. Competing priorities as a barrier to medical care among homeless adults in Los Angeles. *Am J Public Health.* 1997;87(2):217-220.
23. Baggett TP, O'Connell JJ, Singer DE, Rigotti NA. The unmet health care needs of homeless adults: a national study. *Am J Public Health.* 2010;100(7):1326-1333.
24. Kushel MB, Vittinghoff E, Haas JS. Factors associated with the health care utilization of homeless persons. *JAMA.* 2001;285(2):200-206.
25. Weinstein LC, LaNoue M, Hurley K, Sifri R, Myers R. Using Concept Mapping to Explore Barriers and Facilitators to Breast Cancer Screening in Formerly Homeless Women with Serious Mental Illness. *J Health Care Poor Underserved.* 2015;26(3):908-925.
26. Fazel S, Khosla V, Doll H, Geddes J. The prevalence of mental disorders among the homeless in western countries: systematic review and meta-regression analysis. *PLoS Med.* 2008;5(12):e225.
27. Koegel P, Burnam MA, Farr RK. The prevalence of specific psychiatric disorders among homeless individuals in the inner city of Los Angeles. *Arch Gen Psychiatry.* 1988;45(12):1085-1092.
28. Brown RT, Kiely DK, Bharel M, Mitchell SL. Geriatric syndromes in older homeless adults. *J Gen Intern Med.* 2011;27(1):16-22.
29. Topolovec-Vranic J, Ennis N, Colantonio A, et al. Traumatic brain injury among people who are homeless: a systematic review. *BMC Public Health.* 2012;12:1059.
30. Topolovec-Vranic J, Ennis N, Howatt M, et al. Traumatic brain injury among men in an urban homeless shelter: observational study of rates and mechanisms of injury. *CMAJ Open.* 2014;2(2):E69-76.
31. Hwang SW, Colantonio A, Chiu S, et al. The effect of traumatic brain injury on the health of homeless people. *CMAJ.* 2008;179(8):779-784.
32. Oddy M, Moir JF, Fortescue D, Chadwick S. The prevalence of traumatic brain injury in the homeless community in a UK city. *Brain Inj.* 2012;26(9):1058-1064.
33. Baggett TP, Campbell EG, Chang Y, Magid LM, Rigotti NA. Posttraumatic Stress Symptoms and Their Association With Smoking Outcome Expectancies Among Homeless Smokers in Boston. *Nicotine Tob Res.* 2015.
34. Baggett TP, Rigotti NA, Campbell EG. Cost of Smoking among Homeless Adults. *N Engl J Med.* 2016;374(7):697-698.
35. Baggett TP, Campbell EG, Chang Y, Rigotti NA. Other tobacco product and electronic cigarette use among homeless cigarette smokers. *Addict Behav.* 2016;60:124-130.
36. Percac-Lima S, Ashburner JM, Bond B, Oo SA, Atlas SJ. Decreasing disparities in breast cancer screening in refugee women using culturally tailored patient navigation. *J Gen Intern Med.* 2013;28(11):1463-1468.
37. Percac-Lima S, Lopez L, Ashburner JM, Green AR, Atlas SJ. The longitudinal impact of patient navigation on equity in colorectal cancer screening in a large primary care network. *Cancer.* 2014;120(13):2025-2031.
38. Percac-Lima S, Ashburner JM, Zai AH, et al. Patient Navigation for Comprehensive Cancer Screening in High-Risk Patients Using a Population-Based Health Information Technology System: A Randomized Clinical Trial. *JAMA Intern Med.* 2016;176(7):930-937.
39. Freeman HP. Patient navigation: a community based strategy to reduce cancer disparities. *J Urban Health.* 2006;83(2):139-141.

40. Asgary R, Naderi R, Wisnivesky J. Opt-Out Patient Navigation to Improve Breast and Cervical Cancer Screening Among Homeless Women. *J Womens Health (Larchmt).* 2017.
41. Braun KL, Thomas WL, Jr., Domingo JL, et al. Reducing cancer screening disparities in medicare beneficiaries through cancer patient navigation. *J Am Geriatr Soc.* 2015;63(2):365-370.
42. Myers RE, Sifri R, Daskalakis C, et al. Increasing colon cancer screening in primary care among African Americans. *J Natl Cancer Inst.* 2014;106(12).
43. Honeycutt S, Green R, Ballard D, et al. Evaluation of a patient navigation program to promote colorectal cancer screening in rural Georgia, USA. *Cancer.* 2013;119(16):3059-3066.
44. Wang X, Fang C, Tan Y, Liu A, Ma GX. Evidence-based intervention to reduce access barriers to cervical cancer screening among underserved Chinese American women. *J Womens Health (Larchmt).* 2010;19(3):463-469.
45. Paskett ED, Harrop JP, Wells KJ. Patient navigation: an update on the state of the science. *CA Cancer J Clin.* 2011;61(4):237-249.
46. Phillips CE, Rothstein JD, Beaver K, Sherman BJ, Freund KM, Battaglia TA. Patient navigation to increase mammography screening among inner city women. *J Gen Intern Med.* 2010;26(2):123-129.
47. Robinson-White S, Conroy B, Slavish KH, Rosenzweig M. Patient navigation in breast cancer: a systematic review. *Cancer Nurs.* 2010;33(2):127-140.
48. Wells KJ, Battaglia TA, Dudley DJ, et al. Patient navigation: state of the art or is it science? *Cancer.* 2008;113(8):1999-2010.
49. Chen LA, Santos S, Jandorf L, et al. A program to enhance completion of screening colonoscopy among urban minorities. *Clin Gastroenterol Hepatol.* 2008;6(4):443-450.
50. Jandorf L, Braschi C, Ernstoff E, et al. Culturally targeted patient navigation for increasing african americans' adherence to screening colonoscopy: a randomized clinical trial. *Cancer Epidemiol Biomarkers Prev.* 2013;22(9):1577-1587.
51. Lasser KE, Murillo J, Medlin E, et al. A multilevel intervention to promote colorectal cancer screening among community health center patients: results of a pilot study. *BMC Fam Pract.* 2009;10:37.
52. Christie J, Itzkowitz S, Lihau-Nkanza I, Castillo A, Redd W, Jandorf L. A randomized controlled trial using patient navigation to increase colonoscopy screening among low-income minorities. *J Natl Med Assoc.* 2008;100(3):278-284.
53. Jandorf L, Gutierrez Y, Lopez J, Christie J, Itzkowitz SH. Use of a patient navigator to increase colorectal cancer screening in an urban neighborhood health clinic. *J Urban Health.* 2005;82(2):216-224.
54. Lasser KE, Murillo J, Lisboa S, et al. Colorectal cancer screening among ethnically diverse, low-income patients: a randomized controlled trial. *Archives of internal medicine.* 2011;171(10):906-912.
55. Horne HN, Phelan-Emrick DF, Pollack CE, et al. Effect of patient navigation on colorectal cancer screening in a community-based randomized controlled trial of urban African American adults. *Cancer Causes Control.* 2015;26(2):239-246.
56. Percac-Lima S, Grant RW, Green AR, et al. A culturally tailored navigator program for colorectal cancer screening in a community health center: a randomized, controlled trial. *J Gen Intern Med.* 2009;24(2):211-217.
57. Hoffman HJ, LaVerda NL, Young HA, et al. Patient navigation significantly reduces delays in breast cancer diagnosis in the District of Columbia. *Cancer Epidemiol Biomarkers Prev.* 2012;21(10):1655-1663.

58. Markossian TW, Darnell JS, Calhoun EA. Follow-up and timeliness after an abnormal cancer screening among underserved, urban women in a patient navigation program. *Cancer Epidemiol Biomarkers Prev.* 2012;21(10):1691-1700.
59. Battaglia TA, Bak SM, Heeren T, et al. Boston Patient Navigation Research Program: the impact of navigation on time to diagnostic resolution after abnormal cancer screening. *Cancer Epidemiol Biomarkers Prev.* 2012;21(10):1645-1654.
60. Raich PC, Whitley EM, Thorland W, Valverde P, Fairclough D. Patient navigation improves cancer diagnostic resolution: an individually randomized clinical trial in an underserved population. *Cancer Epidemiol Biomarkers Prev.* 2012;21(10):1629-1638.
61. Ramirez AG, Perez-Stable EJ, Penedo FJ, et al. Navigating Latinas with breast screen abnormalities to diagnosis: the Six Cities Study. *Cancer.* 2013;119(7):1298-1305.
62. Lee JH, Fulp W, Wells KJ, Meade CD, Calcano E, Roetzheim R. Patient navigation and time to diagnostic resolution: results for a cluster randomized trial evaluating the efficacy of patient navigation among patients with breast cancer screening abnormalities, Tampa, FL. *PLoS One.* 2013;8(9):e74542.
63. Lee JH, Fulp W, Wells KJ, Meade CD, Calcano E, Roetzheim R. Effect of patient navigation on time to diagnostic resolution among patients with colorectal cancer-related abnormalities. *J Cancer Educ.* 2014;29(1):144-150.
64. American Cancer Society. Patient Navigators Help Cancer Patients Manage Care. https://www.cancer.org/latest-news/navigators-help-cancer-patients-manage-their-care.html. Published February 24, 2017. Accessed March 1, 2017.
65. National Cancer Institute. Patient Navigation Research Program (PNRP). U.S. Department of Health and Human Services, National Institutes of Health, National Cancer Institute. https://www.cancer.gov/about-nci/organization/crchd/disparities-research/pnrp. Accessed March 28, 2017.
66. O'Connell JJ, Oppenheimer SC, Judge CM, et al. The Boston Health Care for the Homeless Program: a public health framework. *Am J Public Health.* 2010;100(8):1400-1408.
67. Bauer LK, Brody JK, Leon C, Baggett TP. Characteristics of Homeless Adults Who Died of Drug Overdose: A Retrospective Record Review. *J Health Care Poor Underserved.* 2016;27(2):846-859.
68. Bharel M, Lin WC, Zhang J, O'Connell E, Taube R, Clark RE. Health care utilization patterns of homeless individuals in Boston: preparing for Medicaid expansion under the Affordable Care Act. *Am J Public Health.* 2013;103 Suppl 2:S311-317.
69. Tsai D. MassHealth Transmittal Letter PHY-147. In: Office of Medicaid, Massachusetts Department of Health and Human Services; March 2016.
70. Wender R, Fontham ET, Barrera E, Jr., et al. American Cancer Society lung cancer screening guidelines. *CA Cancer J Clin.* 2013;63(2):107-117.
71. Detterbeck FC, Mazzone PJ, Naidich DP, Bach PB. Screening for lung cancer: Diagnosis and management of lung cancer, 3rd ed: American College of Chest Physicians evidence-based clinical practice guidelines. *Chest.* 2013;143(5 Suppl):e78S-92S.
72. Jaklitsch MT, Jacobson FL, Austin JH, et al. The American Association for Thoracic Surgery guidelines for lung cancer screening using low-dose computed tomography scans for lung cancer survivors and other high-risk groups. *J Thorac Cardiovasc Surg.* 2012;144(1):33-38.
73. Samet JH, Crowell R, Estepar RSJ, et al. Providing Guidance on Lung Cancer Screening to Patients and Physicians: An Update from the American Lung Association Lung Cancer Screening Committee. . In. Chicago: American Lung Association; April 30, 2015.

74. Bach PB, Mirkin JN, Oliver TK, et al. Benefits and harms of CT screening for lung cancer: a systematic review. *JAMA.* 2012;307(22):2418-2429.
75. Park ER, Streck JM, Gareen IF, et al. A qualitative study of lung cancer risk perceptions and smoking beliefs among national lung screening trial participants. *Nicotine Tob Res.* 2014;16(2):166-173.
76. Park ER, Ostroff JS, Rakowski W, et al. Risk perceptions among participants undergoing lung cancer screening: baseline results from the National Lung Screening Trial. *Ann Behav Med.* 2009;37(3):268-279.
77. Ryan H, Trosclair A, Gfroerer J. Adult current smoking: differences in definitions and prevalence estimates--NHIS and NSDUH, 2008. *J Environ Public Health.* 2012;2012:918368.
78. Health Resources and Services Administration, Bureau of Primary Health Care. Authorizing Legislation of the Health Center Program: Section 330 of the Public Health Service Act (42 USCS § 254b). http://bphc.hrsa.gov/policiesregulations/legislation/authorizing330.pdf. Accessed March 28, 2017.
79. U.S. Department of Housing and Urban Development. S. 896 HEARTH Act of 2009. https://www.hudexchange.info/resource/1717/s-896-hearth-act/. Accessed March 12, 2017.
80. American College of Radiology. Lung CT Screening Reporting and Data System (Lung-RADS). http://www.acr.org/Quality-Safety/Resources/LungRADS. Accessed March 28, 2017.
81. Fiore MC, Jaen CR, Baker TB, et al. Treating Tobacco Use and Dependence: 2008 Update. Clinical Practice Guideline. . In. Rockville, MD: U.S. Department of Health and Human Services. Public Health Service; May 2008.
82. Park ER, Gareen IF, Japuntich S, et al. Primary Care Provider-Delivered Smoking Cessation Interventions and Smoking Cessation Among Participants in the National Lung Screening Trial. *JAMA Intern Med.* 2015;175(9):1509-1516.
83. Poghosyan H, Kennedy Sheldon L, Cooley ME. The impact of computed tomography screening for lung cancer on smoking behaviors: a teachable moment? *Cancer Nurs.* 2012;35(6):446-475.
84. Greene J, Fahrney K, Byron M. Health Care for the Homeless User/Visit Surveys, RTI Project Number 07147.021. . In. Research Triangle Park, NC: RTI International; 2004.
85. Kaufman AR, Koblitz AR, Persoskie A, et al. Factor Structure and Stability of Smoking-Related Health Beliefs in the National Lung Screening Trial. *Nicotine Tob Res.* 2016;18(3):321-329.
86. Lowenstein LM, Richards VF, Leal VB, et al. A brief measure of Smokers' knowledge of lung cancer screening with low-dose computed tomography. *Prev Med Rep.* 2016;4:351-356.
87. Schwarzer R, Jerusalem M. Generalized Self-Efficacy scale. In: Weinman J, Wright S, Johnston M, eds. *Measures in health psychology: A user's portfolio. Causal and control beliefs.* Windsor, UK: NFER-NELSON; 1995:35-37.
88. Scholz U, Gutierrez Dona B, Sud S, Schwarzer R. Is General Self-Efficacy a Universal Construct? Psychometric Findings from 25 Countries. *European Journal of Psychological Assessment.* 2002;18(3):242-251.
89. US Department of Commerce CB. National Cancer Institute and Food and Drug Administration co-sponsored Tobacco Use Supplement to the Current Population Survey. In. *2018-2019*2020.
90. Kish DH, Reitzel LR, Kendzor DE, Okamoto H, Businelle MS. Characterizing Concurrent Tobacco Product Use Among Homeless Cigarette Smokers. *Nicotine Tob Res.* 2014.

91. Heatherton TF, Kozlowski LT, Frecker RC, Fagerstrom KO. The Fagerstrom Test for Nicotine Dependence: a revision of the Fagerstrom Tolerance Questionnaire. *Br J Addict.* 1991;86(9):1119-1127.
92. DiClemente CC, Prochaska JO, Fairhurst SK, Velicer WF, Velasquez MM, Rossi JS. The process of smoking cessation: an analysis of precontemplation, contemplation, and preparation stages of change. *J Consult Clin Psychol.* 1991;59(2):295-304.
93. Snow MG, Prochaska JO, Rossi JS. Stages of change for smoking cessation among former problem drinkers: a cross-sectional analysis. *J Subst Abuse.* 1992;4(2):107-116.
94. Biener L, Abrams DB. The Contemplation Ladder: validation of a measure of readiness to consider smoking cessation. *Health Psychol.* 1991;10(5):360-365.
95. Wong LE, Hawkins JE, Murrell KL. Where are all the patients? Addressing Covid-19 fear to encourage sick patients to seek emergency care. *NEJM Catalyst Innovations in Care Delivery.* 2020.
96. Toolkit P. COVID-19 Community Response Survey Guidance. In:2020.
97. (C3PNO) CCoCPNO. COVID-19 Module for NIDA-Supported Cohorts Affected by HIV and Substance Use. In:2020.
98. Bush K, Kivlahan DR, McDonell MB, Fihn SD, Bradley KA. The AUDIT alcohol consumption questions (AUDIT-C): an effective brief screening test for problem drinking. *Archives of internal medicine.* 1998;158(16):1789-1795.
99. Bradley KA, Bush KR, Epler AJ, et al. Two brief alcohol-screening tests From the Alcohol Use Disorders Identification Test (AUDIT): validation in a female Veterans Affairs patient population. *Archives of internal medicine.* 2003;163(7):821-829.
100. Skinner HA. The drug abuse screening test. *Addictive behaviors.* 1982;7(4):363-371.
101. Yudko E, Lozhkina O, Fouts A. A comprehensive review of the psychometric properties of the Drug Abuse Screening Test. *Journal of substance abuse treatment.* 2007;32(2):189-198.
102. Kessler RC, Andrews G, Colpe LJ, et al. Short screening scales to monitor population prevalences and trends in non-specific psychological distress. *Psychological medicine.* 2002;32(6):959-976.
103. Kessler RC, Barker PR, Colpe LJ, et al. Screening for serious mental illness in the general population. *Arch Gen Psychiatry.* 2003;60:184-189.
104. American College of Radiology. Lung-RADS Version 1.0 Assessment Categories. Available at http://www.acr.org/Quality-Safety/Resources/LungRADS. In:April 28, 2014.
105. Elo S, Kyngas H. The qualitative content analysis process. *J Adv Nurs.* 2008;62(1):107-115.
106. Creswell JW, Plano Clark VL. Chapter 3: Choosing a Mixed Methods Design. In: *Designing and Conducting Mixed Methods Research.* 2nd ed. Los Angeles: Sage; 2011:53-106.
107. Health Resources and Services Administration, U.S. Department of Health & Human Services. 2015 Health Center Data: National Health Care for the Homeless Program Grantee Data. https://bphc.hrsa.gov/uds/datacenter.aspx?year=2015&fd=ho. Accessed March 16, 2017.
108. Center for Tobacco Treatment Research & Training, University of Massachusetts Medical School. Basic Skills for Working with Smokers - Online Course. http://www.umassmed.edu/tobacco/training/basicskills_online/. Accessed March 28, 2017.

TITLE: Lung cancer screening navigation for homeless people: A pragmatic trial

PRINCIPAL INVESTIGATOR: Travis P. Baggett, MD, MPH

GRANT NUMBER: 131162-RSG-17-157-01-CPPB (American Cancer Society)

VERSION DATE: July 23, 2021

I. BACKGROUND AND SIGNIFICANCE

A. Historical background

An estimated 2.3-3.5 million people experience homelessness annually in the US.[1] Lung cancer incidence and mortality are substantially higher in this vulnerable population than in comparably-aged non-homeless individuals.[2-4] To address this disparity, we propose a pragmatic clinical trial to test the effect of patient navigation on lung cancer screening (LCS) completion among homeless-experienced adults at increased risk for lung cancer. Work that we and others have done suggests 2 reasons why such an intervention is needed.

1) Lung cancer is a major cause of death among homeless adults and the leading cause of tobacco-attributable death in this population.

In our study of 28,033 adults who used Boston Health Care for the Homeless Program (BHCHP) services in 2003-08, cancer was the second-leading cause of death and the leading killer of adults ≥45 years old.[5] Lung cancer accounted for one-third of these cancer deaths.[5] In a subsequent study of cancer epidemiology in this cohort, lung cancer was the leading type of incident cancer and the leading cause of cancer death among both men and women, with age-standardized incidence and mortality rates exceeding those in the Massachusetts adult population more than 2-fold.[2] An estimated 88% of incident lung cancer cases[2] and 93% of lung cancer deaths[6] were attributable to tobacco smoking, making lung cancer the leading type of smoking-attributable cancer and the leading cause of smoking-attributable death among homeless people. The excess burden of lung cancer in these studies of homeless adults in Boston was similar to that observed in studies of homeless and marginally housed people in Canada[4] and Glasgow[3] (Table 1), suggesting that these disparities are not geographically constrained. This body of evidence highlights the need for interventions to reduce lung cancer disparities in homeless populations. One avenue for doing so is LCS with low-dose computed tomography (LDCT) to promote detection and potentially curative treatment of early-stage lung cancer. This strategy was associated with a 20% reduction in lung cancer mortality in the National Lung Screening Trial (NLST).[7] However, implementing such a strategy presents formidable hurdles in the setting of homelessness.

Table 1. Lung cancer among homeless adults

Study location (yrs)	SIR/SMR (95% CI)
Boston (2003-2008)	
Incidence, men	2.30 (1.84, 2.84)
Incidence, women	2.23 (1.41, 3.35)
Mortality, men	2.39 (1.83, 3.08)
Mortality, women	2.31 (1.26, 3.88)
Canada (1991-2001)	
Mortality, men	1.91 (1.67, 2.18)
Mortality, women	1.73 (1.26, 2.36)
Glasgow (1975-1993)	
Incidence, men	1.64 (1.41, 1.86)

2) Homeless people have suboptimal rates of cancer screening and are diagnosed with screen-detectable malignancies at later stages than non-homeless people; barriers to implementing LCS in the setting of homelessness could exacerbate existing disparities in lung cancer outcomes.

In the 2003-08 BHCHP study of cancer epidemiology described above, homeless individuals were diagnosed with colorectal cancer and female breast cancer at significantly later stages than adults in the Massachusetts general population.[2] Several studies have documented low rates of screening for these malignancies among homeless individuals.[8-12] Given the high prevalence of smoking among homeless people[8,13-18] and the aging of the homeless population,[19] many homeless individuals are expected to meet the recommended criteria for consideration of LCS with LDCT[20,21]; however, as with colorectal and breast cancer screening, there are several obstacles to successfully implementing LCS in homeless populations. Homeless individuals struggling to meet basic subsistence needs may place a low priority on screening for asymptomatic illness[22] or may feel too emotionally overwhelmed to cope with the possibility of an abnormal result. Additionally, despite a high burden of cancer risk factors, most homeless people appear to believe that their susceptibility to cancer is equal to or less than that of others the same age.[8] Lack of insurance may pose another barrier,[23,24] although this is less of a concern in Massachusetts and could improve elsewhere in states that expanded Medicaid under the Affordable Care Act. Even among insured individuals who are interested in obtaining LDCT screening, the process of scheduling and attending the test may prove logistically challenging because of difficulty navigating complex facilities and care systems,[25] especially for the large proportions of homeless individuals with psychiatric and cognitive impairments.[26-32] Furthermore, ensuring timely follow-up of abnormal results may be challenging because of communication barriers and competing psychosocial issues. These barriers introduce the possibility that existing disparities in lung cancer mortality could worsen if homeless people are less able to complete LCS than other segments of the population.

B. Preliminary data

Our prior epidemiologic work demonstrating the burden of lung cancer disparities among homeless people is described above and provides the motivation for the proposed study. In addition, this study is informed by administrative data from BHCHP, survey and clinical trial studies with homeless smokers conducted by the PI (Dr. Baggett), navigation studies conducted by co-investigator Dr. Percac-Lima, and LCS-oriented studies conducted by co-investigators Dr. Park and Dr. Rigotti. Collectively, this preliminary data has demonstrated the following:

1) We have the patient volume to support this study. Based on internal data from the BHCHP Institute, 3,443 unique individuals aged 55-77 years old were seen at any BHCHP site from 9/1/2015 to 9/1/2016. Of these, 53% were current smokers and 23% were former smokers, totaling 2,616 potentially eligible individuals seen by the program in a 12-month period. When extrapolated over the planned 2.5-year enrollment period, we anticipate being able to recruit and randomize the proposed number of 300 participants.

2) We have characterized the target population. In April-July 2014, we used time-location sampling to conduct a survey of 306 homeless adult smokers at 5 high-volume BHCHP clinical sites.[33-35] The response rate was 86%. One-quarter of respondents (N=78) were 55-77 years old. Of these, the vast majority were non-Hispanic white or black men with a high school degree. Seventy percent had ever experienced a traumatic head injury, 64% had used any drug in the past month, 39% had consumed alcohol to intoxication in the past month, 60% reported feeling seriously depressed, and half screened positive for PTSD. One-third reported COPD and 17% reported cardiovascular disease. Past-month difficulty finding shelter, food, and clothing were common. The mean number of cigarettes per day was 12 and the mean Fagerstrom nicotine dependence score was 3.8 (range 0-12). These findings highlight the medical complexity and competing life issues of older homeless smokers and underscore the need for a navigation-based intervention to reduce the barriers to LCS.

3) We can recruit and longitudinally retain homeless people in a clinical trial. From October 2015 to June 2016, we conducted a 3-arm (N=25 per arm) pilot randomized controlled trial testing the effect of a) contingent financial rewards for smoking abstinence, and b) text messaging to support smoking abstinence, against c) a shared control condition, among homeless smokers at BHCHP (NCT02565381). Sixty-eight percent of eligible individuals enrolled, and we reached our target enrollment within 6 months. Study attendance and retention rates were excellent. Of 14 possible assessment visits, participants attended a median of 10, with 97% attending ≥1 visit and 77% attending ≥7 visits. These findings demonstrate that our team has the expertise to conduct a behavioral treatment trial in the context of homelessness and to engage and retain this vulnerable population in a longitudinal fashion. This is facilitated by our extensive knowledge of the target population, our person-centered approach to participant engagement, our strong partnership with BHCHP, and the central location of BHCHP headquarters with respect to other homeless services.

4) Our navigation intervention is based on a model that has proven effective at improving lung and other cancer screening among underserved patient populations in Boston. Key collaborator, Dr. Sanja Percac-Lima, has developed and tested navigation interventions that have been associated with improved cancer screening rates among diverse, low-income community health center patients in Boston.[36,37] She conducted a randomized controlled trial (RCT) across 18 clinical practices in an academic primary care network evaluating the impact of patient navigation relative to usual care for high-risk individuals who were overdue for breast, colorectal, and/or cervical cancer screening.[38] Patients assigned to navigation were significantly more likely to complete screening for breast (23.4% vs. 16.6%, p=0.009), cervical (14.4% vs. 8.6%, p=0.007), and colorectal (13.7% vs. 7.0%, p<0.001) cancer during the follow-up period.[38] More recently, with grant support from ACS and in collaboration with Drs. Park and Rigotti, Dr. Percac-Lima was the principal investigator of an RCT testing the effect of patient navigation on chest CT completion among low-income patients eligible for LCS at 5 MGH-affiliated health centers in greater Boston. Preliminary analysis of data after 11 months of follow-up has shown that participants in the navigation arm were significantly more likely to obtain a screening lung CT than participants assigned to usual care (24% vs. 9%, p<0.01). The LCS navigation approach used in our study will be based on these successful models.

C. Study rationale

Mitigating disparities in lung cancer outcomes and fully realizing the potential benefits of LCS among homeless people will require thoughtful interventions deployed through the clinical programs that serve this vulnerable population. Patient navigation, a strategy for guiding individuals through complex health systems,[39] may be a promising approach for helping homeless people overcome their unique barriers to LCS.[40] Navigation interventions have been shown to improve cancer screening participation[36,37,41-56] and diagnostic resolution of abnormal results[57-63] in vulnerable populations. Patient navigation has been endorsed by the ACS[64] and the National Cancer Institute[65] as a valuable approach to reducing cancer health disparities,[65] but this approach has not been tested rigorously in a homeless health care setting. Our research group is well-poised to conduct such a study by leveraging our extensive clinical and research experience with this population and our 9-year partnership with BHCHP, an internationally-renowned organization that serves over 12,000 currently (84%) and formerly (16%) homeless people annually through dozens of clinical sites in greater Boston.[66] This innovative community partnership has given rise to numerous impactful studies on the health of homeless-experienced people,[2,5,6,33-35,67] including a recently pilot trial for homeless smokers that had brisk recruitment, high retention rates, and encouraging results (NCT02565381).

II. SPECIFIC AIMS

Aim 1: To determine the effect of patient navigation, added to usual care, on 1°) LCS LDCT completion at 6 months (26 weeks) and 2°) LCS LDCT completion at 6 months (26 weeks) and diagnostic follow-up of abnormal results within 1 month (4 weeks) of the recommended timeframe, among homeless-experienced people who are eligible for LCS.

Hypothesis: Participants assigned to receive patient navigation will have significantly greater 1°) LCS LDCT completion at 6 months (26 weeks) and 2°) LCS LDCT completion at 6 months (26 weeks) with timely diagnostic follow-up of abnormal results.

Aim 2. To conduct post-intervention qualitative interviews of trial participants and BHCHP PCPs to explain and interpret the quantitative findings of the pragmatic clinical trial.

Based on a sequential explanatory mixed methods approach, Aim 2 interviews will examine how and why participants decided whether to undergo LCS, the barriers they encountered in doing so, the ways in which the navigator helped them overcome these barriers, and opportunities for improving the intervention for future use.

III. SUBJECT SELECTION

A. Inclusion and exclusion criteria

To be eligible to participate, individuals must meet **all** the following criteria:

1) Aged 55-77 years old, assessed by self-report and verified by date of birth.
   Rationale: LCS LCDT is covered only for patients aged 55-77.
2) Have a 30 pack-year smoking history *and* have smoked within the past 15 years.
   Rationale: We will be recruiting exclusively smokers because they are at the highest risk for lung cancer and therefore stand to receive the most benefit from a LCS. These smoking history parameters are consistent with current practice guidelines and Medicare eligibility criteria.
3) Have a BHCHP primary care provider (PCP).
   Rationale: Patient navigation is designed to support and extend services provided by medical practitioners. Having a PCP will help to ensure longitudinal medical oversight of the LCS process and appropriate follow-up of abnormal results.
4) Are currently or formerly homeless, assessed by self-report and defined as ever having experienced a time in their life where they did not have fixed, regular housing.
   Rationale: Both currently and formerly homeless individuals were included in our epidemiologic analysis showing a more than 2-fold higher incidence and mortality rate from lung cancer compared to the general Massachusetts adult population.[2] BHCHP and most HCH programs nationally continue to serve patients after they have gained housing.
5) Be proficient in English, assessed with items asking about native language and self-reported comfort communicating in English among non-native speakers.
   Rationale: Because of a budget reduction by the funding agency, we do not have the resources to develop study materials or conduct in-person navigation in languages other than English. Additionally, the vast majority of individuals in the target population speak English.

Individuals meeting **any** of the following criteria will be excluded from the study:

1) A recent prior chest computed tomography (CT) imaging (in the past 12 months).
   Rationale: This study is aimed at individuals who are most likely to benefit from LCS, based on evidence from the National Lung Screening Trial. Potential participants that have recently received a chest CT will have recently been scanned for lung cancer, therefore an additional screening will not benefit their health outcomes.
2) Any personal history of lung cancer, or current presentation with symptoms concerning for lung cancer (e.g. hemoptysis or unexplained weight loss of >15 lbs. in the past year).
   Rationale: Individuals with a history of lung cancer or symptoms concerning for lung cancer require surveillance or diagnostic lung imaging, respectively, and are not appropriate candidates for screening lung imaging, which by definition is designed for individuals without symptoms or history of the illness being screened for.
3) Inability to provide informed consent, assessed with knowledge questions about the material presented during the informed consent process that individuals must correctly answer before providing informed consent to participate.
   Rationale: In this vulnerable population, we wish to take a conservative approach to the informed consent process to help ensure that participants fully understand the pros and cons of participating in the research study.
4) PCP is the study PI (Dr. Travis Baggett).
   Rationale: BHCHP patients under the primary care of Dr. Baggett may feel unduly influenced to participate. In addition, since the primary study outcome (receipt of LDCT for LCS) requires PCP involvement, the inclusion of Dr. Baggett's patients creates a conflict of interest and the potential for bias.

B. Source of subjects and recruitment methods

This study will be registered with ClinicalTrials.gov prior to the recruitment and enrollment of human subjects. All participants will be recruited from BHCHP clinical sites. BHCHP does not have its own IRB but instead will rely on the Partners Human Research Committee for IRB review through a reliance agreement initiated through SMART IRB. In addition to being a faculty physician-investigator at Massachusetts General Hospital and Harvard Medical School, the study PI is the Director of Research at BHCHP and is very familiar with the clinical environment and patient population served by the program.

Individuals must be homeless to enroll in services at BHCHP; however, some patients continue receiving care at the program after they are no longer homeless. An internal analysis of individuals seen at BHCHP in 2011 found that 16% of patients were housed, most without supportive services and therefore at potentially high risk for recurrent homelessness. Because our epidemiologic analyses demonstrating the dramatic lung cancer disparities experienced by BHCHP patients included both currently and formerly homeless individuals, and because of the often cyclic nature of homelessness, we will include both currently and formerly homeless individuals in the proposed study. We refer to this group collectively as "homeless-experienced." Owing to the Massachusetts system of universal health insurance, about 80% of BHCHP patients are insured by the state Medicaid program,[68] which covers LCS LDCT among eligible beneficiaries,[69] and a sizable proportion of those remaining are covered by Medicare alone. This will help ensure the availability of LCS LDCT for the vast majority of potential participants.

Participants will be recruited through a combination of 3 methods:

1) In-person screening of patients at BHCHP clinical sites: Study personnel will screen patients in-person at 6 high-volume BHCHP clinic sites: a) Pine Street Inn, a 470-bed shelter where BHCHP operates a medical clinic 7 days/week in the men's shelter and 6 days/week in the women's shelter; b) Southampton Shelter, a 400-bed men's shelter where BHCHP operates a medical clinic 7 days/week; c) Woods Mullen Shelter, a 200-bed women's shelter where BHCHP operates a medical clinic 5 days/week; d) New England Center and Home for Veterans, a 209-bed emergency and transitional shelter where BHCHP operates a medical clinic 5 days/week; e) St. Francis House, a daytime multiservice facility for homeless people where BHCHP operates a medical clinic 5 days/week; and f) Jean Yawkey Place, the BHCHP headquarters facility where medical, behavioral health, and dental clinics operate 5 days/week with an on-site pharmacy and medical respite unit. Our prior survey fieldwork with this population has given us extensive experience with tactfully deploying this proactive, in-person recruitment technique.[33-35] In light of the COVID-19 pandemic, BHCHP clinical sites and shelters are experiencing a significant decrease in patient volume and a lack of additional space to safely conduct in-person recruitment and data collection activities. Under these circumstances, study personnel will initially focus in-person screening and data collection efforts at Jean Yawkey Place, which has the highest volume of patients and the greatest degree of flexibility in physical space and layout. This will allow the study personnel to have an in-person presence at the clinic while practicing physical distancing and prioritizing the health and well-being of participants and study staff. If COVID-19 restrictions loosen and patient volume increases at shelter sites, we will expand our efforts to include the five additional sites, depending on COVID-19 public health guidelines and facility protocols.

2) Review of BHCHP electronic records: BHCHP has a comprehensive EHR system (Epic) that prompts providers to assess tobacco use at every clinical encounter, facilitating determination of smoking history and current smoking status. Individuals with a BHCHP PCP who have been seen in a BHCHP clinic in the past year and who were current or former smokers at the time of their most recent clinical encounter will be approached in person at BHCHP clinical sites if they have a scheduled appointment or contacted proactively via phone (if they have listed contact information) by a research coordinator and screened for eligibility to participate. The BHCHP Chief Medical Officer has approved this recruitment strategy on behalf of all primary care providers at BHCHP clinical sites, who will in turn be notified about our recruitment approach prior to the start of the study (letter of approval in Appendix J).

3) Referrals from BHCHP providers: We will advertise the study to all BHCHP providers and in all BHCHP clinical sites. Individuals deemed by a BHCHP provider to be potential candidates for LCS can be referred to the research coordinator, who will screen referred patients for eligibility to participate.

IV. SUBJECT ENROLLMENT

An integrated study schema depicting recruitment, enrollment, treatment assignment, and outcomes assessment has been uploaded as an attachment to this application.

A. COVID-19 symptom and exposure screening

BHCHP staff currently screen all patients for COVID-19 symptoms or a recent positive COVID-19 test upon entry to BHCHP clinics and facilities. BHCHP is also providing patients with facemasks before entering the building. Study personnel will re-screen participants using the same approach before conducting any research activities in an enclosed room. If BHCHP ceases their COVID-19 screening procedure, study personnel will use BHCHP's "COVID-19

Screening Tool for People Experiencing Homelessness" to screen all participants for current COVID-19 symptoms and past 14-day COVID-19 exposures (i.e., travel to high risk countries or states; unmasked exposure to a person with COVID-19). Study staff will wear appropriate personal protective equipment (PPE) per MGH guidelines and policies and remain 6 feet away from the patient. If patient endorses any COVID-19 symptoms or high-risk exposures, study personnel will immediately notify a clinic staff member of the presence of a symptomatic patient and will not engage in any study activities. Immediately after departure of the symptomatic patient, study personnel will disinfect surfaces that were within 6 feet of the symptomatic patient. If the participant reports not having symptoms, they can be directed to the study table/interview room. If the study coordinator makes initial contact with a participant over the phone, before scheduling an in-person visit, study personnel will screen the participant within 72 hours before the appointment. If participants report COVID-19 symptoms or exposures, study staff will request participants' permission to contact their BHCHP PCP via Epic messaging who can then advise the participant on next steps (e.g., testing and/or quarantine). If participants do not provide permission to study staff to contact their PCP, study staff will recommend that the participant seek care for their symptoms and/or exposure and offer to do the initial study procedures over the phone. If they are not interested in completing any further study procedures over the phone, study staff will call the participant again in 10 days to check-in and rescreen before attempting to schedule any study procedures in person. Participants will be advised to put on a facemask, regardless of symptoms, before coming in to meet with the research coordinator. Research personnel will instruct participants to notify them before arriving if they have fever or symptoms of COVID-19.

B. Methods of enrollment

Participants recruited through the above methods (section III.B) will be screened for eligibility either in-person or by phone. Prior to contacting participants, study staff will use the eligibility screener (Appendix A) to record information related to eligibility that was able to be collected during the prior review of prospective participants' BHCHP EHR (as referenced above).

Upon approaching the participants, study staff will use participant self-report to verify any information collected via their EHR and will collect the minimum amount of additional information necessary to assess and verify study eligibility. Eligible individuals identified through in-person screening will be offered the chance to enroll immediately. Those identified through phone screening will be asked to attend an in-person enrollment visit at one of clinic sites listed above. Participants will be asked to provide verbal informed consent before enrolling.

At least six months after initial enrollment, participants who were randomized to the patient navigation arm of the study will be contacted via phone or approached in person at their next BHCHP appointment to be offered the opportunity to participate in a 45-minute qualitative interview. Forty participants will be interviewed in total, with purposive sampling of participants who did (N=20) and participants who did not (N=20) attain the primary outcome of completing a lung screening CT scan within six months.

At approximately 6 months after the last patient participant is enrolled, providers who had at least one patient enrolled in the trial will be contacted via phone, email, or in person to be offered the opportunity to participate in a 45-minute qualitative interview. Ten participants will be interviewed in total, with purposive sampling to ensure that at least one provider who works at each of the targeted BHCHP clinic sites are interviewed.

C. Informed consent procedures

We will obtain verbal informed consent for this study. Eligible participants will be read a verbal consent script (Appendix B) and be presented with a study fact sheet (Appendix C) that will reinforce the consent script information. Depending on space availability, participant and research coordinator preferences, and whether in-person research activities are safe and permissible, informed consent will be obtained in one of the following ways: 1) “telephone booth” approach where the research coordinator would conduct the consent via Microsoft Teams or Enterprise Zoom on an encrypted laptop with the participant in a dedicated space on an encrypted study tablet; 2) meet in the clinic lobby, in a screened off area while maintaining at least 6 feet distance; 3) meet in an enclosed BHCHP clinic room while maintaining at least 6 feet distance if safe to do so; or 4) over the phone. The research coordinator will confirm participants’ understanding of the consent materials with basic knowledge questions about the information presented to them to ensure that they have the capacity to provide informed consent. Individuals who are able to correctly answer these questions will be asked to give their verbal consent to participant, which will be recorded by the research coordinator. Participants who provide informed consent over the phone will be given the option to receive a copy of the study fact sheet via picking up in-person at the JYP clinic, via mail if the participant has a mailing address, via secure email, or via email without send secure after obtaining participant’s permission.

40 participants who agree to participate in the post-intervention qualitative interviews will participate in a second verbal informed consent process at least 6 months after their initial enrollment. The research coordinator will read a separate verbal consent script (Appendix G) that reinforces the information presented in a fact sheet (Appendix F) that will be distributed to participants. The fact sheet will focus on the purpose, requirements, risks, and benefits of participating in the qualitative interview. The research coordinator will confirm participants’ understanding of the consent materials with basic knowledge questions about the information presented to them to ensure that they have the capacity to provide informed consent. Individuals who are able to correctly answer these questions will be asked to give their verbal consent to participant, which will be recorded by the research coordinator.

Additionally, we will obtain verbal consent to participate in qualitative interviews from 10 BHCHP PCPs who had at least 1 patient enrolled in the trial. Similar to the participants who participate in the interviews, the providers will be read a verbal consent script (Appendix I) that coincides with a fact sheet (Appendix H) that will be distributed to them. The research coordinator will record the providers’ verbal consent to participate in the interview.

We believe the verbal consent is justified for several reasons:

1) The experimental intervention, patient navigation, is a low-risk and widely-recommended behavioral intervention strategy with a sound evidence base in promoting cancer screening in vulnerable populations. Indeed, patient navigation has been endorsed by the ACS[64] and the National Cancer Institute[65] as a valuable approach to reducing cancer health disparities,[65] and is now part of routine care in some practice settings, including at MGH-affiliated health centers.

2) The outcome that the navigator is facilitating – obtaining LDCT for LCS among individuals at increased risk for lung cancer – is concordant with the guideline recommendations of the USPSTF,[20] ACS,[70] and multiple other professional groups.[71-74]

3) The study procedures present no more than minimal risk, as outlined below in section VII.

In order to ascertain the primary and secondary study outcomes of LDCT completion and LDCT completion with timely diagnostic follow-up (see section VII.B.), we will review BHCHP and MGB records for LDCT results in addition to obtaining written authorization from all enrolled participants to obtain their health/medical records for the 14 months following their trial enrollment date from other Boston-area hospitals and health care facilities using a HIPAA-compliant Release of Information (ROI) form which has been vetted by privacy officers at BHCHP, Boston Medical Center, and MGH (Appendix N). Participants who provide verbal consent during an in-person enrollment appointment will also complete the ROI form in person. Participants with a stable home address who complete the enrollment appointment remotely will be given the option to receive a pre-stamped envelope with the ROI form to have them sign and return back by mail to our MGH or BHCHP office.

If COVID-19 prompts further restrictions around in-person research activities at BHCHP, the research coordinator will transition to completing the verbal consent process over the phone or via the HIPAA compliant video conferencing platforms, Microsoft Teams or Enterprise Zoom depending on each participant's preference and access to technology. Based on feedback from the Boston Medical Center Compliance and Privacy Office, obtaining participant medical records for research purposes will continue to require written authorization regardless of current restrictions. Therefore, as noted previously, the research coordinator will offer participants with a stable home address the option to receive a pre-stamped envelope with the ROI form to sign and mail back. For participants who cannot receive a mailed ROI form, we will need to wait until in-person research restrictions are lifted. In this circumstance, once safe to do so, a research coordinator will either directly contact these research participants by phone or attempt to meet them in person at a clinic site to complete the form in person.

D. Treatment assignment

After providing informed consent and completing a baseline survey (see section V. A.), participants will be block-randomized in a 2:1 ratio to usual care with (N=200) or without (N=100) LCS navigation. The randomization scheme will be concealed from the study coordinator responsible for randomizing participants. Randomization will be stratified by smoking status (current/former), housing status (homeless/housed), prior discussion of lung cancer screening with their PCP, and primary clinical site where the patient sees their PCP (JYP vs. non-JYP sites) to ensure balance between study arms on these variables. We will stratify by smoking status (determined based on responses to the eligibility screener) because lung cancer risk perceptions and LCS motivations may differ between current and former smokers.[75,76] Current smoking will be defined as having smoked all or part of a cigarette in the past 30 days.[77] We will stratify by housing status based on responses to various items within the baseline survey because the navigation needs of formerly homeless people in housing may differ from those who are currently homeless. Current homelessness will be defined in the following ways: a) usually staying in an emergency shelter, transitional shelter, abandoned building, place of business, car or other vehicle, church or mission, hotel or motel, or anywhere outside during the past 7 days, b) usually housed in last 7 days but cannot stay in a residence for at least 14 days, c) usually institutionalized in the last 7 days if the admission was less than 90 days and they were homeless prior to admission, or d) usually institutionalized in the last 7 days if the admission was more than 90 days and they have no identified residence where they can stay at least 14 days upon discharge. These definitions are concordant with definitions of homelessness used by the US Department of Health and Human Services (section 330 of the Public Health Service Act, 42 USC § 254b)[78] and by the US Department of Housing and Urban Development (S. 896 HEARTH Act of 2009).[79] We will stratify based on prior discussion of lung

cancer screening with their PCP (defined by an item within the baseline survey) due to the fact that these participants are farther along in the lung cancer screening process. Finally, we will stratify by the patient's primary care clinical site since clinics may differ in their proximity to facilities that offer LCS or in their practice patterns around recommending and ordering LDCT for LCS. We will create 2 clinical site strata: 1 for JYP and one encompassing all of the 5 non-JYP study sites described above.

The 2 study arms are as follows:

Arm 1: Usual care ***without*** patient navigation (N=100). Participants assigned to this arm will be given basic educational materials on general lung health (Appendix K) and referred back to their PCP to discuss whether to pursue lung cancer screening per usual practice.

Arm 2: Usual care ***with*** patient navigation (N=200). Participants assigned to this arm will be informed about LCS, provided educational materials on LCS and patient navigation (Appendix L), and offered access to an LCS navigator. The navigator's principal role is to guide participants through the LCS process. The navigator's secondary role is to provide a brief tobacco intervention for participants who currently smoke. These roles are described below:

1) LCS navigation: The navigator will introduce the concept of LCS and assist participants in scheduling and attending a shared decision-making (SDM) visit with their PCP to discuss the test in greater depth. The navigator will proactively reach out to PCPs about this via electronic messaging through the BHCHP EHR and make him/herself available to assist in the LCS process. If a PCP and a participant jointly decide to pursue LCS, the navigator will assist with booking the LDCT appointment at a local facility that offers this test and interprets it according to the Lung-RADS framework.[80] BHCHP maintains strong clinical ties with Massachusetts General Hospital, Boston Medical Center, and several other area hospitals, enhancing the feasibility of referring patients to these facilities for LDCT. The navigator will work collaboratively with participants to reduce the barriers to LCS completion while building participants' self-efficacy. If a participant has difficulty obtaining LDCT because of lack of insurance, the navigator will connect the participant with a BHCHP benefits specialist to enroll in health insurance under the state's universal coverage system. Following LDCT, the navigator will coordinate a follow-up appointment with a participant's PCP to discuss the results and arrange any necessary follow-up testing. Throughout this process, the navigator will provide reminder phone calls about upcoming appointments, arrange for transportation assistance and other enabling services when needed, and reinforce the content and concepts covered in the LCS SDM visit through in-person or phone contact. Data from our survey of homeless smokers at BHCHP indicate that over 70% have mobile phones, which will facilitate communication between the navigator and study participants. In addition to documenting their encounters and activities with patients for research data collection purposes (see section VI.A.), the navigator will document within the patients' BHCHP EHR for their care teams to reference as needed.

The navigator will work with each participant for up to 6 months (26 weeks) to receive the initial LCS LDCT scan. If a participant receives their initial LCS LDCT scan within the 6-month (26-week) timeframe and requires follow-up diagnostic testing of abnormal results anytime sooner than the usual LCS LDCT screening interval of 12 months, the navigator will continue to work with the participant until the follow-up diagnostic testing is completed or until the participant no longer wishes to be navigated.

2) Brief tobacco intervention: The navigator will provide a brief tobacco intervention and distribute educational handouts on smoking cessation (on an ad hoc basis; Appendix M)

consistent with the “5 A’s” approach.[81] Within this framework, the navigator will Ask participants if they currently smoke cigarettes and Advise current smokers to quit, emphasizing the connection between smoking and lung cancer and the importance of quitting smoking to lower their lung cancer risk. The navigator will then Assess current smokers’ willingness to quit and Assist them in accessing no-cost or low-cost smoking cessation resources, including:

a) BHCHP PCPs, to discuss smoking cessation medications (e.g. nicotine replacement products, bupropion, or varenicline), which are covered by the state Medicaid program with a $1-3 copayment at retail pharmacies or at no charge through the BHCHP pharmacy. Over 80% of BHCHP patients are insured by the Massachusetts Medicaid program under the state’s universal health insurance system.[68]

b) BHCHP tobacco counselors, who are formally trained in tobacco counseling and conduct weekly support groups at 4 BHCHP venues and individual counseling sessions at 5 BHCHP venues, all free of charge.

c) The Massachusetts Smokers’ Helpline, a free and confidential phone counseling service, for participants with mobile phones. Participants who call the Massachusetts Smokers’ Helpline may receive up to 4 weeks of nicotine replacement therapy (patch, gum, or mini-lozenge) at no cost.

The navigator will then arrange a time to follow-up with current smokers either by phone or in person to discuss their progress on smoking and their engagement in available services. For participants who undergo LDCT, the navigator will conduct a follow-up brief intervention to head off false reassurance about smoking if the findings are negative. Brief tobacco interventions are an evidence-based, effective treatment approach to promoting smoking cessation.[81,82] The brief intervention strategy described above acknowledges the centrality of smoking cessation in reducing lung cancer deaths, capitalizes on the “teachable moment” afforded by the LCS process,[83] satisfies the CMS requirements for LCS LDCT approval,[21] and complements our pragmatic trial design by working within existing tobacco cessation resources available to the target population.

V. Study Procedures

A. Study visits and measurements

Participants who are screened and deemed eligible to participate, whether in person or over the phone, will be asked to complete a research visit during which enrollment, collection of baseline data, and randomization to study arm will occur. Participants will meet with the research coordinator in person at a BHCHP clinic site to complete these activities or complete these activities remotely. After verbal consent and the written ROI form is completed, a study coordinator will meet with the participant to administer a 30-minute baseline survey. In a similar fashion to the verbal consent, the survey can be completed in one of the following ways depending on current pandemic status and/or participant/staff preferences: a) “telephone booth” approach where the research coordinator would conduct the survey on an encrypted laptop via Microsoft Teams or Enterprise Zoom with the participant in a dedicated space with an encrypted study tablet; b) meet in the clinic lobby in a screened off area while maintaining at least 6 feet distance; c) meet in an enclosed BHCHP clinic room while maintaining at least 6 feet distance of safe to do so; or d) remotely, either over the phone if the participant has adequate access to their own phone and phone plan, or over secure video conferencing (Microsoft Teams or Enterprise Zoom) if the participant has adequate access to the necessary technology.

Participants will receive a one-time $25 gift card upon completion of the baseline survey in recognition of their time and effort. If participants complete the baseline survey over the phone, research staff will offer participants the chance to pick up the study gift card in-person at JYP or via mail if the participant has a mailing address.

Participants with a stable home address who complete enrollment and the baseline survey over the phone and who are not willing to travel to JYP to sign the ROI in person, will be given the option to receive a pre-stamped envelope with the ROI form to have them sign and mail back to our MGH or BHCHP office. Participants will be mailed the $25 gift card once the research coordinator receives a signed ROI.

Arm 2 participants will additionally have a series of ad hoc meetings with the LCS navigator as part of the intervention. These meetings may occur in person or over the phone. The navigator will be asked to log the date, time, and nature of activities carried out during these navigation sessions/meetings.

Due to the nature of the primary outcome, which will be ascertained via medical record review, no formal research follow-up visit is necessary. The screening, baseline, and outcome variables for the study are detailed in section VI.A.

40 participants in Arm 1 will participate in post-intervention interviews and be stratified according to whether they did (N=20) or did not (N=20) attain the primary outcome of completing a lung screening CAT scan within six months (26 weeks) of initial enrollment. Participants who agree to participate will go through a separate verbal consent process as illustrated previously. Interviews will occur after at least 6 months of exposure to the navigation intervention. The interviews will last up to 45 minutes and be audio recorded. The selection of qualitative participants following ascertainment of the primary quantitative outcome avoids introducing a treatment effect from the interviews while remaining temporally close enough to the LCS process to facilitate accurate recall. These 40 participants will receive a one-time $25 gift card upon completion of the interview in recognition of their time and effort.

Additionally, we will interview 10 BHCHP PCPs among those who had at least 1 patient enrolled in the trial. Providers who agree to participate will provide verbal consent as illustrated previously. Interviews will last up to 45 minutes and be audio recorded. Interviews will occur following completion of the trial (at least 6 months after the last patient participant is enrolled) to avoid influencing provider practice around LCS ordering and follow-up. The 10 PCPs will receive a one-time $25 gift card upon completion of the interview in recognition of their time and effort.

Study personnel will follow various safety precautions to prevent COVID-19 transmission. For patients that are screened for eligibility over the phone and interested in scheduling an in-person study visit, study personnel will administer a screening instrument for COVID-19 symptoms and recent positive COVID-19 tests within 72 hours prior to the scheduled in-person study visit. Prior to entering BHCHP clinic buildings, per BHCHP policy, all patients are already required to wear a face mask and be screened for COVID-19 symptoms and recent positive COVID-19 tests at the door. If BHCHP ceases these screening procedures, study personnel will implement the same screening measures at the start of any in-person visit and will continue to require participants to wear a face mask while engaging with study staff. If a patient endorses any COVID-19 symptoms, study personnel will immediately cease study activities and notify a clinic staff of the presence of a symptomatic patient.

If rising COVID-19 rates necessitate temporary restrictions on all in-person study activities at BHCHP facilities, we will transition to remotely conducting as many study activities as possible until the restrictions are lifted. Study personnel will be able to screen, perform verbal informed consent, conduct the baseline survey, perform qualitative interviews, and provide navigation services over the phone or by the HIPAA-compliant video conferencing platforms Microsoft Teams or Enterprise Zoom depending on patient preference and access to technology (i.e., owning a device with adequate minutes or video conferencing capabilities). Study personnel will always follow the MGB requirements for using Teams or Zoom with no study visits or activities being audio- or video-recorded within the Teams or Zoom platform. Microsoft Teams will be the preferred platform to use for video call study visits, but in the event of technical difficulties, study personnel will use Enterprise Zoom as a backup platform.

B. Drugs

While the LCS navigator will assist participants who currently smoke with accessing smoking cessation resources, neither the navigator nor study staff will provide participants with medications. Participants in both arms may choose to seek smoking cessation medications through their BHCHP PCP or other health care provider.

C. Devices

No devices will be used in this study.

D. Procedures

There are no procedures or surgical interventions in this study. Participants may elect to undergo LCS with LDCT after consultation with their PCP (and with the LCS navigator, for those in Arm 2), but the ordering and oversight of LDCTs will be handled by BHCHP PCPs as per usual practice for participants in both study arms.

E. Data Collection

We will collect all study data electronically using Partners-approved laptops or tablet devices with full disk encryption. Participant data will be recorded, saved, and managed using the Partners-hosted Research Electronic Data Capture (REDCap) program with data collection forms custom-designed for this study. We will also use BHCHP and MGB EHR to collect information from participants' medical records that relates to their general health history, any charted mental health problems, and any other information that directly relates to lung cancer screening and smoking. The specific data elements collected at each study visit are detailed below in section VI.A.

VI. BIOSTATISTICAL ANALYSIS

A. Specific data variables

1) Screening variables: Screening variables will include self-reported measures related to the inclusion and exclusion criteria for study participation. These will include the following variables, assessed as shown in Appendix A:
- First and last name
- Date of birth

- Age
- Preferred language
- BHCHP PCP
- Preferred BHCHP clinic site
- History of homelessness
- History or symptoms of lung cancer
- Recent non-lung cancer or cancer treatment
- Home oxygen use
- Cigarette smoking history

2) Contact form variables: Enrolled participants will be asked to complete a contact information form. This information will be valuable for following up with participants who cannot complete the baseline survey on the same day as enrollment, as well as for the patient navigator to aid with patient outreach and EHR documentation. This form will include the following variables, assessed as shown in Appendix O:
- BHCHP Epic MRN (entered by the research coordinator from patient's EHR)
- Last 4 digits of social security number
- Phone number (if applicable)
- Address/location of usual nighttime residence
- Alternative contact (who may know of participant's whereabouts)
- Contact in the case of an emergency
- Participant photograph (with participant permission) to facilitate subsequent contact with patient navigator

3) Baseline variables: Enrolled participants will be asked to complete a baseline assessment (Appendix D) of variables mapping to core domains of the Health Belief Model in addition to other key factors that could impact one or more dimensions of the Health Belief Model, as displayed in Table 2.

4) Electronic health record variables: The following information will be collected from the review of patients' BHCHP and MGB electronic health records:
- History of care with BHCHP and BHCHP PCP prior to study enrollment
- Comorbid health conditions.
- Documentation of a shared decision making (SDM) appointment with PCP to discuss lung cancer screening.
- Documentation of referrals for lung CT scans.
- Documentation of completion of lung CT scans
- Documentation of follow-up appointments with PCP that address results of lung cancer screening.
- Documented smoking status (and any changes in smoking status during study enrollment).
- Documented prescriptions to any smoking cessation medications
- Documented insurance status and insurance type.

Table 2. Baseline measures of study participants corresponding to Health Belief Model (HBM) domains and other key factors.

HBM domains	Measures
**Sociodemographic factors**	Age; sex; race/ethnicity; education; (all based on items from the 2003 HCH User Survey[23,84] and our prior survey work with homeless individuals[33,34]); homelessness onset, episodes, and duration[1]; current living situation
**Perceived lung cancer susceptibility/severity**	Perceived risk of lung cancer and smoking, perceived severity of lung cancer, worry about lung cancer, (all developed by Dr. Park for NLST)[76,85];
**Cues for LCS**	Prior LCS recommendation by health care provider; prior exposure to LCS brochures, flyers, posters, or advertisements
**Perceived LCS benefits/barriers**	Lung cancer screening knowledge[86]; perceived benefits of quitting smoking[76,85], comfort navigating hospital/health system; health insurance status[23,84]; competing priorities for meeting subsistence needs[22]
**Self-efficacy for LCS**	General self-efficacy scale[87,88]; self-efficacy for completing LDCT
**Other key factors**	**Measures**
**Tobacco use**	Duration of smoking; past quit attempts; current cigarettes/day; alternative tobacco use[35,89,90], Fagerstrom Test for Nicotine Dependence[91]; stage of change[92,93]; Contemplation Ladder[94]
**COVID-19**	Comfort navigating hospital/attending medical appointment during COVID-19 outbreak[95-97]; Previous history of COVID-19 And Lung Infection/Pneumonia
**Comorbid conditions**	Alcohol Use Disorders Identification Test-Concise (AUDIT-C)[98,99];! Drug Abuse Screening Test (DAST-10)[100,101], and K6 screen for psychological distress[102,103]; single-item general health status (SF-1)[104,105]

3) Navigation log: The navigator will complete a log (Appendix E) capturing the following information each time he/she interacts with a participant in Arm 2.

- Name of study staff who completes navigation activity
- Date and time
- Participant ID number
- Who was contacted for navigation activity
- Contact modality for navigation activity
- Tasks/activities undertaken during encounter

4) Qualitative interview data elements:

Participant interviews: Participants will be asked to articulate their thought process in considering whether to pursue LCS and to identify the factors that most shaped their decision, including logistical, physical, or emotional barriers and facilitators. Based on our Health Belief Model framework (Table 2), we will prompt participants to consider the impact of the navigator on a) altering their perceived susceptibility to lung cancer, b) providing them with cues or reminders to pursue LCS, c) enhancing their self-efficacy for pursuing LCS, and d) helping them to overcome barriers to completing LCS. Among participants who undergo LDCT, we will

assess their perspectives on the ease of the process, its impact on lung cancer worry, perceived lung cancer risk, and smoking behavior, and the need for any follow-up testing.

PCP interviews: These interviews will elicit PCP perspectives on the challenges of implementing LCS guidelines in primary care practice for a vulnerable population and probe the ways in which the navigator did or did not help to reduce these challenges. We will also seek feedback from PCPs on ways to strengthen the integration of patient navigators into clinical care teams.

<u>B. Study Endpoints</u>

1) Primary outcome: The primary outcome is receipt of LDCT for LCS at 6 months (26 weeks). This will be based on radiology records verifying that a chest CT was performed for LCS and interpreted according to the Lung-RADS framework.[80] To ascertain this outcome, study staff will examine available health records from BHCHP, MGB, and any other institution for which the participant signs a release of information, as described above in section IV.C. At 6 months (26 weeks) after enrollment, radiology records will be requested/obtained from these facilities. Most Boston-area hospital records are accessible through Care Everywhere in the BHCHP version of Epic and through MGB Epic, supporting the feasibility of gathering this information in an efficient manner. This approach does not rely on participant self-report and therefore does not require in-person or phone follow-up to ascertain. For those verified to have undergone LDCT for LCS within the 6 month (26-week) timeframe, the Lung-RADS result along with any abnormal or incidental findings will be recorded.

2) Secondary outcome: Increasing LDCT completion will only reduce lung cancer mortality if individuals with abnormal results can undergo evaluation and treatment of early stage lung cancer in a timely fashion. In the setting of homelessness, a valid concern is whether participants with abnormal LDCT results can be recalled for diagnostic follow-up in a timely manner. To address this concern, our secondary outcome is receipt of LDCT with timely diagnostic follow-up of any abnormal results. To satisfy this composite outcome, participants must achieve the primary outcome (LDCT at 6 months (26 weeks) <u>and</u>, if the result is abnormal, also obtain the next recommended follow-up test within 1 month (4 weeks) of the advised timeframe, based on the Lung-RADS framework.[80] Lung-RADS category descriptions, recommended follow-up, associated probabilities of malignancy, and estimated general population prevalences are shown in Table 3.[104] As described above, we will obtain radiology records for participants who underwent LDCT for LCS to document the findings of the study, determine the Lung-RADS category associated with those findings, and ascertain the recommended diagnostic follow-up plan. Participants with Lung-RADS 1-2 findings do not require specific diagnostic action, but rather may continue with annual screening at 12 months. These individuals will automatically satisfy the secondary outcome. In contrast, Lung-RADS 3-4 findings signal an increased risk for malignancy and merit sooner-than-annual follow-up testing. These individuals will satisfy the secondary outcome only if they obtain the next recommended diagnostic study within 1 month (4 weeks) of the recommended timeframe. For example, if a participant has a Lung-RADS 3 finding for which

Table 3. Lung-RADS categories.

<table>
<tr><th>Category</th><th>Description</th><th>Management</th><th>Malignancy probability</th><th>Gen. pop. prevalence</th></tr>
<tr><td>1</td><td>Negative</td><td rowspan="2">Repeat LDCT 12 mos</td><td rowspan="2"><1%</td><td rowspan="2">90%</td></tr>
<tr><td>2</td><td>Benign</td></tr>
<tr><td>3</td><td>Probably benign</td><td>Repeat LDCT 6 mos</td><td>1-2%</td><td>5%</td></tr>
<tr><td>4A</td><td rowspan="2">Suspicious</td><td>Repeat LDCT 3 mos +/- PET/CT</td><td>5-15%</td><td>2%</td></tr>
<tr><td>4B/X</td><td>Chest CT, PET/CT, +/- biopsy</td><td>>15%</td><td>2%</td></tr>
</table>

*Adapted from the American College of Radiology

follow-up LDCT in 6 months is recommended, we will assess whether this follow-up test occurred within 7 months (30 weeks) of the initial LDCT. If a participant has a Lung-RADS 4B/X finding suggesting a high suspicion for malignancy, we will assess whether the recommended follow-up testing occurred within 1 month (4 weeks) of the initial LDCT. The offer of delayed navigation to control arm participants will not interfere with this outcome because a) delayed navigation will be offered only to those who did not undergo an initial LCS LDCT within 6 months (26 weeks) (i.e. the primary outcome), and b) attaining the primary outcome is necessary for satisfying the secondary outcome.

3) Other outcomes: We will examine time to receipt of LDCT for LCS. Process outcomes include acceptance of navigation when offered, number of contacts with the navigator, and attendance of a LCS SDM visit with a BHCHP PCP. Tobacco-related outcomes include receipt of a prescription for any smoking cessation medication and changes in clinically-recorded smoking status (i.e. quitting among current smokers or relapsing among former smokers), both ascertained by BHCHP medical record review. We will track the frequency and types of abnormal findings detected on LCS LDCT, any additional diagnostic testing or therapeutic changes pursued in response to these findings, and the percentage of participants diagnosed with lung cancer (based on radiology and BHCHP and MGB records review). We will also track diagnostic chest CT scans obtained for reasons other than LCS.

C. Statistical Methods

Quantitative analysis: LDCT completion at 6 months (26 weeks) (1° outcome) and LDCT completion with timely diagnostic follow-up (2°outcome) are both binary outcomes. In the principal analysis, we will assess the difference between the navigation and usual care arms on attainment of these outcomes using the Chi-square test. We will also use logistic regression models to examine the associations between treatment condition and outcomes including clinical site and other known predictors of outcomes to increase the precision of effect estimates. Additional analyses will examine time to receipt of LDCT for LCS between study arms using time-to-event methods (log-rank tests and Cox regression models). Although we expect to be underpowered to detect significant interactions, we will examine whether the effect of navigation varies by smoking status, housing status, or clinical site in an exploratory analysis.

Qualitative analysis: Qualitative interviews will be audiotaped and transcribed. We will use inductive content analysis methods to identify major and minor themes, and we will use deductive methods to strengthen, refine, and enhance understanding of identified themes.[105] Qualitative data coding will be performed by 2 trained study staff members using NVivo 11 software. The data coders will meet regularly with Drs. Baggett and Park to discuss the structural thematic framework, categories, and coding plan. We will resolve discrepancies through discussion and comparison to raw data. Coding will continue iteratively until a high level of reliability (Kappa>0.80) is established.

Integration with quantitative data: Based on a mixed methods approach,[106] we will use the qualitative interview data to interpret and explain the quantitative observations from the trial. This will aid in better understanding why overall LDCT completion was higher or lower than expected and why the navigation intervention did or did not improve these outcomes. Following

from our stratified sampling scheme, we will integrate the quantitative and qualitative data by assessing for thematic differences in the interview content of participants who did (N=20) and did not (N=20) attain the primary outcome. We will focus in particular on comparing and contrasting these two groups' views on the utility of the navigator with respect to the Health Belief Model domains of perceived susceptibility, cues, self-efficacy, and barriers, as described above. We will additionally incorporate the quantitative baseline measures tapping these Health Belief Model domains into integrated analyses. Table 4 illustrates the way in which these quantitative and qualitative data elements will be integrated and presented in a blended fashion.

Table 4. Proposed integration of quantitative and qualitative data.

Health Belief Model domains	Measurement (method)	LCS LDCT at 6 months	
		Yes (N=20)	No (N=20)
Perceived susceptibility / severity	Baseline survey (quan)	Lung cancer risk perception, severity, and worry scores	
	Post-intervention interview (qual)	Quotes/themes about navigator impact on perceived susceptibility/severity	
Cues	Baseline survey (quan)	Past provider recommendation for LCS, exposure to LCS information	
	Post-intervention interview (qual)	Quotes/themes about navigator provision of cues for LCS	
Self-efficacy	Baseline survey (quan)	General and LCS-specific self-efficacy scores	
	Post-intervention interview (qual)	Quotes/themes about navigator impact on self-efficacy	
Perceived benefits / barriers	Baseline survey (quan)	LCS knowledge, competing subsistence difficulty scores	
	Post-intervention interview (qual)	Quotes/themes about navigator assistance with barriers	

D. Power Analysis

The sample size justification is based on the primary outcome of LCS LDCT completion at 6 months (26 weeks). Internal BHCHP data suggests a presently negligible baseline rate of LCS LDCT. Allowing for improvement in this rate as LCS comes into wider practice, we will assume that 5% of usual care (Arm 1) participants will obtain an LDCT within 6 months (26 weeks). In our judgment, increasing this completion rate to 15% would represent a clinically meaningful change. We will have >80% power to detect such a difference with a sample size of 200 in the navigation arm (Arm 2) and 100 in the usual care arm (Arm 1).

VII. RISKS AND DISCOMFORTS

The potential risks and discomforts associated with this study are detailed below. The methods for mitigating these risks and monitoring the safety of participants are described in section IX.b.

A. Complications of surgical / non-surgical procedures

Not applicable.

B. Drug side effects and toxicities

Study staff will not be offering any drugs or over-the-counter products to participants.

C. Device complications

Not applicable.

D. Psychosocial (non-medical) risks

Potential risks to participants include psychological distress arising from the possibility of false-positive CT findings leading to additional diagnostic studies or procedures. Psychological and/or physical distress may also be brought about by discussing smoking and/or changing smoking behavior. Finally, because we will collect and record identifying information (e.g. name, date of birth, and social security number), there is a risk to privacy if the data are compromised. Section IX (Monitoring and Quality Assurance) describes our methods for safeguarding the data and privacy of participants. The potential risks of this study are reasonable in relation to the potential benefits of reducing the risk of dying from lung cancer.

E. Radiation risks

There is a slight physical risk associated with exposure to radiation during CT scanning, although the "low-dose" nature of the test is designed to help mitigate this risk. For this reason, LDCT LCS for individuals at increased risk of lung cancer is recommended by a variety of professional societies since the benefits of screening for early stage cancer vastly outweigh the risks presented by small radiation exposures.

VIII. POTENTIAL BENEFITS

A. Potential benefits to participating individuals

Potential benefits for participants include the opportunity to receive assistance in completing LCS, which has been associated with a 20% reduction in lung cancer mortality. Additionally, current smokers will be informed of and referred to various resources to help them quit smoking, which may confer numerous health and financial benefits.

B. Potential benefits to society

Considerable knowledge could be gained from this study. To our knowledge, this will be the first study to focus on LCS among homeless people and the first randomized trial of any cancer screening intervention in this population. By focusing on an understudied topic area pertaining to a common cause of death among homeless people, our study fills an important gap in the literature and could serve as a blueprint for improving cancer screening and outcomes in homeless health care settings, particularly in the 295 HCH programs that serve 890,000 people annually in the US.[107]

IX. MONITORING AND QUALITY ASSURANCE

A. Data monitoring

All data will be collected using password-protected laptops or tablets with full disk encryption. We will use the Partners-hosted Research Electronic Data Capture (REDCap) application for data collection and management. REDCap is a secure, HIPAA compliant web-based application hosted by Partners HealthCare Research Computing, Enterprise Research Infrastructure & Services (ERIS). Because a Partners username is required for logging in to REDCap, all activity on study documents is electronically logged and therefore traceable. All data will be collected according to a standardized protocol preprogrammed into REDCap, which has built-in functions providing real-time data entry validation to help ensure accuracy and completeness. Each data

collection form will have instructions or prompts for collecting the required data elements, and the response fields for each item will have appropriate ranges and formats to ensure that the data is entered in a valid way. Participants who agree to be photographed will have their photo taken using the same device on which the rest of the data is collected (encrypted laptop or tablet). The photograph will be uploaded immediately to the REDCap database and the original photograph file will be permanently deleted from the device.

The PI will review all study data weekly to monitor its integrity. Additionally, the PI, biostatistician, and/or data analyst will download the study data on a weekly basis from the Partners REDCap server and back it up to the PI's SFA on the Partners network, which is protected by the Partners IS firewall, backed up nightly, and accessible only to authorized study staff. Only the minimum necessary number of study staff will have access to this data. Data analyses will be conducted by authorized study staff on the data files residing on the PI's Partners SFA. The data will not be transferred to investigators outside the Partners system.

The qualitative interviews conducted post-intervention will be recorded by a microphone connected directly to a password-protected, Partners-approved laptop or tablet with full-disk encryption. Audio files will be backed up to the PI's SFA described above and then deleted from the portable device.

If study activities are administered to participants using Enterprise Zoom or Microsoft Teams, personnel will always follow the MGB requirements for using video conferencing for research activities with no study visits or activities being audio- or video-recorded, with the exception of qualitative interviews, which will be audio-recorded with an encrypted laptop or tablet microphone (and not via the audio-recording capabilities within the Zoom or Teams platform) as described above. Study personnel will always follow the Principles of Privacy and share the minimum necessary amount of information.

B. Safety monitoring

Because of the narrow range of risks of the proposed study, there will not be a Data and Safety Monitoring Board for this trial. Instead, the safety of participants will be monitored by the PI on a continual basis. Navigators will stay in close contact with BHCHP PCPs and clinical staff to ensure the safety and well-being of all participants.

To minimize the risk to privacy, we will adhere to the data management procedures described above to help ensure the safety and integrity of data collected about study participants. All identifying information will be saved securely within the REDCap database. Paper ROI forms will be scanned into the PI's SFA and paper copies retained in a locked cabinet in the PI's office. Only the minimum necessary number of study staff will have access to this data. At the conclusion of the study, personal identifiers will be removed from the dataset and all analyses will occur in a de-identified fashion.

To help ensure the safety of participants, adverse events monitoring will be handled in the manner described below.

C. Outcomes monitoring

Because of the low-risk nature of the intervention, we will not conduct a formal interim analysis of the data nor define rules for early stopping of the trial.

D. Adverse event reporting guidelines

We will adhere to the Partners policy statement on “Reporting Unanticipated Problems including Adverse Events.” Consistent with this policy, an adverse event will be defined as “any untoward or unfavorable medical occurrence in a human subject, including any abnormal sign, symptom, or disease, temporally associated with the subject’s participation in the research, whether or not considered related to the subject’s participation in the research.” The PI and his designated study staff will be responsible for the monitoring of adverse events in study participants.

Adverse events reported to the PI or other study staff will be documented in an adverse event report containing a description of the event, the date and time of onset, the date and time of resolution, the expectedness of the event, the relationship to the study, the seriousness of the event, and the action taken in response to the event. An adverse event will be deemed unexpected if the nature, severity, or frequency of the event is not consistent with a known or foreseeable risk given the research procedures, the characteristics of the subject population, or with an expected progression of an underlying condition. The following attribution scale will be used to describe the relationship of the adverse event to the study protocol: unrelated to the protocol, or possibly, probably, or definitely related to the protocol. A serious adverse event will be defined as an adverse event that results in death, is life-threatening, requires hospitalization, causes persistent or significant disability, or requires medical or surgical intervention.

A summary safety report detailing all adverse events and their handling will be included in annual study progress reports to the Partners IRB and the American Cancer Society. Unexpected adverse events of any severity that are at least possibly related to the research and suggest the research puts participants at increased harm will be reported to the Partners IRB within 5 working days or 7 calendar days. Serious adverse events that are unexpected and at least possibly related to the research will be assumed to suggest that the research puts participants at increased harm and will be reported to the Partners IRB within the same time frame. Any action resulting in a temporary or permanent suspension of this study will be reported to the American Cancer Society’s program official. Adverse event reports and annual safety summaries will be documented by study ID number; personal identifiers will not be included in these reports.

E. Protocol adherence

1) Research staff: The study coordinators will be trained on the data collection protocols described above. Their understanding of these protocols will be assessed by asking them to demonstrate competency in using the REDCap data collection forms.

2) LCS navigator: To ensure adherence to the navigation intervention strategy, the navigator will receive in-depth training on patient navigation and focused training on tobacco cessation counseling.

a) Navigation training: The navigator will receive training in the core skills of patient navigation, including establishing rapport with patients while maintaining appropriate professional boundaries, motivational interviewing, performing an initial assessment with patients to explore barriers to LCS, working with patients to overcome these barriers, educating patients about the LCS process, introducing the concept of shared decision-making (SDM) around LCS, and empowering participants to discuss the benefits and risks of LCS with their PCP. Training will also encompass an overview of lung cancer, the rationale behind LCS, and the practical steps involved in completing LDCT so that the navigator can better counsel

patients on what to expect from the LCS process. Additionally, the navigator will be trained in identifying and managing logistical hurdles to completing LCS, such as insurance coverage lapses that may need to be remedied before LDCT is feasible. To facilitate timely follow-up of abnormal LDCT results, the navigator will received focused education on the American College of Radiology (ACR) Lung Imaging Reporting and Data System (Lung-RADS) framework.[80] Lung-RADS is a quality assurance tool designed to standardize LCS LDCT reporting and management recommendations, reduce confusion in LCS LDCT interpretations, and facilitate outcome monitoring.[80] The navigator training will be based on the protocol developed for Dr. Percac-Lima 's successful RCT of patient navigation for LCS among community health center patients (see section E). Dr. Percac-Lima, who has extensive experience in navigator training, will provide key components of this training and facilitate role playing sessions to reinforce skills. To the greatest extent possible, experienced navigators will be included in the training process. Depending upon prior experience, the navigator trainee will spend up to 2 weeks shadowing and observing an experienced navigator in the field prior to the start of the study. Refresher training and review of specific case scenarios will occur every 3 months throughout the study to ensure that the navigator is adhering faithfully to the intended roles and responsibilities.

b) Tobacco counseling training: The goal of the tobacco training component is to equip the navigator with the skills to provide a brief tobacco intervention. To achieve this goal, the navigator will complete an online 9-module course titled "Basic Skills for Working with Smokers," sponsored by the University of Massachusetts Center for Tobacco Treatment Research & Training.[108] This course provides an introduction to the theory and practice of working with smokers in a health care setting. The navigator will additionally receive 4 hours of case-based teaching from Dr. Baggett on practical approaches to working with homeless smokers, followed by 6 hours of observing a certified Tobacco Treatment Specialist interact with and counsel smokers about tobacco cessation.

The navigator's adherence to their intended duties will be assessed during weekly team meetings and through periodic audit of their navigation encounter logs.

X. REFERENCES

1. Burt MR, Aron LY, Douglas T, et al. Homelessness: Programs and the People They Serve: Findings of the National Survey of Homeless Assistance Providers and Clients: Technical Report. In. Washington, D.C.: U.S. Department of Housing and Urban Development, Office of Policy Development and Research; 1999.
2. Baggett TP, Chang Y, Porneala BC, Bharel M, Singer DE, Rigotti NA. Disparities in Cancer Incidence, Stage, and Mortality at Boston Health Care for the Homeless Program. *Am J Prev Med.* 2015;49(5):694-702.
3. Lamont DW, Toal FM, Crawford M. Socioeconomic deprivation and health in Glasgow and the west of Scotland--a study of cancer incidence among male residents of hostels for the single homeless. *J Epidemiol Community Health.* 1997;51(6):668-671.
4. Hwang SW, Wilkins R, Tjepkema M, O'Campo PJ, Dunn JR. Mortality among residents of shelters, rooming houses, and hotels in Canada: 11 year follow-up study. *BMJ.* 2009;339:b4036.
5. Baggett TP, Hwang SW, O'Connell JJ, et al. Mortality among homeless adults in Boston: shifts in causes of death over a 15-year period. *JAMA Intern Med.* 2013;173(3):189-195.
6. Baggett TP, Chang Y, Singer DE, et al. Tobacco-, alcohol-, and drug-attributable deaths and their contribution to mortality disparities in a cohort of homeless adults in Boston. *Am J Public Health.* 2015;105(6):1189-1197.
7. Aberle DR, Adams AM, Berg CD, et al. Reduced lung-cancer mortality with low-dose computed tomographic screening. *N Engl J Med.* 2011;365(5):395-409.
8. Chau S, Chin M, Chang J, et al. Cancer risk behaviors and screening rates among homeless adults in Los Angeles County. *Cancer Epidemiol Biomarkers Prev.* 2002;11(5):431-438.
9. Lebrun-Harris LA, Baggett TP, Jenkins DM, et al. Health status and health care experiences among homeless patients in federally supported health centers: findings from the 2009 patient survey. *Health services research.* 2013;48(3):992-1017.
10. Asgary R, Garland V, Jakubowski A, Sckell B. Colorectal cancer screening among the homeless population of New York City shelter-based clinics. *Am J Public Health.* 2014;104(7):1307-1313.
11. Asgary R, Garland V, Sckell B. Breast Cancer Screening Among Homeless Women of New York City Shelter-Based Clinics. *Womens Health Issues.* 2014.
12. Long HL, Tulsky JP, Chambers DB, et al. Cancer screening in homeless women: attitudes and behaviors. *J Health Care Poor Underserved.* 1998;9(3):276-292.
13. Baggett TP, Rigotti NA. Cigarette smoking and advice to quit in a national sample of homeless adults. *American Journal of Preventive Medicine.* 2010;39(2):164-172.
14. Connor SE, Cook RL, Herbert MI, Neal SM, Williams JT. Smoking cessation in a homeless population: there is a will, but is there a way? *Journal of General Internal Medicine.* 2002;17(5):369-372.
15. Snyder LD, Eisner MD. Obstructive lung disease among the urban homeless. *Chest.* 2004;125(5):1719-1725.
16. Szerlip MI, Szerlip HM. Identification of cardiovascular risk factors in homeless adults. *Am J Med Sci.* 2002;324(5):243-246.
17. Tsai J, Rosenheck RA. Smoking Among Chronically Homeless Adults: Prevalence and Correlates. *Psychiatr Serv.* 2012;63(6):569-576.
18. Torchalla I, Strehlau V, Okoli CT, Li K, Schuetz C, Krausz M. Smoking and predictors of nicotine dependence in a homeless population. *Nicotine Tob Res.* 2011;13(10):934-942.
19. Hahn JA, Kushel MB, Bangsberg DR, Riley E, Moss AR. BRIEF REPORT: the aging of the homeless population: fourteen-year trends in San Francisco. *J Gen Intern Med.* 2006;21(7):775-778.

20. Moyer VA. Screening for lung cancer: U.S. Preventive Services Task Force recommendation statement. *Ann Intern Med.* 2014;160(5):330-338.
21. Jenson TS, Chin J, Ashby L, Hermansen J, Hutter JD. Decision Memo for Screening for Lung Cancer with Low Dose Computed Tomography (LDCT) (CAG-00439N). . In. Baltimore, MD: Centers for Medicare & Medicaid Services; 2015.
22. Gelberg L, Gallagher TC, Andersen RM, Koegel P. Competing priorities as a barrier to medical care among homeless adults in Los Angeles. *Am J Public Health.* 1997;87(2):217-220.
23. Baggett TP, O'Connell JJ, Singer DE, Rigotti NA. The unmet health care needs of homeless adults: a national study. *Am J Public Health.* 2010;100(7):1326-1333.
24. Kushel MB, Vittinghoff E, Haas JS. Factors associated with the health care utilization of homeless persons. *JAMA.* 2001;285(2):200-206.
25. Weinstein LC, LaNoue M, Hurley K, Sifri R, Myers R. Using Concept Mapping to Explore Barriers and Facilitators to Breast Cancer Screening in Formerly Homeless Women with Serious Mental Illness. *J Health Care Poor Underserved.* 2015;26(3):908-925.
26. Fazel S, Khosla V, Doll H, Geddes J. The prevalence of mental disorders among the homeless in western countries: systematic review and meta-regression analysis. *PLoS Med.* 2008;5(12):e225.
27. Koegel P, Burnam MA, Farr RK. The prevalence of specific psychiatric disorders among homeless individuals in the inner city of Los Angeles. *Arch Gen Psychiatry.* 1988;45(12):1085-1092.
28. Brown RT, Kiely DK, Bharel M, Mitchell SL. Geriatric syndromes in older homeless adults. *J Gen Intern Med.* 2011;27(1):16-22.
29. Topolovec-Vranic J, Ennis N, Colantonio A, et al. Traumatic brain injury among people who are homeless: a systematic review. *BMC Public Health.* 2012;12:1059.
30. Topolovec-Vranic J, Ennis N, Howatt M, et al. Traumatic brain injury among men in an urban homeless shelter: observational study of rates and mechanisms of injury. *CMAJ Open.* 2014;2(2):E69-76.
31. Hwang SW, Colantonio A, Chiu S, et al. The effect of traumatic brain injury on the health of homeless people. *CMAJ.* 2008;179(8):779-784.
32. Oddy M, Moir JF, Fortescue D, Chadwick S. The prevalence of traumatic brain injury in the homeless community in a UK city. *Brain Inj.* 2012;26(9):1058-1064.
33. Baggett TP, Campbell EG, Chang Y, Magid LM, Rigotti NA. Posttraumatic Stress Symptoms and Their Association With Smoking Outcome Expectancies Among Homeless Smokers in Boston. *Nicotine Tob Res.* 2015.
34. Baggett TP, Rigotti NA, Campbell EG. Cost of Smoking among Homeless Adults. *N Engl J Med.* 2016;374(7):697-698.
35. Baggett TP, Campbell EG, Chang Y, Rigotti NA. Other tobacco product and electronic cigarette use among homeless cigarette smokers. *Addict Behav.* 2016;60:124-130.
36. Percac-Lima S, Ashburner JM, Bond B, Oo SA, Atlas SJ. Decreasing disparities in breast cancer screening in refugee women using culturally tailored patient navigation. *J Gen Intern Med.* 2013;28(11):1463-1468.
37. Percac-Lima S, Lopez L, Ashburner JM, Green AR, Atlas SJ. The longitudinal impact of patient navigation on equity in colorectal cancer screening in a large primary care network. *Cancer.* 2014;120(13):2025-2031.
38. Percac-Lima S, Ashburner JM, Zai AH, et al. Patient Navigation for Comprehensive Cancer Screening in High-Risk Patients Using a Population-Based Health Information Technology System: A Randomized Clinical Trial. *JAMA Intern Med.* 2016;176(7):930-937.
39. Freeman HP. Patient navigation: a community based strategy to reduce cancer disparities. *J Urban Health.* 2006;83(2):139-141.

40. Asgary R, Naderi R, Wisnivesky J. Opt-Out Patient Navigation to Improve Breast and Cervical Cancer Screening Among Homeless Women. *J Womens Health (Larchmt).* 2017.
41. Braun KL, Thomas WL, Jr., Domingo JL, et al. Reducing cancer screening disparities in medicare beneficiaries through cancer patient navigation. *J Am Geriatr Soc.* 2015;63(2):365-370.
42. Myers RE, Sifri R, Daskalakis C, et al. Increasing colon cancer screening in primary care among African Americans. *J Natl Cancer Inst.* 2014;106(12).
43. Honeycutt S, Green R, Ballard D, et al. Evaluation of a patient navigation program to promote colorectal cancer screening in rural Georgia, USA. *Cancer.* 2013;119(16):3059-3066.
44. Wang X, Fang C, Tan Y, Liu A, Ma GX. Evidence-based intervention to reduce access barriers to cervical cancer screening among underserved Chinese American women. *J Womens Health (Larchmt).* 2010;19(3):463-469.
45. Paskett ED, Harrop JP, Wells KJ. Patient navigation: an update on the state of the science. *CA Cancer J Clin.* 2011;61(4):237-249.
46. Phillips CE, Rothstein JD, Beaver K, Sherman BJ, Freund KM, Battaglia TA. Patient navigation to increase mammography screening among inner city women. *J Gen Intern Med.* 2010;26(2):123-129.
47. Robinson-White S, Conroy B, Slavish KH, Rosenzweig M. Patient navigation in breast cancer: a systematic review. *Cancer Nurs.* 2010;33(2):127-140.
48. Wells KJ, Battaglia TA, Dudley DJ, et al. Patient navigation: state of the art or is it science? *Cancer.* 2008;113(8):1999-2010.
49. Chen LA, Santos S, Jandorf L, et al. A program to enhance completion of screening colonoscopy among urban minorities. *Clin Gastroenterol Hepatol.* 2008;6(4):443-450.
50. Jandorf L, Braschi C, Ernstoff E, et al. Culturally targeted patient navigation for increasing african americans' adherence to screening colonoscopy: a randomized clinical trial. *Cancer Epidemiol Biomarkers Prev.* 2013;22(9):1577-1587.
51. Lasser KE, Murillo J, Medlin E, et al. A multilevel intervention to promote colorectal cancer screening among community health center patients: results of a pilot study. *BMC Fam Pract.* 2009;10:37.
52. Christie J, Itzkowitz S, Lihau-Nkanza I, Castillo A, Redd W, Jandorf L. A randomized controlled trial using patient navigation to increase colonoscopy screening among low-income minorities. *J Natl Med Assoc.* 2008;100(3):278-284.
53. Jandorf L, Gutierrez Y, Lopez J, Christie J, Itzkowitz SH. Use of a patient navigator to increase colorectal cancer screening in an urban neighborhood health clinic. *J Urban Health.* 2005;82(2):216-224.
54. Lasser KE, Murillo J, Lisboa S, et al. Colorectal cancer screening among ethnically diverse, low-income patients: a randomized controlled trial. *Archives of internal medicine.* 2011;171(10):906-912.
55. Horne HN, Phelan-Emrick DF, Pollack CE, et al. Effect of patient navigation on colorectal cancer screening in a community-based randomized controlled trial of urban African American adults. *Cancer Causes Control.* 2015;26(2):239-246.
56. Percac-Lima S, Grant RW, Green AR, et al. A culturally tailored navigator program for colorectal cancer screening in a community health center: a randomized, controlled trial. *J Gen Intern Med.* 2009;24(2):211-217.
57. Hoffman HJ, LaVerda NL, Young HA, et al. Patient navigation significantly reduces delays in breast cancer diagnosis in the District of Columbia. *Cancer Epidemiol Biomarkers Prev.* 2012;21(10):1655-1663.

58. Markossian TW, Darnell JS, Calhoun EA. Follow-up and timeliness after an abnormal cancer screening among underserved, urban women in a patient navigation program. *Cancer Epidemiol Biomarkers Prev.* 2012;21(10):1691-1700.
59. Battaglia TA, Bak SM, Heeren T, et al. Boston Patient Navigation Research Program: the impact of navigation on time to diagnostic resolution after abnormal cancer screening. *Cancer Epidemiol Biomarkers Prev.* 2012;21(10):1645-1654.
60. Raich PC, Whitley EM, Thorland W, Valverde P, Fairclough D. Patient navigation improves cancer diagnostic resolution: an individually randomized clinical trial in an underserved population. *Cancer Epidemiol Biomarkers Prev.* 2012;21(10):1629-1638.
61. Ramirez AG, Perez-Stable EJ, Penedo FJ, et al. Navigating Latinas with breast screen abnormalities to diagnosis: the Six Cities Study. *Cancer.* 2013;119(7):1298-1305.
62. Lee JH, Fulp W, Wells KJ, Meade CD, Calcano E, Roetzheim R. Patient navigation and time to diagnostic resolution: results for a cluster randomized trial evaluating the efficacy of patient navigation among patients with breast cancer screening abnormalities, Tampa, FL. *PLoS One.* 2013;8(9):e74542.
63. Lee JH, Fulp W, Wells KJ, Meade CD, Calcano E, Roetzheim R. Effect of patient navigation on time to diagnostic resolution among patients with colorectal cancer-related abnormalities. *J Cancer Educ.* 2014;29(1):144-150.
64. American Cancer Society. Patient Navigators Help Cancer Patients Manage Care. https://www.cancer.org/latest-news/navigators-help-cancer-patients-manage-their-care.html. Published February 24, 2017. Accessed March 1, 2017.
65. National Cancer Institute. Patient Navigation Research Program (PNRP). U.S. Department of Health and Human Services, National Institutes of Health, National Cancer Institute. https://www.cancer.gov/about-nci/organization/crchd/disparities-research/pnrp. Accessed March 28, 2017.
66. O'Connell JJ, Oppenheimer SC, Judge CM, et al. The Boston Health Care for the Homeless Program: a public health framework. *Am J Public Health.* 2010;100(8):1400-1408.
67. Bauer LK, Brody JK, Leon C, Baggett TP. Characteristics of Homeless Adults Who Died of Drug Overdose: A Retrospective Record Review. *J Health Care Poor Underserved.* 2016;27(2):846-859.
68. Bharel M, Lin WC, Zhang J, O'Connell E, Taube R, Clark RE. Health care utilization patterns of homeless individuals in Boston: preparing for Medicaid expansion under the Affordable Care Act. *Am J Public Health.* 2013;103 Suppl 2:S311-317.
69. Tsai D. MassHealth Transmittal Letter PHY-147. In: Office of Medicaid, Massachusetts Department of Health and Human Services; March 2016.
70. Wender R, Fontham ET, Barrera E, Jr., et al. American Cancer Society lung cancer screening guidelines. *CA Cancer J Clin.* 2013;63(2):107-117.
71. Detterbeck FC, Mazzone PJ, Naidich DP, Bach PB. Screening for lung cancer: Diagnosis and management of lung cancer, 3rd ed: American College of Chest Physicians evidence-based clinical practice guidelines. *Chest.* 2013;143(5 Suppl):e78S-92S.
72. Jaklitsch MT, Jacobson FL, Austin JH, et al. The American Association for Thoracic Surgery guidelines for lung cancer screening using low-dose computed tomography scans for lung cancer survivors and other high-risk groups. *J Thorac Cardiovasc Surg.* 2012;144(1):33-38.
73. Samet JH, Crowell R, Estepar RSJ, et al. Providing Guidance on Lung Cancer Screening to Patients and Physicians: An Update from the American Lung Association Lung Cancer Screening Committee. . In. Chicago: American Lung Association; April 30, 2015.

74. Bach PB, Mirkin JN, Oliver TK, et al. Benefits and harms of CT screening for lung cancer: a systematic review. *JAMA.* 2012;307(22):2418-2429.
75. Park ER, Streck JM, Gareen IF, et al. A qualitative study of lung cancer risk perceptions and smoking beliefs among national lung screening trial participants. *Nicotine Tob Res.* 2014;16(2):166-173.
76. Park ER, Ostroff JS, Rakowski W, et al. Risk perceptions among participants undergoing lung cancer screening: baseline results from the National Lung Screening Trial. *Ann Behav Med.* 2009;37(3):268-279.
77. Ryan H, Trosclair A, Gfroerer J. Adult current smoking: differences in definitions and prevalence estimates--NHIS and NSDUH, 2008. *J Environ Public Health.* 2012;2012:918368.
78. Health Resources and Services Administration, Bureau of Primary Health Care. Authorizing Legislation of the Health Center Program: Section 330 of the Public Health Service Act (42 USCS § 254b). http://bphc.hrsa.gov/policiesregulations/legislation/authorizing330.pdf. Accessed March 28, 2017.
79. U.S. Department of Housing and Urban Development. S. 896 HEARTH Act of 2009. https://www.hudexchange.info/resource/1717/s-896-hearth-act/. Accessed March 12, 2017.
80. American College of Radiology. Lung CT Screening Reporting and Data System (Lung-RADS). http://www.acr.org/Quality-Safety/Resources/LungRADS. Accessed March 28, 2017.
81. Fiore MC, Jaen CR, Baker TB, et al. Treating Tobacco Use and Dependence: 2008 Update. Clinical Practice Guideline. . In. Rockville, MD: U.S. Department of Health and Human Services. Public Health Service; May 2008.
82. Park ER, Gareen IF, Japuntich S, et al. Primary Care Provider-Delivered Smoking Cessation Interventions and Smoking Cessation Among Participants in the National Lung Screening Trial. *JAMA Intern Med.* 2015;175(9):1509-1516.
83. Poghosyan H, Kennedy Sheldon L, Cooley ME. The impact of computed tomography screening for lung cancer on smoking behaviors: a teachable moment? *Cancer Nurs.* 2012;35(6):446-475.
84. Greene J, Fahrney K, Byron M. Health Care for the Homeless User/Visit Surveys, RTI Project Number 07147.021. . In. Research Triangle Park, NC: RTI International; 2004.
85. Kaufman AR, Koblitz AR, Persoskie A, et al. Factor Structure and Stability of Smoking-Related Health Beliefs in the National Lung Screening Trial. *Nicotine Tob Res.* 2016;18(3):321-329.
86. Lowenstein LM, Richards VF, Leal VB, et al. A brief measure of Smokers' knowledge of lung cancer screening with low-dose computed tomography. *Prev Med Rep.* 2016;4:351-356.
87. Schwarzer R, Jerusalem M. Generalized Self-Efficacy scale. In: Weinman J, Wright S, Johnston M, eds. *Measures in health psychology: A user's portfolio. Causal and control beliefs.* Windsor, UK: NFER-NELSON; 1995:35-37.
88. Scholz U, Gutierrez Dona B, Sud S, Schwarzer R. Is General Self-Efficacy a Universal Construct? Psychometric Findings from 25 Countries. *European Journal of Psychological Assessment.* 2002;18(3):242-251.
89. US Department of Commerce CB. National Cancer Institute and Food and Drug Administration co-sponsored Tobacco Use Supplement to the Current Population Survey. In. *2018-2019*2020.
90. Kish DH, Reitzel LR, Kendzor DE, Okamoto H, Businelle MS. Characterizing Concurrent Tobacco Product Use Among Homeless Cigarette Smokers. *Nicotine Tob Res.* 2014.

91. Heatherton TF, Kozlowski LT, Frecker RC, Fagerstrom KO. The Fagerstrom Test for Nicotine Dependence: a revision of the Fagerstrom Tolerance Questionnaire. *Br J Addict.* 1991;86(9):1119-1127.
92. DiClemente CC, Prochaska JO, Fairhurst SK, Velicer WF, Velasquez MM, Rossi JS. The process of smoking cessation: an analysis of precontemplation, contemplation, and preparation stages of change. *J Consult Clin Psychol.* 1991;59(2):295-304.
93. Snow MG, Prochaska JO, Rossi JS. Stages of change for smoking cessation among former problem drinkers: a cross-sectional analysis. *J Subst Abuse.* 1992;4(2):107-116.
94. Biener L, Abrams DB. The Contemplation Ladder: validation of a measure of readiness to consider smoking cessation. *Health Psychol.* 1991;10(5):360-365.
95. Wong LE, Hawkins JE, Murrell KL. Where are all the patients? Addressing Covid-19 fear to encourage sick patients to seek emergency care. *NEJM Catalyst Innovations in Care Delivery.* 2020.
96. Toolkit P. COVID-19 Community Response Survey Guidance. In:2020.
97. (C3PNO) CCoCPNO. COVID-19 Module for NIDA-Supported Cohorts Affected by HIV and Substance Use. In:2020.
98. Bush K, Kivlahan DR, McDonell MB, Fihn SD, Bradley KA. The AUDIT alcohol consumption questions (AUDIT-C): an effective brief screening test for problem drinking. *Archives of internal medicine.* 1998;158(16):1789-1795.
99. Bradley KA, Bush KR, Epler AJ, et al. Two brief alcohol-screening tests From the Alcohol Use Disorders Identification Test (AUDIT): validation in a female Veterans Affairs patient population. *Archives of internal medicine.* 2003;163(7):821-829.
100. Skinner HA. The drug abuse screening test. *Addictive behaviors.* 1982;7(4):363-371.
101. Yudko E, Lozhkina O, Fouts A. A comprehensive review of the psychometric properties of the Drug Abuse Screening Test. *Journal of substance abuse treatment.* 2007;32(2):189-198.
102. Kessler RC, Andrews G, Colpe LJ, et al. Short screening scales to monitor population prevalences and trends in non-specific psychological distress. *Psychological medicine.* 2002;32(6):959-976.
103. Kessler RC, Barker PR, Colpe LJ, et al. Screening for serious mental illness in the general population. *Arch Gen Psychiatry.* 2003;60:184-189.
104. American College of Radiology. Lung-RADS Version 1.0 Assessment Categories. Available at http://www.acr.org/Quality-Safety/Resources/LungRADS. In:April 28, 2014.
105. Elo S, Kyngas H. The qualitative content analysis process. *J Adv Nurs.* 2008;62(1):107-115.
106. Creswell JW, Plano Clark VL. Chapter 3: Choosing a Mixed Methods Design. In: *Designing and Conducting Mixed Methods Research.* 2nd ed. Los Angeles: Sage; 2011:53-106.
107. Health Resources and Services Administration, U.S. Department of Health & Human Services. 2015 Health Center Data: National Health Care for the Homeless Program Grantee Data. https://bphc.hrsa.gov/uds/datacenter.aspx?year=2015&fd=ho. Accessed March 16, 2017.
108. Center for Tobacco Treatment Research & Training, University of Massachusetts Medical School. Basic Skills for Working with Smokers - Online Course. http://www.umassmed.edu/tobacco/training/basicskills_online/. Accessed March 28, 2017.

TITLE: Lung cancer screening navigation for homeless people: A pragmatic trial

PRINCIPAL INVESTIGATOR: Travis P. Baggett, MD, MPH

GRANT NUMBER: 131162-RSG-17-157-01-CPPB (American Cancer Society)

VERSION DATE: September 30, 2021

I. BACKGROUND AND SIGNIFICANCE

A. Historical background

An estimated 2.3-3.5 million people experience homelessness annually in the US.[1] Lung cancer incidence and mortality are substantially higher in this vulnerable population than in comparably-aged non-homeless individuals.[2-4] To address this disparity, we propose a pragmatic clinical trial to test the effect of patient navigation on lung cancer screening (LCS) completion among homeless-experienced adults at increased risk for lung cancer. Work that we and others have done suggests 2 reasons why such an intervention is needed.

1) Lung cancer is a major cause of death among homeless adults and the leading cause of tobacco-attributable death in this population.

In our study of 28,033 adults who used Boston Health Care for the Homeless Program (BHCHP) services in 2003-08, cancer was the second-leading cause of death and the leading killer of adults ≥45 years old.[5] Lung cancer accounted for one-third of these cancer deaths.[5] In a subsequent study of cancer epidemiology in this cohort, lung cancer was the leading type of incident cancer and the leading cause of cancer death among both men and women, with age-standardized incidence and mortality rates exceeding those in the Massachusetts adult population more than 2-fold.[2] An estimated 88% of incident lung cancer cases[2] and 93% of lung cancer deaths[6] were attributable to tobacco smoking, making lung cancer the leading type of smoking-attributable cancer and the leading cause of smoking-attributable death among homeless people. The excess burden of lung cancer in these studies of homeless adults in Boston was similar to that observed in studies of homeless and marginally housed people in Canada[4] and Glasgow[3] (Table 1), suggesting that these disparities are not geographically constrained. This body of evidence highlights the need for interventions to reduce lung cancer disparities in homeless populations. One avenue for doing so is LCS with low-dose computed tomography (LDCT) to promote detection and potentially curative treatment of early-stage lung cancer. This strategy was associated with a 20% reduction in lung cancer mortality in the National Lung Screening Trial (NLST).[7] However, implementing such a strategy presents formidable hurdles in the setting of homelessness.

Table 1. Lung cancer among homeless adults

Study location (yrs)	SIR/SMR (95% CI)
Boston (2003-2008)	
Incidence, men	2.30 (1.84, 2.84)
Incidence, women	2.23 (1.41, 3.35)
Mortality, men	2.39 (1.83, 3.08)
Mortality, women	2.31 (1.26, 3.88)
Canada (1991-2001)	
Mortality, men	1.91 (1.67, 2.18)
Mortality, women	1.73 (1.26, 2.36)
Glasgow (1975-1993)	
Incidence, men	1.64 (1.41, 1.86)

2) Homeless people have suboptimal rates of cancer screening and are diagnosed with screen-detectable malignancies at later stages than non-homeless people; barriers to implementing LCS in the setting of homelessness could exacerbate existing disparities in lung cancer outcomes.

In the 2003-08 BHCHP study of cancer epidemiology described above, homeless individuals were diagnosed with colorectal cancer and female breast cancer at significantly later stages than adults in the Massachusetts general population.[2] Several studies have documented low rates of screening for these malignancies among homeless individuals.[8-12] Given the high prevalence of smoking among homeless people[8,13-18] and the aging of the homeless population,[19] many homeless individuals are expected to meet the recommended criteria for consideration of LCS with LDCT[20,21]; however, as with colorectal and breast cancer screening, there are several obstacles to successfully implementing LCS in homeless populations. Homeless individuals struggling to meet basic subsistence needs may place a low priority on screening for asymptomatic illness[22] or may feel too emotionally overwhelmed to cope with the possibility of an abnormal result. Additionally, despite a high burden of cancer risk factors, most homeless people appear to believe that their susceptibility to cancer is equal to or less than that of others the same age.[8] Lack of insurance may pose another barrier,[23,24] although this is less of a concern in Massachusetts and could improve elsewhere in states that expanded Medicaid under the Affordable Care Act. Even among insured individuals who are interested in obtaining LDCT screening, the process of scheduling and attending the test may prove logistically challenging because of difficulty navigating complex facilities and care systems,[25] especially for the large proportions of homeless individuals with psychiatric and cognitive impairments.[26-32] Furthermore, ensuring timely follow-up of abnormal results may be challenging because of communication barriers and competing psychosocial issues. These barriers introduce the possibility that existing disparities in lung cancer mortality could worsen if homeless people are less able to complete LCS than other segments of the population.

B. Preliminary data

Our prior epidemiologic work demonstrating the burden of lung cancer disparities among homeless people is described above and provides the motivation for the proposed study. In addition, this study is informed by administrative data from BHCHP, survey and clinical trial studies with homeless smokers conducted by the PI (Dr. Baggett), navigation studies conducted by co-investigator Dr. Percac-Lima, and LCS-oriented studies conducted by co-investigators Dr. Park and Dr. Rigotti. Collectively, this preliminary data has demonstrated the following:

1) We have the patient volume to support this study. Based on internal data from the BHCHP Institute, 3,443 unique individuals aged 55-77 years old were seen at any BHCHP site from 9/1/2015 to 9/1/2016. Of these, 53% were current smokers and 23% were former smokers, totaling 2,616 potentially eligible individuals seen by the program in a 12-month period. When extrapolated over the planned 2.5-year enrollment period, we anticipate being able to recruit and randomize the proposed number of 300 participants.

2) We have characterized the target population. In April-July 2014, we used time-location sampling to conduct a survey of 306 homeless adult smokers at 5 high-volume BHCHP clinical sites.[33-35] The response rate was 86%. One-quarter of respondents (N=78) were 55-77 years old. Of these, the vast majority were non-Hispanic white or black men with a high school degree. Seventy percent had ever experienced a traumatic head injury, 64% had used any drug in the past month, 39% had consumed alcohol to intoxication in the past month, 60% reported feeling seriously depressed, and half screened positive for PTSD. One-third reported COPD and 17% reported cardiovascular disease. Past-month difficulty finding shelter, food, and clothing were common. The mean number of cigarettes per day was 12 and the mean Fagerstrom nicotine dependence score was 3.8 (range 0-12). These findings highlight the medical complexity and competing life issues of older homeless smokers and underscore the need for a navigation-based intervention to reduce the barriers to LCS.

3) We can recruit and longitudinally retain homeless people in a clinical trial. From October 2015 to June 2016, we conducted a 3-arm (N=25 per arm) pilot randomized controlled trial testing the effect of a) contingent financial rewards for smoking abstinence, and b) text messaging to support smoking abstinence, against c) a shared control condition, among homeless smokers at BHCHP (NCT02565381). Sixty-eight percent of eligible individuals enrolled, and we reached our target enrollment within 6 months. Study attendance and retention rates were excellent. Of 14 possible assessment visits, participants attended a median of 10, with 97% attending ≥1 visit and 77% attending ≥7 visits. These findings demonstrate that our team has the expertise to conduct a behavioral treatment trial in the context of homelessness and to engage and retain this vulnerable population in a longitudinal fashion. This is facilitated by our extensive knowledge of the target population, our person-centered approach to participant engagement, our strong partnership with BHCHP, and the central location of BHCHP headquarters with respect to other homeless services.

4) Our navigation intervention is based on a model that has proven effective at improving lung and other cancer screening among underserved patient populations in Boston. Key collaborator, Dr. Sanja Percac-Lima, has developed and tested navigation interventions that have been associated with improved cancer screening rates among diverse, low-income community health center patients in Boston.[36,37] She conducted a randomized controlled trial (RCT) across 18 clinical practices in an academic primary care network evaluating the impact of patient navigation relative to usual care for high-risk individuals who were overdue for breast, colorectal, and/or cervical cancer screening.[38] Patients assigned to navigation were significantly more likely to complete screening for breast (23.4% vs. 16.6%, $p=0.009$), cervical (14.4% vs. 8.6%, $p=0.007$), and colorectal (13.7% vs. 7.0%, $p<0.001$) cancer during the follow-up period.[38] More recently, with grant support from ACS and in collaboration with Drs. Park and Rigotti, Dr. Percac-Lima was the principal investigator of an RCT testing the effect of patient navigation on chest CT completion among low-income patients eligible for LCS at 5 MGH-affiliated health centers in greater Boston. Preliminary analysis of data after 11 months of follow-up has shown that participants in the navigation arm were significantly more likely to obtain a screening lung CT than participants assigned to usual care (24% vs. 9%, $p<0.01$). The LCS navigation approach used in our study will be based on these successful models.

<u>C. Study rationale</u>

Mitigating disparities in lung cancer outcomes and fully realizing the potential benefits of LCS among homeless people will require thoughtful interventions deployed through the clinical programs that serve this vulnerable population. Patient navigation, a strategy for guiding individuals through complex health systems,[39] may be a promising approach for helping homeless people overcome their unique barriers to LCS.[40] Navigation interventions have been shown to improve cancer screening participation[36,37,41-56] and diagnostic resolution of abnormal results[57-63] in vulnerable populations. Patient navigation has been endorsed by the ACS[64] and the National Cancer Institute[65] as a valuable approach to reducing cancer health disparities,[65] but this approach has not been tested rigorously in a homeless health care setting. Our research group is well-poised to conduct such a study by leveraging our extensive clinical and research experience with this population and our 9-year partnership with BHCHP, an internationally-renowned organization that serves over 12,000 currently (84%) and formerly (16%) homeless people annually through dozens of clinical sites in greater Boston.[66] This innovative community partnership has given rise to numerous impactful studies on the health of homeless-experienced people,[2,5,6,33-35,67] including a recently pilot trial for homeless smokers that had brisk recruitment, high retention rates, and encouraging results (NCT02565381).

II. SPECIFIC AIMS

Aim 1: To determine the effect of patient navigation, added to usual care, on 1°) LCS LDCT completion at 6 months (26 weeks) and 2°) LCS LDCT completion at 6 months (26 weeks) and diagnostic follow-up of abnormal results within 1 month (4 weeks) of the recommended timeframe, among homeless-experienced people who are eligible for LCS.

Hypothesis: Participants assigned to receive patient navigation will have significantly greater 1°) LCS LDCT completion at 6 months (26 weeks) and 2°) LCS LDCT completion at 6 months (26 weeks) with timely diagnostic follow-up of abnormal results.

Aim 2. To conduct post-intervention qualitative interviews of trial participants and BHCHP PCPs to explain and interpret the quantitative findings of the pragmatic clinical trial.

Based on a sequential explanatory mixed methods approach, Aim 2 interviews will examine how and why participants decided whether to undergo LCS, the barriers they encountered in doing so, the ways in which the navigator helped them overcome these barriers, and opportunities for improving the intervention for future use.

III. SUBJECT SELECTION

A. Inclusion and exclusion criteria

To be eligible to participate, individuals must meet **all** the following criteria:

1) Aged 55-77 years old, assessed by self-report and verified by date of birth.
   Rationale: LCS LCDT is covered only for patients aged 55-77.
2) Have a 30 pack-year smoking history *and* have smoked within the past 15 years.
   Rationale: We will be recruiting exclusively smokers because they are at the highest risk for lung cancer and therefore stand to receive the most benefit from a LCS. These smoking history parameters are consistent with current practice guidelines and Medicare eligibility criteria.
3) Have a BHCHP primary care provider (PCP).
   Rationale: Patient navigation is designed to support and extend services provided by medical practitioners. Having a PCP will help to ensure longitudinal medical oversight of the LCS process and appropriate follow-up of abnormal results.
4) Are currently or formerly homeless, assessed by self-report and defined as ever having experienced a time in their life where they did not have fixed, regular housing.
   Rationale: Both currently and formerly homeless individuals were included in our epidemiologic analysis showing a more than 2-fold higher incidence and mortality rate from lung cancer compared to the general Massachusetts adult population.[2] BHCHP and most HCH programs nationally continue to serve patients after they have gained housing.
5) Be proficient in English, assessed with items asking about native language and self-reported comfort communicating in English among non-native speakers.
   Rationale: Because of a budget reduction by the funding agency, we do not have the resources to develop study materials or conduct in-person navigation in languages other than English. Additionally, the vast majority of individuals in the target population speak English.

Individuals meeting **any** of the following criteria will be excluded from the study:

1) A recent prior chest computed tomography (CT) imaging (in the past 12 months).
   Rationale: This study is aimed at individuals who are most likely to benefit from LCS, based on evidence from the National Lung Screening Trial. Potential participants that have recently received a chest CT will have recently been scanned for lung cancer, therefore an additional screening will not benefit their health outcomes.
2) Any personal history of lung cancer, or current presentation with symptoms concerning for lung cancer (e.g. hemoptysis or unexplained weight loss of >15 lbs. in the past year).
   Rationale: Individuals with a history of lung cancer or symptoms concerning for lung cancer require surveillance or diagnostic lung imaging, respectively, and are not appropriate candidates for screening lung imaging, which by definition is designed for individuals without symptoms or history of the illness being screened for.
3) Inability to provide informed consent, assessed with knowledge questions about the material presented during the informed consent process that individuals must correctly answer before providing informed consent to participate.
   Rationale: In this vulnerable population, we wish to take a conservative approach to the informed consent process to help ensure that participants fully understand the pros and cons of participating in the research study.
4) PCP is the study PI (Dr. Travis Baggett).
   Rationale: BHCHP patients under the primary care of Dr. Baggett may feel unduly influenced to participate. In addition, since the primary study outcome (receipt of LDCT for LCS) requires PCP involvement, the inclusion of Dr. Baggett's patients creates a conflict of interest and the potential for bias.

B. Source of subjects and recruitment methods

This study will be registered with ClinicalTrials.gov prior to the recruitment and enrollment of human subjects. All participants will be recruited from BHCHP clinical sites. BHCHP does not have its own IRB but instead will rely on the Partners Human Research Committee for IRB review through a reliance agreement initiated through SMART IRB. In addition to being a faculty physician-investigator at Massachusetts General Hospital and Harvard Medical School, the study PI is the Director of Research at BHCHP and is very familiar with the clinical environment and patient population served by the program.

Individuals must be homeless to enroll in services at BHCHP; however, some patients continue receiving care at the program after they are no longer homeless. An internal analysis of individuals seen at BHCHP in 2011 found that 16% of patients were housed, most without supportive services and therefore at potentially high risk for recurrent homelessness. Because our epidemiologic analyses demonstrating the dramatic lung cancer disparities experienced by BHCHP patients included both currently and formerly homeless individuals, and because of the often cyclic nature of homelessness, we will include both currently and formerly homeless individuals in the proposed study. We refer to this group collectively as "homeless-experienced." Owing to the Massachusetts system of universal health insurance, about 80% of BHCHP patients are insured by the state Medicaid program,[68] which covers LCS LDCT among eligible beneficiaries,[69] and a sizable proportion of those remaining are covered by Medicare alone. This will help ensure the availability of LCS LDCT for the vast majority of potential participants.

Participants will be recruited through a combination of 3 methods:

1) In-person screening of patients at BHCHP clinical sites: Study personnel will screen patients in-person at 6 high-volume BHCHP clinic sites: a) Pine Street Inn, a 470-bed shelter where BHCHP operates a medical clinic 7 days/week in the men's shelter and 6 days/week in the women's shelter; b) Southampton Shelter, a 400-bed men's shelter where BHCHP operates a medical clinic 7 days/week; c) Woods Mullen Shelter, a 200-bed women's shelter where BHCHP operates a medical clinic 5 days/week; d) New England Center and Home for Veterans, a 209-bed emergency and transitional shelter where BHCHP operates a medical clinic 5 days/week; e) St. Francis House, a daytime multiservice facility for homeless people where BHCHP operates a medical clinic 5 days/week; and f) Jean Yawkey Place, the BHCHP headquarters facility where medical, behavioral health, and dental clinics operate 5 days/week with an on-site pharmacy and medical respite unit. We will also screen and recruit patients at selected other BHCHP clinical sites with the approval of their respective site directors and BHCHP medical officers. BHCHP operates at dozens of smaller clinical sites in the Boston area that likely reach the subsets of patient population that are eligible for the INHALE Study. Our prior survey fieldwork with this population has given us extensive experience with tactfully deploying this proactive, in-person recruitment technique.[33-35] In light of the COVID-19 pandemic, BHCHP clinical sites and shelters are experiencing a significant decrease in patient volume and a lack of additional space to safely conduct in-person recruitment and data collection activities. Under these circumstances, study personnel will initially focus in-person screening and data collection efforts at Jean Yawkey Place, which has the highest volume of patients and the greatest degree of flexibility in physical space and layout. This will allow the study personnel to have an in-person presence at the clinic while practicing physical distancing and prioritizing the health and well-being of participants and study staff. If COVID-19 restrictions loosen and patient volume increases at shelter sites, we will expand our efforts to include the five additional sites, depending on COVID-19 public health guidelines and facility protocols.

2) Review of BHCHP electronic records: BHCHP has a comprehensive EHR system (Epic) that prompts providers to assess tobacco use at every clinical encounter, facilitating determination of smoking history and current smoking status. Individuals with a BHCHP PCP who have been seen in a BHCHP clinic in the past year and who were current or former smokers at the time of their most recent clinical encounter will be approached in person at BHCHP clinical sites if they have a scheduled appointment or contacted proactively via phone (if they have listed contact information) by a research coordinator and screened for eligibility to participate. The BHCHP Chief Medical Officer has approved this recruitment strategy on behalf of all primary care providers at BHCHP clinical sites, who will in turn be notified about our recruitment approach prior to the start of the study (letter of approval in Appendix J).

3) Referrals from BHCHP providers: We will advertise the study to all BHCHP providers and in all BHCHP clinical sites. Individuals deemed by a BHCHP provider to be potential candidates for LCS can be referred to the research coordinator, who will screen referred patients for eligibility to participate.

IV. SUBJECT ENROLLMENT

An integrated study schema depicting recruitment, enrollment, treatment assignment, and outcomes assessment has been uploaded as an attachment to this application.

A. COVID-19 symptom and exposure screening

BHCHP staff currently screen all patients for COVID-19 symptoms or a recent positive COVID-19 test upon entry to BHCHP clinics and facilities. BHCHP is also providing patients with

facemasks before entering the building. Study personnel will re-screen participants using the same approach before conducting any research activities in an enclosed room. If BHCHP ceases their COVID-19 screening procedure, study personnel will use BHCHP's "COVID-19 Screening Tool for People Experiencing Homelessness" to screen all participants for current COVID-19 symptoms and past 14-day COVID-19 exposures (i.e., travel to high risk countries or states; unmasked exposure to a person with COVID-19). Study staff will wear appropriate personal protective equipment (PPE) per MGH guidelines and policies and remain 6 feet away from the patient. If patient endorses any COVID-19 symptoms or high-risk exposures, study personnel will immediately notify a clinic staff member of the presence of a symptomatic patient and will not engage in any study activities. Immediately after departure of the symptomatic patient, study personnel will disinfect surfaces that were within 6 feet of the symptomatic patient. If the participant reports not having symptoms, they can be directed to the study table/interview room. If the study coordinator makes initial contact with a participant over the phone, before scheduling an in-person visit, study personnel will screen the participant within 72 hours before the appointment. If participants report COVID-19 symptoms or exposures, study staff will request participants' permission to contact their BHCHP PCP via Epic messaging who can then advise the participant on next steps (e.g., testing and/or quarantine). If participants do not provide permission to study staff to contact their PCP, study staff will recommend that the participant seek care for their symptoms and/or exposure and offer to do the initial study procedures over the phone. If they are not interested in completing any further study procedures over the phone, study staff will call the participant again in 10 days to check-in and rescreen before attempting to schedule any study procedures in person. Participants will be advised to put on a facemask, regardless of symptoms, before coming in to meet with the research coordinator. Research personnel will instruct participants to notify them before arriving if they have fever or symptoms of COVID-19.

B. Methods of enrollment

Participants recruited through the above methods (section III.B) will be screened for eligibility either in-person or by phone. Prior to contacting participants, study staff will use the eligibility screener (Appendix A) to record information related to eligibility that was able to be collected during the prior review of prospective participants' BHCHP EHR (as referenced above).

Upon approaching the participants, study staff will use participant self-report to verify any information collected via their EHR and will collect the minimum amount of additional information necessary to assess and verify study eligibility. Eligible individuals identified through in-person screening will be offered the chance to enroll immediately. Those identified through phone screening will be asked to attend an in-person enrollment visit at one of clinic sites listed above. Participants will be asked to provide verbal informed consent before enrolling.

At least six months after initial enrollment, participants who were randomized to the patient navigation arm of the study will be contacted via phone or approached in person at their next BHCHP appointment to be offered the opportunity to participate in a 45-minute qualitative interview. Forty participants will be interviewed in total, with purposive sampling of participants who did (N=20) and participants who did not (N=20) attain the primary outcome of completing a lung screening CT scan within six months.

At approximately 6 months after the last patient participant is enrolled, providers who had at least one patient enrolled in the trial will be contacted via phone, email, or in person to be offered the opportunity to participate in a 45-minute qualitative interview. Ten participants will be

interviewed in total, with purposive sampling to ensure that at least one provider who works at each of the targeted BHCHP clinic sites are interviewed.

C. Informed consent procedures

We will obtain verbal informed consent for this study. Eligible participants will be read a verbal consent script (Appendix B) and be presented with a study fact sheet (Appendix C) that will reinforce the consent script information. Depending on space availability, participant and research coordinator preferences, and whether in-person research activities are safe and permissible, informed consent will be obtained in one of the following ways: 1) "telephone booth" approach where the research coordinator would conduct the consent via Microsoft Teams or Enterprise Zoom on an encrypted laptop with the participant in a dedicated space on an encrypted study tablet; 2) meet in the clinic lobby, in a screened off area while maintaining at least 6 feet distance; 3) meet in an enclosed BHCHP clinic room while maintaining at least 6 feet distance if safe to do so; or 4) over the phone. The research coordinator will confirm participants' understanding of the consent materials with basic knowledge questions about the information presented to them to ensure that they have the capacity to provide informed consent. Individuals who are able to correctly answer these questions will be asked to give their verbal consent to participant, which will be recorded by the research coordinator. Participants who provide informed consent over the phone will be given the option to receive a copy of the study fact sheet via picking up in-person at the JYP clinic, via mail if the participant has a mailing address, via secure email, or via email without send secure after obtaining participant's permission.

40 participants who agree to participate in the post-intervention qualitative interviews will participate in a second verbal informed consent process either at least 6 months after their initial enrollment or sooner if the participant had completed lung cancer screening and the navigation intervention was terminated as a result. The research coordinator will read a separate verbal consent script (Appendix G) that reinforces the information presented in a fact sheet (Appendix F) that will be distributed to participants. The fact sheet will focus on the purpose, requirements, risks, and benefits of participating in the qualitative interview. The research coordinator will confirm participants' understanding of the consent materials with basic knowledge questions about the information presented to them to ensure that they have the capacity to provide informed consent. Individuals who are able to correctly answer these questions will be asked to give their verbal consent to participant, which will be recorded by the research coordinator.

Additionally, we will obtain verbal consent to participate in qualitative interviews from 10 BHCHP PCPs who had at least 1 patient enrolled in the trial. Similar to the participants who participate in the interviews, the providers will be read a verbal consent script (Appendix I) that coincides with a fact sheet (Appendix H) that will be distributed to them. The research coordinator will record the providers' verbal consent to participate in the interview.

We believe the verbal consent is justified for several reasons:

1) The experimental intervention, patient navigation, is a low-risk and widely-recommended behavioral intervention strategy with a sound evidence base in promoting cancer screening in vulnerable populations. Indeed, patient navigation has been endorsed by the ACS[64] and the National Cancer Institute[65] as a valuable approach to reducing cancer health disparities,[65] and is now part of routine care in some practice settings, including at MGH-affiliated health centers.

2) The outcome that the navigator is facilitating – obtaining LDCT for LCS among individuals at increased risk for lung cancer – is concordant with the guideline recommendations of the USPSTF,[20] ACS,[70] and multiple other professional groups.[71-74]

3) The study procedures present no more than minimal risk, as outlined below in section VII.

In order to ascertain the primary and secondary study outcomes of LDCT completion and LDCT completion with timely diagnostic follow-up (see section VII.B.), we will review BHCHP and MGB records for LDCT results in addition to obtaining written authorization from all enrolled participants to obtain their health/medical records for the 14 months following their trial enrollment date from other Boston-area hospitals and health care facilities using a HIPAA-compliant Release of Information (ROI) form which has been vetted by privacy officers at BHCHP, Boston Medical Center, and MGH (Appendix N). Participants who provide verbal consent during an in-person enrollment appointment will also complete the ROI form in person. Participants with a stable home address who complete the enrollment appointment remotely will be given the option to receive a pre-stamped envelope with the ROI form to have them sign and return back by mail to our MGH or BHCHP office.

If COVID-19 prompts further restrictions around in-person research activities at BHCHP, the research coordinator will transition to completing the verbal consent process over the phone or via the HIPAA compliant video conferencing platforms, Microsoft Teams or Enterprise Zoom depending on each participant's preference and access to technology. Based on feedback from the Boston Medical Center Compliance and Privacy Office, obtaining participant medical records for research purposes will continue to require written authorization regardless of current restrictions. Therefore, as noted previously, the research coordinator will offer participants with a stable home address the option to receive a pre-stamped envelope with the ROI form to sign and mail back. For participants who cannot receive a mailed ROI form, we will need to wait until in-person research restrictions are lifted. In this circumstance, once safe to do so, a research coordinator will either directly contact these research participants by phone or attempt to meet them in person at a clinic site to complete the form in person.

D. Treatment assignment

After providing informed consent and completing a baseline survey (see section V. A.), participants will be block-randomized in a 2:1 ratio to usual care with (N=200) or without (N=100) LCS navigation. The randomization scheme will be concealed from the study coordinator responsible for randomizing participants. Randomization will be stratified by smoking status (current/former), housing status (homeless/housed), prior discussion of lung cancer screening with their PCP, and primary clinical site where the patient sees their PCP (JYP vs. non-JYP sites) to ensure balance between study arms on these variables. We will stratify by smoking status (determined based on responses to the eligibility screener) because lung cancer risk perceptions and LCS motivations may differ between current and former smokers.[75,76] Current smoking will be defined as having smoked all or part of a cigarette in the past 30 days.[77] We will stratify by housing status based on responses to various items within the baseline survey because the navigation needs of formerly homeless people in housing may differ from those who are currently homeless. Current homelessness will be defined in the following ways: a) usually staying in an emergency shelter, transitional shelter, abandoned building, place of business, car or other vehicle, church or mission, hotel or motel, or anywhere outside during the past 7 days, b) usually housed in last 7 days but cannot stay in a residence for at least 14 days, c) usually institutionalized in the last 7 days if the admission was less than 90 days and they were homeless prior to admission, or d) usually institutionalized in the last 7 days if the

admission was more than 90 days and they have no identified residence where they can stay at least 14 days upon discharge. These definitions are concordant with definitions of homelessness used by the US Department of Health and Human Services (section 330 of the Public Health Service Act, 42 USC § 254b)[78] and by the US Department of Housing and Urban Development (S. 896 HEARTH Act of 2009).[79] We will stratify based on prior discussion of lung cancer screening with their PCP (defined by an item within the baseline survey) due to the fact that these participants are farther along in the lung cancer screening process. Finally, we will stratify by the patient's primary care clinical site since clinics may differ in their proximity to facilities that offer LCS or in their practice patterns around recommending and ordering LDCT for LCS. We will create 2 clinical site strata: 1 for JYP and one encompassing all of the 5 non-JYP study sites described above.

The 2 study arms are as follows:

Arm 1: Usual care ***without*** patient navigation (N=100). Participants assigned to this arm will be given basic educational materials on general lung health (Appendix K) and referred back to their PCP to discuss whether to pursue lung cancer screening per usual practice.

Arm 2: Usual care ***with*** patient navigation (N=200). Participants assigned to this arm will be informed about LCS, provided educational materials on LCS and patient navigation (Appendix L), and offered access to an LCS navigator. The navigator's principal role is to guide participants through the LCS process. The navigator's secondary role is to provide a brief tobacco intervention for participants who currently smoke. These roles are described below:

1) LCS navigation: The navigator will introduce the concept of LCS and assist participants in scheduling and attending a shared decision-making (SDM) visit with their PCP to discuss the test in greater depth. The navigator will proactively reach out to PCPs about this via electronic messaging through the BHCHP EHR and make him/herself available to assist in the LCS process. If a PCP and a participant jointly decide to pursue LCS, the navigator will assist with booking the LDCT appointment at a local facility that offers this test and interprets it according to the Lung-RADS framework.[80] BHCHP maintains strong clinical ties with Massachusetts General Hospital, Boston Medical Center, and several other area hospitals, enhancing the feasibility of referring patients to these facilities for LDCT. The navigator will work collaboratively with participants to reduce the barriers to LCS completion while building participants' self-efficacy. If a participant has difficulty obtaining LDCT because of lack of insurance, the navigator will connect the participant with a BHCHP benefits specialist to enroll in health insurance under the state's universal coverage system. Following LDCT, the navigator will coordinate a follow-up appointment with a participant's PCP to discuss the results and arrange any necessary follow-up testing. Throughout this process, the navigator will provide reminder phone calls about upcoming appointments, arrange for transportation assistance and other enabling services when needed, and reinforce the content and concepts covered in the LCS SDM visit through in-person or phone contact. Data from our survey of homeless smokers at BHCHP indicate that over 70% have mobile phones, which will facilitate communication between the navigator and study participants. In addition to documenting their encounters and activities with patients for research data collection purposes (see section VI.A.), the navigator will document within the patients' BHCHP EHR for their care teams to reference as needed.

The navigator will work with each participant for up to 6 months (26 weeks) to receive the initial LCS LDCT scan. If a participant receives their initial LCS LDCT scan within the 6-month (26-week) timeframe and requires follow-up diagnostic testing of abnormal results anytime sooner than the usual LCS LDCT screening interval of 12 months, the navigator will continue to work

with the participant until the follow-up diagnostic testing is completed or until the participant no longer wishes to be navigated.

2) Brief tobacco intervention: The navigator will provide a brief tobacco intervention and distribute educational handouts on smoking cessation (on an ad hoc basis; Appendix M) consistent with the “5 A’s” approach.[81] Within this framework, the navigator will Ask participants if they currently smoke cigarettes and Advise current smokers to quit, emphasizing the connection between smoking and lung cancer and the importance of quitting smoking to lower their lung cancer risk. The navigator will then Assess current smokers’ willingness to quit and Assist them in accessing no-cost or low-cost smoking cessation resources, including:

a) BHCHP PCPs, to discuss smoking cessation medications (e.g. nicotine replacement products, bupropion, or varenicline), which are covered by the state Medicaid program with a $1-3 copayment at retail pharmacies or at no charge through the BHCHP pharmacy. Over 80% of BHCHP patients are insured by the Massachusetts Medicaid program under the state’s universal health insurance system.[68]

b) BHCHP tobacco counselors, who are formally trained in tobacco counseling and conduct weekly support groups at 4 BHCHP venues and individual counseling sessions at 5 BHCHP venues, all free of charge.

c) The Massachusetts Smokers’ Helpline, a free and confidential phone counseling service, for participants with mobile phones. Participants who call the Massachusetts Smokers’ Helpline may receive up to 4 weeks of nicotine replacement therapy (patch, gum, or mini-lozenge) at no cost.

The navigator will then arrange a time to follow-up with current smokers either by phone or in person to discuss their progress on smoking and their engagement in available services. For participants who undergo LDCT, the navigator will conduct a follow-up brief intervention to head off false reassurance about smoking if the findings are negative. Brief tobacco interventions are an evidence-based, effective treatment approach to promoting smoking cessation.[81,82] The brief intervention strategy described above acknowledges the centrality of smoking cessation in reducing lung cancer deaths, capitalizes on the “teachable moment” afforded by the LCS process,[83] satisfies the CMS requirements for LCS LDCT approval,[21] and complements our pragmatic trial design by working within existing tobacco cessation resources available to the target population.

V. Study Procedures

A. Study visits and measurements

Participants who are screened and deemed eligible to participate, whether in person or over the phone, will be asked to complete a research visit during which enrollment, collection of baseline data, and randomization to study arm will occur. Participants will meet with the research coordinator in person at a BHCHP clinic site to complete these activities or complete these activities remotely. After verbal consent and the written ROI form is completed, a study coordinator will meet with the participant to administer a 30-minute baseline survey. In a similar fashion to the verbal consent, the survey can be completed in one of the following ways depending on current pandemic status and/or participant/staff preferences: a) “telephone booth” approach where the research coordinator would conduct the survey on an encrypted laptop via Microsoft Teams or Enterprise Zoom with the participant in a dedicated space with an encrypted study tablet; b) meet in the clinic lobby in a screened off area while maintaining at least 6 feet

distance; c) meet in an enclosed BHCHP clinic room while maintaining at least 6 feet distance of safe to do so; or d) remotely, either over the phone if the participant has adequate access to their own phone and phone plan, or over secure video conferencing (Microsoft Teams or Enterprise Zoom) if the participant has adequate access to the necessary technology.

Participants will receive a one-time $25 gift card upon completion of the baseline survey in recognition of their time and effort. If participants complete the baseline survey over the phone, research staff will offer participants the chance to pick up the study gift card in-person at JYP or via mail if the participant has a mailing address.

Participants with a stable home address who complete enrollment and the baseline survey over the phone and who are not willing to travel to JYP to sign the ROI in person, will be given the option to receive a pre-stamped envelope with the ROI form to have them sign and mail back to our MGH or BHCHP office. Participants will be mailed the $25 gift card once the research coordinator receives a signed ROI.

Arm 2 participants will additionally have a series of ad hoc meetings with the LCS navigator as part of the intervention. These meetings may occur in person or over the phone. The navigator will be asked to log the date, time, and nature of activities carried out during these navigation sessions/meetings.

Due to the nature of the primary outcome, which will be ascertained via medical record review, no formal research follow-up visit is necessary. The screening, baseline, and outcome variables for the study are detailed in section VI.A.

40 participants in Arm 1 will participate in post-intervention interviews and be stratified according to whether they did (N=20) or did not (N=20) attain the primary outcome of completing a lung screening CAT scan within six months (26 weeks) of initial enrollment. Participants who agree to participate will go through a separate verbal consent process as illustrated previously. Interviews will occur after at least 6 months of exposure to the navigation intervention or sooner if the navigation intervention was terminated due to the participant completing lung cancer screening and requiring no additional follow-up or support. The interviews will last up to 45 minutes and be audio recorded. The selection of qualitative participants following ascertainment of the primary quantitative outcome avoids introducing a treatment effect from the interviews while remaining temporally close enough to the LCS process to facilitate accurate recall. These 40 participants will receive a one-time $25 gift card upon completion of the interview in recognition of their time and effort.

Additionally, we will interview 10 BHCHP PCPs among those who had at least 1 patient enrolled in the trial. Providers who agree to participate will provide verbal consent as illustrated previously. Interviews will last up to 45 minutes and be audio recorded. Interviews will occur following completion of the trial (at least 6 months after the last patient participant is enrolled) to avoid influencing provider practice around LCS ordering and follow-up. The 10 PCPs will receive a one-time $25 gift card upon completion of the interview in recognition of their time and effort.

Study personnel will follow various safety precautions to prevent COVID-19 transmission. For patients that are screened for eligibility over the phone and interested in scheduling an in-person study visit, study personnel will administer a screening instrument for COVID-19 symptoms and recent positive COVID-19 tests within 72 hours prior to the scheduled in-person study visit. Prior to entering BHCHP clinic buildings, per BHCHP policy, all patients are already

required to wear a face mask and be screened for COVID-19 symptoms and recent positive COVID-19 tests at the door. If BHCHP ceases these screening procedures, study personnel will implement the same screening measures at the start of any in-person visit and will continue to require participants to wear a face mask while engaging with study staff. If a patient endorses any COVID-19 symptoms, study personnel will immediately cease study activities and notify a clinic staff of the presence of a symptomatic patient.

If rising COVID-19 rates necessitate temporary restrictions on all in-person study activities at BHCHP facilities, we will transition to remotely conducting as many study activities as possible until the restrictions are lifted. Study personnel will be able to screen, perform verbal informed consent, conduct the baseline survey, perform qualitative interviews, and provide navigation services over the phone or by the HIPAA-compliant video conferencing platforms Microsoft Teams or Enterprise Zoom depending on patient preference and access to technology (i.e., owning a device with adequate minutes or video conferencing capabilities). Study personnel will always follow the MGB requirements for using Teams or Zoom with no study visits or activities being audio- or video-recorded within the Teams or Zoom platform. Microsoft Teams will be the preferred platform to use for video call study visits, but in the event of technical difficulties, study personnel will use Enterprise Zoom as a backup platform.

B. Drugs

While the LCS navigator will assist participants who currently smoke with accessing smoking cessation resources, neither the navigator nor study staff will provide participants with medications. Participants in both arms may choose to seek smoking cessation medications through their BHCHP PCP or other health care provider.

C. Devices

No devices will be used in this study.

D. Procedures

There are no procedures or surgical interventions in this study. Participants may elect to undergo LCS with LDCT after consultation with their PCP (and with the LCS navigator, for those in Arm 2), but the ordering and oversight of LDCTs will be handled by BHCHP PCPs as per usual practice for participants in both study arms.

E. Data Collection

We will collect all study data electronically using Partners-approved laptops or tablet devices with full disk encryption. Participant data will be recorded, saved, and managed using the Partners-hosted Research Electronic Data Capture (REDCap) program with data collection forms custom-designed for this study. We will also use BHCHP and MGB EHR to collect information from participants' medical records that relates to their general health history, any charted mental health problems, and any other information that directly relates to lung cancer screening and smoking. The specific data elements collected at each study visit are detailed below in section VI.A.

VI. BIOSTATISTICAL ANALYSIS

A. Specific data variables

1) Screening variables: Screening variables will include self-reported measures related to the inclusion and exclusion criteria for study participation. These will include the following variables, assessed as shown in Appendix A:
- First and last name
- Date of birth
- Age
- Preferred language
- BHCHP PCP
- Preferred BHCHP clinic site
- History of homelessness
- History or symptoms of lung cancer
- Recent non-lung cancer or cancer treatment
- Home oxygen use
- Cigarette smoking history

2) Contact form variables: Enrolled participants will be asked to complete a contact information form. This information will be valuable for following up with participants who cannot complete the baseline survey on the same day as enrollment, as well as for the patient navigator to aid with patient outreach and EHR documentation. This form will include the following variables, assessed as shown in Appendix O:
- BHCHP Epic MRN (entered by the research coordinator from patient's EHR)
- Last 4 digits of social security number
- Phone number (if applicable)
- Address/location of usual nighttime residence
- Alternative contact (who may know of participant's whereabouts)
- Contact in the case of an emergency
- Participant photograph (with participant permission) to facilitate subsequent contact with patient navigator

3) Baseline variables: Enrolled participants will be asked to complete a baseline assessment (Appendix D) of variables mapping to core domains of the Health Belief Model in addition to other key factors that could impact one or more dimensions of the Health Belief Model, as displayed in Table 2.

4) Electronic health record variables: The following information will be collected from the review of patients' BHCHP and MGB electronic health records:
- History of care with BHCHP and BHCHP PCP prior to study enrollment
- Comorbid health conditions.
- Documentation of a shared decision making (SDM) appointment with PCP to discuss lung cancer screening.
- Documentation of referrals for lung CT scans.
- Documentation of completion of lung CT scans
- Documentation of follow-up appointments with PCP that address results of lung cancer screening.
- Documented smoking status (and any changes in smoking status during study enrollment).
- Documented prescriptions to any smoking cessation medications
- Documented insurance status and insurance type.

Table 2. Baseline measures of study participants corresponding to Health Belief Model (HBM) domains and other key factors.

HBM domains	Measures
**Sociodemographic factors**	Age; sex; race/ethnicity; education; (all based on items from the 2003 HCH User Survey[23,84] and our prior survey work with homeless individuals[33,34]); homelessness onset, episodes, and duration[1]; current living situation
**Perceived lung cancer susceptibility/severity**	Perceived risk of lung cancer and smoking, perceived severity of lung cancer, worry about lung cancer, (all developed by Dr. Park for NLST)[76,85];
**Cues for LCS**	Prior LCS recommendation by health care provider; prior exposure to LCS brochures, flyers, posters, or advertisements
**Perceived LCS benefits/barriers**	Lung cancer screening knowledge[86]; perceived benefits of quitting smoking[76,85], comfort navigating hospital/health system; health insurance status[23,84]; competing priorities for meeting subsistence needs[22]
**Self-efficacy for LCS**	General self-efficacy scale[87,88]; self-efficacy for completing LDCT
**Other key factors**	**Measures**
**Tobacco use**	Duration of smoking; past quit attempts; current cigarettes/day; alternative tobacco use[35,89,90], Fagerstrom Test for Nicotine Dependence[91]; stage of change[92,93]; Contemplation Ladder[94]
**COVID-19**	Comfort navigating hospital/attending medical appointment during COVID-19 outbreak[95-97]; Previous history of COVID-19 And Lung Infection/Pneumonia
**Comorbid conditions**	Alcohol Use Disorders Identification Test-Concise (AUDIT-C)[98,99];! Drug Abuse Screening Test (DAST-10)[100,101], and K6 screen for psychological distress[102,103]; single-item general health status (SF-1)[104,105]

3) Navigation log: The navigator will complete a log (Appendix E) capturing the following information each time he/she interacts with a participant in Arm 2.
- Name of study staff who completes navigation activity
- Date and time
- Participant ID number
- Who was contacted for navigation activity
- Contact modality for navigation activity
- Tasks/activities undertaken during encounter

4) Qualitative interview data elements:

Participant interviews: Participants will be asked to articulate their thought process in considering whether to pursue LCS and to identify the factors that most shaped their decision,

including logistical, physical, or emotional barriers and facilitators. Based on our Health Belief Model framework (Table 2), we will prompt participants to consider the impact of the navigator on a) altering their perceived susceptibility to lung cancer, b) providing them with cues or reminders to pursue LCS, c) enhancing their self-efficacy for pursuing LCS, and d) helping them to overcome barriers to completing LCS. Among participants who undergo LDCT, we will assess their perspectives on the ease of the process, its impact on lung cancer worry, perceived lung cancer risk, and smoking behavior, and the need for any follow-up testing.

PCP interviews: These interviews will elicit PCP perspectives on the challenges of implementing LCS guidelines in primary care practice for a vulnerable population and probe the ways in which the navigator did or did not help to reduce these challenges. We will also seek feedback from PCPs on ways to strengthen the integration of patient navigators into clinical care teams.

B. Study Endpoints

1) Primary outcome: The primary outcome is receipt of LDCT for LCS at 6 months (26 weeks). This will be based on radiology records verifying that a chest CT was performed for LCS and interpreted according to the Lung-RADS framework.[80] To ascertain this outcome, study staff will examine available health records from BHCHP, MGB, and any other institution for which the participant signs a release of information, as described above in section IV.C. At 6 months (26 weeks) after enrollment, radiology records will be requested/obtained from these facilities. Most Boston-area hospital records are accessible through Care Everywhere in the BHCHP version of Epic and through MGB Epic, supporting the feasibility of gathering this information in an efficient manner. This approach does not rely on participant self-report and therefore does not require in-person or phone follow-up to ascertain. For those verified to have undergone LDCT for LCS within the 6 month (26-week) timeframe, the Lung-RADS result along with any abnormal or incidental findings will be recorded.

2) Secondary outcome: Increasing LDCT completion will only reduce lung cancer mortality if individuals with abnormal results can undergo evaluation and treatment of early stage lung cancer in a timely fashion. In the setting of homelessness, a valid concern is whether participants with abnormal LDCT results can be recalled for diagnostic follow-up in a timely manner. To address this concern, our secondary outcome is receipt of LDCT with timely diagnostic follow-up of any abnormal results. To satisfy this composite outcome, participants must achieve the primary outcome (LDCT at 6 months (26 weeks) and, if the result is abnormal, also obtain the next recommended follow-up test within 1 month (4 weeks) of the advised timeframe, based on the Lung-RADS framework.[80] Lung-RADS category descriptions, recommended follow-up, associated probabilities of malignancy, and estimated general population prevalences are shown in Table 3.[104] As described above, we will obtain radiology records for participants who underwent LDCT for LCS to document the findings of the study, determine the Lung-RADS category associated with those findings, and ascertain the recommended diagnostic follow-up plan. Participants with Lung-RADS 1-2 findings do not require specific diagnostic action, but rather may continue with

Table 3. Lung-RADS categories.

Category	Description	Management	Malignancy probability	Gen. pop. prevalence
1	Negative	Repeat LDCT 12 mos	<1%	90%
2	Benign			
3	Probably benign	Repeat LDCT 6 mos	1-2%	5%
4A	Suspicious	Repeat LDCT 3 mos +/- PET/CT	5-15%	2%
4B/X		Chest CT, PET/CT, +/- biopsy	>15%	2%

*Adapted from the American College of Radiology

annual screening at 12 months. These individuals will automatically satisfy the secondary outcome. In contrast, Lung-RADS 3-4 findings signal an increased risk for malignancy and merit sooner-than-annual follow-up testing. These individuals will satisfy the secondary outcome only if they obtain the next recommended diagnostic study within 1 month (4 weeks) of the recommended timeframe. For example, if a participant has a Lung-RADS 3 finding for which follow-up LDCT in 6 months is recommended, we will assess whether this follow-up test occurred within 7 months (30 weeks) of the initial LDCT. If a participant has a Lung-RADS 4B/X finding suggesting a high suspicion for malignancy, we will assess whether the recommended follow-up testing occurred within 1 month (4 weeks) of the initial LDCT. The offer of delayed navigation to control arm participants will not interfere with this outcome because a) delayed navigation will be offered only to those who did not undergo an initial LCS LDCT within 6 months (26 weeks) (i.e. the primary outcome), and b) attaining the primary outcome is necessary for satisfying the secondary outcome.

3) Other outcomes: We will examine time to receipt of LDCT for LCS. Process outcomes include acceptance of navigation when offered, number of contacts with the navigator, and attendance of a LCS SDM visit with a BHCHP PCP. Tobacco-related outcomes include receipt of a prescription for any smoking cessation medication and changes in clinically-recorded smoking status (i.e. quitting among current smokers or relapsing among former smokers), both ascertained by BHCHP medical record review. We will track the frequency and types of abnormal findings detected on LCS LDCT, any additional diagnostic testing or therapeutic changes pursued in response to these findings, and the percentage of participants diagnosed with lung cancer (based on radiology and BHCHP and MGB records review). We will also track diagnostic chest CT scans obtained for reasons other than LCS.

C. Statistical Methods

Quantitative analysis: LDCT completion at 6 months (26 weeks) (1° outcome) and LDCT completion with timely diagnostic follow-up (2°outcome) are both binary outcomes. In the principal analysis, we will assess the difference between the navigation and usual care arms on attainment of these outcomes using the Chi-square test. We will also use logistic regression models to examine the associations between treatment condition and outcomes including clinical site and other known predictors of outcomes to increase the precision of effect estimates. Additional analyses will examine time to receipt of LDCT for LCS between study arms using time-to-event methods (log-rank tests and Cox regression models). Although we expect to be underpowered to detect significant interactions, we will examine whether the effect of navigation varies by smoking status, housing status, or clinical site in an exploratory analysis.

Qualitative analysis: Qualitative interviews will be audiotaped and transcribed. We will use inductive content analysis methods to identify major and minor themes, and we will use deductive methods to strengthen, refine, and enhance understanding of identified themes.[105] Qualitative data coding will be performed by 2 trained study staff members using NVivo 11 software. The data coders will meet regularly with Drs. Baggett and Park to discuss the structural thematic framework, categories, and coding plan. We will resolve discrepancies through discussion and comparison to raw data. Coding will continue iteratively until a high level of reliability (Kappa>0.80) is established.

Integration with quantitative data: Based on a mixed methods approach,[106] we will use the qualitative interview data to interpret and explain the quantitative observations from the trial. This will aid in better understanding why overall LDCT completion was higher or lower than

expected and why the navigation intervention did or did not improve these outcomes. Following from our stratified sampling scheme, we will integrate the quantitative and qualitative data by assessing for thematic differences in the interview content of participants who did (N=20) and did not (N=20) attain the primary outcome. We will focus in particular on comparing and contrasting these two groups' views on the utility of the navigator with respect to the Health Belief Model domains of perceived susceptibility, cues, self-efficacy, and barriers, as described above. We will additionally incorporate the quantitative baseline measures tapping these Health Belief Model domains into integrated analyses. Table 4 illustrates the way in which these quantitative and qualitative data elements will be integrated and presented in a blended fashion.

Table 4. Proposed integration of quantitative and qualitative data.

Health Belief Model domains	Measurement (method)	LCS LDCT at 6 months	
		Yes (N=20)	No (N=20)
Perceived susceptibility / severity	Baseline survey (quan)	Lung cancer risk perception, severity, and worry scores	
	Post-intervention interview (qual)	Quotes/themes about navigator impact on perceived susceptibility/severity	
Cues	Baseline survey (quan)	Past provider recommendation for LCS, exposure to LCS information	
	Post-intervention interview (qual)	Quotes/themes about navigator provision of cues for LCS	
Self-efficacy	Baseline survey (quan)	General and LCS-specific self-efficacy scores	
	Post-intervention interview (qual)	Quotes/themes about navigator impact on self-efficacy	
Perceived benefits / barriers	Baseline survey (quan)	LCS knowledge, competing subsistence difficulty scores	
	Post-intervention interview (qual)	Quotes/themes about navigator assistance with barriers	

D. Power Analysis

The sample size justification is based on the primary outcome of LCS LDCT completion at 6 months (26 weeks). Internal BHCHP data suggests a presently negligible baseline rate of LCS LDCT. Allowing for improvement in this rate as LCS comes into wider practice, we will assume that 5% of usual care (Arm 1) participants will obtain an LDCT within 6 months (26 weeks). In our judgment, increasing this completion rate to 15% would represent a clinically meaningful change. We will have >80% power to detect such a difference with a sample size of 200 in the navigation arm (Arm 2) and 100 in the usual care arm (Arm 1).

VII. RISKS AND DISCOMFORTS

The potential risks and discomforts associated with this study are detailed below. The methods for mitigating these risks and monitoring the safety of participants are described in section IX.b.

A. Complications of surgical / non-surgical procedures

Not applicable.

B. Drug side effects and toxicities

Study staff will not be offering any drugs or over-the-counter products to participants.

C. Device complications

Not applicable.

D. Psychosocial (non-medical) risks

Potential risks to participants include psychological distress arising from the possibility of false-positive CT findings leading to additional diagnostic studies or procedures. Psychological and/or physical distress may also be brought about by discussing smoking and/or changing smoking behavior. Finally, because we will collect and record identifying information (e.g. name, date of birth, and social security number), there is a risk to privacy if the data are compromised. Section IX (Monitoring and Quality Assurance) describes our methods for safeguarding the data and privacy of participants. The potential risks of this study are reasonable in relation to the potential benefits of reducing the risk of dying from lung cancer.

E. Radiation risks

There is a slight physical risk associated with exposure to radiation during CT scanning, although the “low-dose” nature of the test is designed to help mitigate this risk. For this reason, LDCT LCS for individuals at increased risk of lung cancer is recommended by a variety of professional societies since the benefits of screening for early stage cancer vastly outweigh the risks presented by small radiation exposures.

VIII. POTENTIAL BENEFITS

A. Potential benefits to participating individuals

Potential benefits for participants include the opportunity to receive assistance in completing LCS, which has been associated with a 20% reduction in lung cancer mortality. Additionally, current smokers will be informed of and referred to various resources to help them quit smoking, which may confer numerous health and financial benefits.

B. Potential benefits to society

Considerable knowledge could be gained from this study. To our knowledge, this will be the first study to focus on LCS among homeless people and the first randomized trial of any cancer screening intervention in this population. By focusing on an understudied topic area pertaining to a common cause of death among homeless people, our study fills an important gap in the literature and could serve as a blueprint for improving cancer screening and outcomes in homeless health care settings, particularly in the 295 HCH programs that serve 890,000 people annually in the US.[107]

IX. MONITORING AND QUALITY ASSURANCE

A. Data monitoring

All data will be collected using password-protected laptops or tablets with full disk encryption. We will use the Partners-hosted Research Electronic Data Capture (REDCap) application for data collection and management. REDCap is a secure, HIPAA compliant web-based application hosted by Partners HealthCare Research Computing, Enterprise Research Infrastructure & Services (ERIS). Because a Partners username is required for logging in to REDCap, all activity on study documents is electronically logged and therefore traceable. All data will be collected according to a standardized protocol preprogrammed into REDCap, which has built-in functions

providing real-time data entry validation to help ensure accuracy and completeness. Each data collection form will have instructions or prompts for collecting the required data elements, and the response fields for each item will have appropriate ranges and formats to ensure that the data is entered in a valid way. Participants who agree to be photographed will have their photo taken using the same device on which the rest of the data is collected (encrypted laptop or tablet). The photograph will be uploaded immediately to the REDCap database and the original photograph file will be permanently deleted from the device.

The PI will review all study data weekly to monitor its integrity. Additionally, the PI, biostatistician, and/or data analyst will download the study data on a weekly basis from the Partners REDCap server and back it up to the PI's SFA on the Partners network, which is protected by the Partners IS firewall, backed up nightly, and accessible only to authorized study staff. Only the minimum necessary number of study staff will have access to this data. Data analyses will be conducted by authorized study staff on the data files residing on the PI's Partners SFA. The data will not be transferred to investigators outside the Partners system.

The qualitative interviews conducted post-intervention will be recorded by a microphone connected directly to a password-protected, Partners-approved laptop or tablet with full-disk encryption. Audio files will be backed up to the PI's SFA described above and then deleted from the portable device.

If study activities are administered to participants using Enterprise Zoom or Microsoft Teams, personnel will always follow the MGB requirements for using video conferencing for research activities with no study visits or activities being audio- or video-recorded, with the exception of qualitative interviews, which will be audio-recorded with an encrypted laptop or tablet microphone (and not via the audio-recording capabilities within the Zoom or Teams platform) as described above. Study personnel will always follow the Principles of Privacy and share the minimum necessary amount of information.

B. Safety monitoring

Because of the narrow range of risks of the proposed study, there will not be a Data and Safety Monitoring Board for this trial. Instead, the safety of participants will be monitored by the PI on a continual basis. Navigators will stay in close contact with BHCHP PCPs and clinical staff to ensure the safety and well-being of all participants.

To minimize the risk to privacy, we will adhere to the data management procedures described above to help ensure the safety and integrity of data collected about study participants. All identifying information will be saved securely within the REDCap database. Paper ROI forms will be scanned into the PI's SFA and paper copies retained in a locked cabinet in the PI's office. Only the minimum necessary number of study staff will have access to this data. At the conclusion of the study, personal identifiers will be removed from the dataset and all analyses will occur in a de-identified fashion.

To help ensure the safety of participants, adverse events monitoring will be handled in the manner described below.

C. Outcomes monitoring

Because of the low-risk nature of the intervention, we will not conduct a formal interim analysis of the data nor define rules for early stopping of the trial.

D. Adverse event reporting guidelines

We will adhere to the Partners policy statement on "Reporting Unanticipated Problems including Adverse Events." Consistent with this policy, an adverse event will be defined as "any untoward or unfavorable medical occurrence in a human subject, including any abnormal sign, symptom, or disease, temporally associated with the subject's participation in the research, whether or not considered related to the subject's participation in the research." The PI and his designated study staff will be responsible for the monitoring of adverse events in study participants.

Adverse events reported to the PI or other study staff will be documented in an adverse event report containing a description of the event, the date and time of onset, the date and time of resolution, the expectedness of the event, the relationship to the study, the seriousness of the event, and the action taken in response to the event. An adverse event will be deemed unexpected if the nature, severity, or frequency of the event is not consistent with a known or foreseeable risk given the research procedures, the characteristics of the subject population, or with an expected progression of an underlying condition. The following attribution scale will be used to describe the relationship of the adverse event to the study protocol: unrelated to the protocol, or possibly, probably, or definitely related to the protocol. A serious adverse event will be defined as an adverse event that results in death, is life-threatening, requires hospitalization, causes persistent or significant disability, or requires medical or surgical intervention.

A summary safety report detailing all adverse events and their handling will be included in annual study progress reports to the Partners IRB and the American Cancer Society. Unexpected adverse events of any severity that are at least possibly related to the research and suggest the research puts participants at increased harm will be reported to the Partners IRB within 5 working days or 7 calendar days. Serious adverse events that are unexpected and at least possibly related to the research will be assumed to suggest that the research puts participants at increased harm and will be reported to the Partners IRB within the same time frame. Any action resulting in a temporary or permanent suspension of this study will be reported to the American Cancer Society's program official. Adverse event reports and annual safety summaries will be documented by study ID number; personal identifiers will not be included in these reports.

E. Protocol adherence

1) Research staff: The study coordinators will be trained on the data collection protocols described above. Their understanding of these protocols will be assessed by asking them to demonstrate competency in using the REDCap data collection forms.

2) LCS navigator: To ensure adherence to the navigation intervention strategy, the navigator will receive in-depth training on patient navigation and focused training on tobacco cessation counseling.

a) Navigation training: The navigator will receive training in the core skills of patient navigation, including establishing rapport with patients while maintaining appropriate professional boundaries, motivational interviewing, performing an initial assessment with patients to explore barriers to LCS, working with patients to overcome these barriers, educating patients about the LCS process, introducing the concept of shared decision-making (SDM) around LCS, and empowering participants to discuss the benefits and risks of LCS with their PCP. Training will also encompass an overview of lung cancer, the rationale behind LCS, and

the practical steps involved in completing LDCT so that the navigator can better counsel patients on what to expect from the LCS process. Additionally, the navigator will be trained in identifying and managing logistical hurdles to completing LCS, such as insurance coverage lapses that may need to be remedied before LDCT is feasible. To facilitate timely follow-up of abnormal LDCT results, the navigator will received focused education on the American College of Radiology (ACR) Lung Imaging Reporting and Data System (Lung-RADS) framework.[80] Lung-RADS is a quality assurance tool designed to standardize LCS LDCT reporting and management recommendations, reduce confusion in LCS LDCT interpretations, and facilitate outcome monitoring.[80] The navigator training will be based on the protocol developed for Dr. Percac-Lima 's successful RCT of patient navigation for LCS among community health center patients (see section E). Dr. Percac-Lima, who has extensive experience in navigator training, will provide key components of this training and facilitate role playing sessions to reinforce skills. To the greatest extent possible, experienced navigators will be included in the training process. Depending upon prior experience, the navigator trainee will spend up to 2 weeks shadowing and observing an experienced navigator in the field prior to the start of the study. Refresher training and review of specific case scenarios will occur every 3 months throughout the study to ensure that the navigator is adhering faithfully to the intended roles and responsibilities.

b) Tobacco counseling training: The goal of the tobacco training component is to equip the navigator with the skills to provide a brief tobacco intervention. To achieve this goal, the navigator will complete an online 9-module course titled “Basic Skills for Working with Smokers,” sponsored by the University of Massachusetts Center for Tobacco Treatment Research & Training.[108] This course provides an introduction to the theory and practice of working with smokers in a health care setting. The navigator will additionally receive 4 hours of case-based teaching from Dr. Baggett on practical approaches to working with homeless smokers, followed by 6 hours of observing a certified Tobacco Treatment Specialist interact with and counsel smokers about tobacco cessation.

The navigator’s adherence to their intended duties will be assessed during weekly team meetings and through periodic audit of their navigation encounter logs.

X. REFERENCES

1. Burt MR, Aron LY, Douglas T, et al. Homelessness: Programs and the People They Serve: Findings of the National Survey of Homeless Assistance Providers and Clients: Technical Report. In. Washington, D.C.: U.S. Department of Housing and Urban Development, Office of Policy Development and Research; 1999.
2. Baggett TP, Chang Y, Porneala BC, Bharel M, Singer DE, Rigotti NA. Disparities in Cancer Incidence, Stage, and Mortality at Boston Health Care for the Homeless Program. *Am J Prev Med.* 2015;49(5):694-702.
3. Lamont DW, Toal FM, Crawford M. Socioeconomic deprivation and health in Glasgow and the west of Scotland--a study of cancer incidence among male residents of hostels for the single homeless. *J Epidemiol Community Health.* 1997;51(6):668-671.
4. Hwang SW, Wilkins R, Tjepkema M, O'Campo PJ, Dunn JR. Mortality among residents of shelters, rooming houses, and hotels in Canada: 11 year follow-up study. *BMJ.* 2009;339:b4036.
5. Baggett TP, Hwang SW, O'Connell JJ, et al. Mortality among homeless adults in Boston: shifts in causes of death over a 15-year period. *JAMA Intern Med.* 2013;173(3):189-195.
6. Baggett TP, Chang Y, Singer DE, et al. Tobacco-, alcohol-, and drug-attributable deaths and their contribution to mortality disparities in a cohort of homeless adults in Boston. *Am J Public Health.* 2015;105(6):1189-1197.
7. Aberle DR, Adams AM, Berg CD, et al. Reduced lung-cancer mortality with low-dose computed tomographic screening. *N Engl J Med.* 2011;365(5):395-409.
8. Chau S, Chin M, Chang J, et al. Cancer risk behaviors and screening rates among homeless adults in Los Angeles County. *Cancer Epidemiol Biomarkers Prev.* 2002;11(5):431-438.
9. Lebrun-Harris LA, Baggett TP, Jenkins DM, et al. Health status and health care experiences among homeless patients in federally supported health centers: findings from the 2009 patient survey. *Health services research.* 2013;48(3):992-1017.
10. Asgary R, Garland V, Jakubowski A, Sckell B. Colorectal cancer screening among the homeless population of New York City shelter-based clinics. *Am J Public Health.* 2014;104(7):1307-1313.
11. Asgary R, Garland V, Sckell B. Breast Cancer Screening Among Homeless Women of New York City Shelter-Based Clinics. *Womens Health Issues.* 2014.
12. Long HL, Tulsky JP, Chambers DB, et al. Cancer screening in homeless women: attitudes and behaviors. *J Health Care Poor Underserved.* 1998;9(3):276-292.
13. Baggett TP, Rigotti NA. Cigarette smoking and advice to quit in a national sample of homeless adults. *American Journal of Preventive Medicine.* 2010;39(2):164-172.
14. Connor SE, Cook RL, Herbert MI, Neal SM, Williams JT. Smoking cessation in a homeless population: there is a will, but is there a way? *Journal of General Internal Medicine.* 2002;17(5):369-372.
15. Snyder LD, Eisner MD. Obstructive lung disease among the urban homeless. *Chest.* 2004;125(5):1719-1725.
16. Szerlip MI, Szerlip HM. Identification of cardiovascular risk factors in homeless adults. *Am J Med Sci.* 2002;324(5):243-246.
17. Tsai J, Rosenheck RA. Smoking Among Chronically Homeless Adults: Prevalence and Correlates. *Psychiatr Serv.* 2012;63(6):569-576.
18. Torchalla I, Strehlau V, Okoli CT, Li K, Schuetz C, Krausz M. Smoking and predictors of nicotine dependence in a homeless population. *Nicotine Tob Res.* 2011;13(10):934-942.
19. Hahn JA, Kushel MB, Bangsberg DR, Riley E, Moss AR. BRIEF REPORT: the aging of the homeless population: fourteen-year trends in San Francisco. *J Gen Intern Med.* 2006;21(7):775-778.

20. Moyer VA. Screening for lung cancer: U.S. Preventive Services Task Force recommendation statement. *Ann Intern Med.* 2014;160(5):330-338.
21. Jenson TS, Chin J, Ashby L, Hermansen J, Hutter JD. Decision Memo for Screening for Lung Cancer with Low Dose Computed Tomography (LDCT) (CAG-00439N). . In. Baltimore, MD: Centers for Medicare & Medicaid Services; 2015.
22. Gelberg L, Gallagher TC, Andersen RM, Koegel P. Competing priorities as a barrier to medical care among homeless adults in Los Angeles. *Am J Public Health.* 1997;87(2):217-220.
23. Baggett TP, O'Connell JJ, Singer DE, Rigotti NA. The unmet health care needs of homeless adults: a national study. *Am J Public Health.* 2010;100(7):1326-1333.
24. Kushel MB, Vittinghoff E, Haas JS. Factors associated with the health care utilization of homeless persons. *JAMA.* 2001;285(2):200-206.
25. Weinstein LC, LaNoue M, Hurley K, Sifri R, Myers R. Using Concept Mapping to Explore Barriers and Facilitators to Breast Cancer Screening in Formerly Homeless Women with Serious Mental Illness. *J Health Care Poor Underserved.* 2015;26(3):908-925.
26. Fazel S, Khosla V, Doll H, Geddes J. The prevalence of mental disorders among the homeless in western countries: systematic review and meta-regression analysis. *PLoS Med.* 2008;5(12):e225.
27. Koegel P, Burnam MA, Farr RK. The prevalence of specific psychiatric disorders among homeless individuals in the inner city of Los Angeles. *Arch Gen Psychiatry.* 1988;45(12):1085-1092.
28. Brown RT, Kiely DK, Bharel M, Mitchell SL. Geriatric syndromes in older homeless adults. *J Gen Intern Med.* 2011;27(1):16-22.
29. Topolovec-Vranic J, Ennis N, Colantonio A, et al. Traumatic brain injury among people who are homeless: a systematic review. *BMC Public Health.* 2012;12:1059.
30. Topolovec-Vranic J, Ennis N, Howatt M, et al. Traumatic brain injury among men in an urban homeless shelter: observational study of rates and mechanisms of injury. *CMAJ Open.* 2014;2(2):E69-76.
31. Hwang SW, Colantonio A, Chiu S, et al. The effect of traumatic brain injury on the health of homeless people. *CMAJ.* 2008;179(8):779-784.
32. Oddy M, Moir JF, Fortescue D, Chadwick S. The prevalence of traumatic brain injury in the homeless community in a UK city. *Brain Inj.* 2012;26(9):1058-1064.
33. Baggett TP, Campbell EG, Chang Y, Magid LM, Rigotti NA. Posttraumatic Stress Symptoms and Their Association With Smoking Outcome Expectancies Among Homeless Smokers in Boston. *Nicotine Tob Res.* 2015.
34. Baggett TP, Rigotti NA, Campbell EG. Cost of Smoking among Homeless Adults. *N Engl J Med.* 2016;374(7):697-698.
35. Baggett TP, Campbell EG, Chang Y, Rigotti NA. Other tobacco product and electronic cigarette use among homeless cigarette smokers. *Addict Behav.* 2016;60:124-130.
36. Percac-Lima S, Ashburner JM, Bond B, Oo SA, Atlas SJ. Decreasing disparities in breast cancer screening in refugee women using culturally tailored patient navigation. *J Gen Intern Med.* 2013;28(11):1463-1468.
37. Percac-Lima S, Lopez L, Ashburner JM, Green AR, Atlas SJ. The longitudinal impact of patient navigation on equity in colorectal cancer screening in a large primary care network. *Cancer.* 2014;120(13):2025-2031.
38. Percac-Lima S, Ashburner JM, Zai AH, et al. Patient Navigation for Comprehensive Cancer Screening in High-Risk Patients Using a Population-Based Health Information Technology System: A Randomized Clinical Trial. *JAMA Intern Med.* 2016;176(7):930-937.
39. Freeman HP. Patient navigation: a community based strategy to reduce cancer disparities. *J Urban Health.* 2006;83(2):139-141.

40. Asgary R, Naderi R, Wisnivesky J. Opt-Out Patient Navigation to Improve Breast and Cervical Cancer Screening Among Homeless Women. *J Womens Health (Larchmt).* 2017.
41. Braun KL, Thomas WL, Jr., Domingo JL, et al. Reducing cancer screening disparities in medicare beneficiaries through cancer patient navigation. *J Am Geriatr Soc.* 2015;63(2):365-370.
42. Myers RE, Sifri R, Daskalakis C, et al. Increasing colon cancer screening in primary care among African Americans. *J Natl Cancer Inst.* 2014;106(12).
43. Honeycutt S, Green R, Ballard D, et al. Evaluation of a patient navigation program to promote colorectal cancer screening in rural Georgia, USA. *Cancer.* 2013;119(16):3059-3066.
44. Wang X, Fang C, Tan Y, Liu A, Ma GX. Evidence-based intervention to reduce access barriers to cervical cancer screening among underserved Chinese American women. *J Womens Health (Larchmt).* 2010;19(3):463-469.
45. Paskett ED, Harrop JP, Wells KJ. Patient navigation: an update on the state of the science. *CA Cancer J Clin.* 2011;61(4):237-249.
46. Phillips CE, Rothstein JD, Beaver K, Sherman BJ, Freund KM, Battaglia TA. Patient navigation to increase mammography screening among inner city women. *J Gen Intern Med.* 2010;26(2):123-129.
47. Robinson-White S, Conroy B, Slavish KH, Rosenzweig M. Patient navigation in breast cancer: a systematic review. *Cancer Nurs.* 2010;33(2):127-140.
48. Wells KJ, Battaglia TA, Dudley DJ, et al. Patient navigation: state of the art or is it science? *Cancer.* 2008;113(8):1999-2010.
49. Chen LA, Santos S, Jandorf L, et al. A program to enhance completion of screening colonoscopy among urban minorities. *Clin Gastroenterol Hepatol.* 2008;6(4):443-450.
50. Jandorf L, Braschi C, Ernstoff E, et al. Culturally targeted patient navigation for increasing african americans' adherence to screening colonoscopy: a randomized clinical trial. *Cancer Epidemiol Biomarkers Prev.* 2013;22(9):1577-1587.
51. Lasser KE, Murillo J, Medlin E, et al. A multilevel intervention to promote colorectal cancer screening among community health center patients: results of a pilot study. *BMC Fam Pract.* 2009;10:37.
52. Christie J, Itzkowitz S, Lihau-Nkanza I, Castillo A, Redd W, Jandorf L. A randomized controlled trial using patient navigation to increase colonoscopy screening among low-income minorities. *J Natl Med Assoc.* 2008;100(3):278-284.
53. Jandorf L, Gutierrez Y, Lopez J, Christie J, Itzkowitz SH. Use of a patient navigator to increase colorectal cancer screening in an urban neighborhood health clinic. *J Urban Health.* 2005;82(2):216-224.
54. Lasser KE, Murillo J, Lisboa S, et al. Colorectal cancer screening among ethnically diverse, low-income patients: a randomized controlled trial. *Archives of internal medicine.* 2011;171(10):906-912.
55. Horne HN, Phelan-Emrick DF, Pollack CE, et al. Effect of patient navigation on colorectal cancer screening in a community-based randomized controlled trial of urban African American adults. *Cancer Causes Control.* 2015;26(2):239-246.
56. Percac-Lima S, Grant RW, Green AR, et al. A culturally tailored navigator program for colorectal cancer screening in a community health center: a randomized, controlled trial. *J Gen Intern Med.* 2009;24(2):211-217.
57. Hoffman HJ, LaVerda NL, Young HA, et al. Patient navigation significantly reduces delays in breast cancer diagnosis in the District of Columbia. *Cancer Epidemiol Biomarkers Prev.* 2012;21(10):1655-1663.

58. Markossian TW, Darnell JS, Calhoun EA. Follow-up and timeliness after an abnormal cancer screening among underserved, urban women in a patient navigation program. *Cancer Epidemiol Biomarkers Prev.* 2012;21(10):1691-1700.
59. Battaglia TA, Bak SM, Heeren T, et al. Boston Patient Navigation Research Program: the impact of navigation on time to diagnostic resolution after abnormal cancer screening. *Cancer Epidemiol Biomarkers Prev.* 2012;21(10):1645-1654.
60. Raich PC, Whitley EM, Thorland W, Valverde P, Fairclough D. Patient navigation improves cancer diagnostic resolution: an individually randomized clinical trial in an underserved population. *Cancer Epidemiol Biomarkers Prev.* 2012;21(10):1629-1638.
61. Ramirez AG, Perez-Stable EJ, Penedo FJ, et al. Navigating Latinas with breast screen abnormalities to diagnosis: the Six Cities Study. *Cancer.* 2013;119(7):1298-1305.
62. Lee JH, Fulp W, Wells KJ, Meade CD, Calcano E, Roetzheim R. Patient navigation and time to diagnostic resolution: results for a cluster randomized trial evaluating the efficacy of patient navigation among patients with breast cancer screening abnormalities, Tampa, FL. *PLoS One.* 2013;8(9):e74542.
63. Lee JH, Fulp W, Wells KJ, Meade CD, Calcano E, Roetzheim R. Effect of patient navigation on time to diagnostic resolution among patients with colorectal cancer-related abnormalities. *J Cancer Educ.* 2014;29(1):144-150.
64. American Cancer Society. Patient Navigators Help Cancer Patients Manage Care. https://www.cancer.org/latest-news/navigators-help-cancer-patients-manage-their-care.html. Published February 24, 2017. Accessed March 1, 2017.
65. National Cancer Institute. Patient Navigation Research Program (PNRP). U.S. Department of Health and Human Services, National Institutes of Health, National Cancer Institute. https://www.cancer.gov/about-nci/organization/crchd/disparities-research/pnrp. Accessed March 28, 2017.
66. O'Connell JJ, Oppenheimer SC, Judge CM, et al. The Boston Health Care for the Homeless Program: a public health framework. *Am J Public Health.* 2010;100(8):1400-1408.
67. Bauer LK, Brody JK, Leon C, Baggett TP. Characteristics of Homeless Adults Who Died of Drug Overdose: A Retrospective Record Review. *J Health Care Poor Underserved.* 2016;27(2):846-859.
68. Bharel M, Lin WC, Zhang J, O'Connell E, Taube R, Clark RE. Health care utilization patterns of homeless individuals in Boston: preparing for Medicaid expansion under the Affordable Care Act. *Am J Public Health.* 2013;103 Suppl 2:S311-317.
69. Tsai D. MassHealth Transmittal Letter PHY-147. In: Office of Medicaid, Massachusetts Department of Health and Human Services; March 2016.
70. Wender R, Fontham ET, Barrera E, Jr., et al. American Cancer Society lung cancer screening guidelines. *CA Cancer J Clin.* 2013;63(2):107-117.
71. Detterbeck FC, Mazzone PJ, Naidich DP, Bach PB. Screening for lung cancer: Diagnosis and management of lung cancer, 3rd ed: American College of Chest Physicians evidence-based clinical practice guidelines. *Chest.* 2013;143(5 Suppl):e78S-92S.
72. Jaklitsch MT, Jacobson FL, Austin JH, et al. The American Association for Thoracic Surgery guidelines for lung cancer screening using low-dose computed tomography scans for lung cancer survivors and other high-risk groups. *J Thorac Cardiovasc Surg.* 2012;144(1):33-38.
73. Samet JH, Crowell R, Estepar RSJ, et al. Providing Guidance on Lung Cancer Screening to Patients and Physicians: An Update from the American Lung Association Lung Cancer Screening Committee. . In. Chicago: American Lung Association; April 30, 2015.

74. Bach PB, Mirkin JN, Oliver TK, et al. Benefits and harms of CT screening for lung cancer: a systematic review. *JAMA.* 2012;307(22):2418-2429.
75. Park ER, Streck JM, Gareen IF, et al. A qualitative study of lung cancer risk perceptions and smoking beliefs among national lung screening trial participants. *Nicotine Tob Res.* 2014;16(2):166-173.
76. Park ER, Ostroff JS, Rakowski W, et al. Risk perceptions among participants undergoing lung cancer screening: baseline results from the National Lung Screening Trial. *Ann Behav Med.* 2009;37(3):268-279.
77. Ryan H, Trosclair A, Gfroerer J. Adult current smoking: differences in definitions and prevalence estimates--NHIS and NSDUH, 2008. *J Environ Public Health.* 2012;2012:918368.
78. Health Resources and Services Administration, Bureau of Primary Health Care. Authorizing Legislation of the Health Center Program: Section 330 of the Public Health Service Act (42 USCS § 254b). http://bphc.hrsa.gov/policiesregulations/legislation/authorizing330.pdf. Accessed March 28, 2017.
79. U.S. Department of Housing and Urban Development. S. 896 HEARTH Act of 2009. https://www.hudexchange.info/resource/1717/s-896-hearth-act/. Accessed March 12, 2017.
80. American College of Radiology. Lung CT Screening Reporting and Data System (Lung-RADS). http://www.acr.org/Quality-Safety/Resources/LungRADS. Accessed March 28, 2017.
81. Fiore MC, Jaen CR, Baker TB, et al. Treating Tobacco Use and Dependence: 2008 Update. Clinical Practice Guideline. . In. Rockville, MD: U.S. Department of Health and Human Services. Public Health Service; May 2008.
82. Park ER, Gareen IF, Japuntich S, et al. Primary Care Provider-Delivered Smoking Cessation Interventions and Smoking Cessation Among Participants in the National Lung Screening Trial. *JAMA Intern Med.* 2015;175(9):1509-1516.
83. Poghosyan H, Kennedy Sheldon L, Cooley ME. The impact of computed tomography screening for lung cancer on smoking behaviors: a teachable moment? *Cancer Nurs.* 2012;35(6):446-475.
84. Greene J, Fahrney K, Byron M. Health Care for the Homeless User/Visit Surveys, RTI Project Number 07147.021. . In. Research Triangle Park, NC: RTI International; 2004.
85. Kaufman AR, Koblitz AR, Persoskie A, et al. Factor Structure and Stability of Smoking-Related Health Beliefs in the National Lung Screening Trial. *Nicotine Tob Res.* 2016;18(3):321-329.
86. Lowenstein LM, Richards VF, Leal VB, et al. A brief measure of Smokers' knowledge of lung cancer screening with low-dose computed tomography. *Prev Med Rep.* 2016;4:351-356.
87. Schwarzer R, Jerusalem M. Generalized Self-Efficacy scale. In: Weinman J, Wright S, Johnston M, eds. *Measures in health psychology: A user's portfolio. Causal and control beliefs.* Windsor, UK: NFER-NELSON; 1995:35-37.
88. Scholz U, Gutierrez Dona B, Sud S, Schwarzer R. Is General Self-Efficacy a Universal Construct? Psychometric Findings from 25 Countries. *European Journal of Psychological Assessment.* 2002;18(3):242-251.
89. US Department of Commerce CB. National Cancer Institute and Food and Drug Administration co-sponsored Tobacco Use Supplement to the Current Population Survey. In. *2018-2019*2020.
90. Kish DH, Reitzel LR, Kendzor DE, Okamoto H, Businelle MS. Characterizing Concurrent Tobacco Product Use Among Homeless Cigarette Smokers. *Nicotine Tob Res.* 2014.

91. Heatherton TF, Kozlowski LT, Frecker RC, Fagerstrom KO. The Fagerstrom Test for Nicotine Dependence: a revision of the Fagerstrom Tolerance Questionnaire. *Br J Addict.* 1991;86(9):1119-1127.
92. DiClemente CC, Prochaska JO, Fairhurst SK, Velicer WF, Velasquez MM, Rossi JS. The process of smoking cessation: an analysis of precontemplation, contemplation, and preparation stages of change. *J Consult Clin Psychol.* 1991;59(2):295-304.
93. Snow MG, Prochaska JO, Rossi JS. Stages of change for smoking cessation among former problem drinkers: a cross-sectional analysis. *J Subst Abuse.* 1992;4(2):107-116.
94. Biener L, Abrams DB. The Contemplation Ladder: validation of a measure of readiness to consider smoking cessation. *Health Psychol.* 1991;10(5):360-365.
95. Wong LE, Hawkins JE, Murrell KL. Where are all the patients? Addressing Covid-19 fear to encourage sick patients to seek emergency care. *NEJM Catalyst Innovations in Care Delivery.* 2020.
96. Toolkit P. COVID-19 Community Response Survey Guidance. In:2020.
97. (C3PNO) CCoCPNO. COVID-19 Module for NIDA-Supported Cohorts Affected by HIV and Substance Use. In:2020.
98. Bush K, Kivlahan DR, McDonell MB, Fihn SD, Bradley KA. The AUDIT alcohol consumption questions (AUDIT-C): an effective brief screening test for problem drinking. *Archives of internal medicine.* 1998;158(16):1789-1795.
99. Bradley KA, Bush KR, Epler AJ, et al. Two brief alcohol-screening tests From the Alcohol Use Disorders Identification Test (AUDIT): validation in a female Veterans Affairs patient population. *Archives of internal medicine.* 2003;163(7):821-829.
100. Skinner HA. The drug abuse screening test. *Addictive behaviors.* 1982;7(4):363-371.
101. Yudko E, Lozhkina O, Fouts A. A comprehensive review of the psychometric properties of the Drug Abuse Screening Test. *Journal of substance abuse treatment.* 2007;32(2):189-198.
102. Kessler RC, Andrews G, Colpe LJ, et al. Short screening scales to monitor population prevalences and trends in non-specific psychological distress. *Psychological medicine.* 2002;32(6):959-976.
103. Kessler RC, Barker PR, Colpe LJ, et al. Screening for serious mental illness in the general population. *Arch Gen Psychiatry.* 2003;60:184-189.
104. American College of Radiology. Lung-RADS Version 1.0 Assessment Categories. Available at http://www.acr.org/Quality-Safety/Resources/LungRADS. In:April 28, 2014.
105. Elo S, Kyngas H. The qualitative content analysis process. *J Adv Nurs.* 2008;62(1):107-115.
106. Creswell JW, Plano Clark VL. Chapter 3: Choosing a Mixed Methods Design. In: *Designing and Conducting Mixed Methods Research.* 2nd ed. Los Angeles: Sage; 2011:53-106.
107. Health Resources and Services Administration, U.S. Department of Health & Human Services. 2015 Health Center Data: National Health Care for the Homeless Program Grantee Data. https://bphc.hrsa.gov/uds/datacenter.aspx?year=2015&fd=ho. Accessed March 16, 2017.
108. Center for Tobacco Treatment Research & Training, University of Massachusetts Medical School. Basic Skills for Working with Smokers - Online Course. http://www.umassmed.edu/tobacco/training/basicskills_online/. Accessed March 28, 2017.
