# Supplementary material for: Homelessness, Patient Navigation, and Lung Cancer Screening in a Health Center Setting: A Subgroup Analysis of a Randomized Clinical Trial
Source: JAMA Netw Open. 2025 Jul 17;8(7):e2519780. doi: 10.1001/jamanetworkopen.2025.19780 (PMC12272284; doi:10.1001/jamanetworkopen.2025.19780)
Supplement: Supplement 2. — eFigure 1. LCS LDCT Scan Attainment Within 6 Months, Comparing Participants Currently and Formerly Experiencing Homelessness Stratified by Study Group eFigure 2. LCS LDCT Scan Attainment Within 6 Months Among Participants With Stable or Unstable Housing Who Formerly Experienced Homelessness eTable 1. Characteristics of Study Participants by Baseline Homelessness Status, With Individuals Who Formerly Experienced Homelessness Subdivided as Having Unstable or Stable Housing eTable 2. Navigation Activities Among Navigation Group Participants by Baseline Homelessness Status, With Individuals Who Formerly Experienced Homelessness Subdivided as Having Unstable or Stable Housing [file jamanetwopen-e2519780-s002.pdf]

Supplemental Online Content

Baggett TP, Sporn N, Barbosa Teixeira J, et al. Homelessness, patient navigation, and lung cancer screening in a health center setting: a subgroup analysis of a randomized clinical trial. *JAMA Netw Open*. 2025;8(7):e2519780. doi:10.1001/jamanetworkopen.2025.19780

eFigure 1. LCS LDCT Scan Attainment Within 6 Months, Comparing Participants Currently and Formerly Experiencing Homelessness Stratified by Study Group

eFigure 2. LCS LDCT Scan Attainment Within 6 Months Among Participants With Stable or Unstable Housing Who Formerly Experienced Homelessness

eTable 1. Characteristics of Study Participants by Baseline Homelessness Status, With Individuals Who Formerly Experienced Homelessness Subdivided as Having Unstable or Stable Housing

eTable 2. Navigation Activities Among Navigation Group Participants by Baseline Homelessness Status, With Individuals Who Formerly Experienced Homelessness Subdivided as Having Unstable or Stable Housing

This supplemental material has been provided by the authors to give readers additional information about their work.

© 2025 Baggett TP et al. *JAMA Network Open*.

eFigure 1. LCS LDCT Scan Attainment Within 6 Months, Comparing Participants Currently and Formerly Experiencing Homelessness Stratified by Study Group

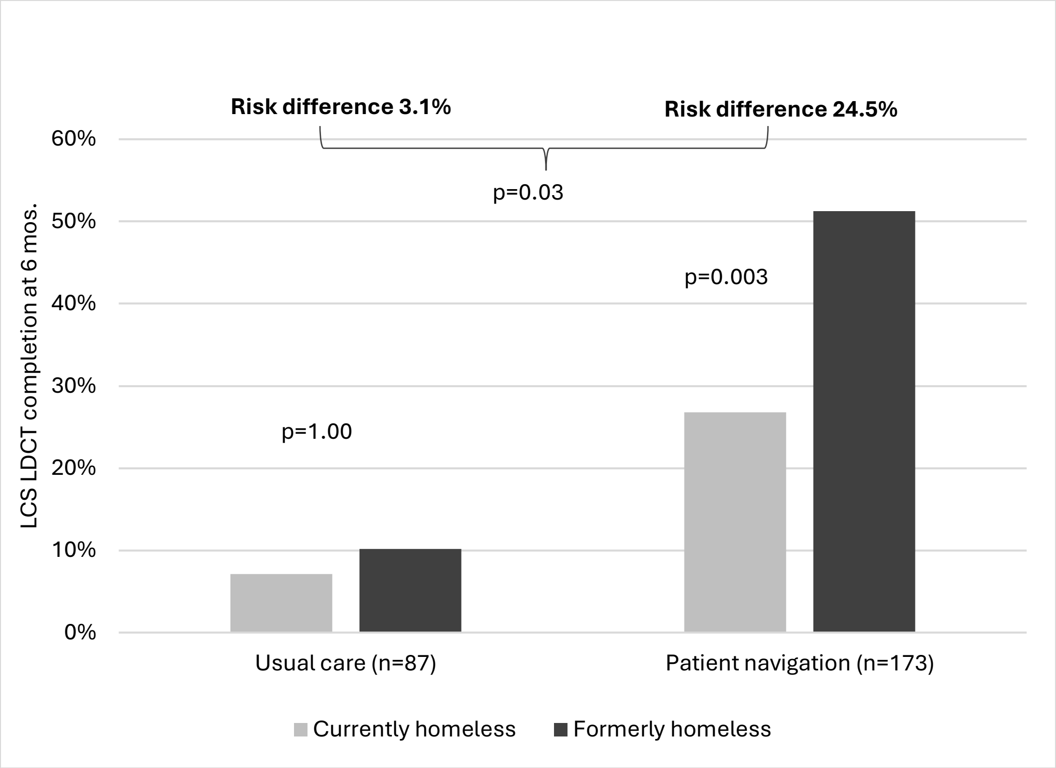

Abbreviations:
LCS, lung cancer screening
LDCT, low-dose computed tomography

© 2025 Baggett TP et al. *JAMA Network Open*.

eFigure 2. LCS LDCT Scan Attainment Within 6 Months Among Participants With Stable or Unstable Housing Who Formerly Experienced Homelessness

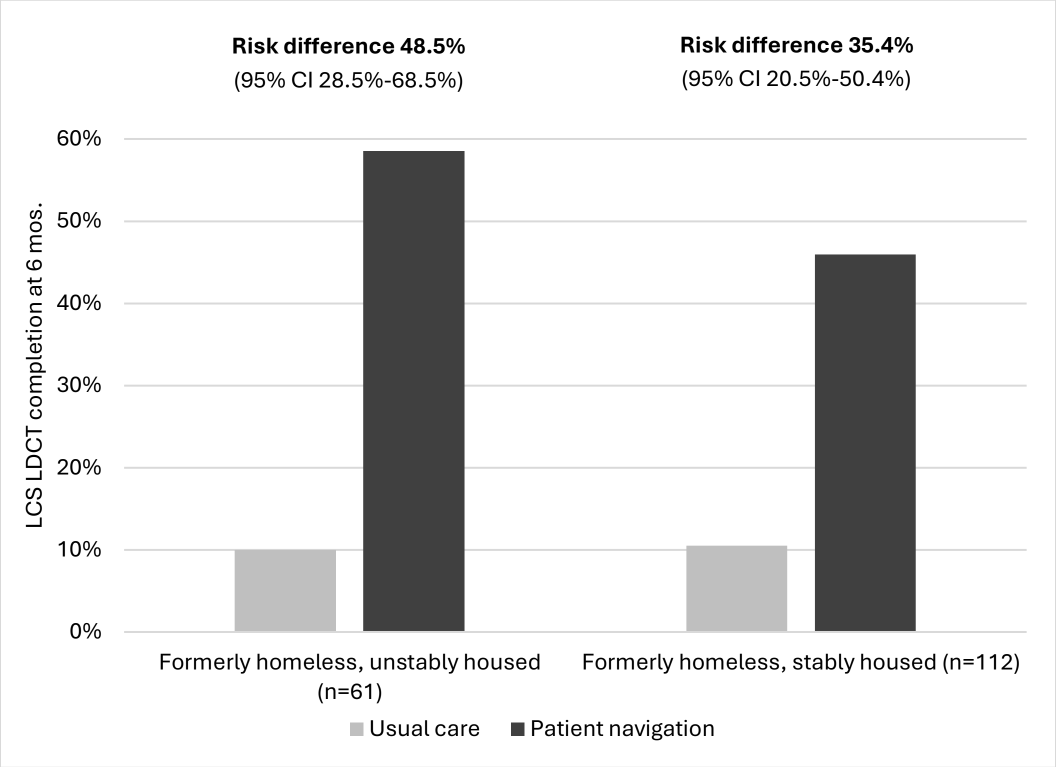

Abbreviations:
CI, confidence interval
LCS, lung cancer screening
LDCT, low-dose computed tomography

© 2025 Baggett TP et al. *JAMA Network Open*.

eTable 1. Characteristics of Study Participants by Baseline Homelessness Status, With Individuals Who Formerly Experienced Homelessness Subdivided as Having Unstable or Stable Housing[a]

	No. (%)		
**Characteristic**	**Currently homeless** (n=84)	**Formerly homeless, unstably housed** (n=61)	**Formerly homeless, stably housed** (n=112)
**Sociodemographic measures**			
Age, mean (SD)	58.9 (4.2)	60.4 (4.5)	61.7 (5.0)
Gender [b]			
Male	61 (72.6)	44 (72.1)	77 (68.8)
Female	22 (26.2)	17 (27.9)	34 (30.4)
Other	1 (1.2)	0	1 (0.9)
Race/ethnicity [c]			
Hispanic/Latine	17 (20.2)	4 (6.6)	17 (15.2)
Non-Hispanic Black	25 (29.8)	19 (31.1)	50 (44.6)
Non-Hispanic White	29 (34.5)	29 (47.5)	37 (33.0)
Non-Hispanic Other	12 (14.3)	7 (11.5)	7 (6.3)
High school graduate or GED [d]	32 (38.1)	27 (45.0)	60 (53.6)
Has a cell phone	62 (73.8)	61 (100)	110 (98.2)
Subsistence difficulties [e]			
None	20 (23.8)	33 (54.1)	85 (75.9)
Low	24 (28.6)	20 (32.8)	24 (21.4)
High	40 (47.6)	8 (13.1)	3 (2.7)
**General health measures**			
Health insurance	84 (100)	61 (100)	111 (99.1)
Primary care location			
Program headquarters	53 (63.1)	46 (75.4)	83 (74.1)
Satellite site	31 (36.9)	15 (24.6)	29 (25.9)
Fair/poor health status	45 (53.6)	38 (62.3)	47 (42.0)
Mental health disorder [f]	33 (39.3)	17 (27.9)	25 (22.7)
Alcohol use disorder [g]	25 (30.5)	12 (19.7)	23 (21.1)
Drug use disorder [h]	28 (33.3)	18 (30.0)	25 (22.3)
General self-efficacy score (10-40), mean (SD) [i]	31.6 (5.7)	32.9 (4.7)	33.0 (5.8)
**Lung cancer risk and LCS-related measures**			
Smoking status			
Current	81 (96.4)	48 (78.7)	90 (80.4)
Former	3 (3.6)	13 (21.3)	22 (19.6)
Pack-years of smoking, mean (SD)	44.0 (19.8)	50.8 (19.4)	49.9 (19.1)
Prior discussion of LCS with PCP	24 (28.6)	24 (39.3)	48 (42.9)

© 2025 Baggett TP et al. *JAMA Network Open*.

Lung cancer risk perceptions, mean (SD)[j]			
	No. (%)		
**Characteristic**	**Currently homeless** (n=84)	**Formerly homeless, unstably housed** (n=61)	**Formerly homeless, stably housed** (n=112)
Perceived comparative risk (range 3-15)	9.6 (2.7)	9.9 (2.6)	10.0 (2.8)
Worry about lung cancer (range 2-8)	4.7 (2.0)	4.6 (1.6)	4.8 (2.0)
Perceived lung cancer severity (range 2-10)	9.4 (1.2)	9.0 (1.4)	9.3 (1.5)
Perceived LCS benefits (range 3-15)	8.3 (1.6)	8.1 (1.8)	8.2 (1.4)

Abbreviations:
SD, standard deviation
GED, general equivalency degree
LCS, lung cancer screening
PCP, primary care practitioner

Notes:
[a] See the manuscript for definitions of unstably and stable housing. Three formerly homeless participants could not have their housing situation further characterized due to missing data.
[b] "Other" includes participants who selected "Non-binary/genderqueer" (n=1) or "Other" (n=1).
[c] 4 participants had unknown race/ethnicity due to item non-response. "Other non-Hispanic" includes participants who reported not being of Hispanic or Latino descent and who selected "American Indian or Alaskan Native" race (n=3), "Other" race (n=8), or multiple races (n=15).
[d] 2 participants had unknown educational status.
[e] Categories based on summed responses to the 5-item RAND Course of Homelessness subsistence difficulty scale (range 0-15), where "none" is score=0, "low" is score=1-5, and "high" is score=6+.
[f] Defined as a score ≥13 on the 6-item Kessler Psychological Distress Scale (K6); 2 participants had unknown status due to item non-response.
[g] Defined as a score ≥3 for females and ≥4 for males and other genders on the Alcohol Use Disorders Identification Test – Concise (AUDIT-C); 5 participants had unknown status due to item non-response.
[h] Defined as a score ≥3 on the Drug Abuse Screening Test (DAST-10); 1 participant had unknown status due to item non-response.
[i] Higher scores represent greater self-efficacy.
[j] Perceived personal risk was assessed with 2 items with 5-point Likert-type response options (score range 2-10; n=258), perceived comparative risk was assessed with 3 items with 5-point Likert-type response options (score range 3-15; n=251), worry about lung cancer was assessed with 2 items with 4-point Likert-type response options (score range 2-8; n=260), perceived lung cancer severity was assessed with 2 items with 5-point Likert-type response options (score range 2-10; n=258), and perceived LCS benefits were assessed with 3 items with 5-point Likert-type response options (score range 3-15; n=231).

© 2025 Baggett TP et al. *JAMA Network Open*.

eTable 2. Navigation Activities Among Navigation Group Participants by Baseline Homelessness Status, With Individuals Who Formerly Experienced Homelessness Subdivided as Having Unstable or Stable Housing[a]

	No. (%)		
**Navigation activity**	**Currently homeless** (n=56)	**Formerly homeless, unstably housed** (n=41)	**Formerly homeless, stably housed** (n=74)
Established initial contact	50 (89.3)	41 (100.0)	70 (94.6)
No. contacts (per patient), mean (SD)	7.7 (8.7)	11.2 (8.5)	10.2 (8.3)
Phone contacts (per patient), mean (SD)	7.6 (8.7)	11.1 (8.2)	10.1 (8.3)
In-person contacts (per patient), mean (SD)	0.1 (0.5)	0.1 (0.4)	0.1 (0.5)
Provided LCS education/counseling	36 (64.3)	31 (75.6)	53 (71.6)
Facilitated LCS shared decision-making visit with PCP	41 (73.2)	32 (78.0)	55 (74.3)
Facilitated LCS referral	41 (73.2)	32 (78.0)	61 (82.4)
Scheduled/arranged LCS appointment	38 (67.9)	30 (73.2)	52 (70.3)
Reminded about LCS appointment	25 (44.6)	24 (58.5)	44 (59.5)
Rescheduled missed LCS appointment	16 (28.6)	11 (26.8)	22 (29.7)
Facilitated follow-up of LCS results	19 (33.9)	25 (61.0)	38 (51.4)
Facilitated additional studies/testing	3 (5.4)	5 (12.2)	5 (6.8)
Provided smoking cessation support	17 (30.4)	12 (29.3)	28 (37.8)
Addressed insurance issues	9 (16.1)	12 (29.3)	28 (37.8)
Facilitated transportation	6 (10.7)	8 (19.5)	13 (17.6)
Coordinated care with PCP	51 (91.1)	38 (92.7)	68 (91.9)
No. PCP contacts (per patient), mean (SD)	4.8 (4.5)	4.8 (3.9)	5.2 (4.0)
Provided non-LCS care coordination	19 (33.9)	18 (43.9)	25 (33.8)

Abbreviations:
SD, standard deviation
LCS, lung cancer screening
PCP, primary care practitioner

Notes:
[a] See the manuscript for definitions of unstably and stable housing. Two formerly homeless navigation arm participants could not have their housing situation further characterized due to missing data.

© 2025 Baggett TP et al. *JAMA Network Open*.
