# Supplementary material for: Homelessness, Patient Navigation, and Lung Cancer Screening in a Health Center Setting: A Subgroup Analysis of a Randomized Clinical Trial
Source: JAMA Netw Open. 2025 Jul 17;8(7):e2519780. doi: 10.1001/jamanetworkopen.2025.19780 (PMC12272284; doi:10.1001/jamanetworkopen.2025.19780)
Supplement: Supplement 3. — Data Sharing Statement [file jamanetwopen-e2519780-s003.pdf]

Data Sharing Statement

Baggett. Homelessness, Patient Navigation, and Lung Cancer Screening in a Health Center Setting. *JAMA Netw Open*. Published July 17, 2025.
doi:10.1001/jamanetworkopen.2025.19780

Data
Additional Information: ClinicalTrials.gov: NCT04308226
Data available: No

Additional Information
Explanation for why data not available: Data sharing parameters are outlined in the primary trial report.
